# Tandem C–H oxidation/cyclization/rearrangement and its application to asymmetric syntheses of (−)-brussonol and (−)-przewalskine *E*

**DOI:** 10.1038/ncomms8332

**Published:** 2015-06-17

**Authors:** Zhi-Wei Jiao, Yong-Qiang Tu, Qing Zhang, Wen-Xing Liu, Shu-Yu Zhang, Shao-Hua Wang, Fu-Min Zhang, Sen Jiang

**Affiliations:** 1 State Key Laboratory of Applied Organic Chemistry & College of Chemistry and Chemical Engineering, Lanzhou University, Lanzhou 730000, P. R. China; 2 Collaborative Innovation Center of Chemical Science and Engineering, Tianjin 300071, P. R. China

## Abstract

Natural products are a vital source of lead compounds in drug discovery. Development of efficient tandem reactions to build useful compounds and apply them to the synthesis of natural products is not only a significant challenge but also an important goal for chemists. Here we describe a tandem C–H oxidation/cyclization/rearrangement of isochroman-derived allylic silylethers, promoted by DDQ and InCl_3_. This method allows the efficient construction of tricyclic benzoxa[3.2.1]octanes with a wide substrate scope. We employ this tandem reaction to achieve the asymmetric total syntheses of (−)-brussonol and (−)-przewalskine *E*.

## Abstract (web summary)

The ability to form complex molecular architectures from simpler precursors is important for drug discovery and medicinal chemistry. Here, the authors report a cascade reaction giving access to tricyclic products and apply it in the total synthesis of two natural products.

## Graphical abstract

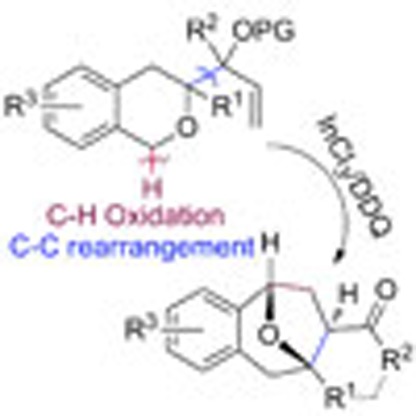

The strained tricyclic benzoxa[3.2.1]octane skeleton exists in numerous bioactive and pharmaceutical molecules such as przewalskone^1^ and brussonol^2^, and it is a useful building block in organic synthesis such as for producing platensimycin^3^ (Fig. 1a). In recent decades, the significant biological activity and potential pharmaceutical value of molecules with this skeleton have driven chemists to devise several methods for constructing it (Fig. 1b)^3^,^4^,^5^,^6^,^7^,^8^,^9^,^10^,^11^,^12^,^13^,^14^. Nevertheless, more efficient and practical methods are needed.

One possible approach is the C–C bond formation via direct (sp^3^) α-C–H bond functionalization, which is increasingly being used to synthesize complex N- or O-containing molecules^15^,^16^,^17^,^18^. While several reactions have been described to achieve C–C formation at the α-position in amines, only a handful of reactions have been reported for C–C formation in ethers^19^,^20^,^21^,^22^,^23^,^24^,^25^,^26^,^27^,^28^,^29^. In connection with our long-standing interest in α-C–H bond functionalization of ethers^30^,^31^ and in carbon–carbon rearrangement of allylic alcohol/silylether^32^,^33^,^34^, we speculated that it might be possible to construct the benzoxa[3.2.1]octane unit via a tandem reaction that is initiated by benzylic C–H oxidation of an isochroman-derived allylic silylether and triggered by C–C bond rearrangement.

Here we use 2,3-dichloro-5,6-dicyanobenzoquinone (DDQ) and InCl_3_ to promote tandem C–H oxidation/cyclization/rearrangement of isochroman-derived allylic silylether. We then apply this efficient tandem reaction to asymmetric syntheses of the bioactive natural products (−)-brussonol and (−)-przewalskine *E*.

## Results

### Reaction optimization

We began our efforts to generate the benzoxa[3.2.1]octane unit using the model substrate **1a,** prepared as a single diastereoisomer in a general procedure (see Supplementary Methods 1 and Supplementary Data 1 and 4). Initial experiment with the common oxidant DDQ (2.0 equiv.) as the sole promoter failed to give the desired product and resulted in fully recovery of **1a** (Table 1, entry 1). Next we tried combinations of DDQ (1.2 equiv.) and Lewis acids in the presence of 4-Å molecular sieve. Using FeCl_3_ or SnCl_4_ led to the decomposition of **1a** (entries 2 and 3), whereas using SnBr_4_ led to the consumption of 1**a** in 8 h and the desired product **2a** in 33% yield (entry 4). Cu(OTf)_2_ and LiClO_4_ also promoted this transformation, albeit in lower yield (entries 5 and 6). When 1.0 equiv. InCl_3_ was used, **2a** was obtained in 36% yield after 24 h and 15% of **1a** was recovered (entry 7). Increasing the load of DDQ to 2.0 equiv. gave higher yield (56%) in shorter time (4 h). Interestingly, decreasing the load of InCl_3_ to 0.1 equiv. further increased the yield of **2a** to 69% (entry 9). The molecular sieve was essential to this reaction, since omitting it led to only 30% yield (entry 10). With InCl_3_ as the catalyst, a side reaction in which **1a** was partially desilylated to give free allylic alcohol was always observed. To inhibit this, we screened several weakly basic additives (entries 11–14). We were pleased to find that using 2,6-dibromopyridine (DBP, 5.0 equiv.) significantly improved yield to 81% (entry 14). In addition, the reaction was also carried out in some other solvents (C_2_H_4_Cl_2_, CH_3_NO_2_, CH_3_CN, toluene, tetrahydrofuran (THF)) and oxidants (TEMPO, benzoquinone), but results were not better than with CH_2_Cl_2_ and DDQ. Therefore, the optimal conditions were defined to be DDQ (2.0 equiv.), InCl_3_ (0.1 equiv.), DBP (5.0 equiv.) and 4-Å molecular sieve in CH_2_Cl_2_ (entry 14, see Supplementary Methods 2).

### Substrate scope

**Figure 1:**
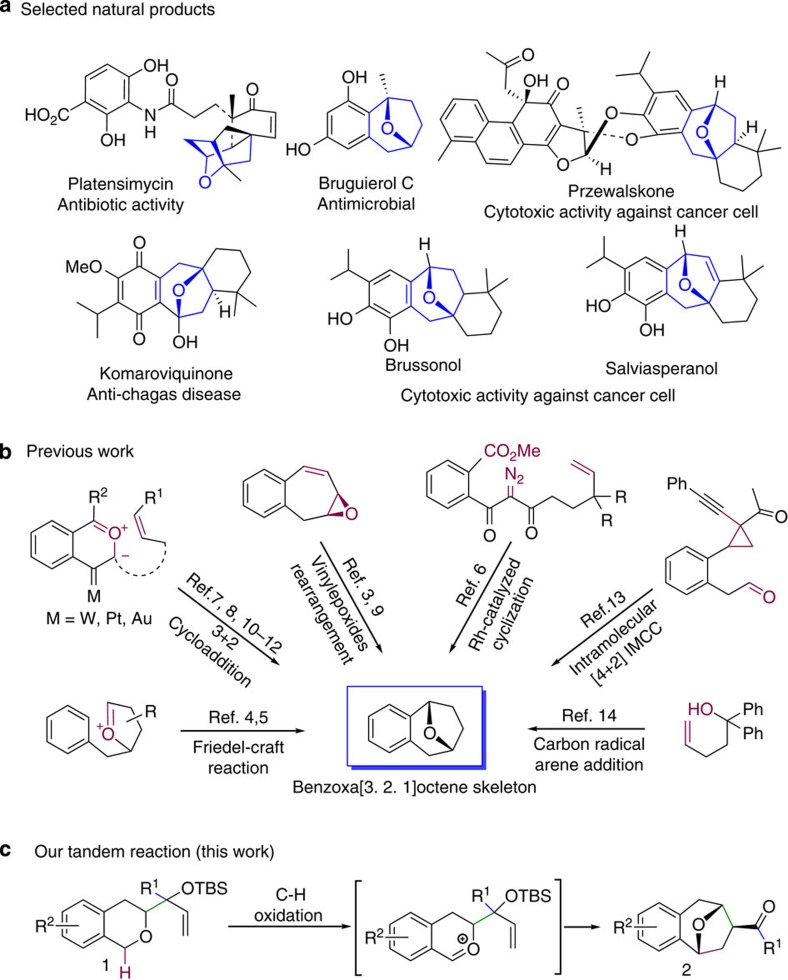
Representative natural products containing benzoxa[3.2.1]octane skeleton and approaches to it. (**a**) Selected bioactive natural products having benzoxa[3.2.1]octane skeleton. (**b**) Previous methods for construction of benzoxa[3.2.1]octane skeleton. (**c**) Our synthetic proposal via a tandem C–H oxidation/cyclization/rearrangement reaction.

**Figure 2:**
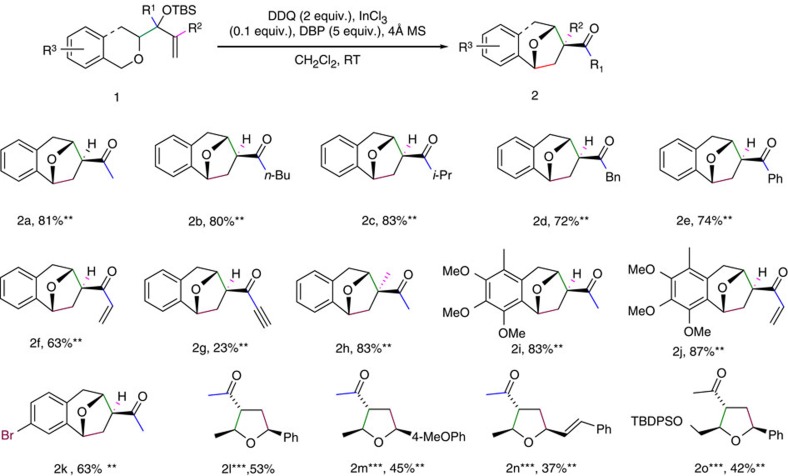
Reaction scope*. *All reactions unless notified were performed in experimental section procedure on a 0.1–1.5 mmol substrate scale in CH_2_Cl_2_ (0.1 mmol ml^−1^), 0.1 equiv. InCl_3_, 4 Å MS (50 mg per 0.1 mmol), 2.0 equiv. DDQ, 5.0 equiv. DBP at RT. Relative configuration of the products were assigned based on X-ray structure of **2h** and **2j** (CCDC 1000811, CCDC 1000827, See Supplementary Data 2 and 3 for more details). **Isolated yields. ***1.1 equiv. DDQ was used.

Using these optimal reaction conditions, we tested the substrate scope of this transformation extensively (Fig. 2), starting with the allylic substituents at R^1^. Substrates **1b** and **1c** with *n*-butyl and *i*-propyl groups at this position reacted well and gave the desired products **2b** and **2c** in respective yields of 80 and 83%. Benzyl-substituted **1d** also afforded the desired product **2d** in good 72% yield. In contrast, substrates with more electron-rich groups at R^1^ gave only medium to good yields. For example, substrates **1e** and **1f** with phenyl and vinyl substituents gave the corresponding products **2e** and **2f** in respective yields of 74 and 63% yield. The substrate **1g** with an acetylenyl substituent generated the product **2g** in a lower yield of 23%. Using substrate **1h** with a methyl substitution at the ally C=C led to smooth production of **2h** with a quaternary stereocenter in 83% yield. The product's relative configuration was confirmed by X-ray diffraction analysis. Next, the substituent effects on the aromatic ring of the isochroman moiety were investigated—compounds **1i** and **1j** fully substituted with MeO or Me reacted well, giving products **2i** and **2j** in respective yields of 83 and 87%. These relatively high yields may mean that the electron-donating methoxyl group stabilizes the benzylic oxonium carbocation in the transition state in Fig. 1c better than hydrogen does. Compound **1k** with a bromo substituent at the 7th position in the isochroman moiety led to the desired product **2k** in 63% yield, extending the flexibility of our approach for further derivatization.

We also expanded the substrate scope to acyclic systems to allow the synthesis of multi-substituted THF derivatives, which exist in numerous bioactive and pharmaceutical molecules^35^,^36^,^37^. Four representative allylic siylethers **1l**–**1o** with terminal benzylic or allylic ethers as the reaction trigger generated the corresponding products **2l**–**2o** in 37–53% yields under the same optimal conditions. Notably, the reaction was triggered efficiently by either benzylic ethers (**1l**, **1m**, **1o**) or cinnamylic ethers. (**1n**) When a TBDPS ethylene ether was created in **1o** to compete with benzyl ether during the expected reaction, only the benzyl ether underwent reaction, affording the product **2o** in 42% yield. Under the same optimal conditions, yields were generally lower with acyclic substrates than with cyclic ones, which may be due to hyperoxidation of THF products that produced furan-type by-products (Supplementary Methods 2.2).

### Asymmetric syntheses of (−)-brussonol and (−)-przewalskine *E*

**Figure 3:**
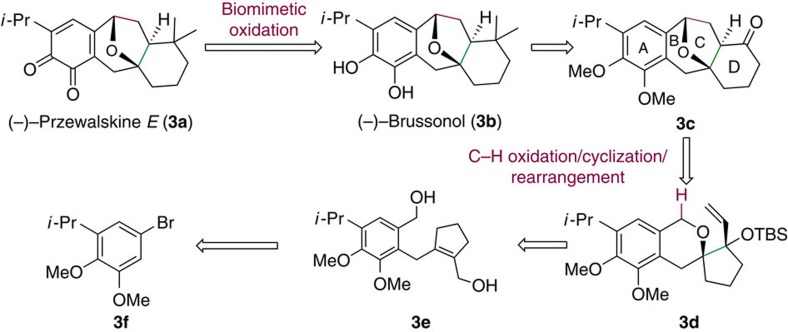
Retrosynthetic analysis of **3a,b**. The current method was used to construct the core framework.

To further demonstrate the utility of this novel method for synthesizing polycyclic molecules, we applied it to the total synthesis of two important bioactive natural products—tetracyclic (−)-przewalskine *E* (**3a)** and (−)-brussonol (**3b**)^2^,^38^,^39^,^40^. Two diastereoselective routes to **3b** have been reported by Sarpong^41^ and Jennings^42^, while its asymmetric synthesis has been achieved by Majetich^43^. We are unaware of reports of the synthesis of **3a**. In our retrosynthetic analysis (Fig. 3), we hypothesized that we could generate **3a** from **3b** via biomimetic oxidation, and that **3b** could be obtained from ketone intermediate **3c**. We planned to construct rings C and D in **3c** by applying our novel method to the tricyclic allylic siylether **3d,** which could be obtained from achiral allylic alcohol **3e** via a challenging tandem Sharpless asymmetric epoxidation/epoxy opening. The precursor **3e** could be prepared from the simple starting material **3f**^44^ in a few short steps.

**Figure 4:**
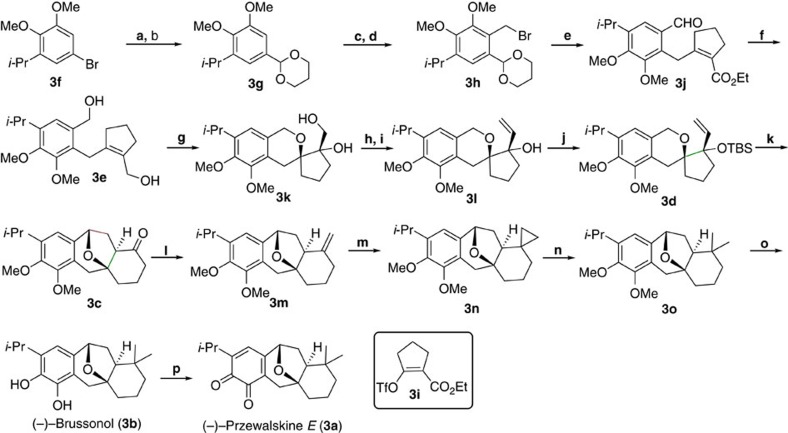
Asymmetric total synthesis of (−)-przewalskine *E* (3a) and (−)-brussonol (3b). Reagent and conditions: (**a**) *n*-BuLi, Et_2_O, −78 °C, then DMF; (**b**) 1,3-propanediol, CH(OEt)_3_, (*n*-Bu)_4_N^+^Br_3_^-^, 65 °C; 77% (2 steps); (**c**) *n*-BuLi, Hexane/Et_2_O, RT, then CH_2_O in THF, −78 °C; (**d**) Ph_3_P, CBr_4_, CH_2_Cl_2_, 0 °C, 41% (2 steps); (**e**) (i) Zn, THF, 0 °C to RT, then Pd(Ph_3_P)_2_Cl_2_, **3i** in DMF, 90 °C; (ii) 4 mol l^−1^ HCl, THF/H_2_O (4:1), 69% (2 steps); (**f**) DIBAL-H, CH_2_Cl_2_, −78 °C to RT, 93%; (**g**) (−)-DET, Ti(*i-*PrO)_4_, *t*-BuO_2_H, −25 °C to −50 °C, CH_2_Cl_2_, 90% yield, 83% ee; (**h**) DMSO, DIPEA, SO_3_·Py, CH_2_Cl_2_;(**i**) Ph_3_PCH_3_Br, *t*-BuOK, Tol, 72% (2 steps); (**j**) TBSOTf, Et_3_N, CH_2_Cl_2_, 0 °C to 40 °C, 98%; (**k**) 4 Å MS, 2,6-DBP, InCl_3_, DDQ, CH_2_Cl_2_, RT, 82%; (**l**) Ph_3_PCH_3_Br, *t*-BuOK, Toluene, 88%; (**m**) Et_2_Zn, CH_2_I_2_, Tol., 58%; (**n**) Pt_2_O, H_2_ (1 atm), AcOH, 65 °C, 89%; (**o**) EtSH, NaH, DMF, 150 °C, 76%; (**p**) Ag_2_O, Et_2_O, RT, 71%. DIPEA, *N*,*N*-diisopropylethylamine.

On the basis of this strategy, we started our synthesis by preparing allylic alcohol **3e** from bromide **3f** (Fig. 4), which was formalized and protected as a 1,3-dioxane **3g** to survive subsequent metallization^45^,^46^. Deprotonation of **3g** with *n*-BuLi followed by quenching with formaldehyde and finally bromination of the resulting hydroxyl group with Ph_3_P/CBr_4_ gave benzyl bromide **3h** in 41% yield over two steps. Coupling bromide **3h** with vinyl triflate **3i**^47^ and then removing the 1,3-dioxane protecting group in aqueous HCl afforded aldehyde **3j** in 69% yield over two steps. Excess diisobutylaluminum hydride (DIBAL-H) was used to reduce **3j** in a single step to diol **3e** in 93% yield. Then we investigated the key tandem Sharpless asymmetric epoxidation/epoxy opening of **3e**. The expected tricyclic species **3k** was obtained in 90% yield and 83% ee in the presence of classic Sharpless catalyst (1.5 equiv.) at −50 °C. While this enantioselectivity is not ideal, it appears to be a rare example of successful *tetra*-substituted olefin epoxidation^48^. Selective oxidation of the primary hydroxyl of **3k** followed by methylenenation afforded the desired tertiary allylic alcohol **3l**, which was protected to give the precursor **3d**. Fortunately, our optimal conditions of oxidative cyclization/ring enlargement gave the expected tetracyclic skeleton **3c** in high yield (81%) and excellent stereoselectivity, no other isomer was detected.

At this stage, only the installation of a gem-dimethyl group remained to complete the synthesis of **3b**. Initial attemps to transform the carbonyl in ketone **3c** directly into gem-dimethyl using TiMe_2_Cl_2_ reagent^49^ gave the desired product **3o** in low yield. Therefore, we adopted a three-step protocol involving Wittig reaction, cyclopropanation and reduction. Treating **3c** with Ph_3_PCH_3_Br/*t*-BuOK followed by Simmons–Smith cyclopropanation of the resulting exo-cyclic olefin gave the cyclopropane compound **3n**, which was hydrogenated with H_2_ (1 atm)/PtO_2_ to afford the desired **3o** in 45% yield over three steps^50^. Demethylating **3o** using with EtSNa/DMF provided natural product **3b** in 76% yield, and the spectral data were identical to those reported by Sarpong^41^ (Supplementary Tables 1 and 2). We screened various oxidants for the biomimetic oxidation of **3b** to **3a**; Ag_2_O proved to be the best, affording the natural product **3a** in 71% yield. Its spectral data were identical to those reported by Zhao^40^ (Supplementary Tables 3 and 4).

## Discussion

The tandem C–H oxidation/cyclization/rearrangement of isochroman-derived allylic silylether described here shows good chemo- and stereoselectivity as well as good product yield. We demonstrated the usefulness of this approach by using it to achieve the asymmetric total syntheses of (−)-brussonol and (−)-przewalskine *E*. We expect that this approach will find additional applications in organic synthesis.

**Figure 5:**
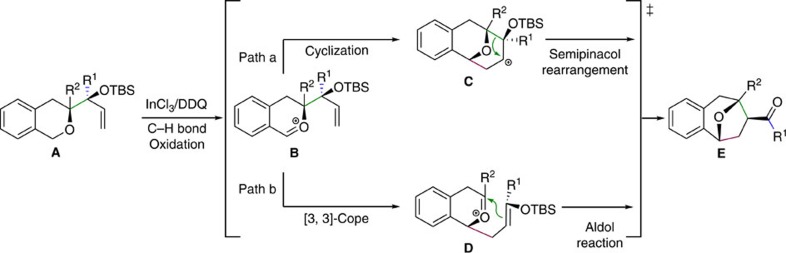
Proposed mechanism. (**a**) Tandem C–H bond oxidation/cyclization/semipinacol rearrangement reaction; (**b**) tandem C–H bond oxidation/[3,3]-Cope rearrangement/aldol reaction.

This approach involves simpler initiation and substrate preparation as well as milder reaction conditions than a similar acetal rearrangement under acidic conditions via alkoxy release reported by Overman's group^51^,^52^,^53^,^54^,^55^,^56^,^57^,^58^. Notably, we nearly always obtained the desired product with a benzoxa[3.2.1]octane framework as a single diastereomer (Fig. 2). In term of the mechanism of this transformation, there are two possible pathways whereby intermediate **B** may react with a benzylic oxocarbenium cation under oxidative conditions (Fig. 5). One is a tandem cyclization/semipinacol rearrangement to give **E** (pathway a) and the other is a tandem [3,3]-Cope rearrangement/aldol reaction (pathway b)^52^. Our results suggest that pathway b is more likely, since we did not obtain products with a benzoxa[3.3.1] skeleton when we used substrates **1e**, **1f**, **1g** or **1j**, all of which should preferentially undergo migration of an electron-rich R^1^ group when a semipinacol rearrangement process is involved. In addition, both substrates **1h** and **1h′**, which are epimers at the allylic position, gave a single diastereomeric product. Whether pathway b is the true one under our optimal conditions needs to be confirmed.

## Methods

### Materials

For NMR analysis, see Supplementary Figs 1–136 and 139–154. For high-performance liquid chromatography traces, see Supplementary Figs 137 and 138. For X-ray structures of the compounds, see Supplementary Figs 155–158.

### General

All reactions under standard conditions were monitored by thin-layer chromatography. Column chromatography was performed on silica gel (200–300 mesh). Reaction solvents were distilled before use, and all air- or moisture-sensitive reactions were conducted under an argon atmosphere. Melting points were measured using a micro-melting point apparatus. Optical rotations were measured using a 0.1-ml cell with a 1-cm path length. ^1^H NMR and ^13^C NMR spectra were recorded in CDCl_3_ on a 400-MHz instrument (^1^H NMR) and a 100-MHz instrument (^13^C NMR); spectral data were reported in p.p.m. relative to trimethylsilane as the internal standard. Infrared spectra were recorded on a Fourier transform infrared spectrometer. High-resolution mass spectral analysis data were measured using the electrospray ionization technique on a Fourier transform ion cyclotron resonance mass analyser.

### General procedure for this tandem reaction

To a solution of **1a** (249 mg, 0.78 mmol) in CH_2_Cl_2_ (8.0 ml), we successively added a 4-Å molecular sieve (400 mg), 2,6-DBP (924 mg, 3.90 mmol, 5.0 equiv.) and InCl_3_ (18 mg, 0.08 mmol, 0.1 equiv.) at room temperature under an argon atmosphere. The mixture was stirred for 15 min, then DDQ (361 mg, 98%, 1.56 mmol, 2.0 equiv.) was added. The resulting brown mixture was stirred for 12 h at room temperature before it was filtered via an SiO_2_ column with petroleum ether/EtOAc (4:1) as eluent to remove the molecular sieve and 2,6-DBP. The filtrate was concentrated under vacuum and chromatographed on an SiO_2_ column (petroleum ether/EtOAc, 10:1) to give product **2a** as a colourless oil (128 mg, 0.63 mmol, 81% yield).

## Additional information

**Accession codes:** The X-ray crystallographic coordinates for structures reported in this study have been deposited at the Cambridge Crystallographic Data Centre (CCDC), under deposition numbers 1000817, 1000816, 1000811 and 1000827. These data can be obtained free of charge from The Cambridge Crystallographic Data Centre via www.ccdc.cam.ac.uk/data_request/cif.

**How to cite this article:** Jiao, Z-W. *et al.* Tandem C–H oxidation/cyclization/rearrangement and its application to asymmetric syntheses of (−)-brussonol and (−)-przewalskine *E*. *Nat. Commun.* 6:7332 doi: 10.1038/ncomms8332 (2015).

## Supplementary Material

Supplementary Information Supplementary Figures 1-158, Supplementary Tables 1-4, Supplementary Methods and Supplementary References

Supplementary Data 1 Single crystal X-ray diffraction of 1e

Supplementary Data 1 Single crystal X-ray diffraction of 2h

Supplementary Data 1 Single crystal X-ray diffraction of 2j

Supplementary Data 1 Single crystal X-ray diffraction of S16'

## Figures and Tables

**Table 1:**
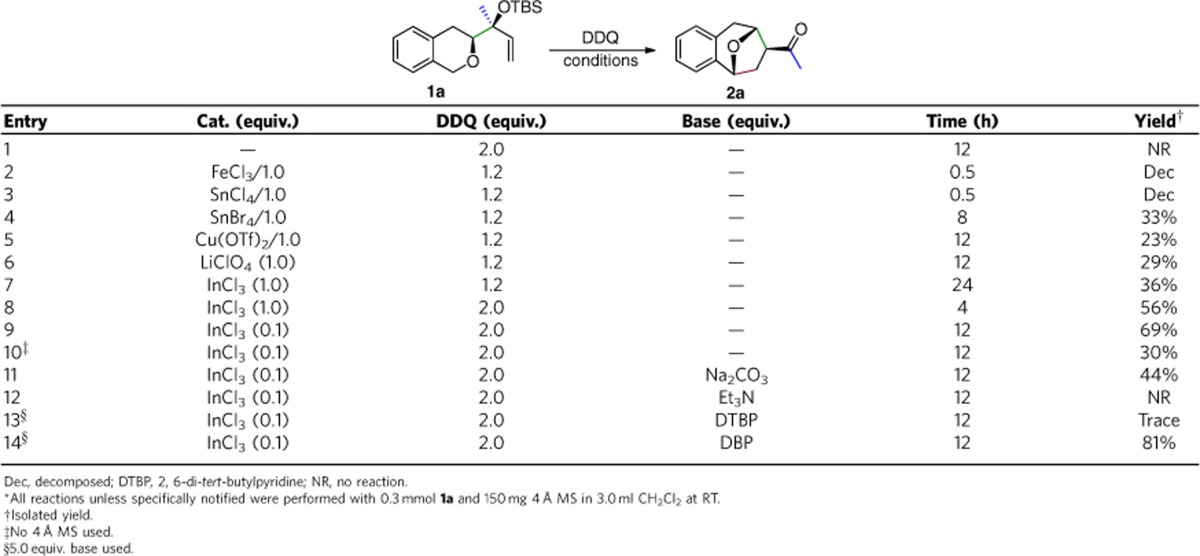
Conditions optimization*.
