# Supplementary material for: Tandem C–H oxidation/cyclization/rearrangement and its application to asymmetric syntheses of (−)-brussonol and (−)-przewalskine E
Source: Nat Commun. 2015 Jun 17;6:7332. doi: 10.1038/ncomms8332 (PMC4557391; doi:10.1038/ncomms8332)
Supplement: Supplementary Information — Supplementary Figures 1-158, Supplementary Tables 1-4, Supplementary Methods and Supplementary References [file ncomms8332-s1.pdf]

Supplementary Figure 1. ^{1}H NMR Spectrum of S2 (400 MHz, $CDCl_3$)

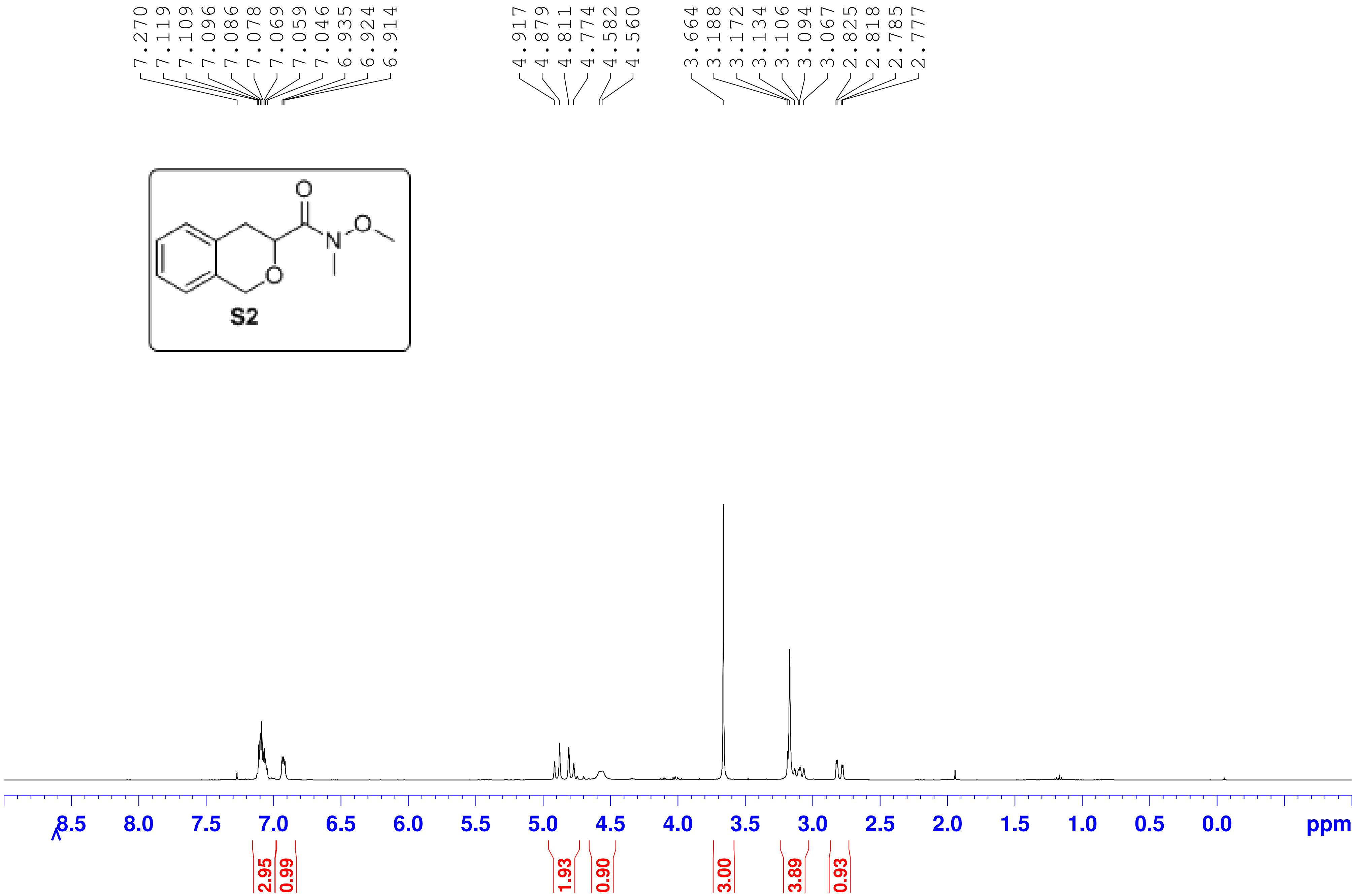

Supplementary Figure 2. ^{13}C NMR Spectrum of S2 (100 MHz, $CDCl_3$)

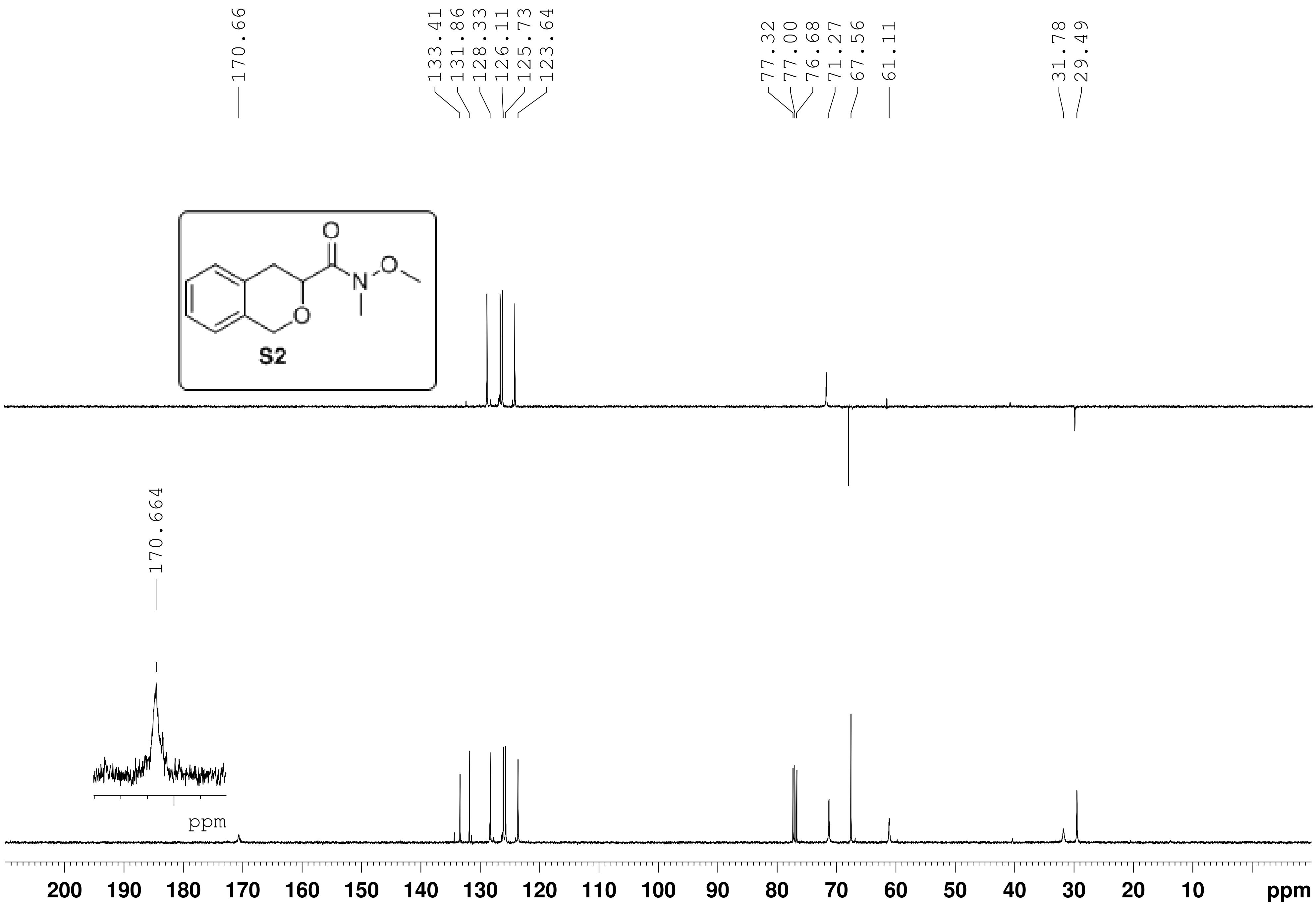

Supplementary Figure 3. ^{1}H NMR Spectrum of S3 (400 MHz, $CDCl_3$)

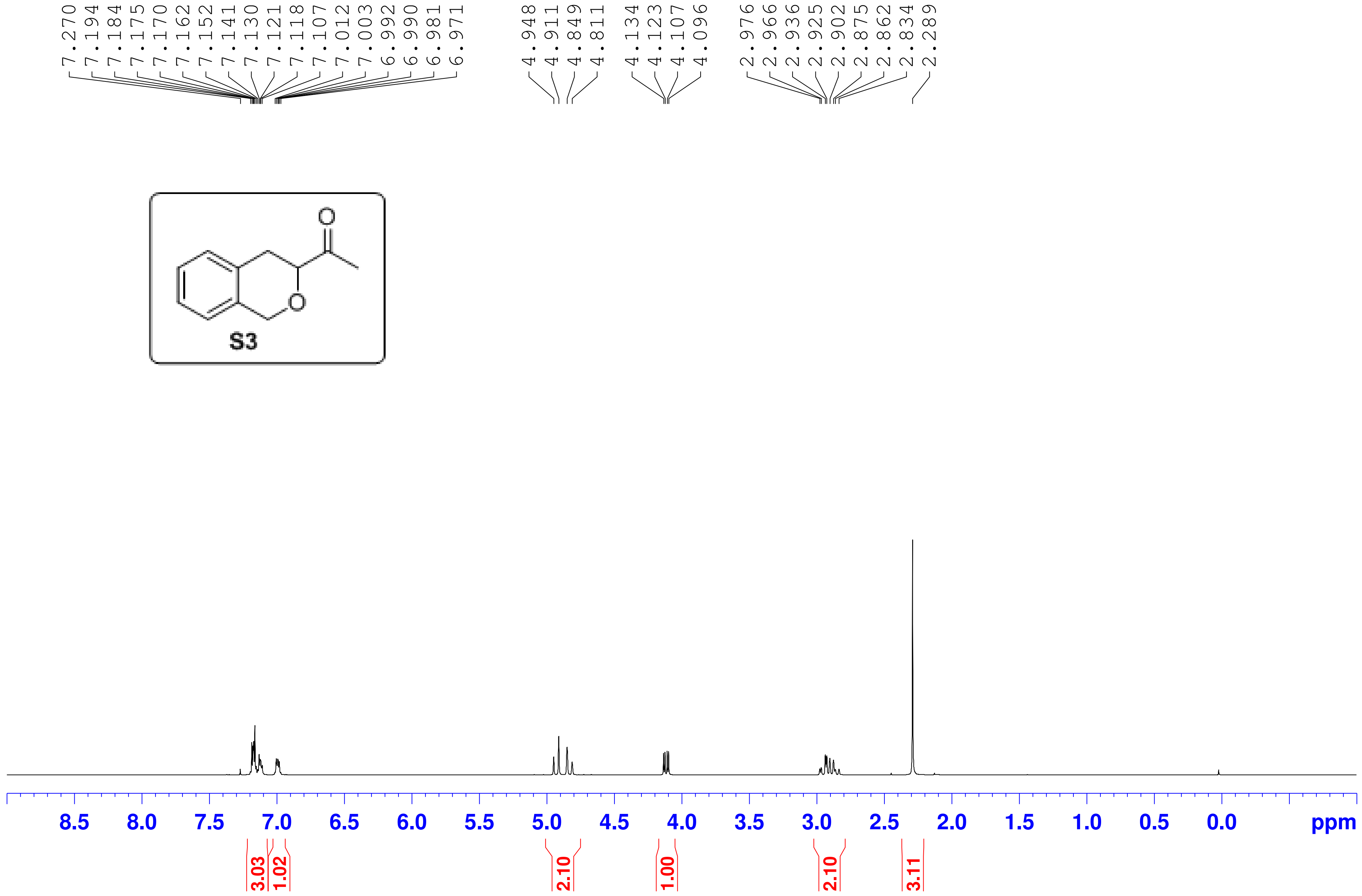

Supplementary Figure 4. ^{13}C NMR Spectrum of S3 (100 MHz, $CDCl_3$)

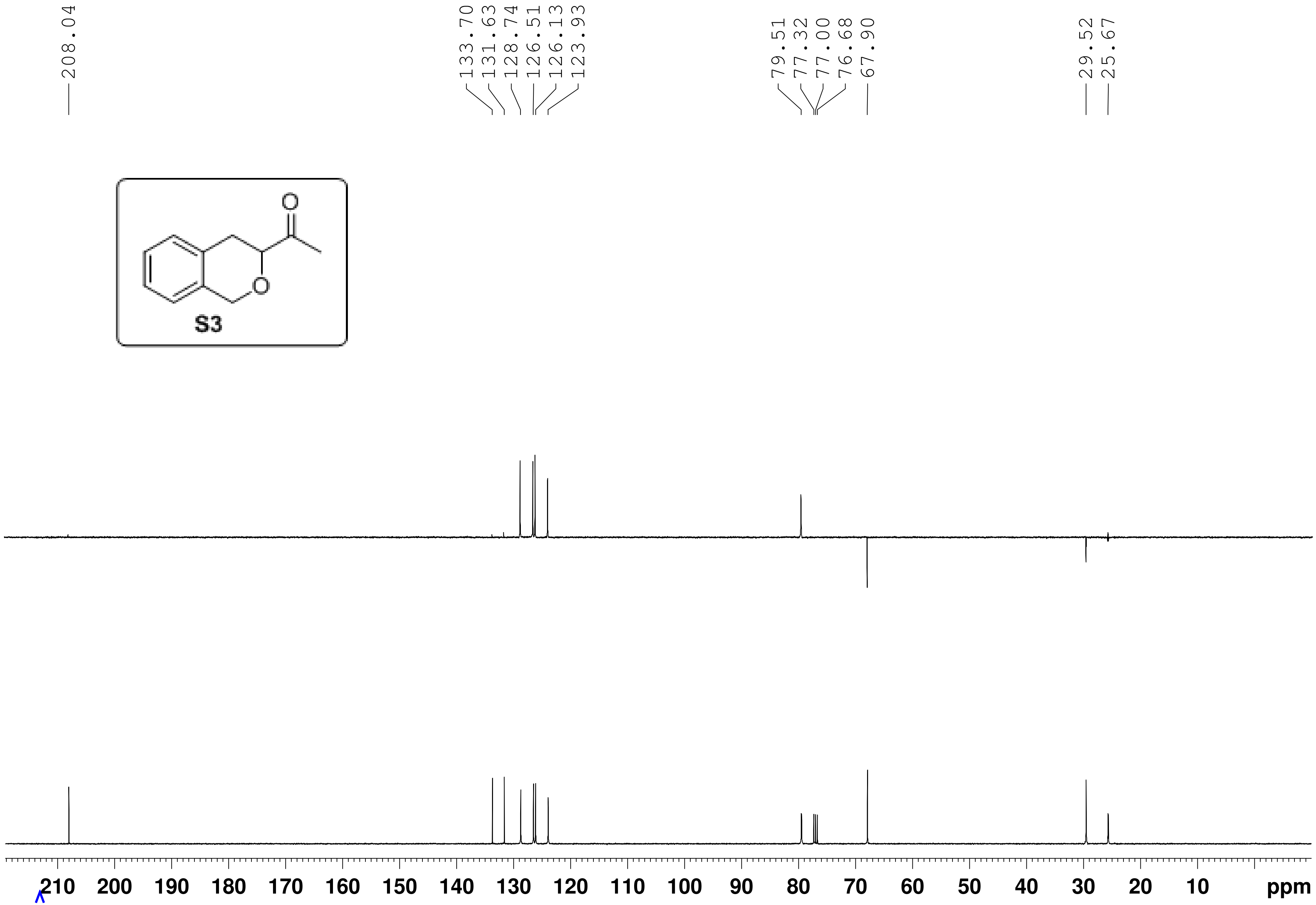

Supplementary Figure 5. ^{1}H NMR Spectrum of S4 (400 MHz, $CDCl_3$)

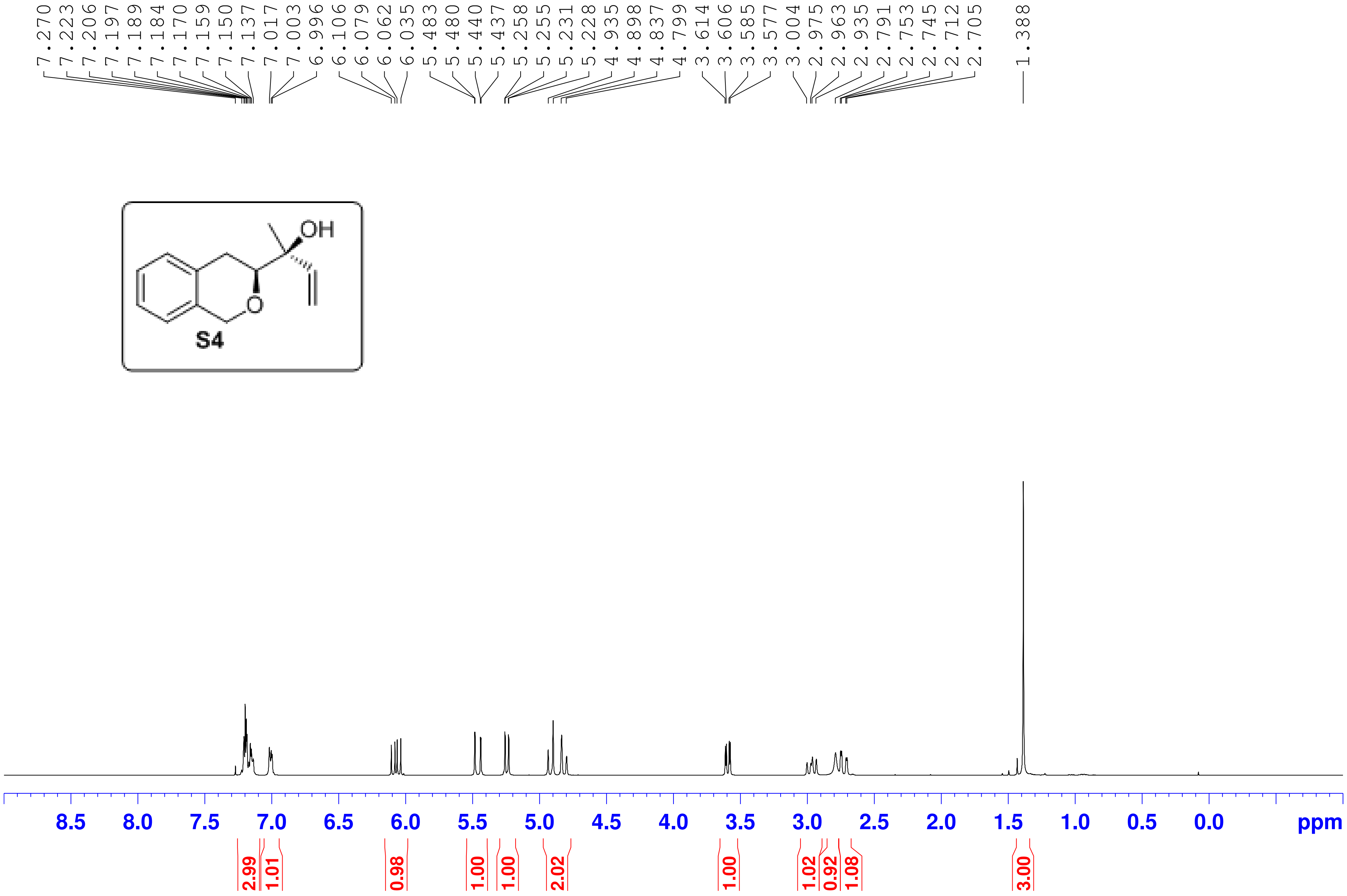

Supplementary Figure 6. ^{13}C NMR Spectrum of S4 (100 MHz, $CDCl_3$)

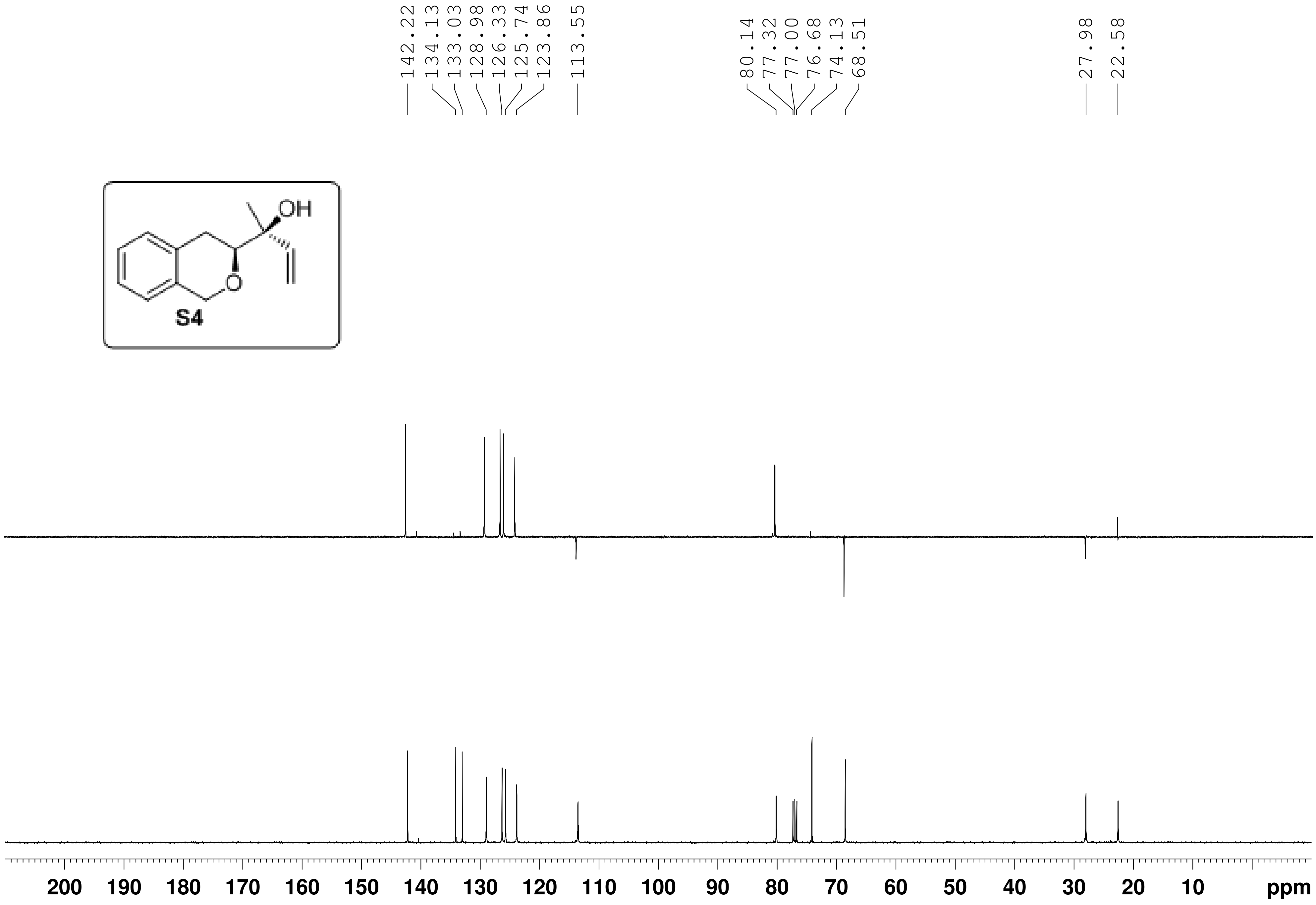

Supplementary Figure 7. ^1H NMR Spectrum of 1a (400 MHz, $CDCl_3$)

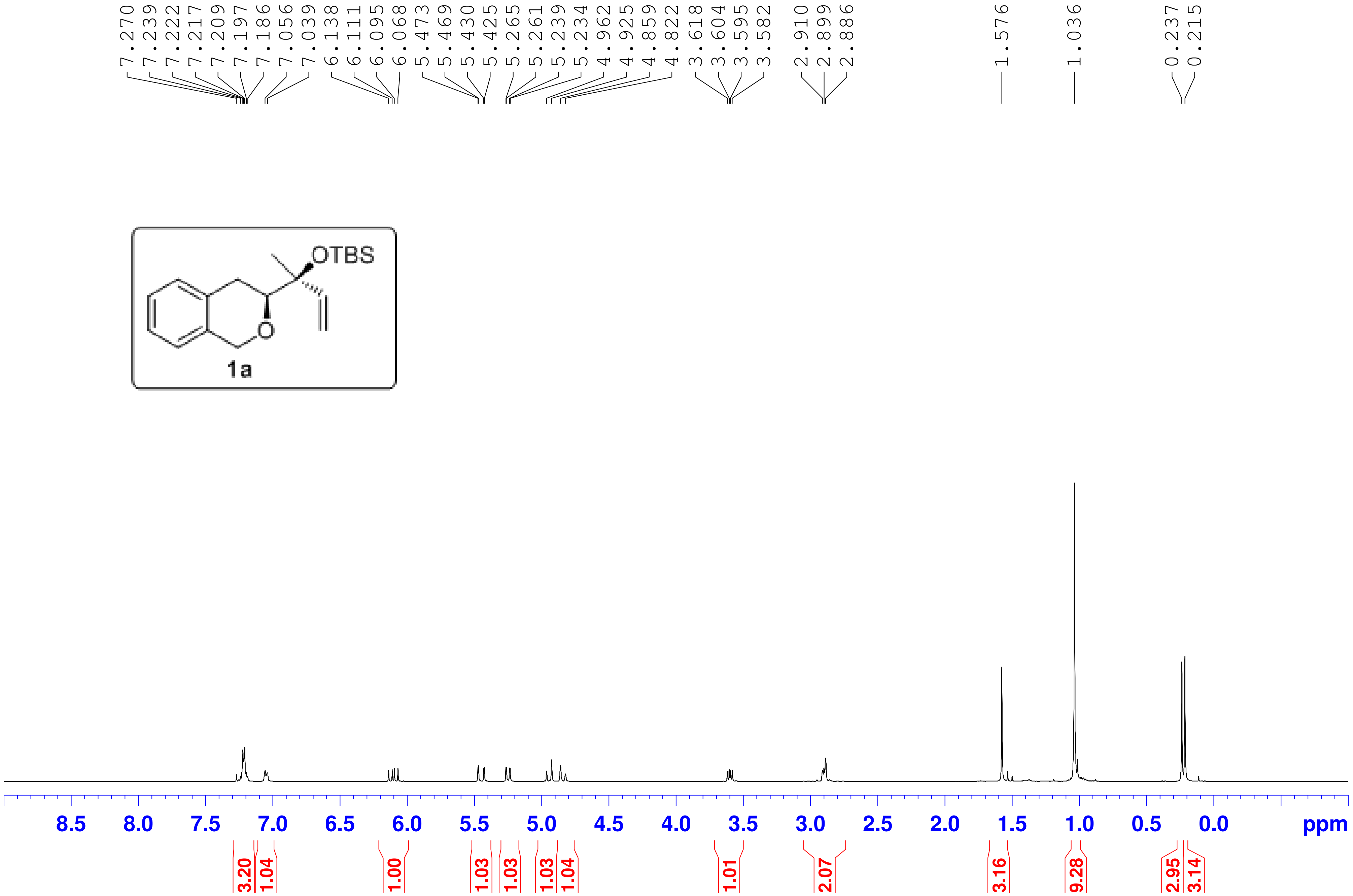

Supplementary Figure 8. ^{13}C NMR Spectrum of 1a (100 MHz, $CDCl_3$)

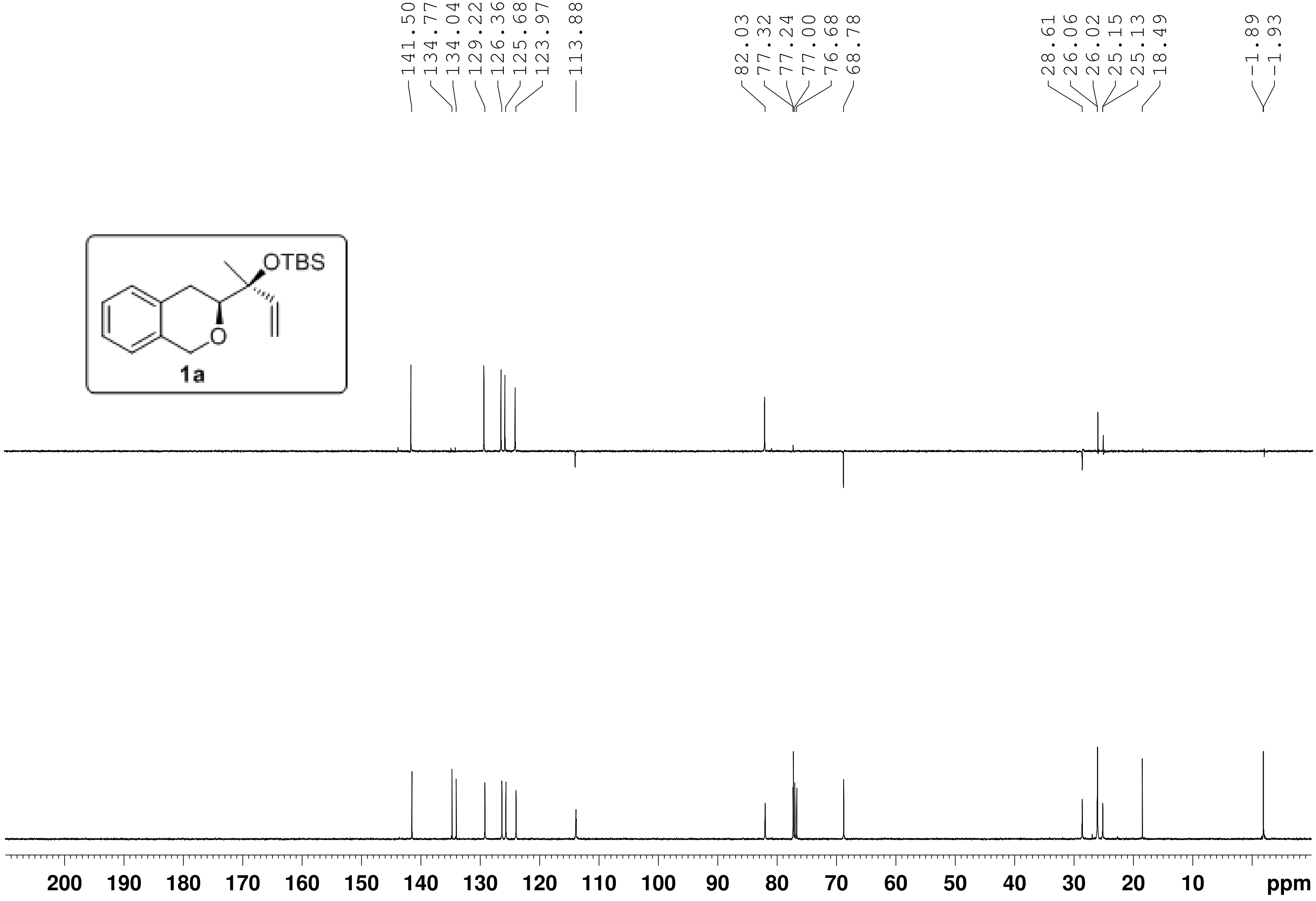

Supplementary Figure 9. ^1H NMR Spectrum of 2a (400 MHz, $CDCl_3$)

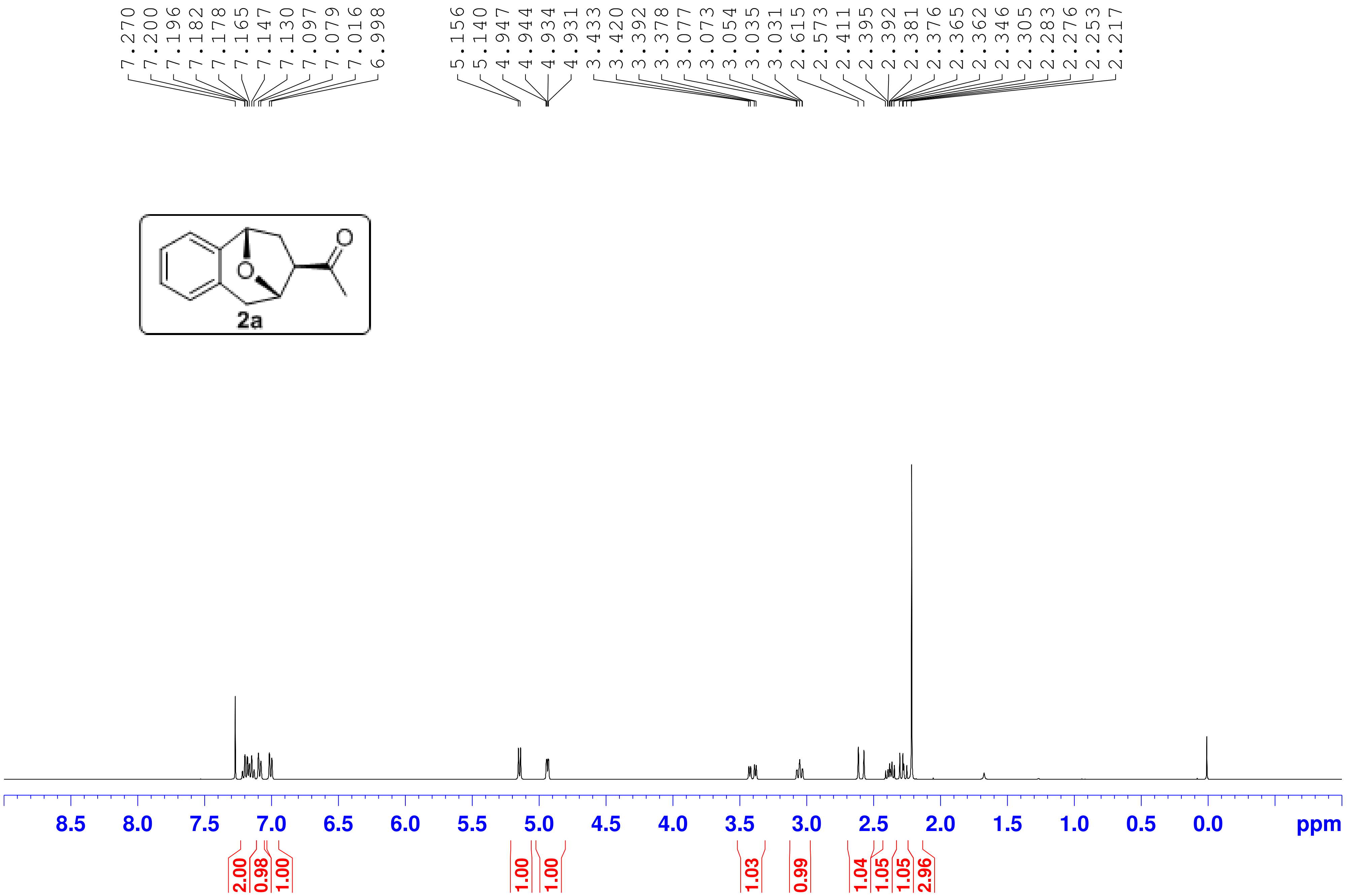

Supplementary Figure 10. ^{13}C NMR Spectrum of 2a (100 MHz, $CDCl_3$)

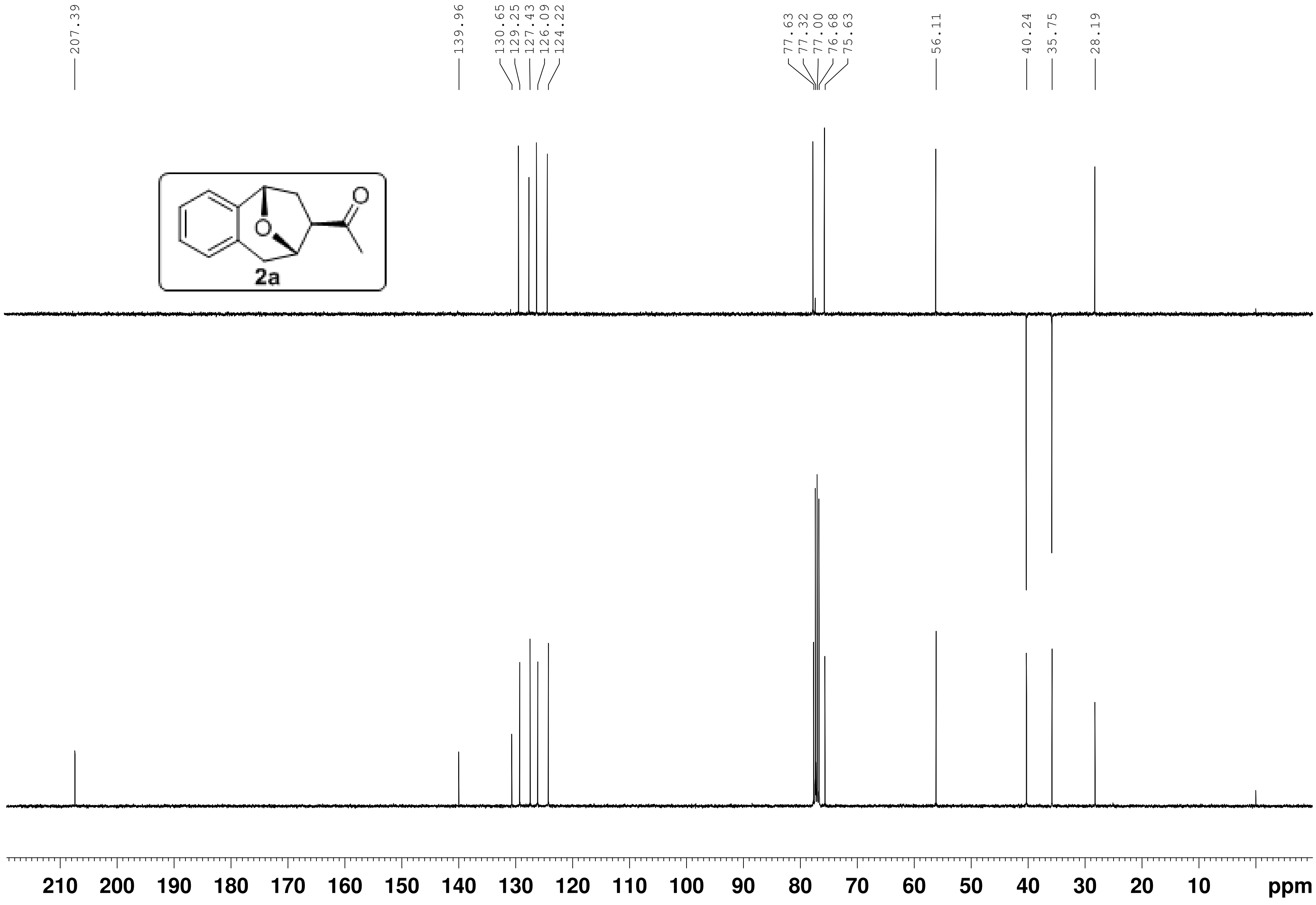

Supplementary Figure 11. ^{1}H NMR Spectrum of S5 (400 MHz, $CDCl_3$)

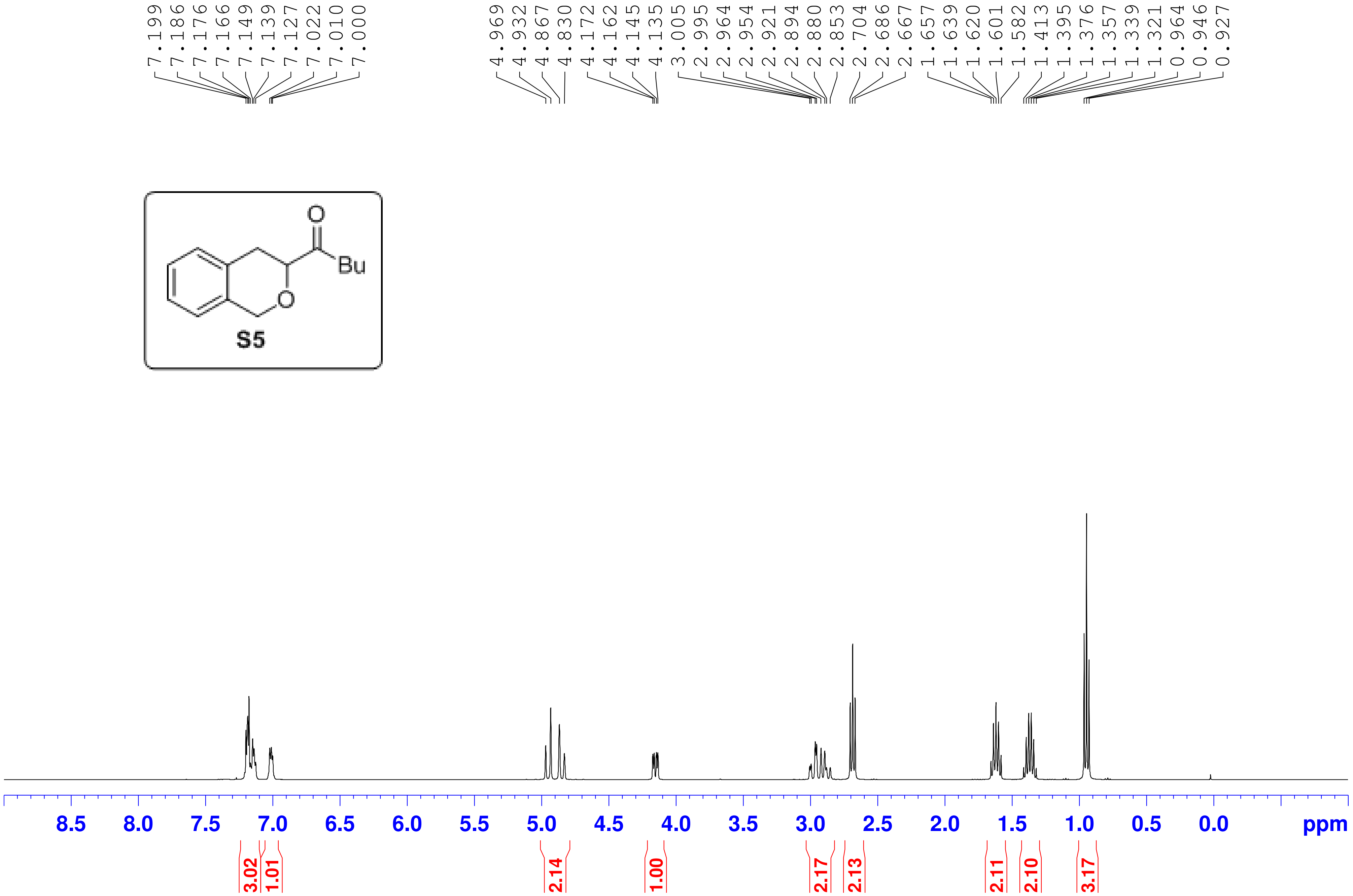

Supplementary Figure 12. ^{13}C NMR Spectrum of S5 (100 MHz, $CDCl_3$)

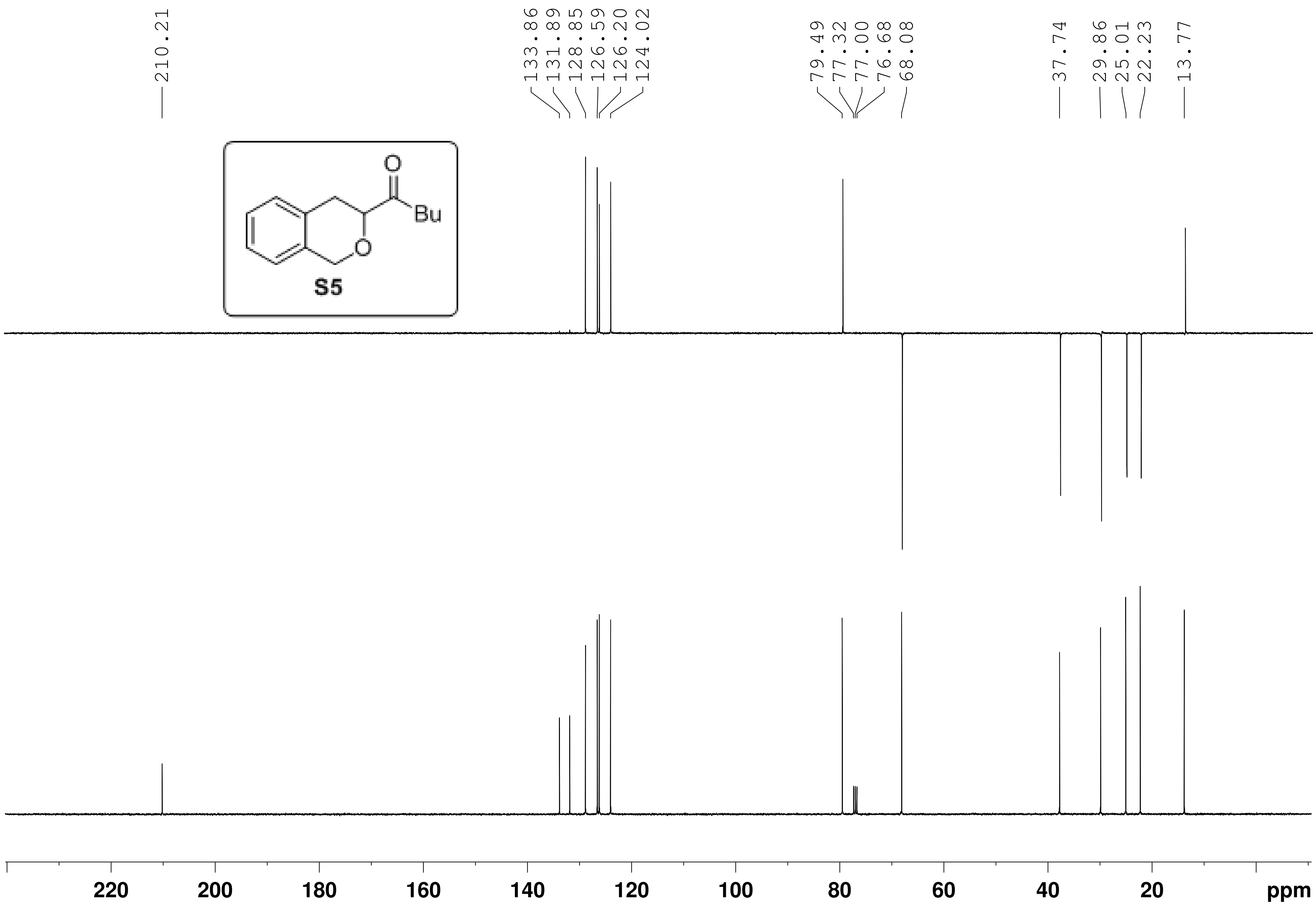

Supplementary Figure 13. ^{1}H **NMR Spectrum of S6 (400 MHz, $CDCl_3$)**

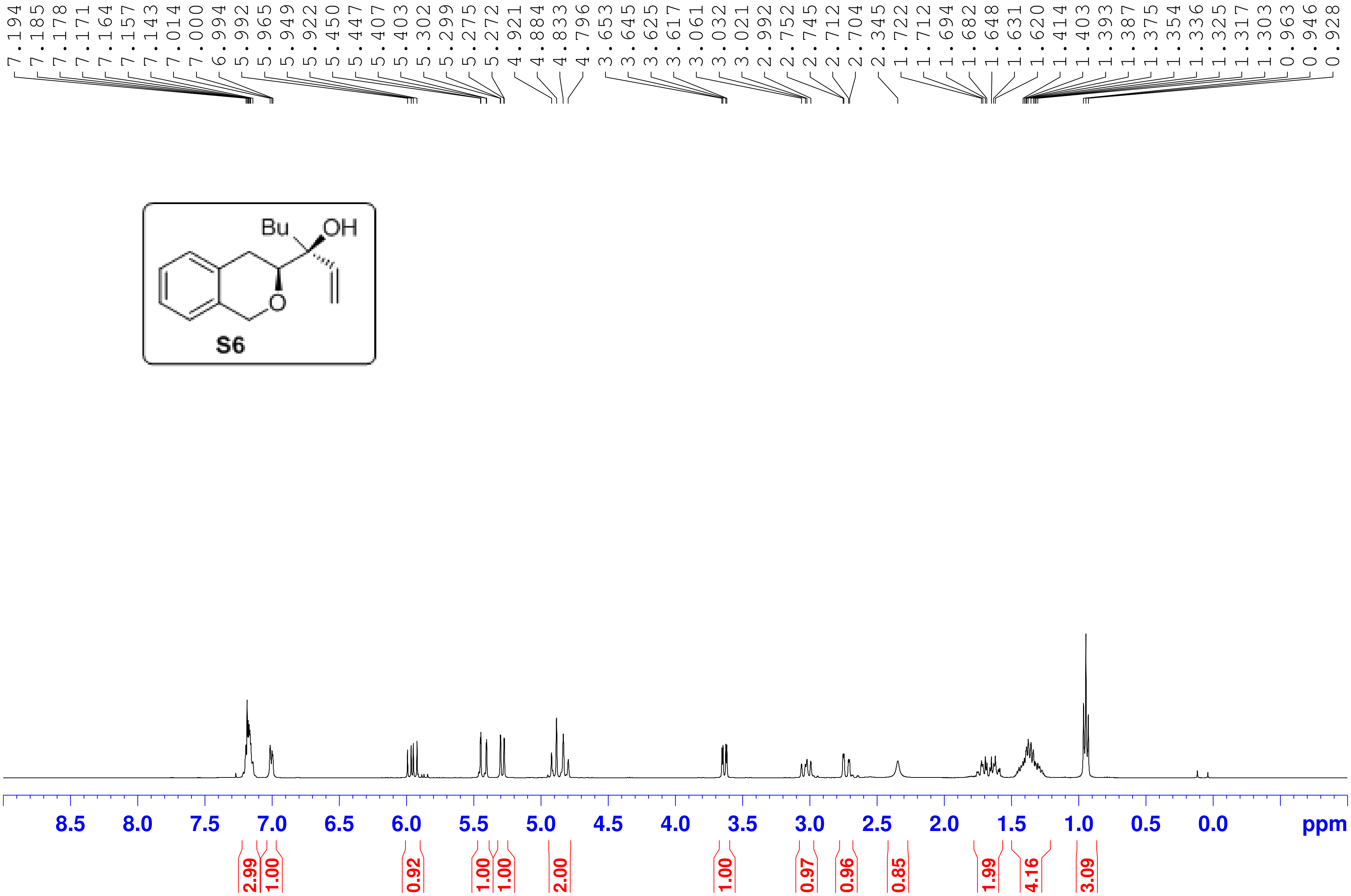

Supplementary Figure 14. ^{13}C NMR Spectrum of S6 (100 MHz, $CDCl_3$)

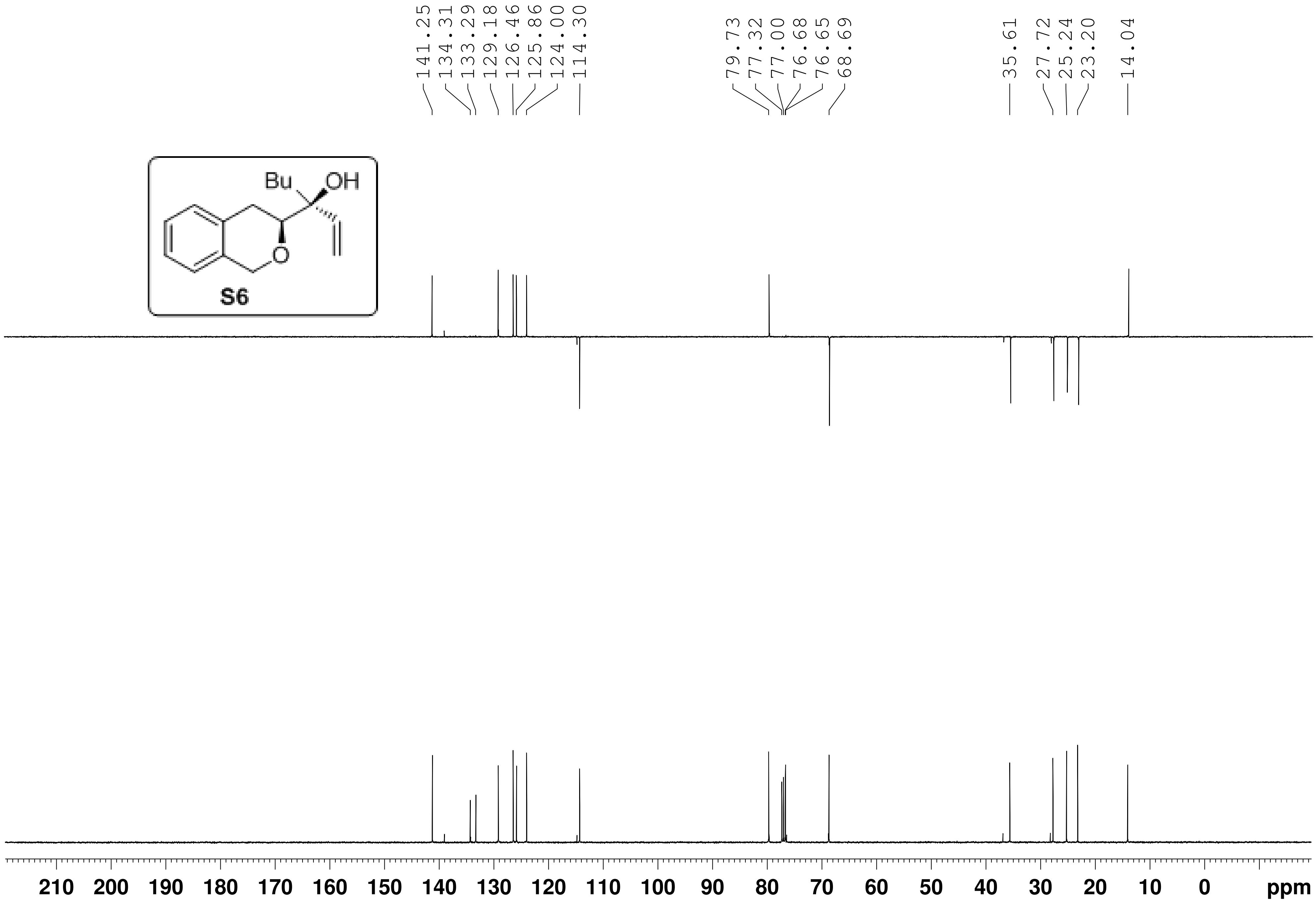

Supplementary Figure 15. ^{1}H NMR Spectrum of 1b (400 MHz, $CDCl_3$)

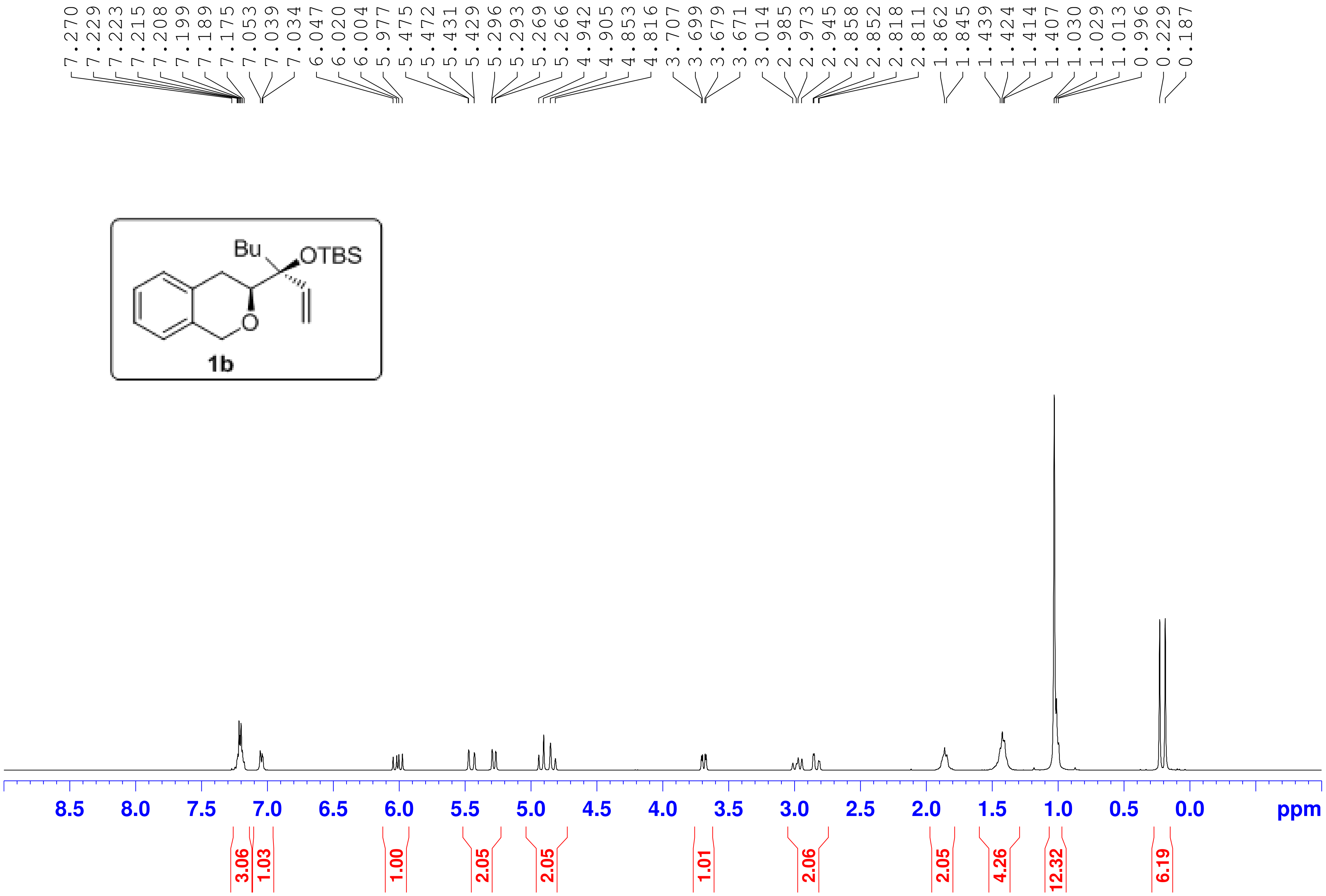

Supplementary Figure 16. ^{13}C NMR Spectrum of 1b (100 MHz, $CDCl_3$)

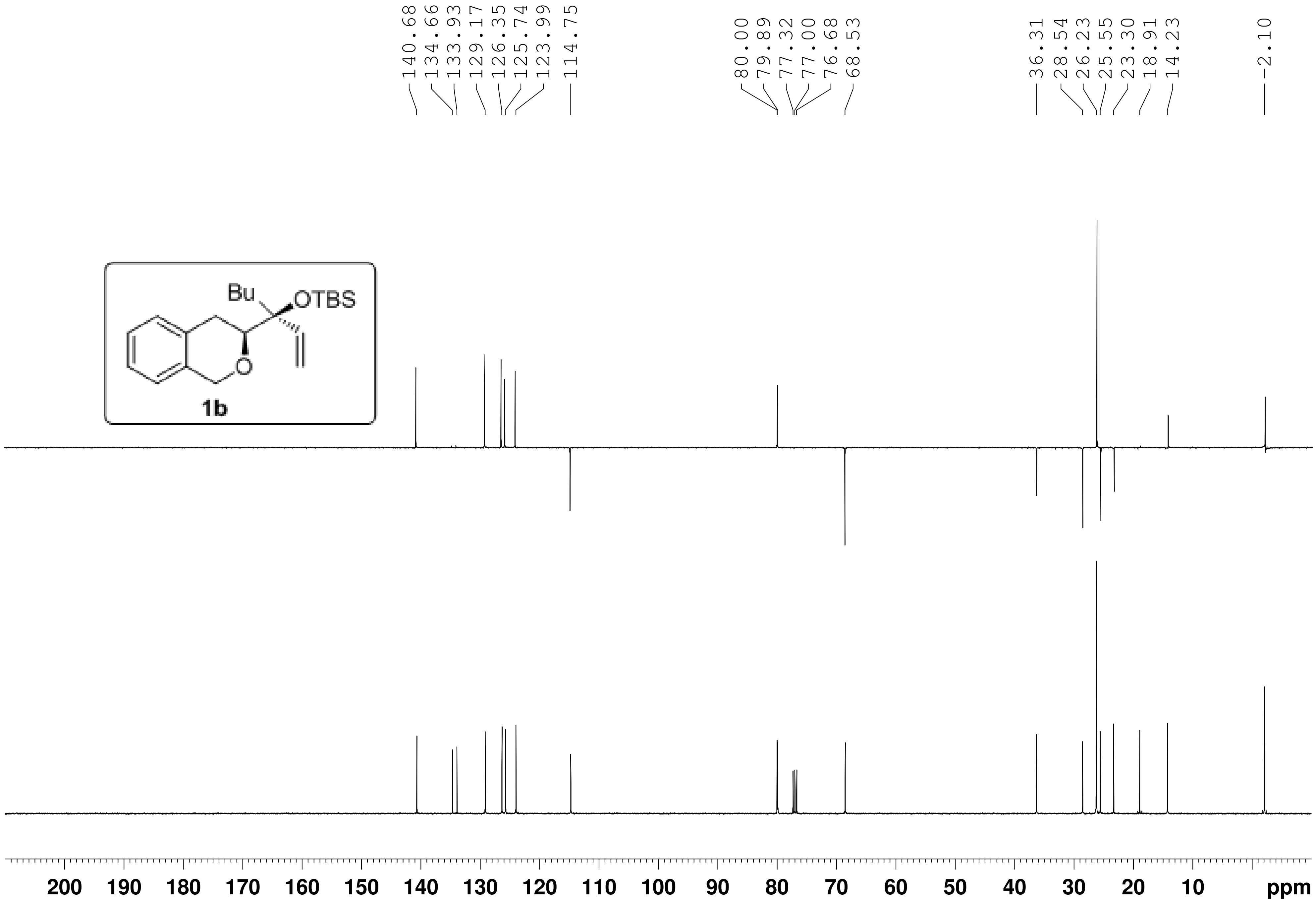

Supplementary Figure 17. ^{1}H **NMR Spectrum of 2b (400 MHz, $CDCl_3$)**

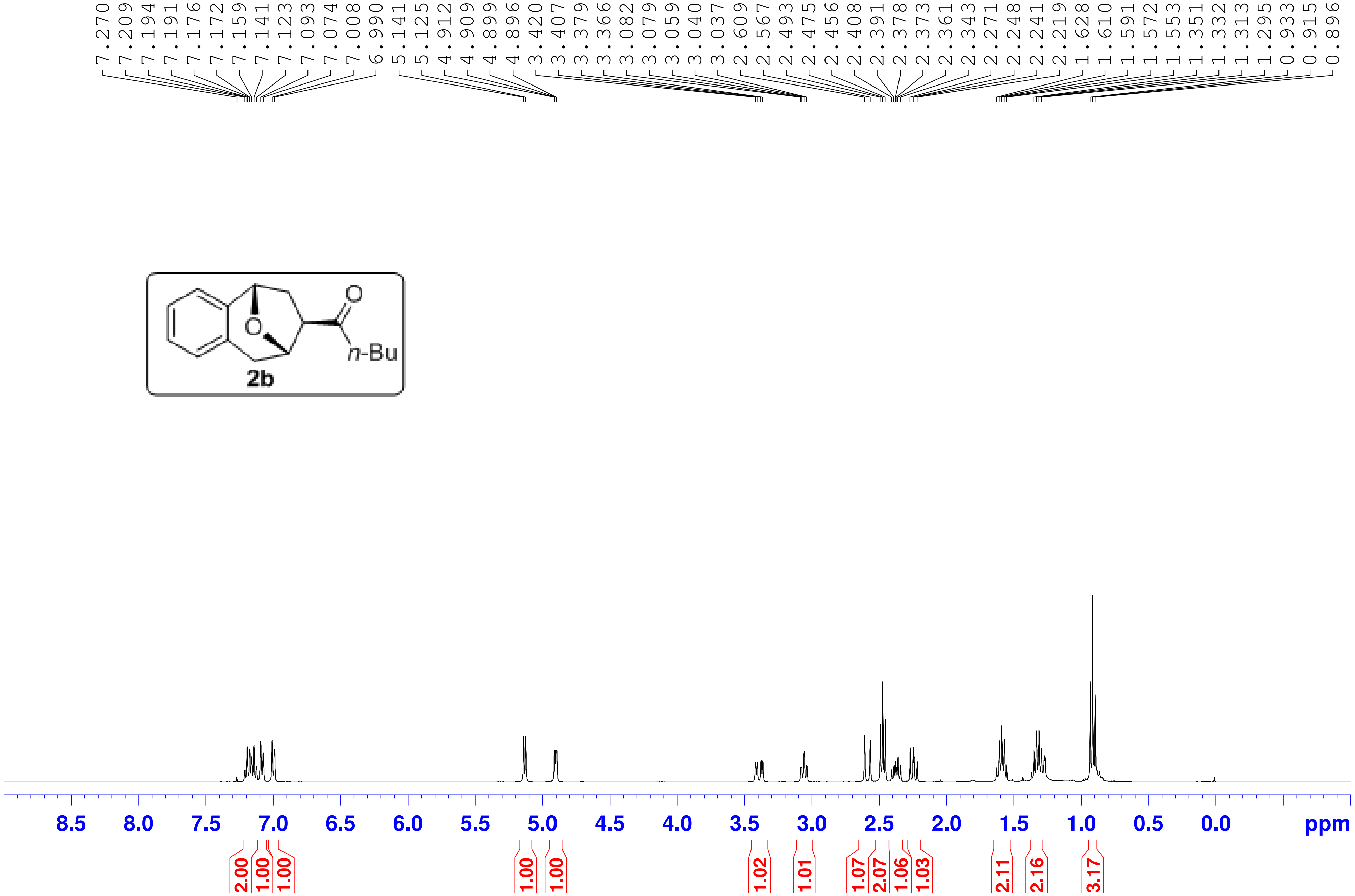

Supplementary Figure 18. ^{13}C NMR Spectrum of 2b (100 MHz, $CDCl_3$)

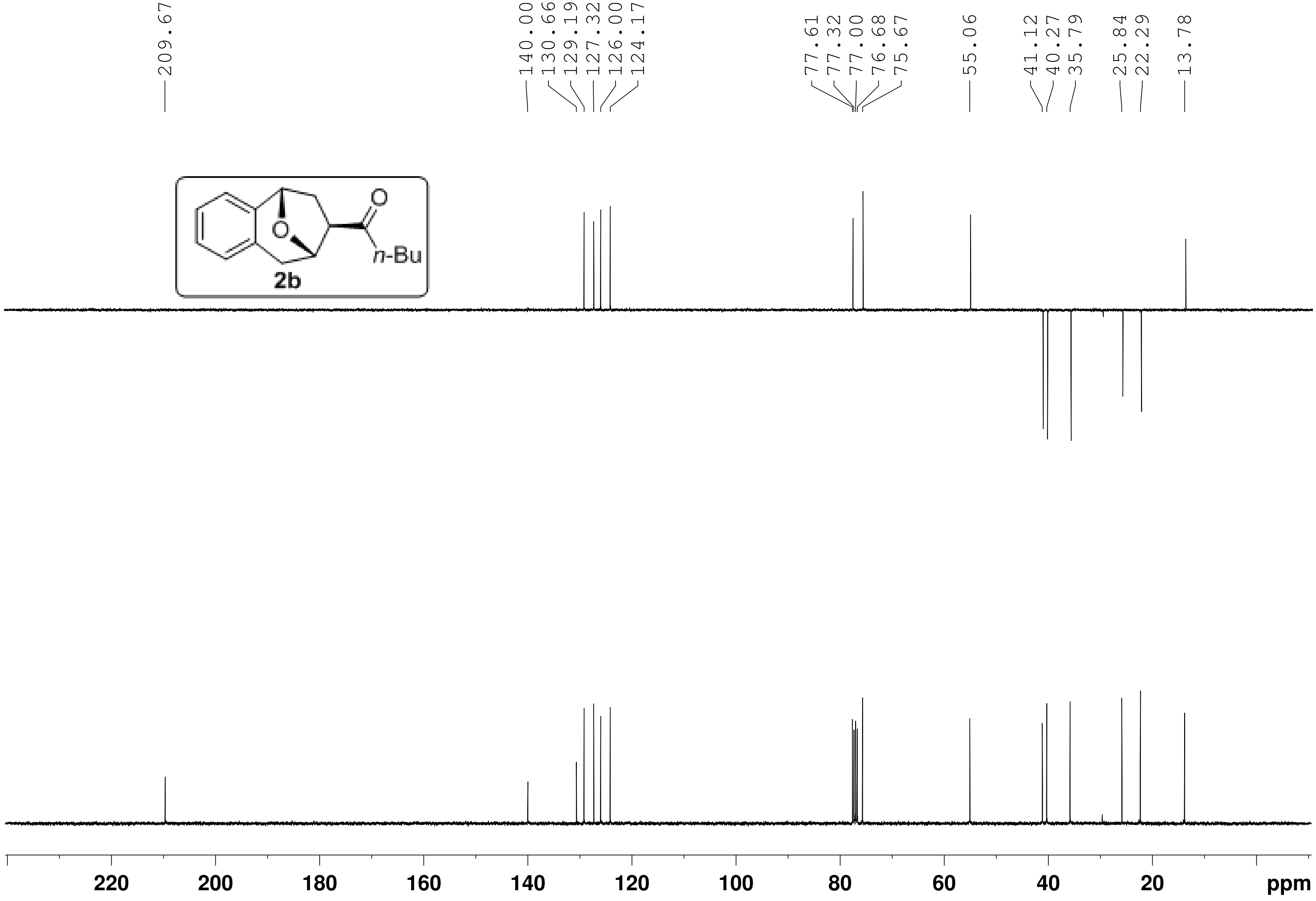

Supplementary Figure 19. ^{1}H NMR Spectrum of S7 (400 MHz, $CDCl_3$)

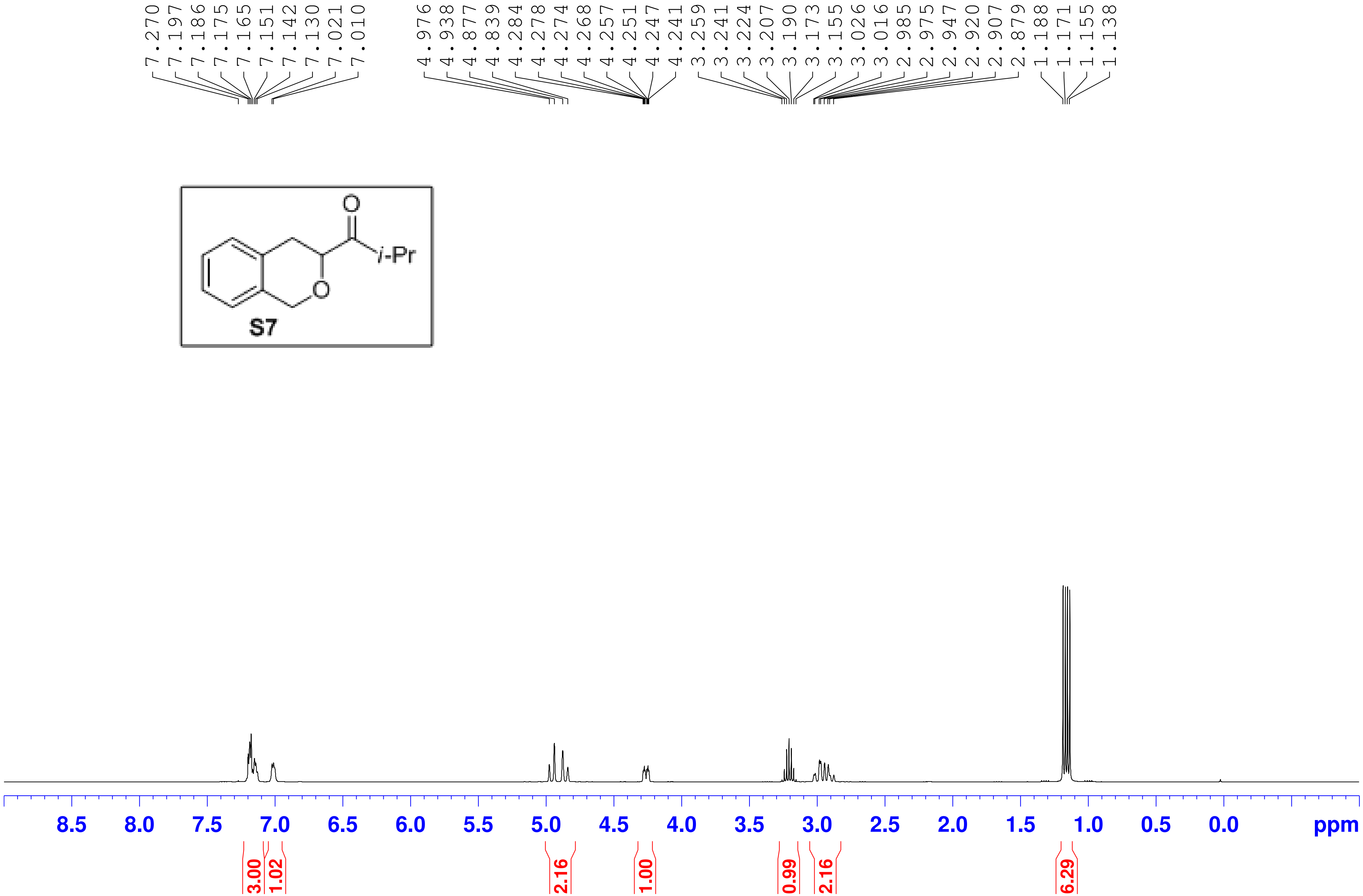

Supplementary Figure 20. ^{13}C NMR Spectrum of S7 (100 MHz, $CDCl_3$)

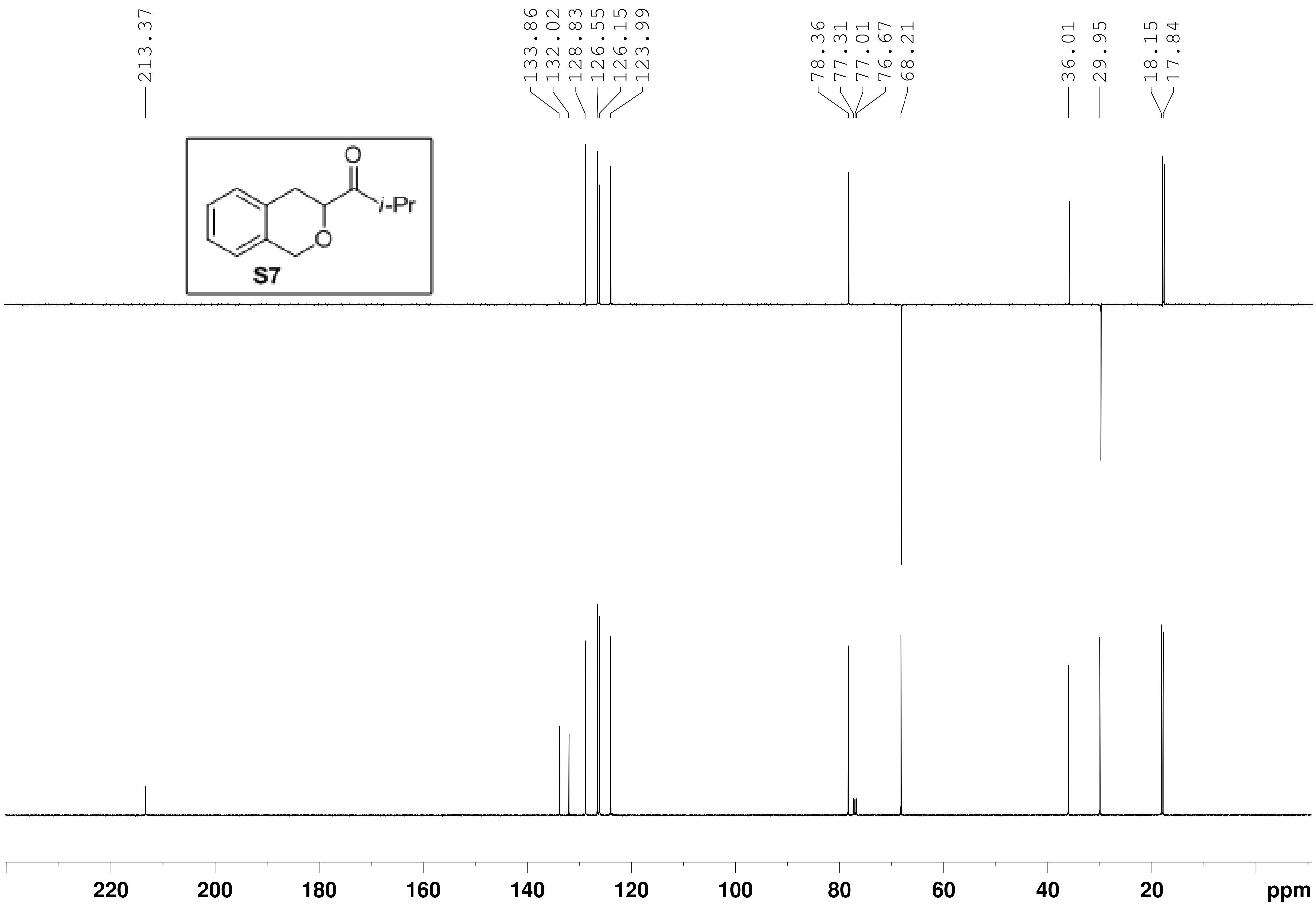

Supplementary Figure 21. ^{1}H NMR Spectrum of S8 (400 MHz, $CDCl_3$)

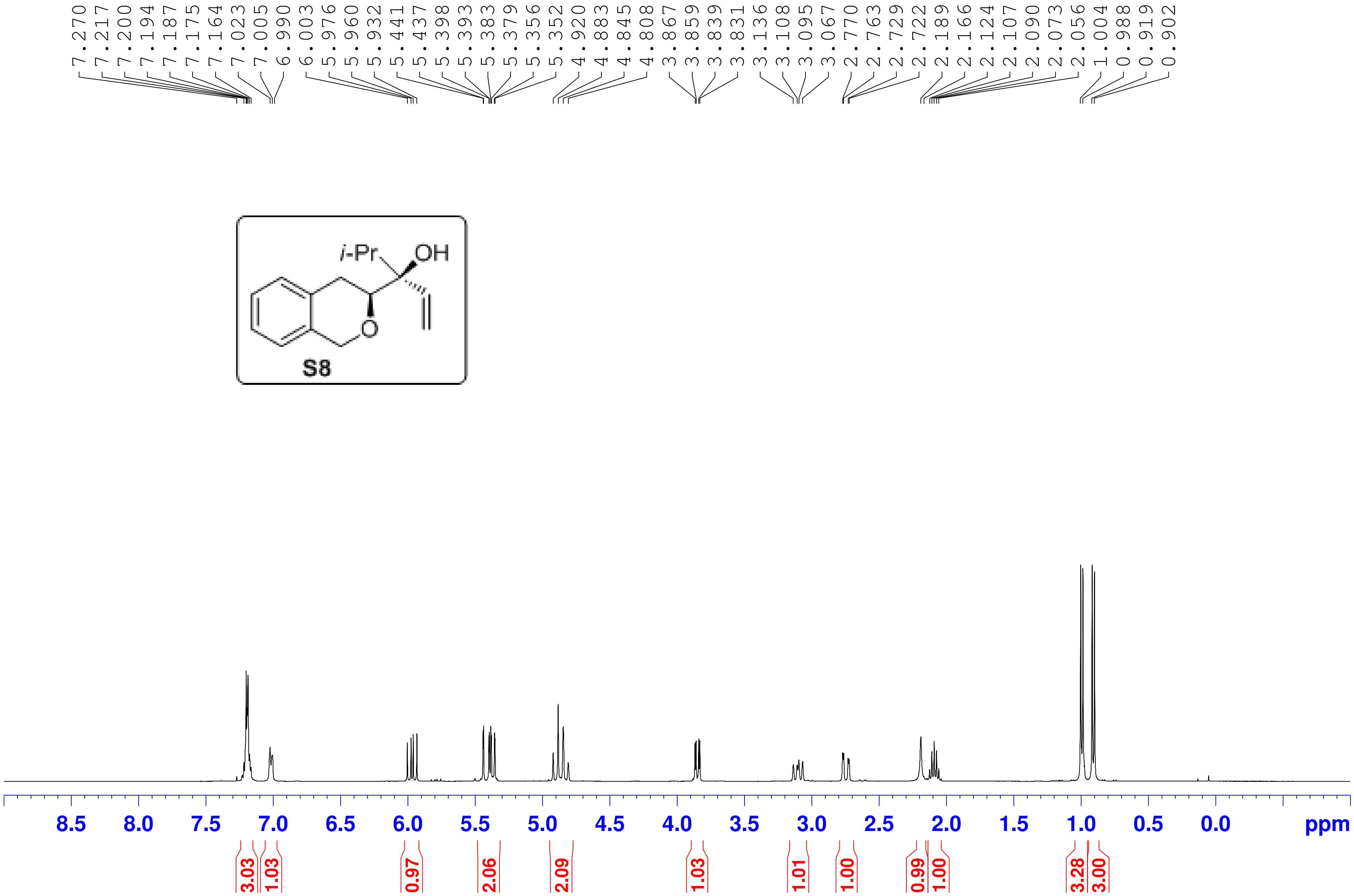

Supplementary Figure 22. ^{13}C NMR Spectrum of S8 (100 MHz, $CDCl_3$)

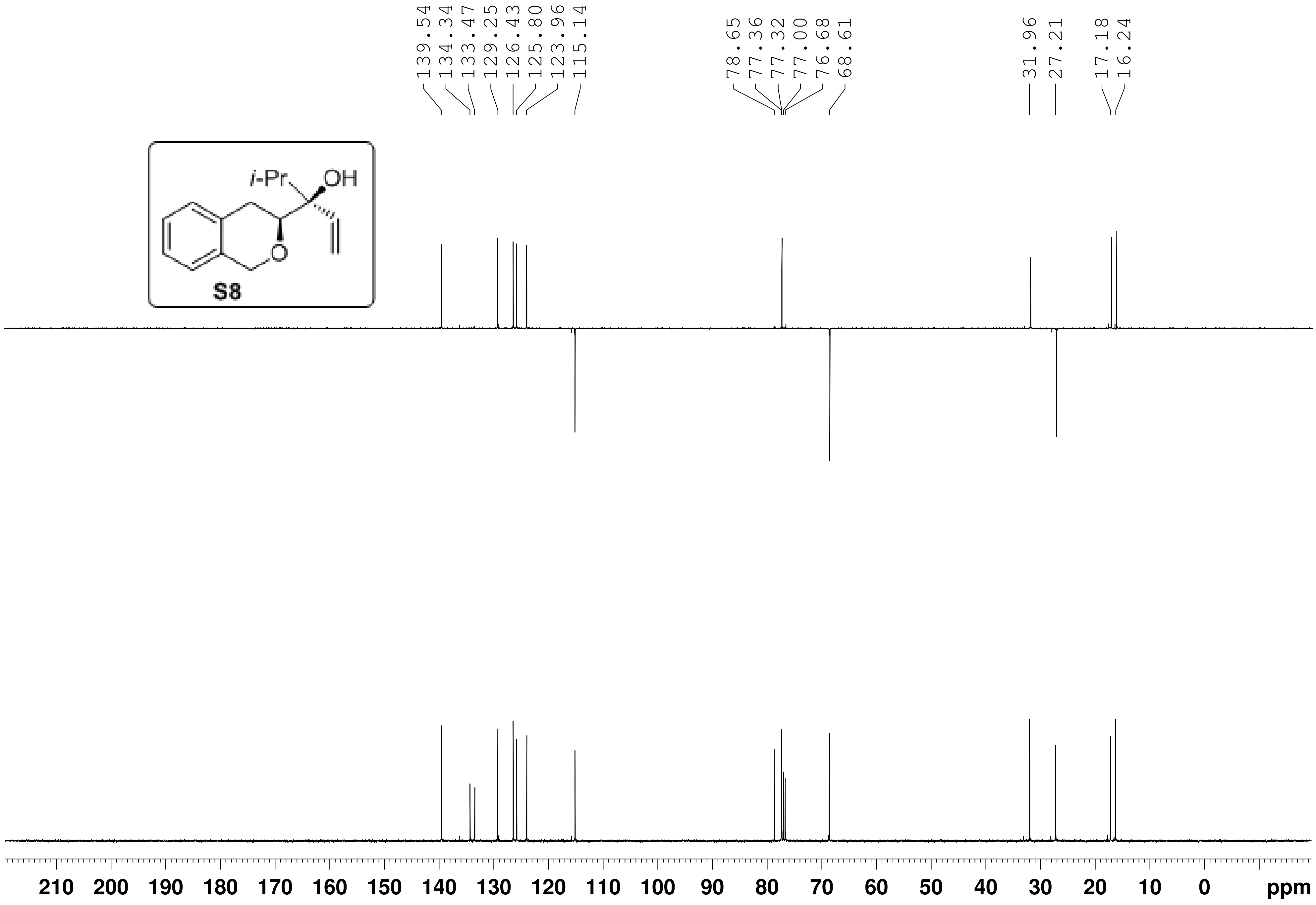

Supplementary Figure 23. ^{1}H NMR Spectrum of 1c (400 MHz, $CDCl_3$)

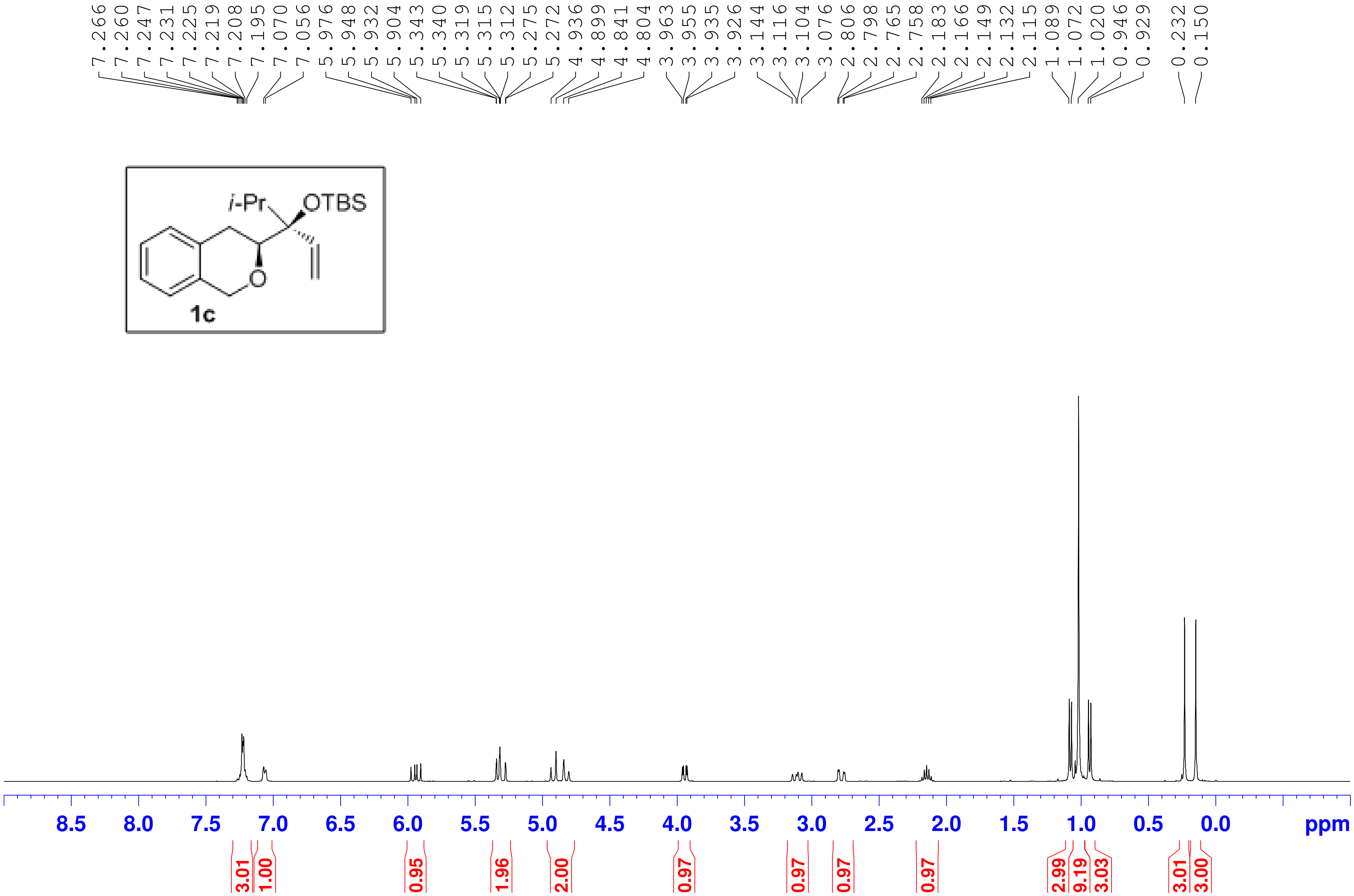

Supplementary Figure 24. ^{13}C NMR Spectrum of 1c (100 MHz, CDCl$_3$)

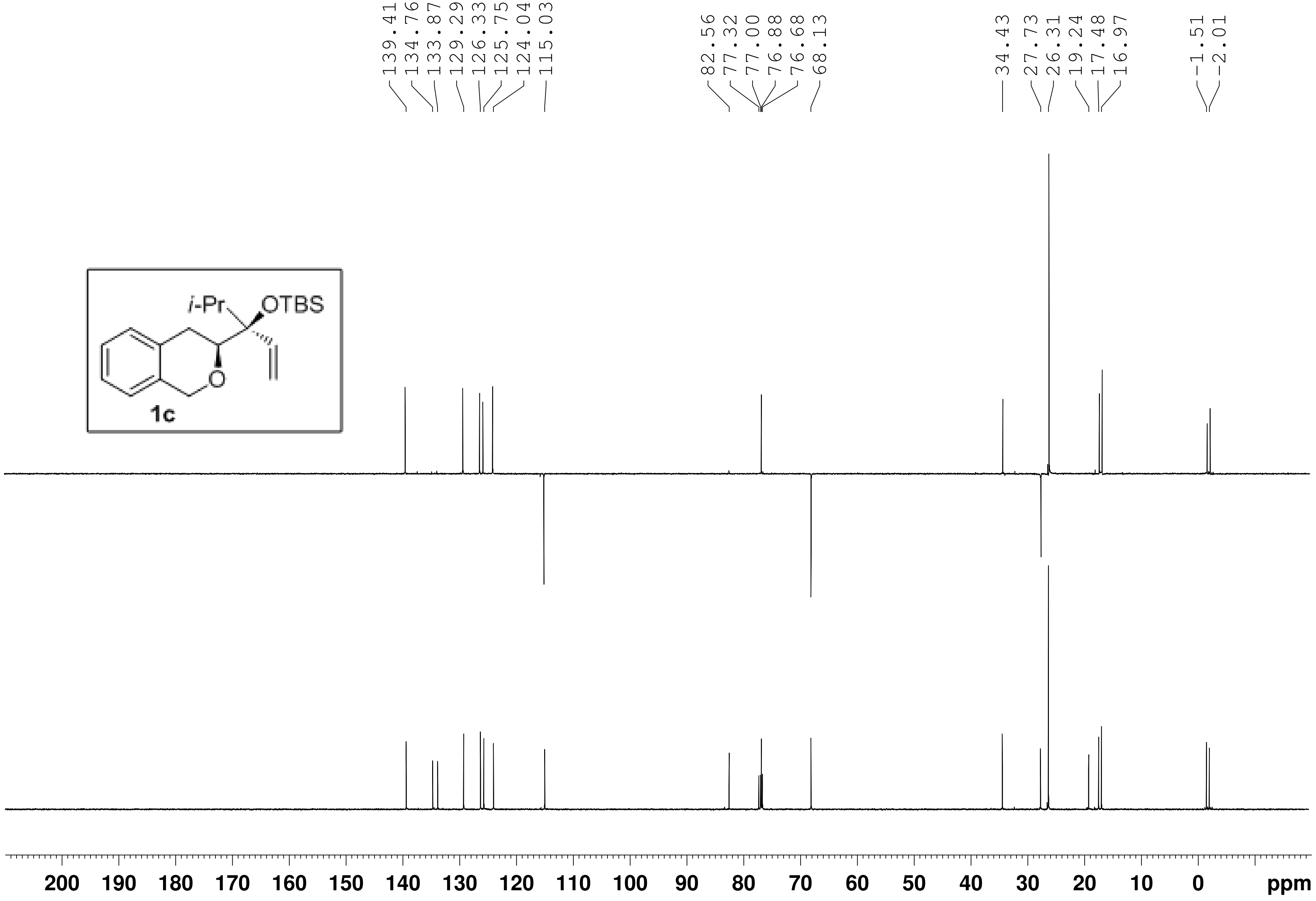

Supplementary Figure 25. ^{1}H NMR Spectrum of 2c (400 MHz, $CDCl_3$)

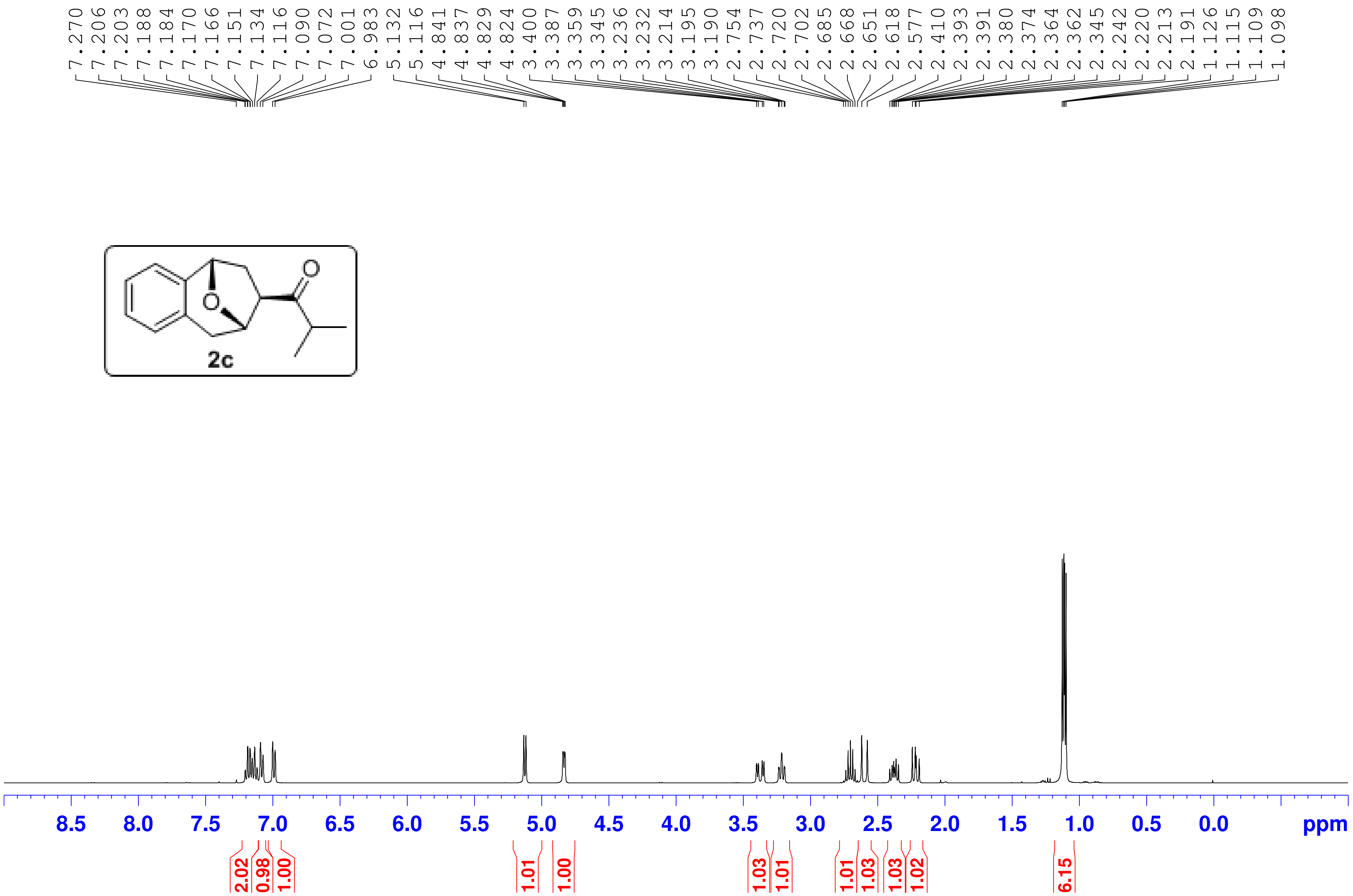

Supplementary Figure 26. ^{13}C NMR Spectrum of 2c (100 MHz, $CDCl_3$)

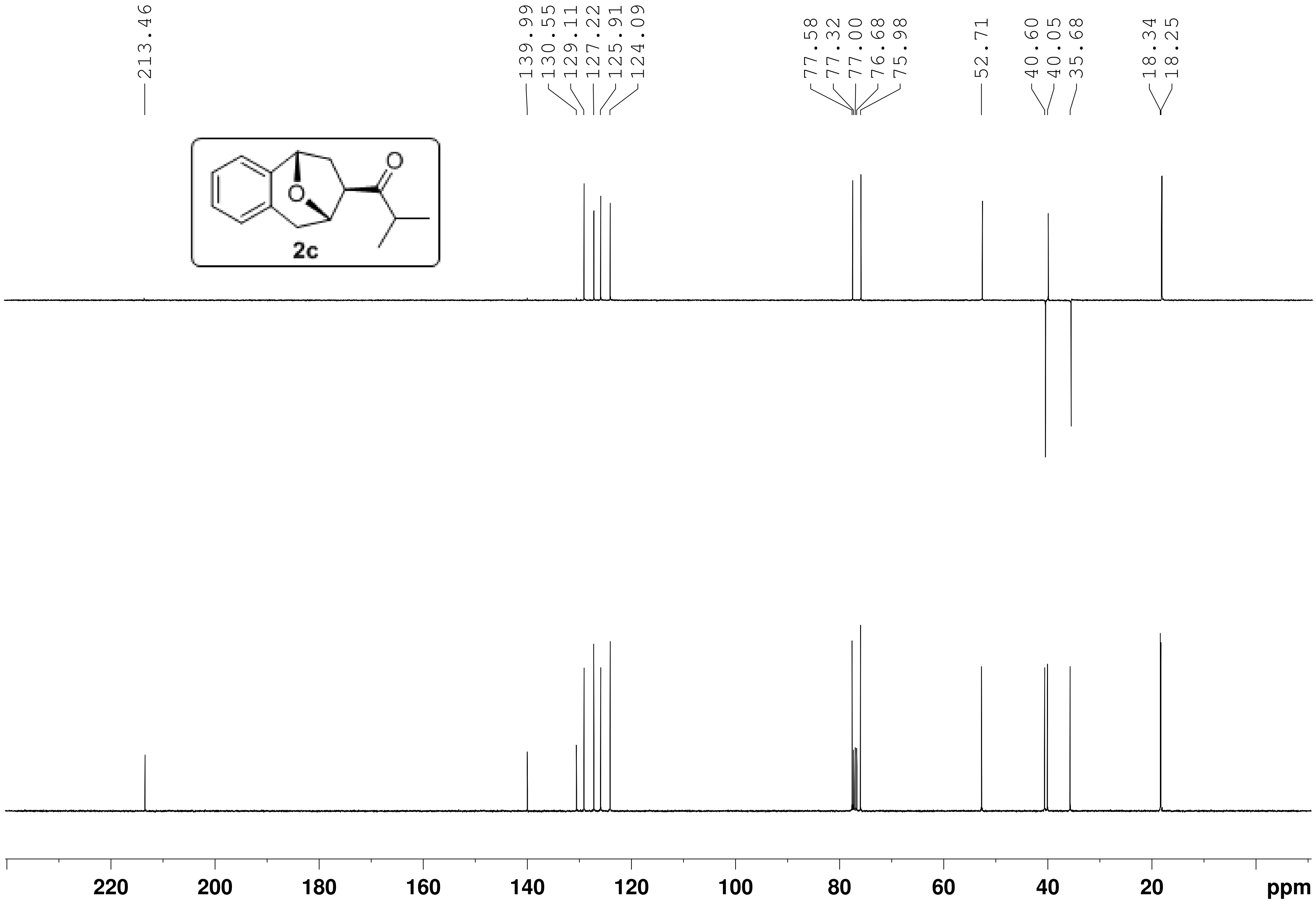

Supplementary Figure 27. ^{1}H NMR Spectrum of S9 (400 MHz, $CDCl_3$)

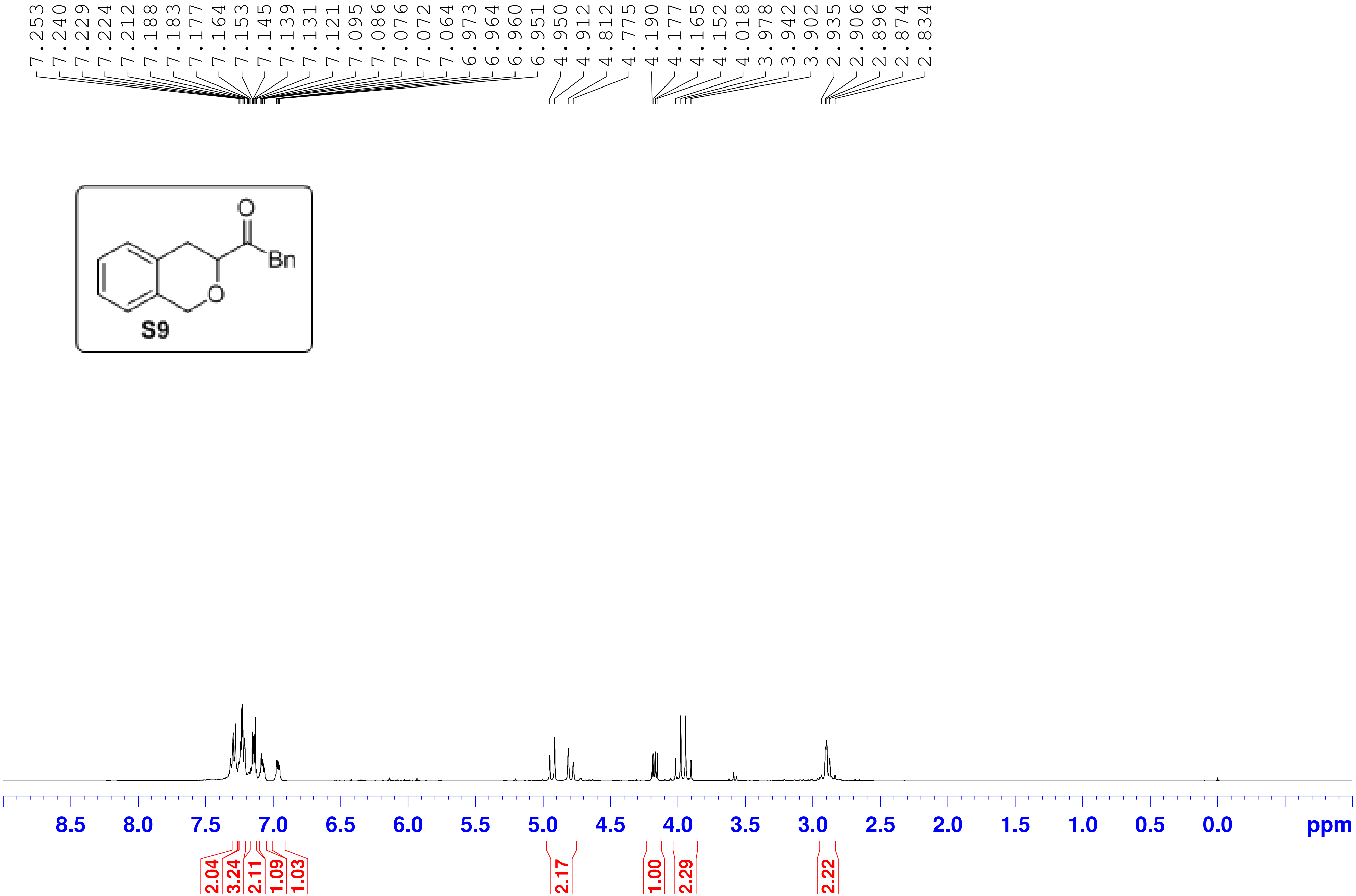

Supplementary Figure 28. ^{13}C NMR Spectrum of S9 (100 MHz, $CDCl_3$)

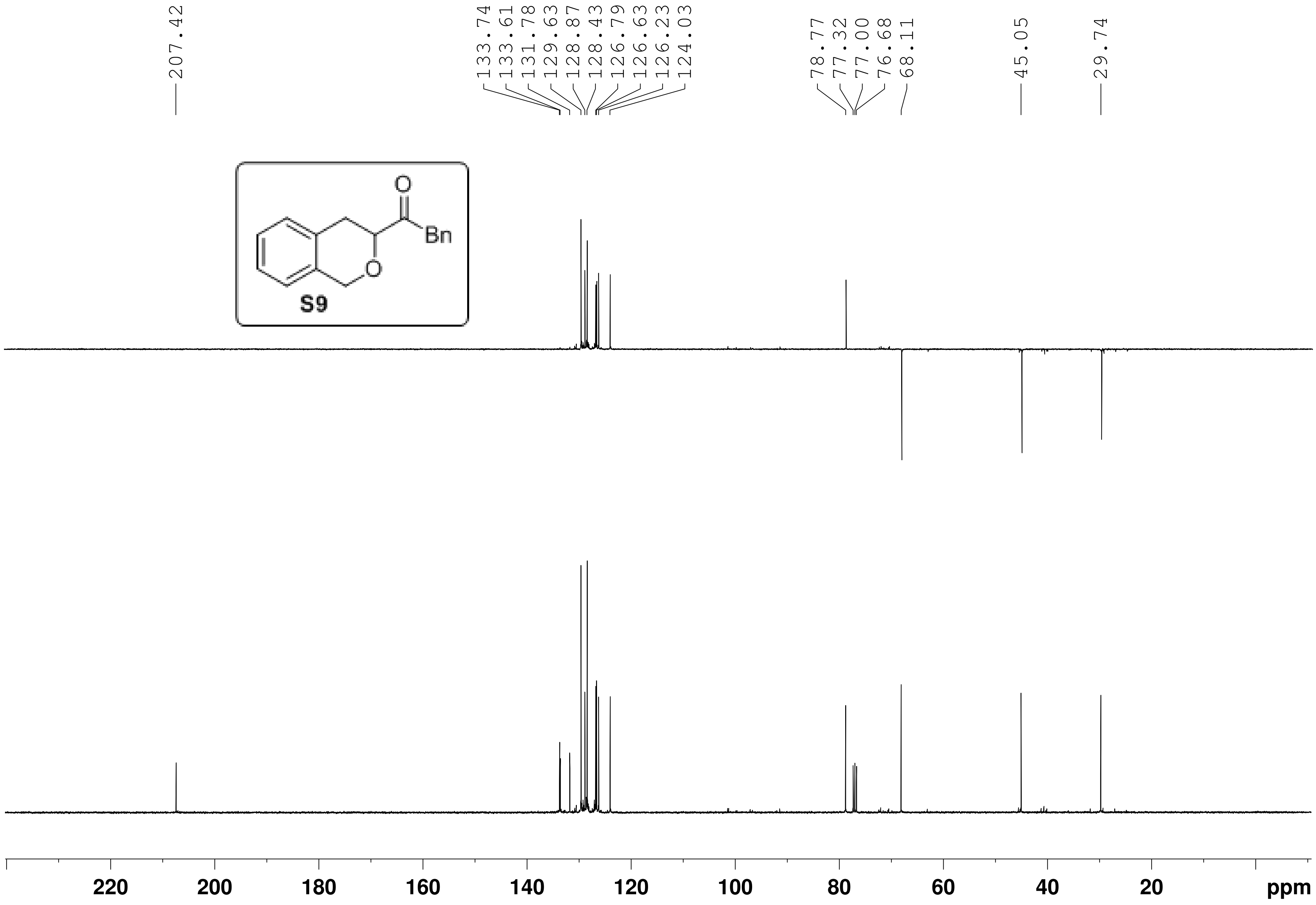

Supplementary Figure 29. ^{1}H NMR Spectrum of S10 (400 MHz, $CDCl_3$)

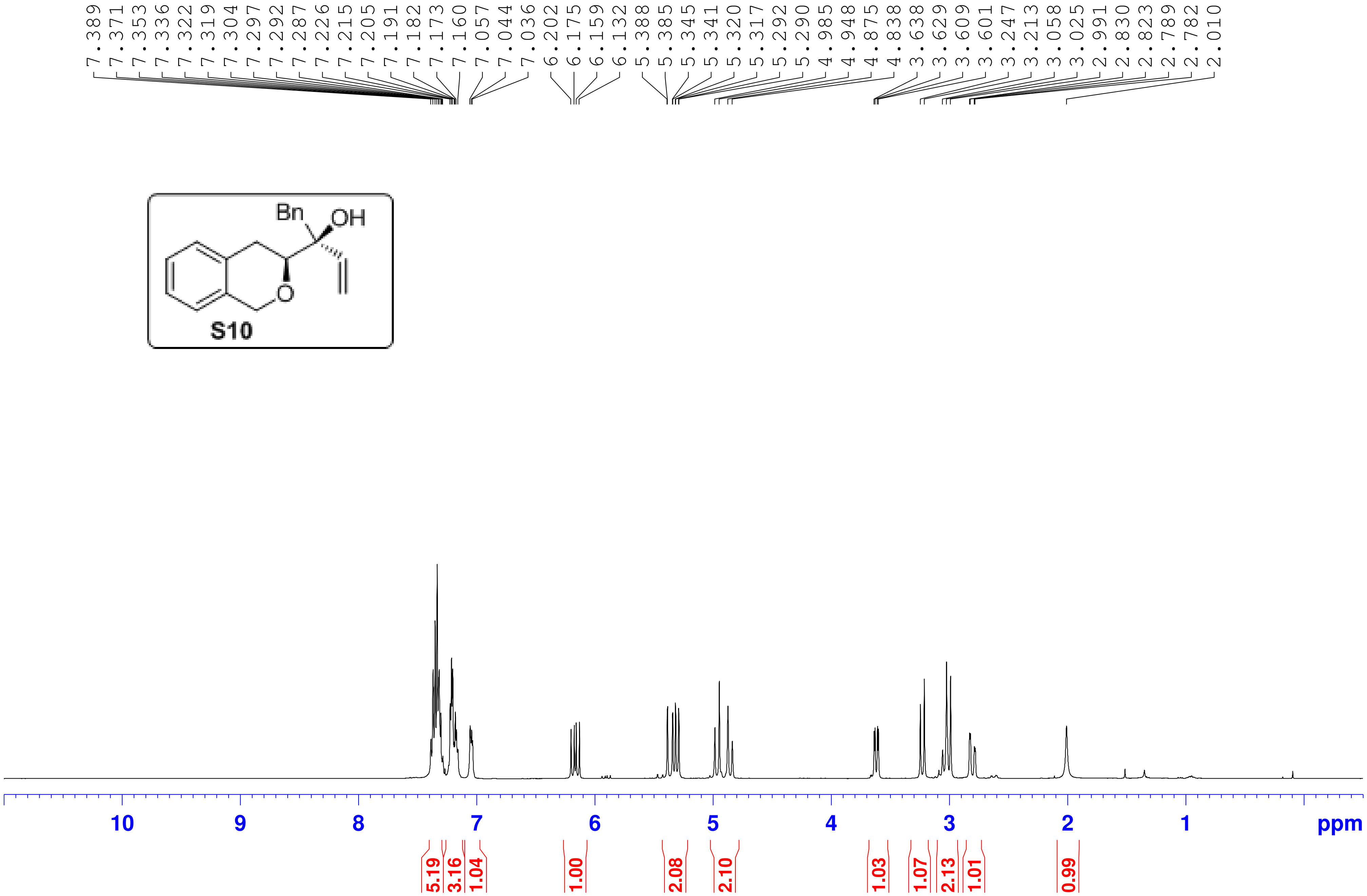

Supplementary Figure 30. ^{13}C NMR Spectrum of S10 (100 MHz, $CDCl_3$)

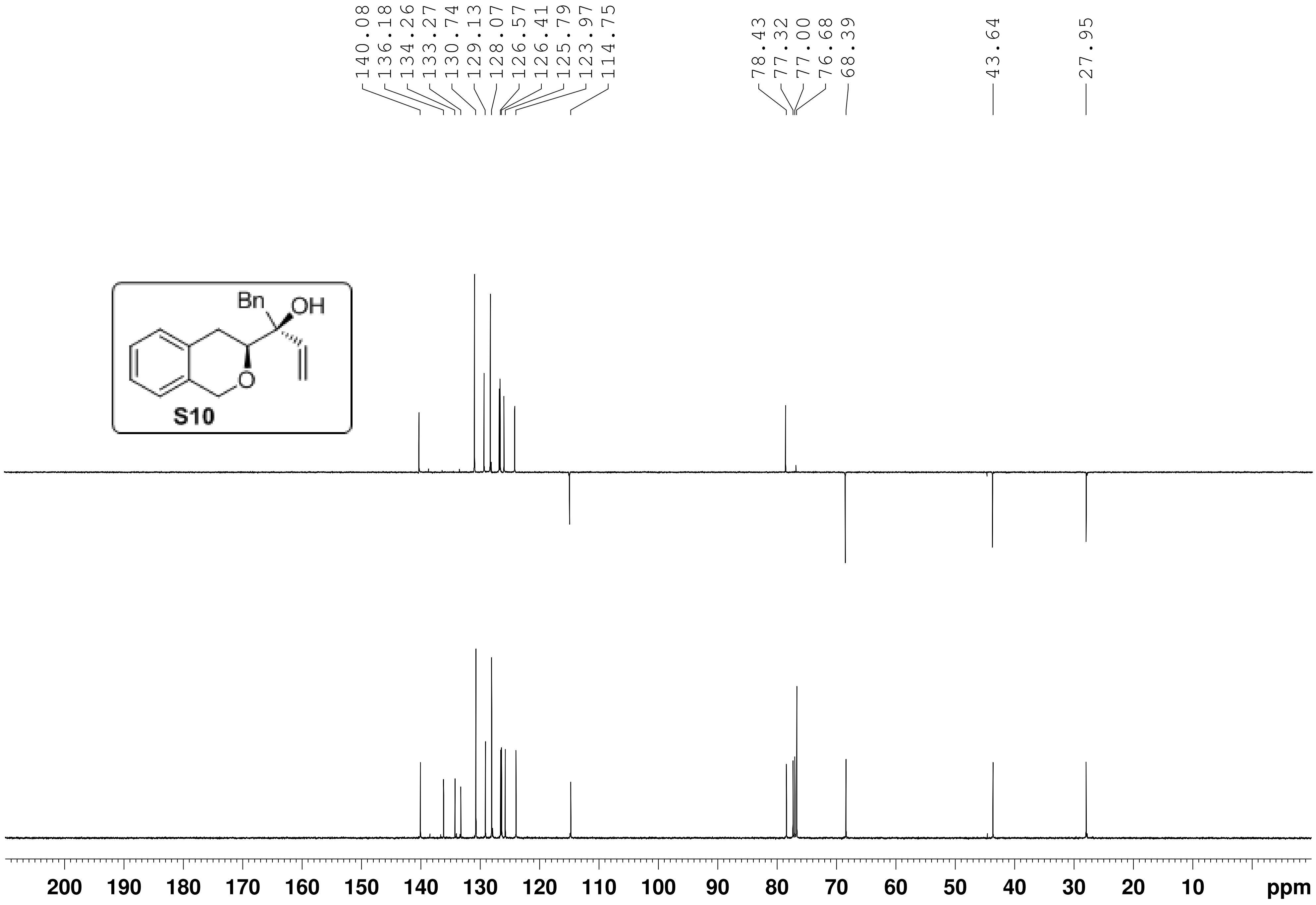

Supplementary Figure 31. ^{1}H NMR Spectrum of 1d (400 MHz, $CDCl_3$)

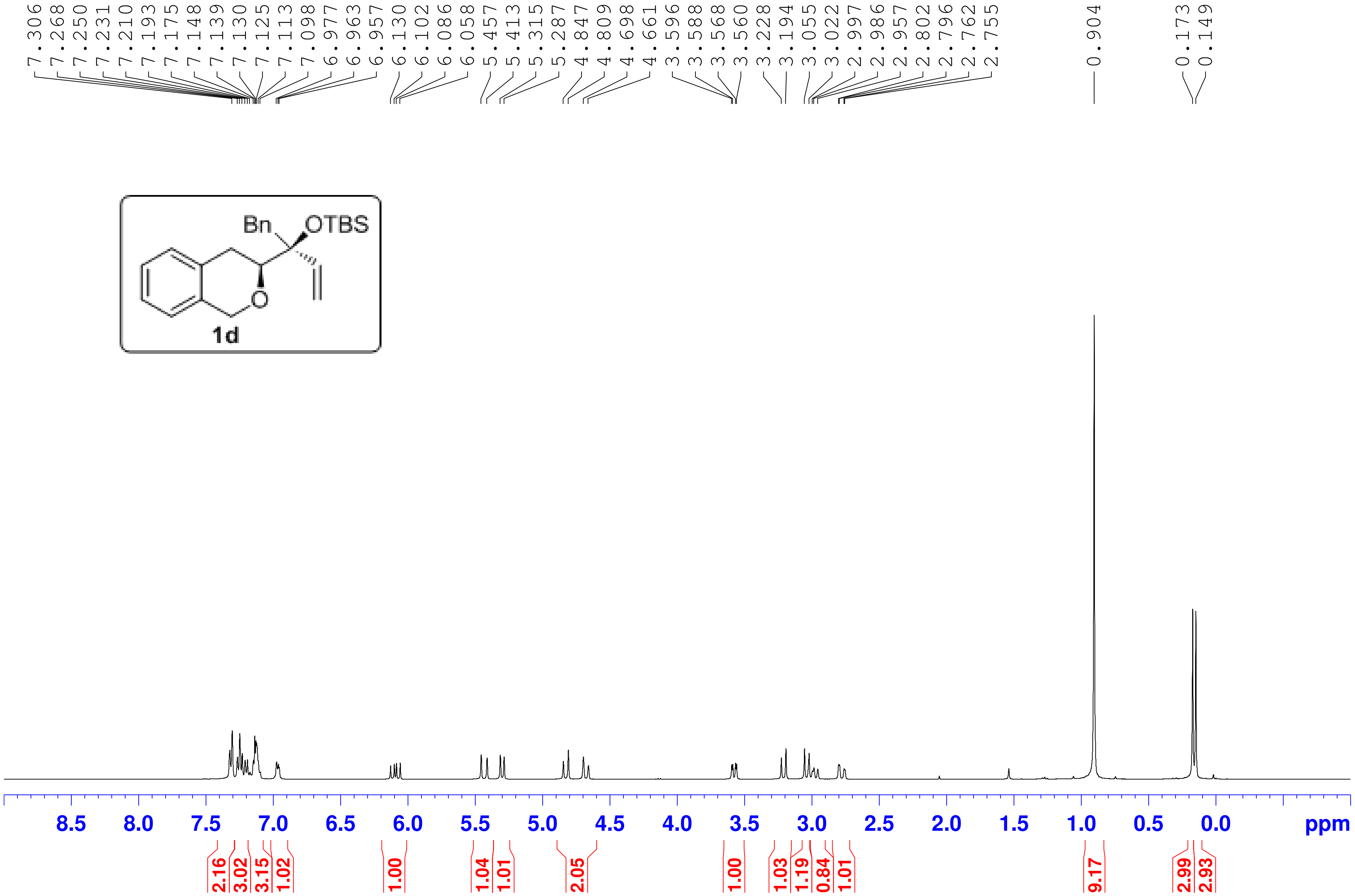

Supplementary Figure 32. ^{13}C NMR Spectrum of 1d (100 MHz, $CDCl_3$)

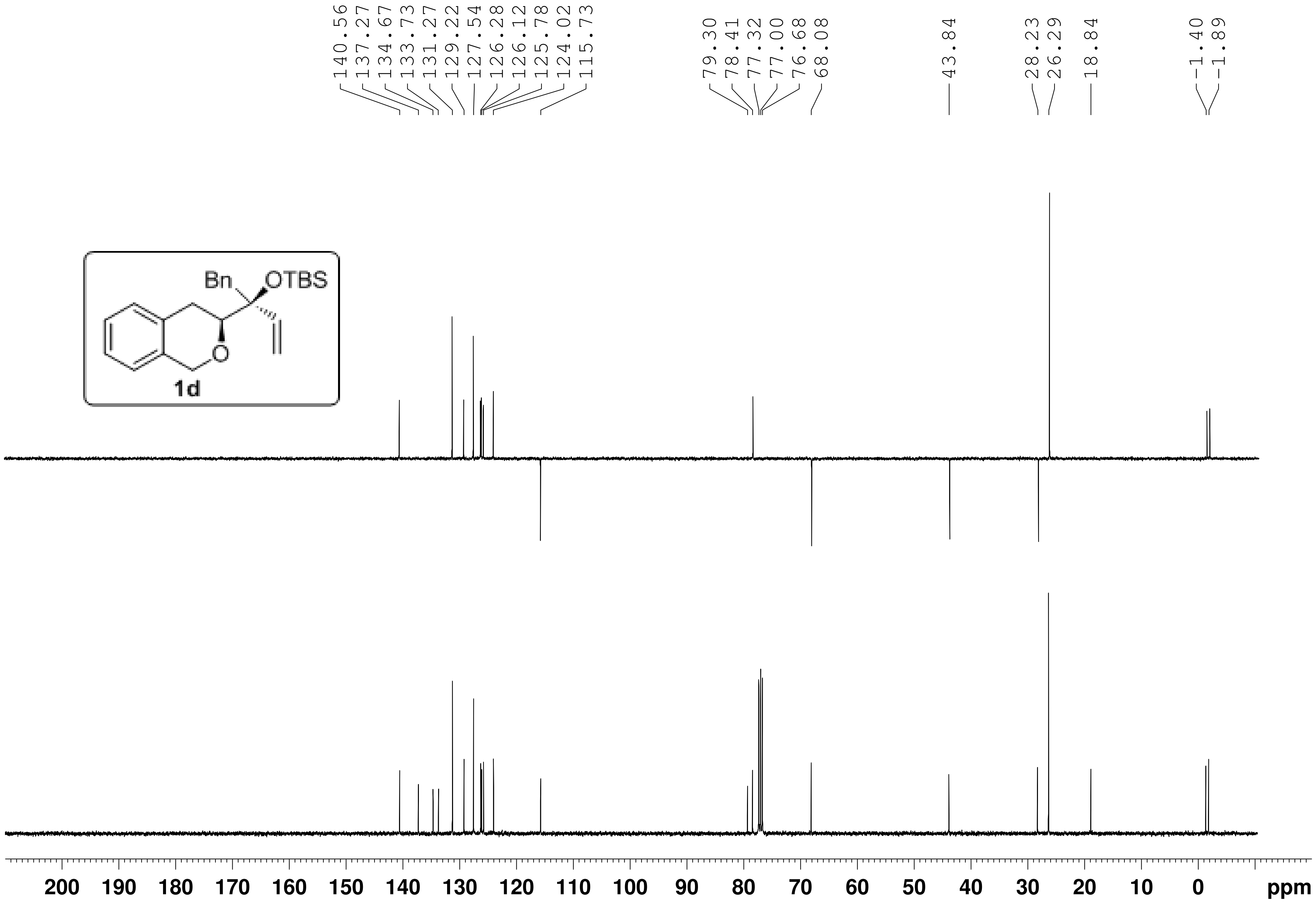

Supplementary Figure 33. ¹H NMR Spectrum of 2d (400 MHz, CDCl₃)

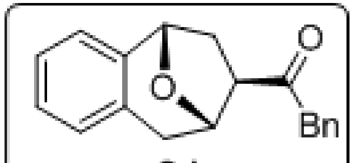

2d

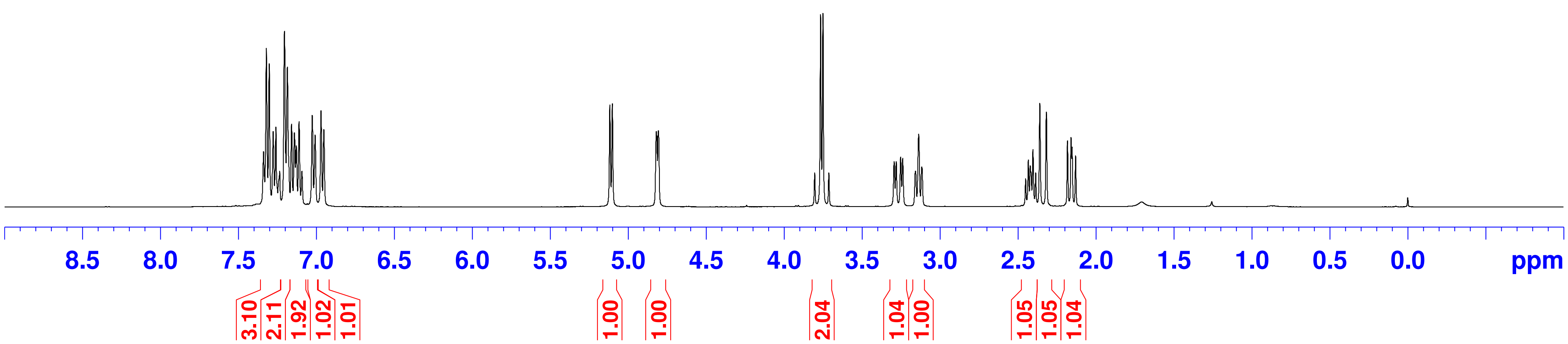

Supplementary Figure 34. ^{13}C NMR Spectrum of 2d (100 MHz, $CDCl_3$)

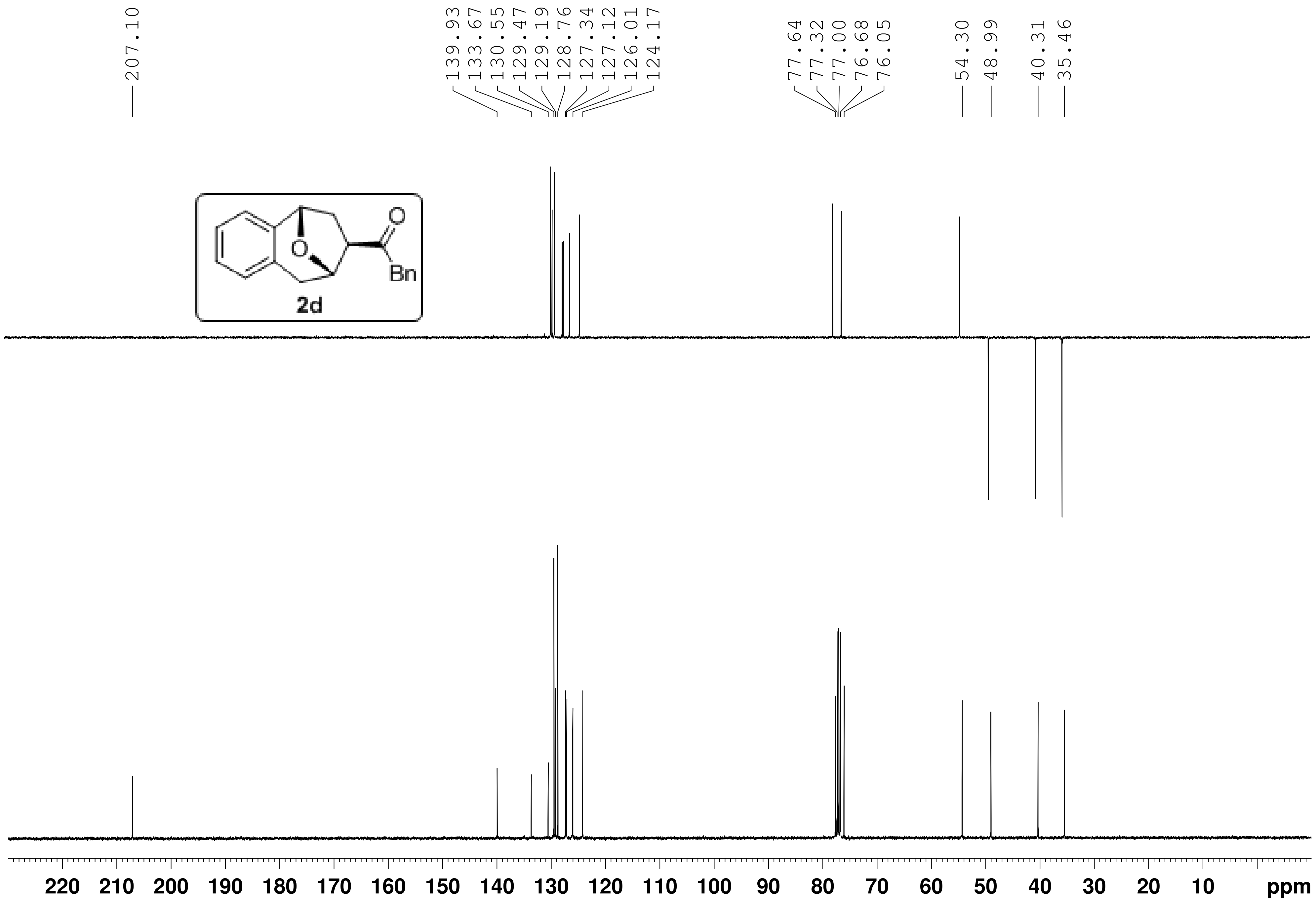

Supplementary Figure 35. ^{1}H NMR Spectrum of S11 (400 MHz, $CDCl_3$)

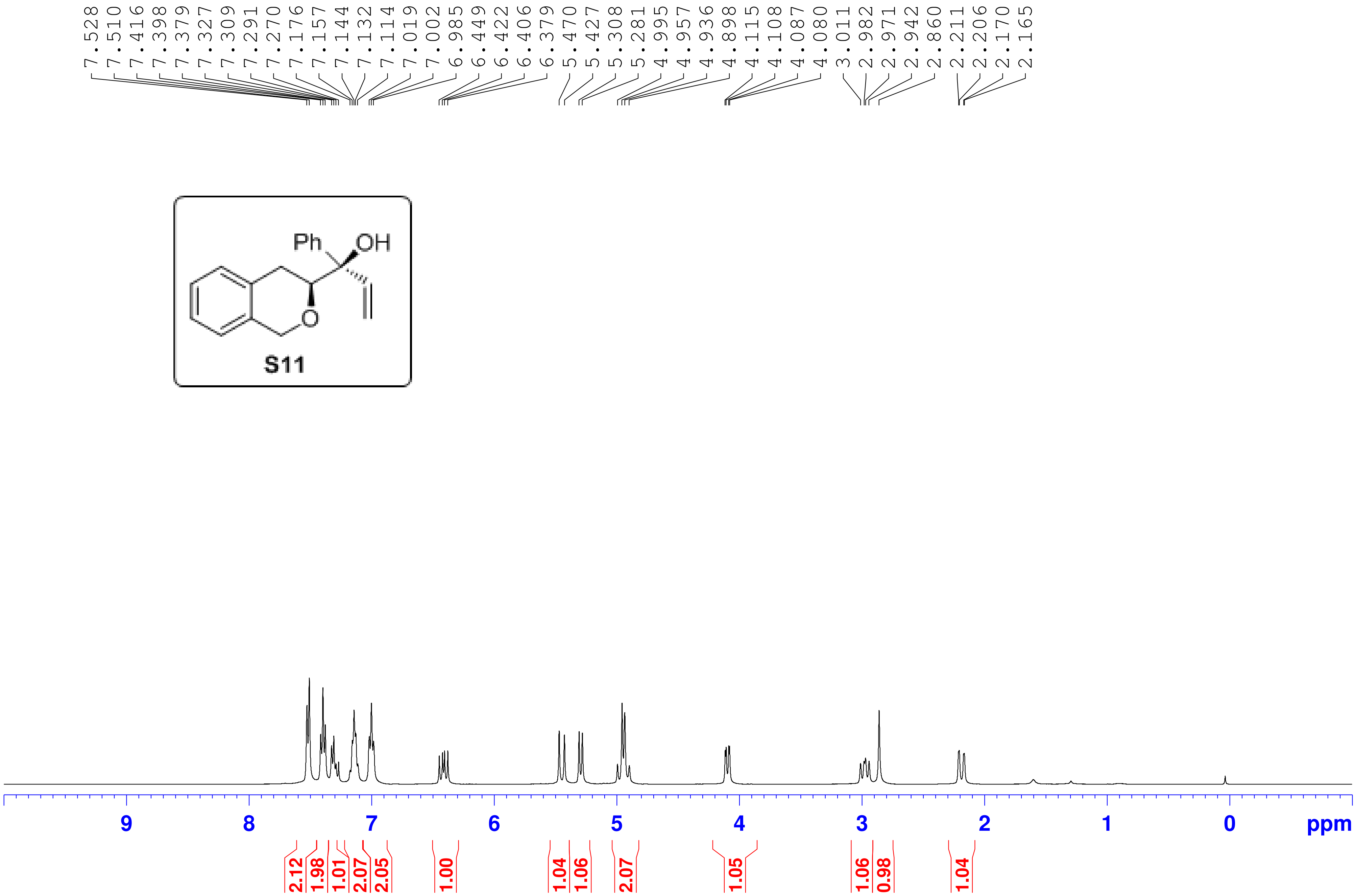

Supplementary Figure 36. ^{13}C NMR Spectrum of S11 (100 MHz, $CDCl_3$)

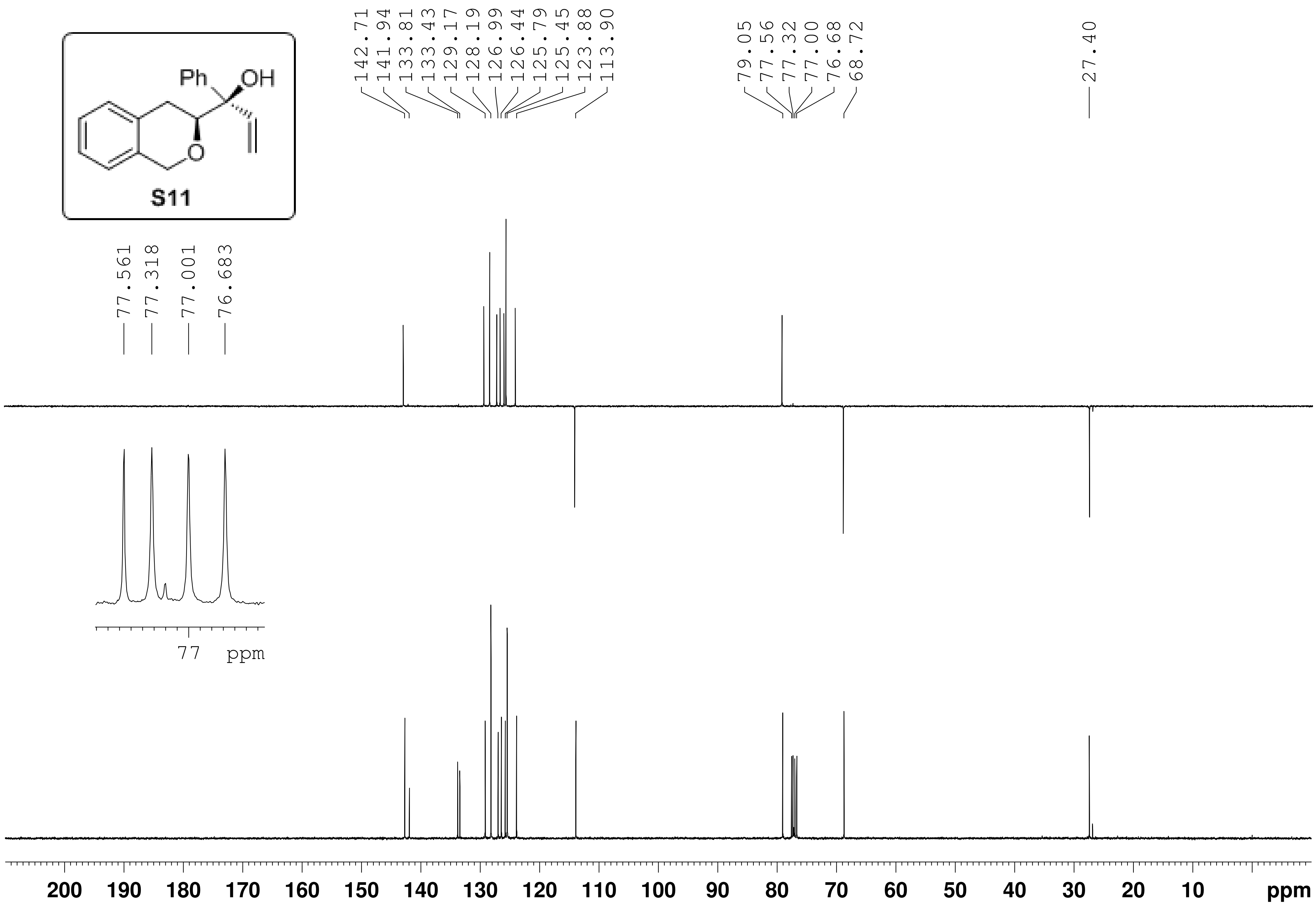

Supplementary Figure 37. ^{1}**H NMR Spectrum of 1e (400 MHz, CDCl$_3$)**

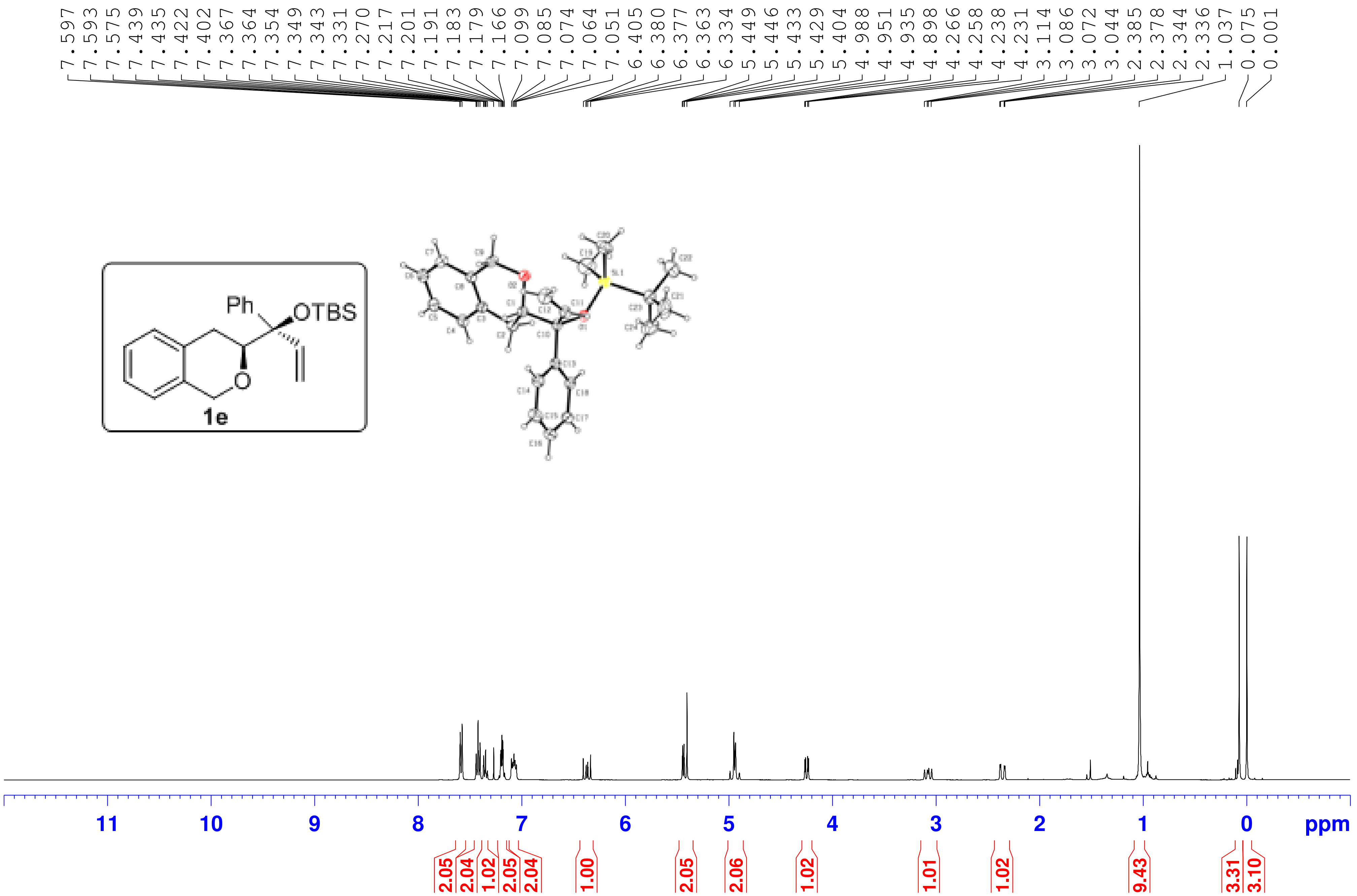

Supplementary Figure 38. ^{13}C NMR Spectrum of 1e (100 MHz, $CDCl_3$)

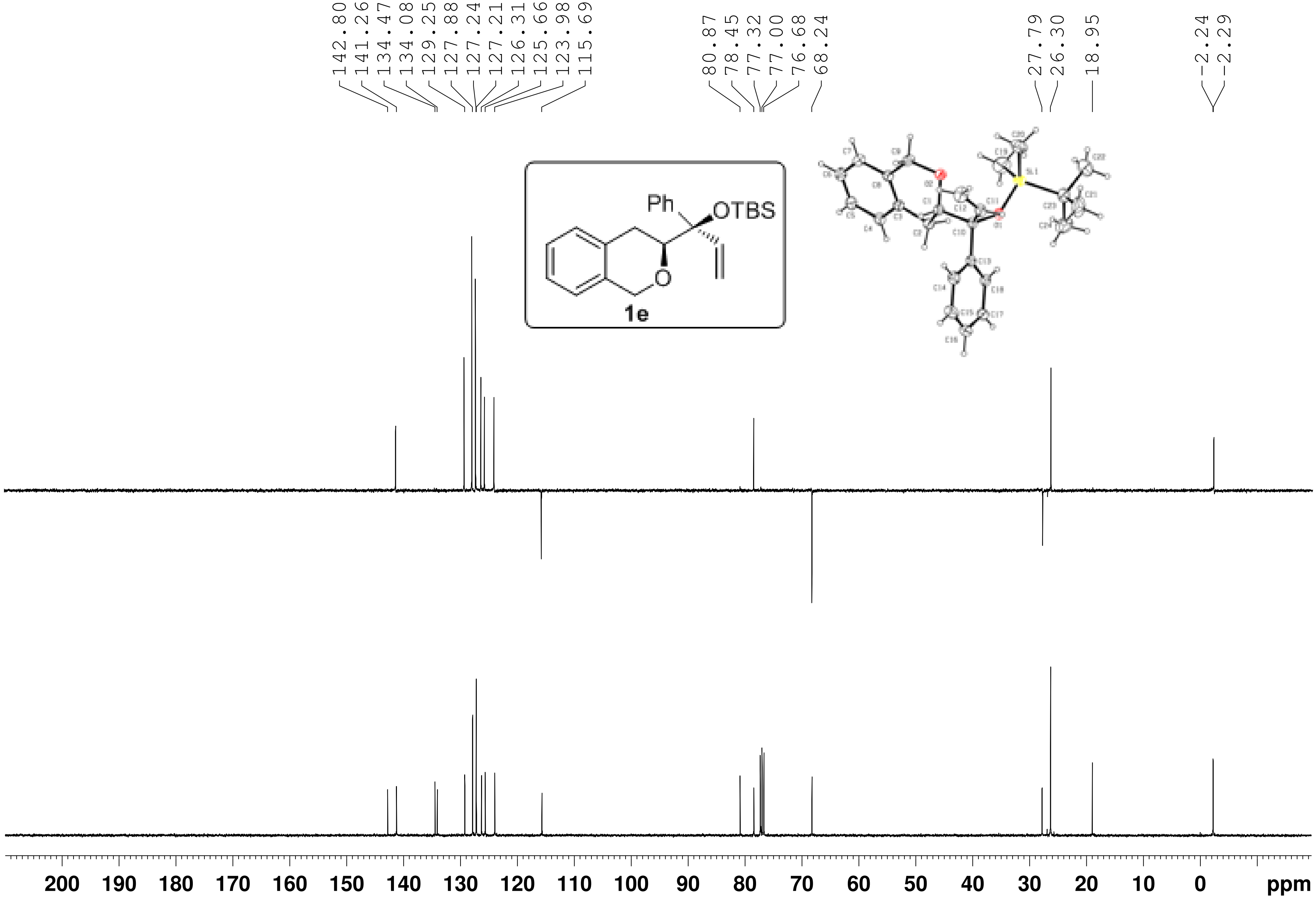

Supplementary Figure 39. ^{1}H NMR Spectrum of 2e (400 MHz, $CDCl_3$)

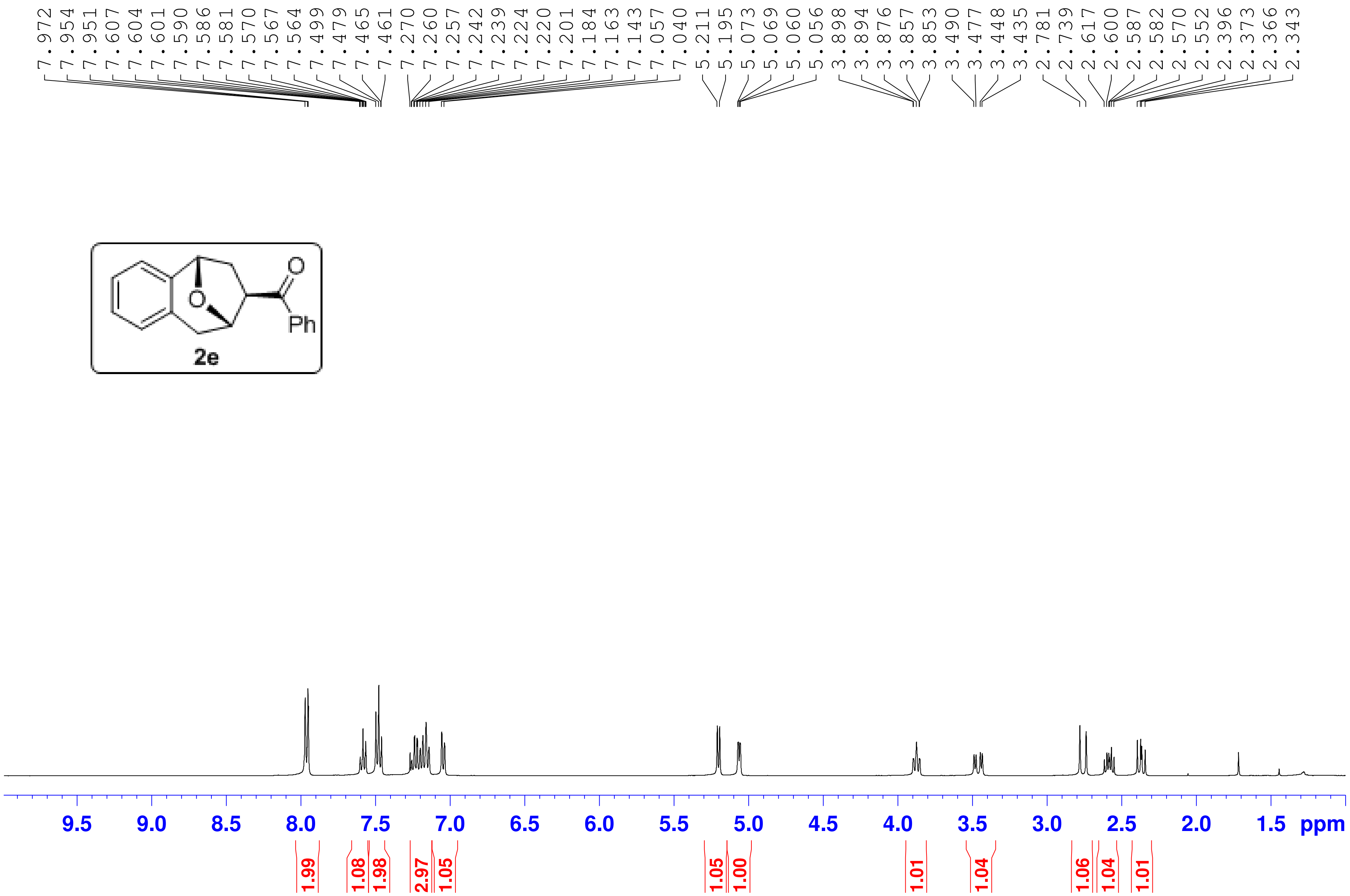

Supplementary Figure 40. ^{13}C NMR Spectrum of 2e (100 MHz, $CDCl_3$)

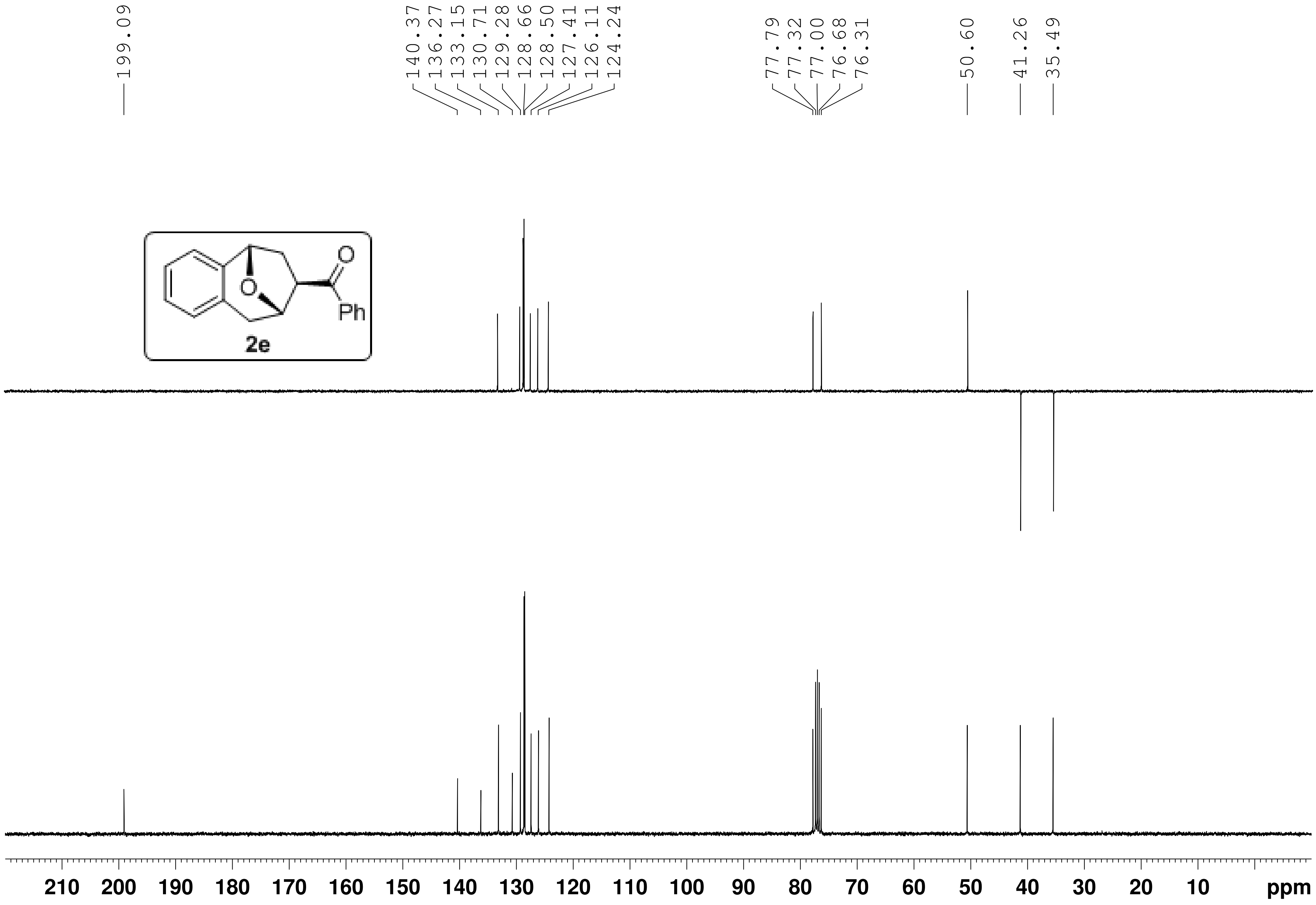

Supplementary Figure 41. ^{1}H NMR Spectrum of S13 (400 MHz, $CDCl_3$)

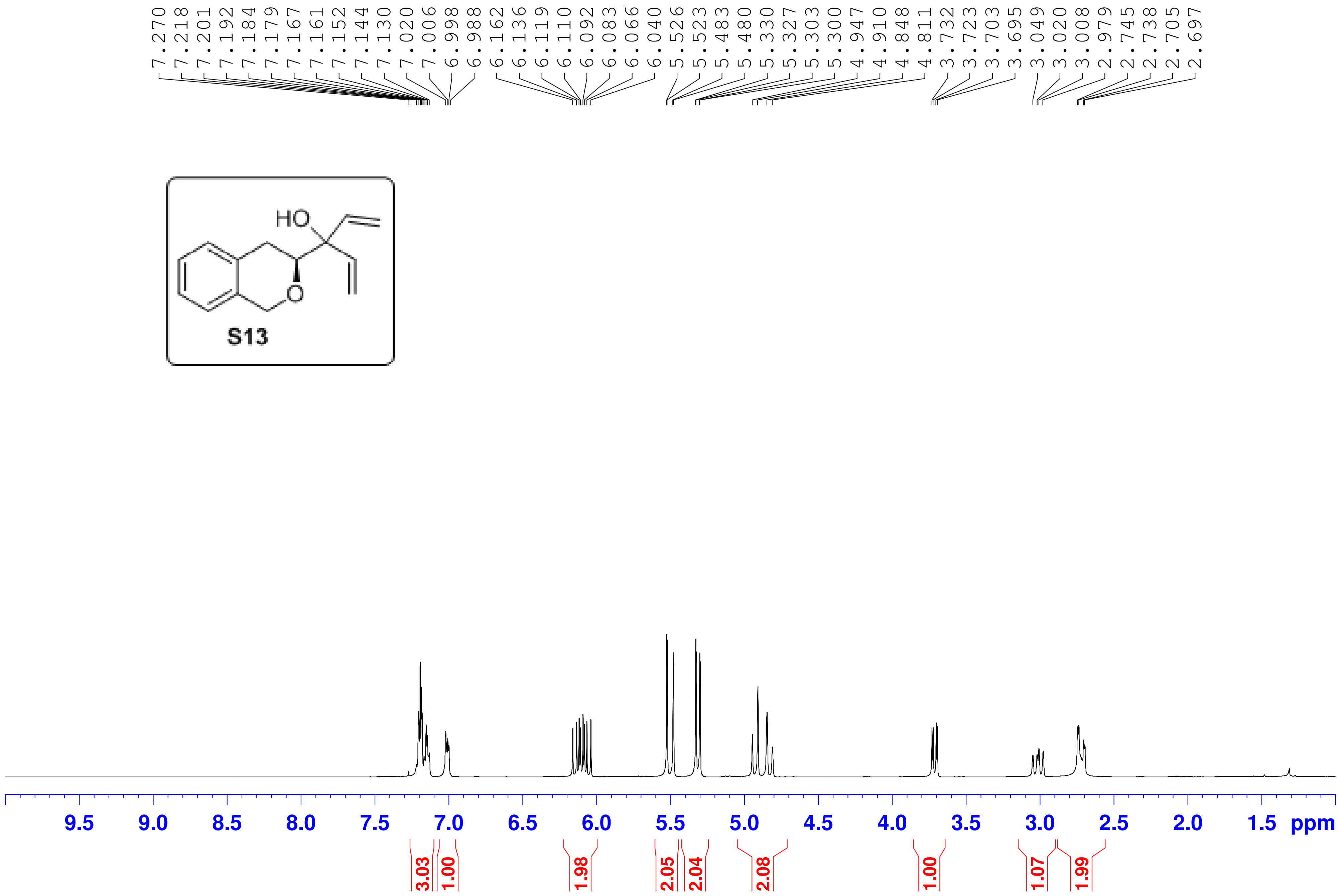

Supplementary Figure 42. ^{13}C NMR Spectrum of S13 (100 MHz, $CDCl_3$)

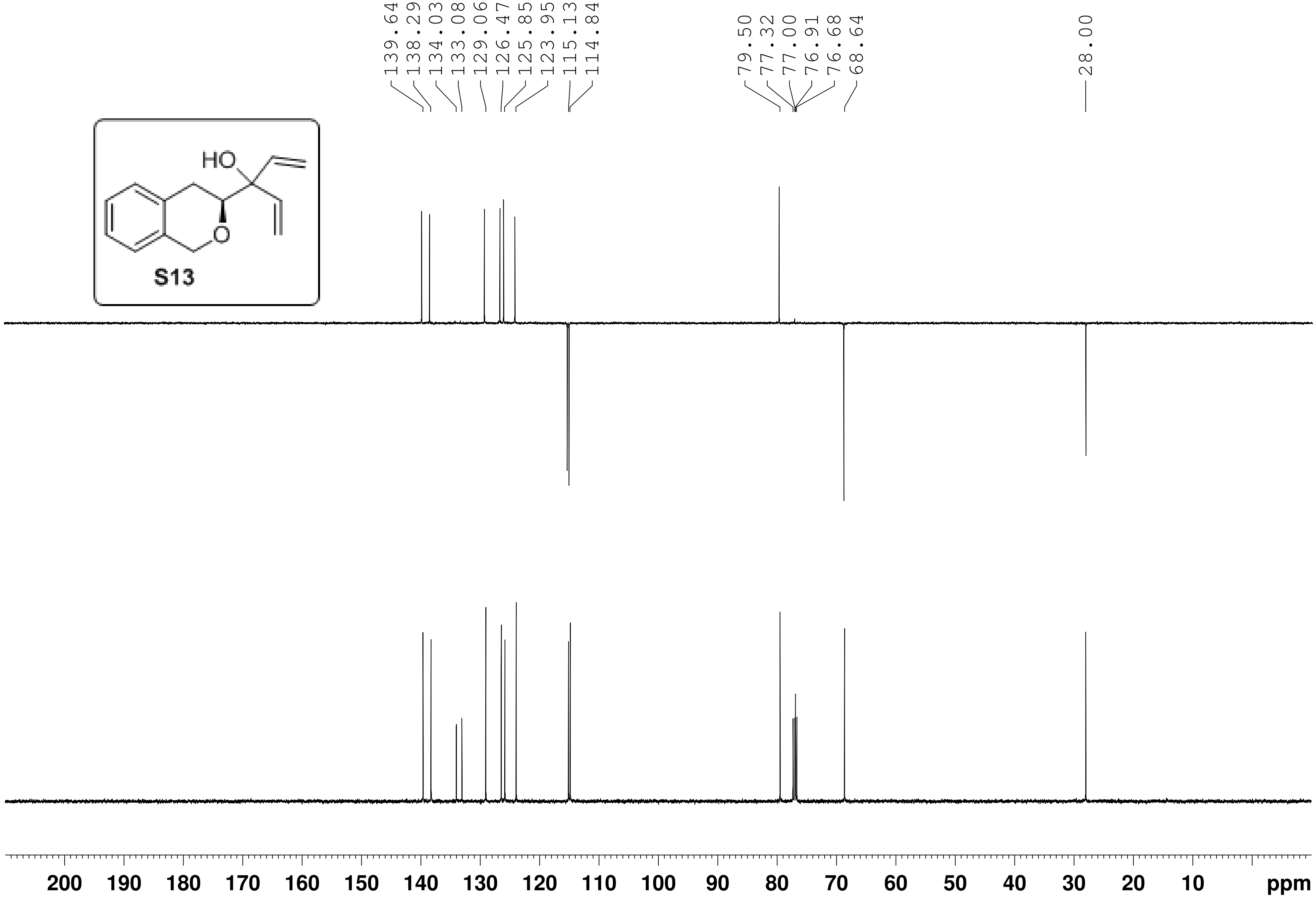

Supplementary Figure 43. ^{1}H NMR Spectrum of 1f (400 MHz, $CDCl_3$)

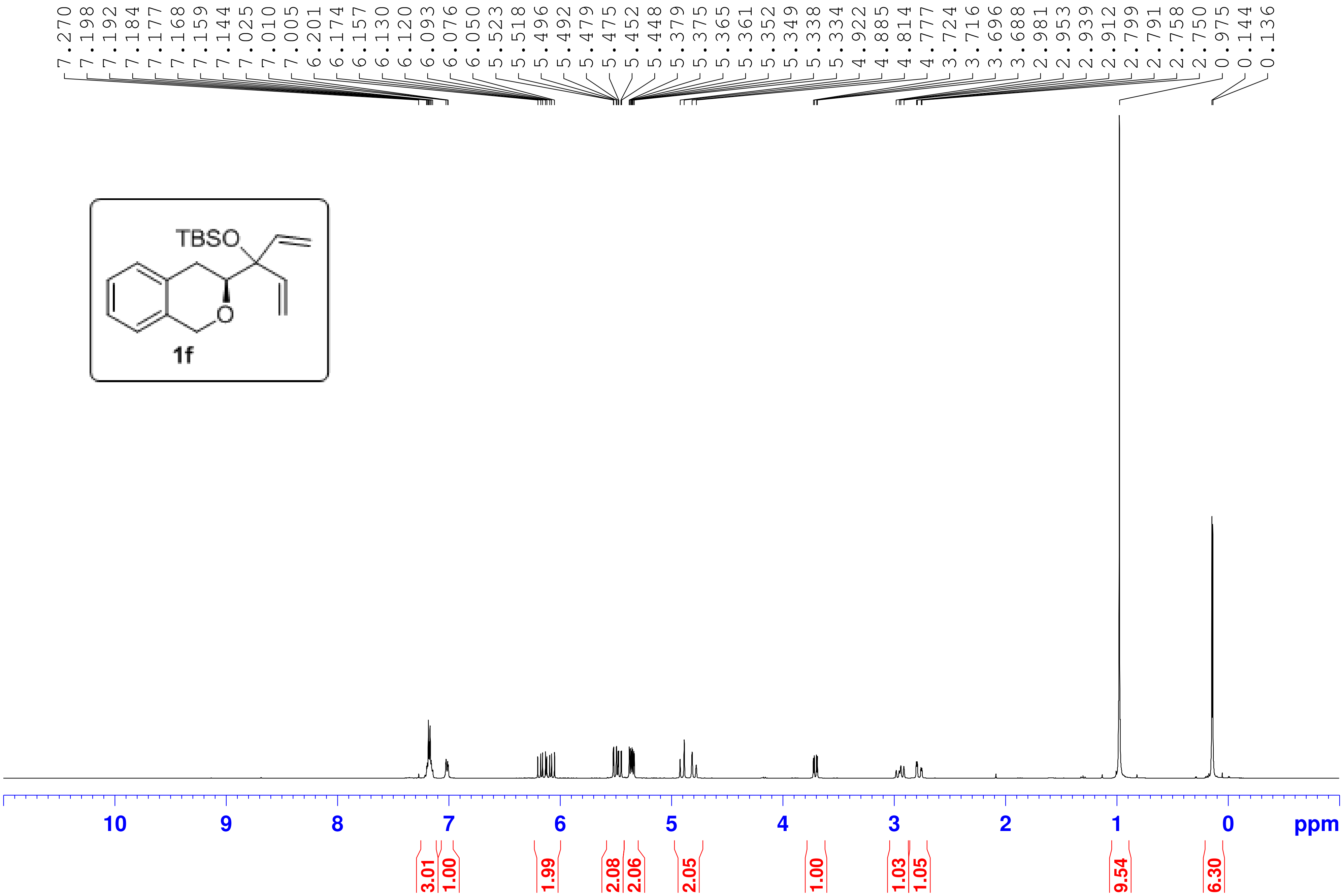

Supplementary Figure 44. ^{13}C NMR Spectrum of 1f (100 MHz, $CDCl_3$)

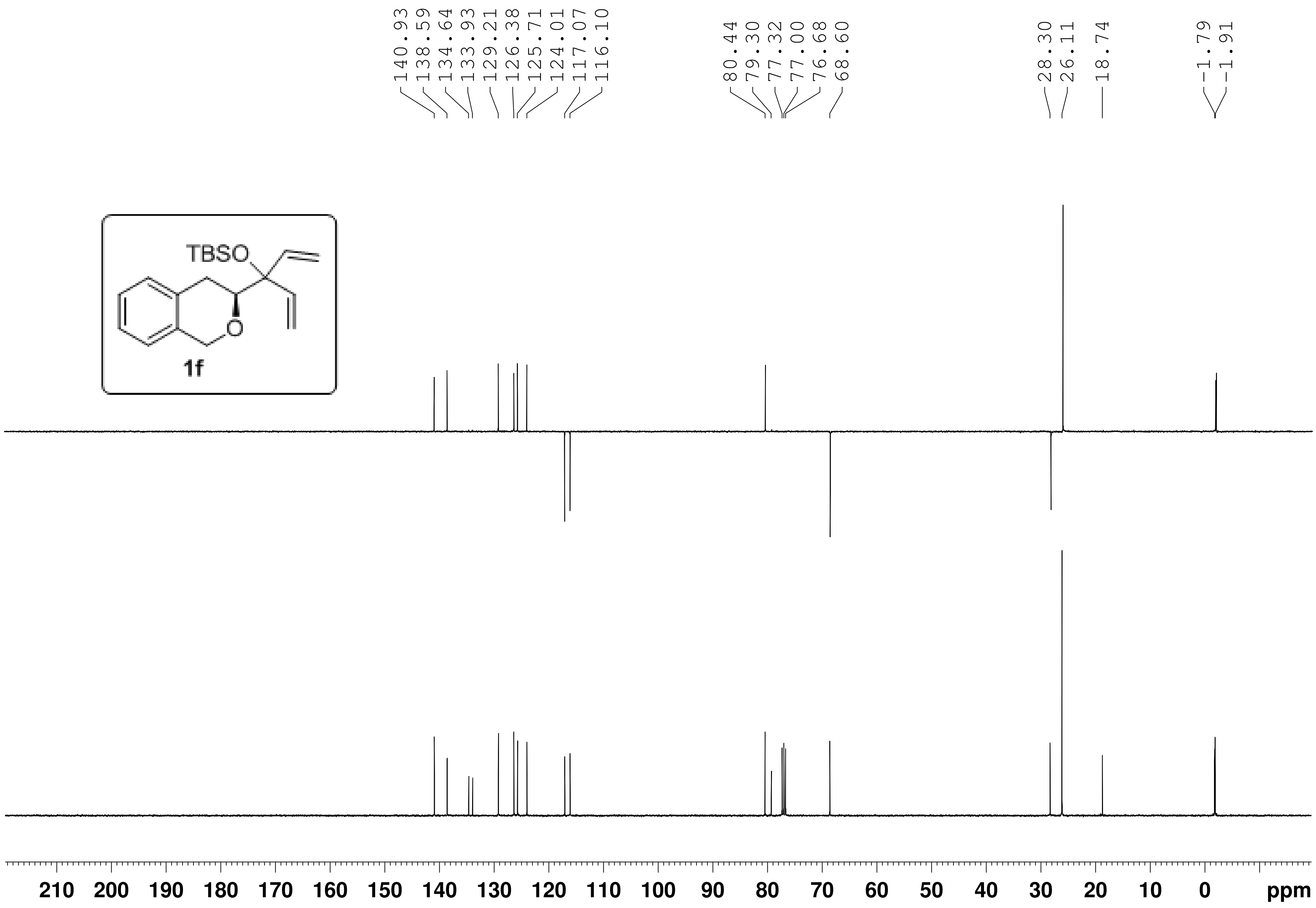

Supplementary Figure 45. ^{1}H NMR Spectrum of 2f (400 MHz, $CDCl_3$)

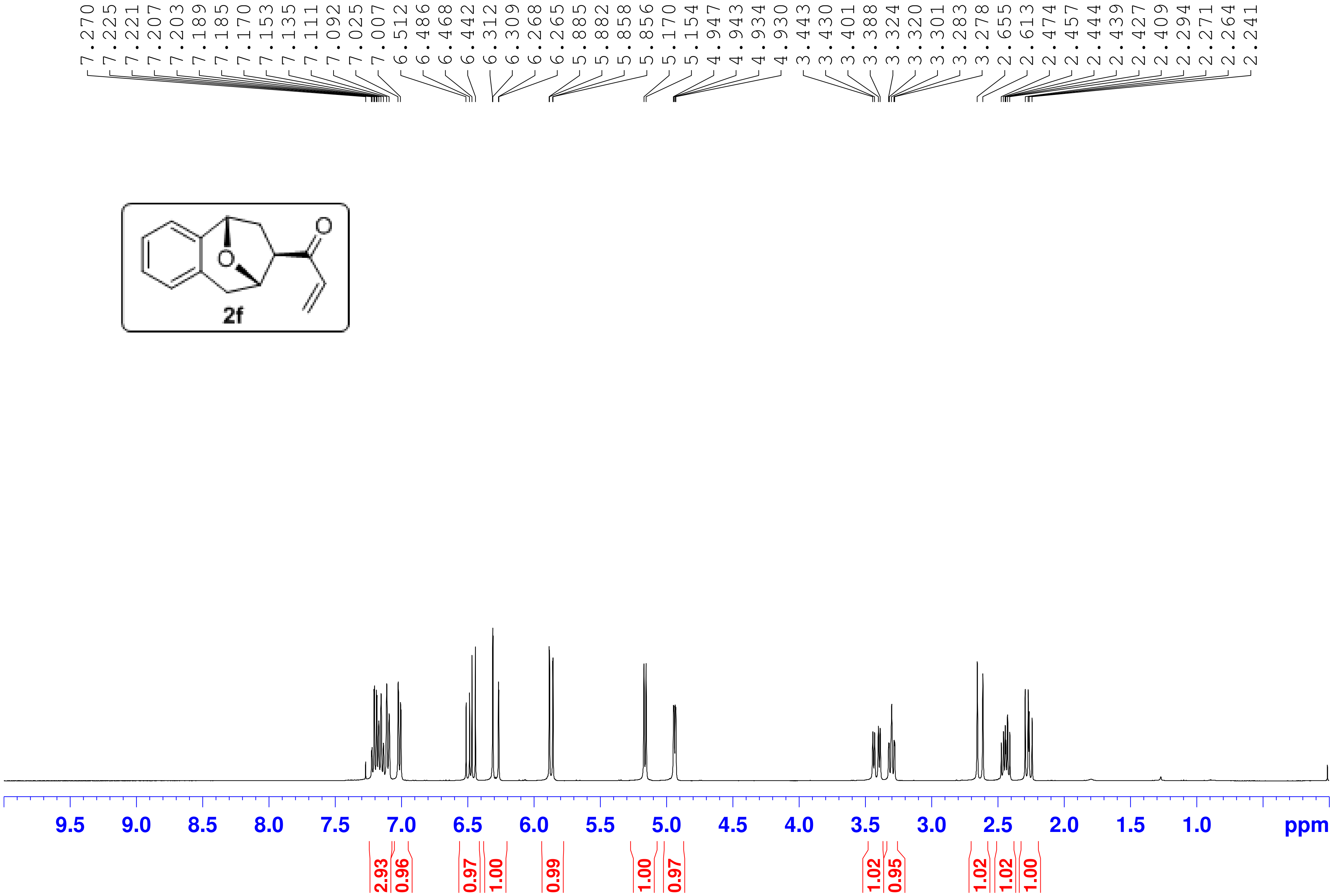

Supplementary Figure 46. ^{13}C NMR Spectrum of 2f (100 MHz, $CDCl_3$)

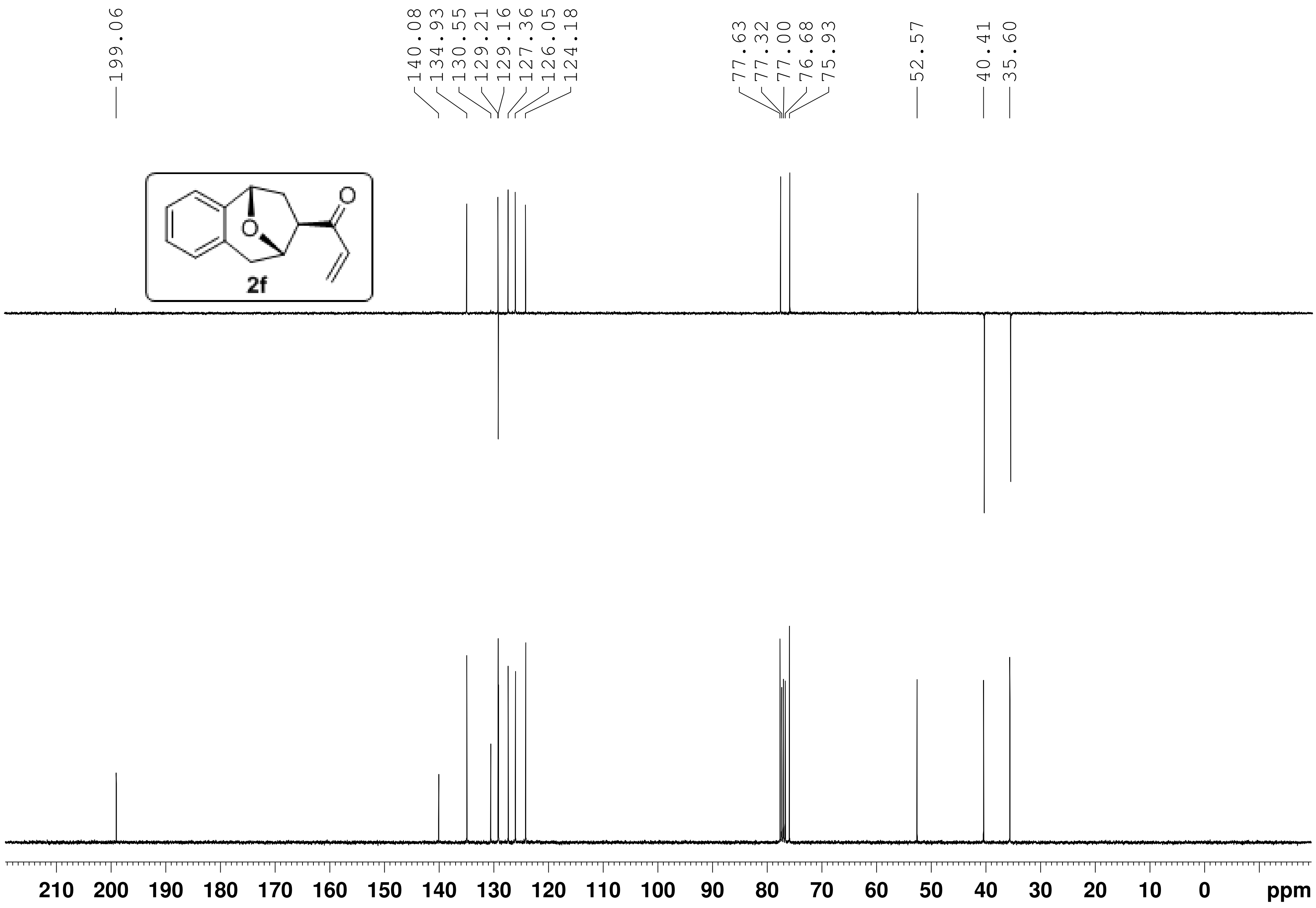

Supplementary Figure 47. ^{1}H NMR Spectrum of S14 (400 MHz, $CDCl_3$)

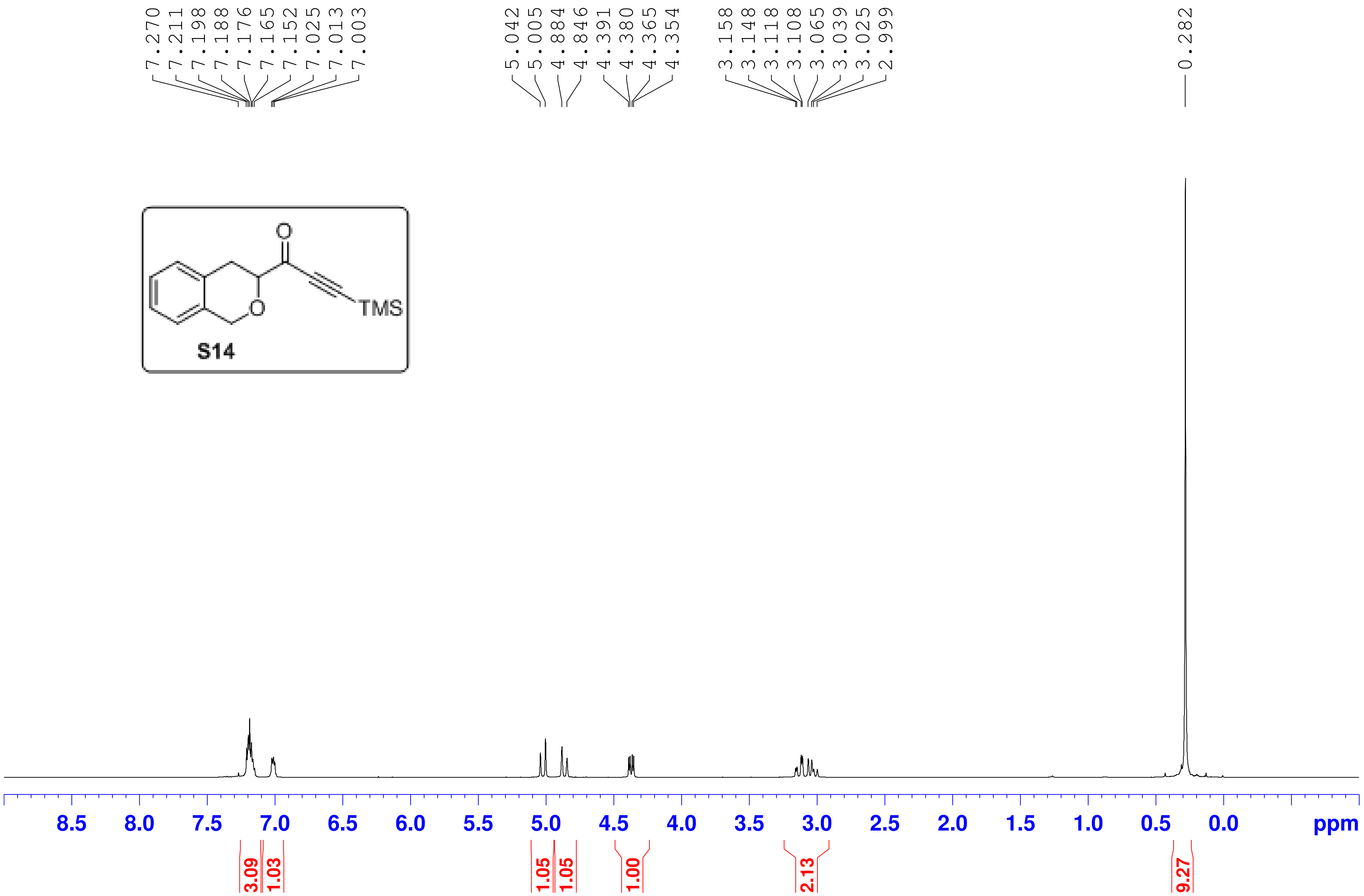

Supplementary Figure 48. ^{13}C NMR Spectrum of S14 (100 MHz, $CDCl_3$)

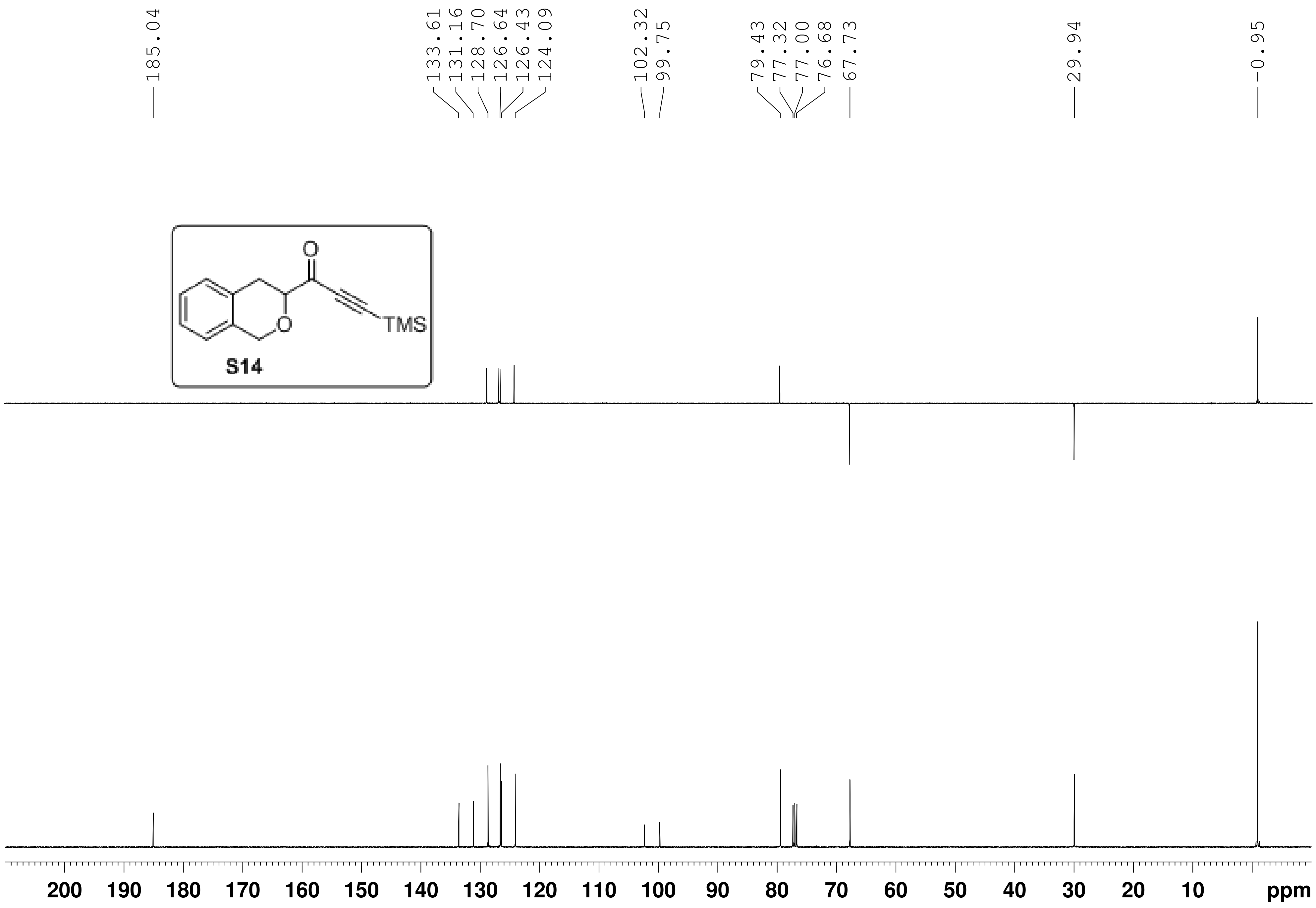

Supplementary Figure 49. ^{1}H NMR Spectrum of S15 (400 MHz, $CDCl_3$)

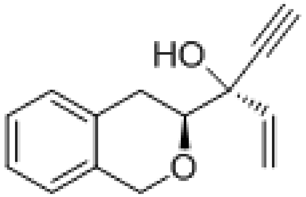

S15

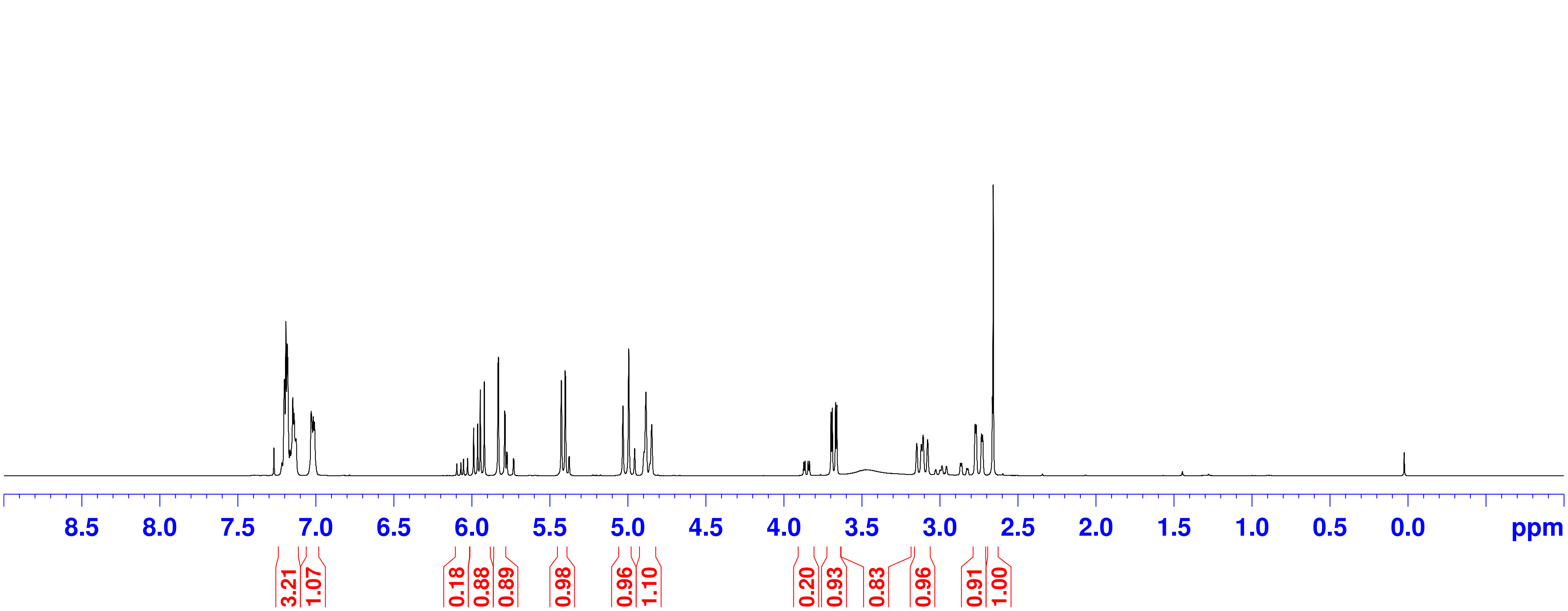

Supplementary Figure 50. ^{13}C NMR Spectrum of S15 (100 MHz, $CDCl_3$)

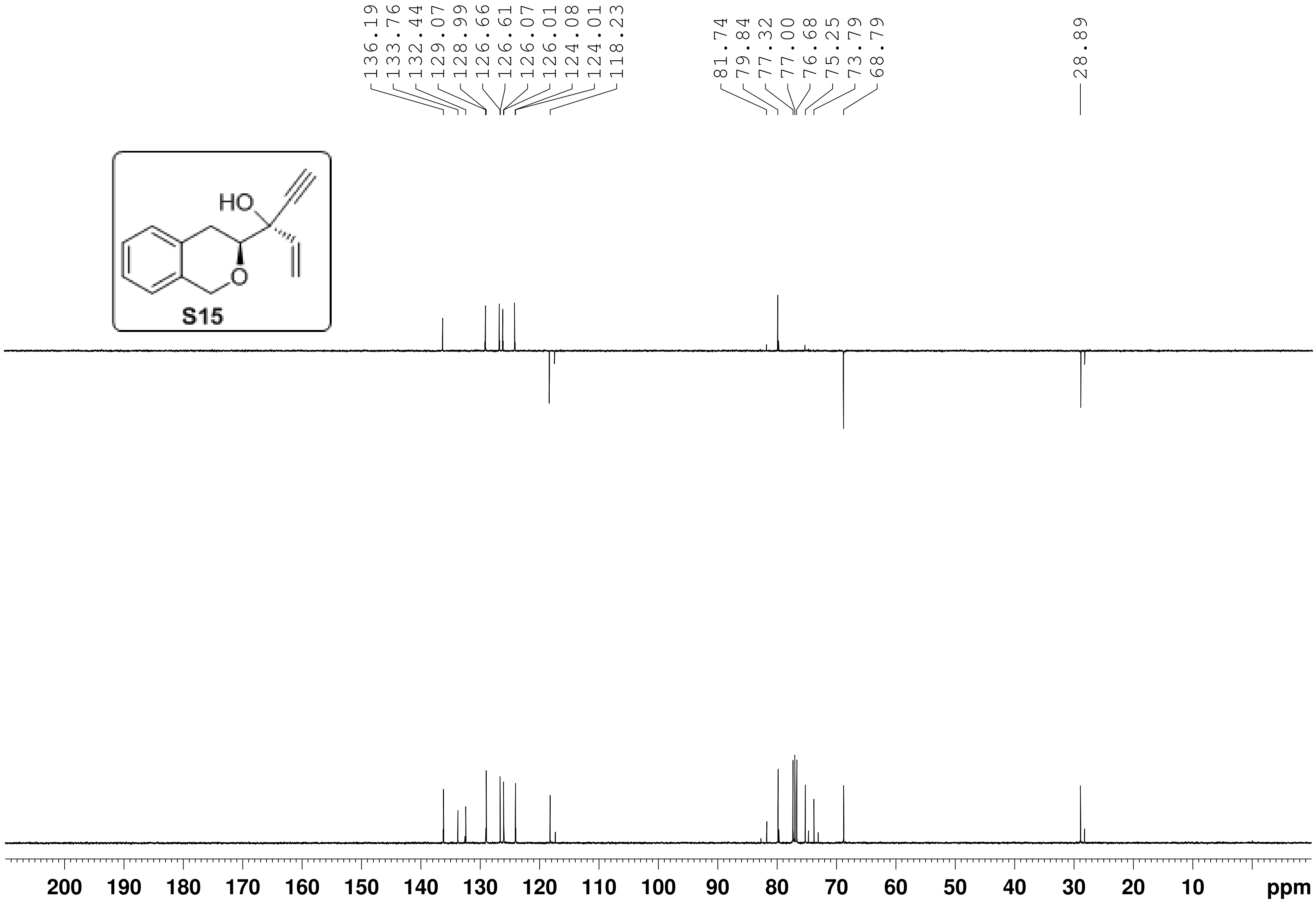

Supplementary Figure 51. ^{1}H **NMR Spectrum of 1g (400 MHz,** $CDCl_3$**)**

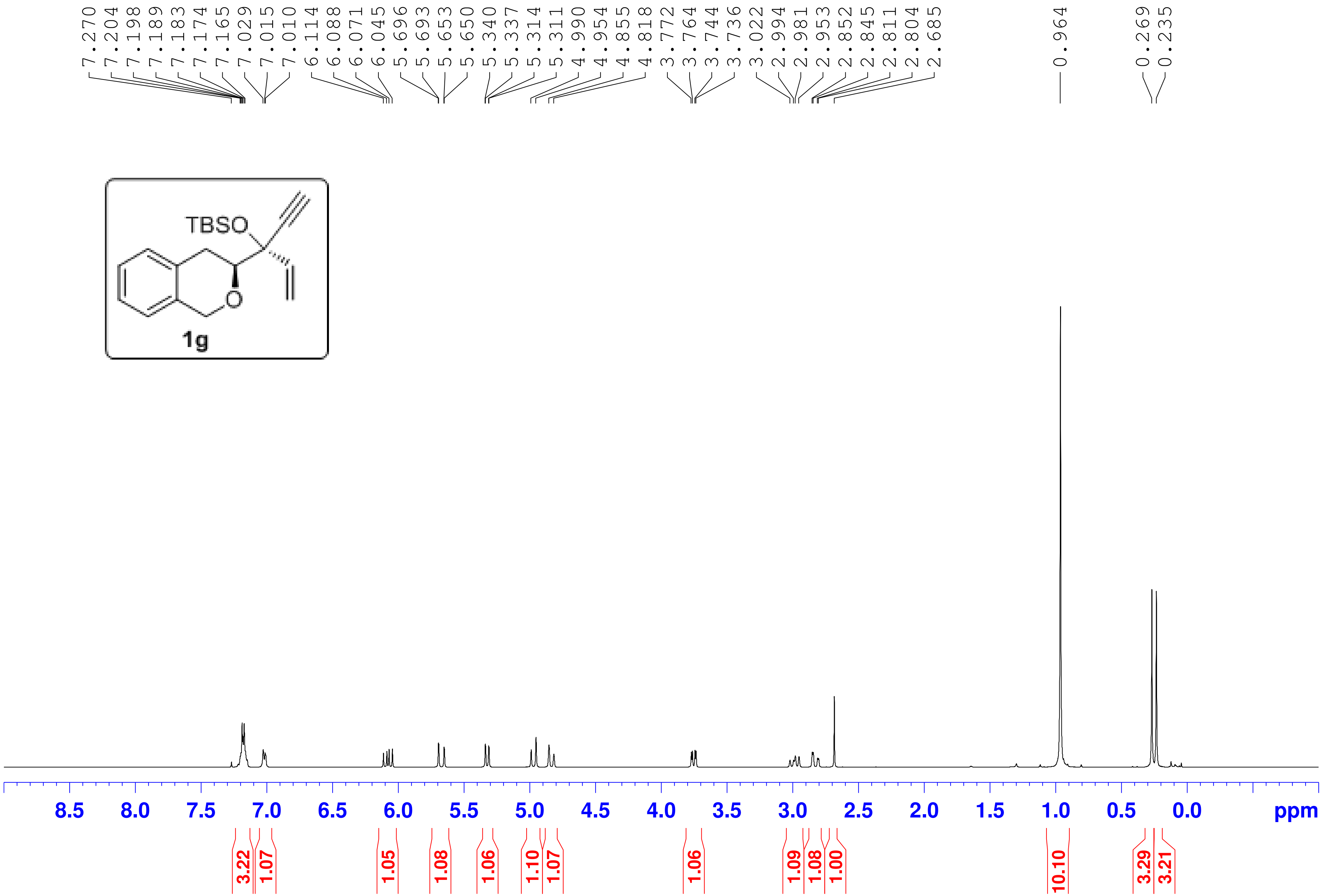

Supplementary Figure 52. ^{13}C **NMR Spectrum of 1g (100 MHz,** $CDCl_3$**)**

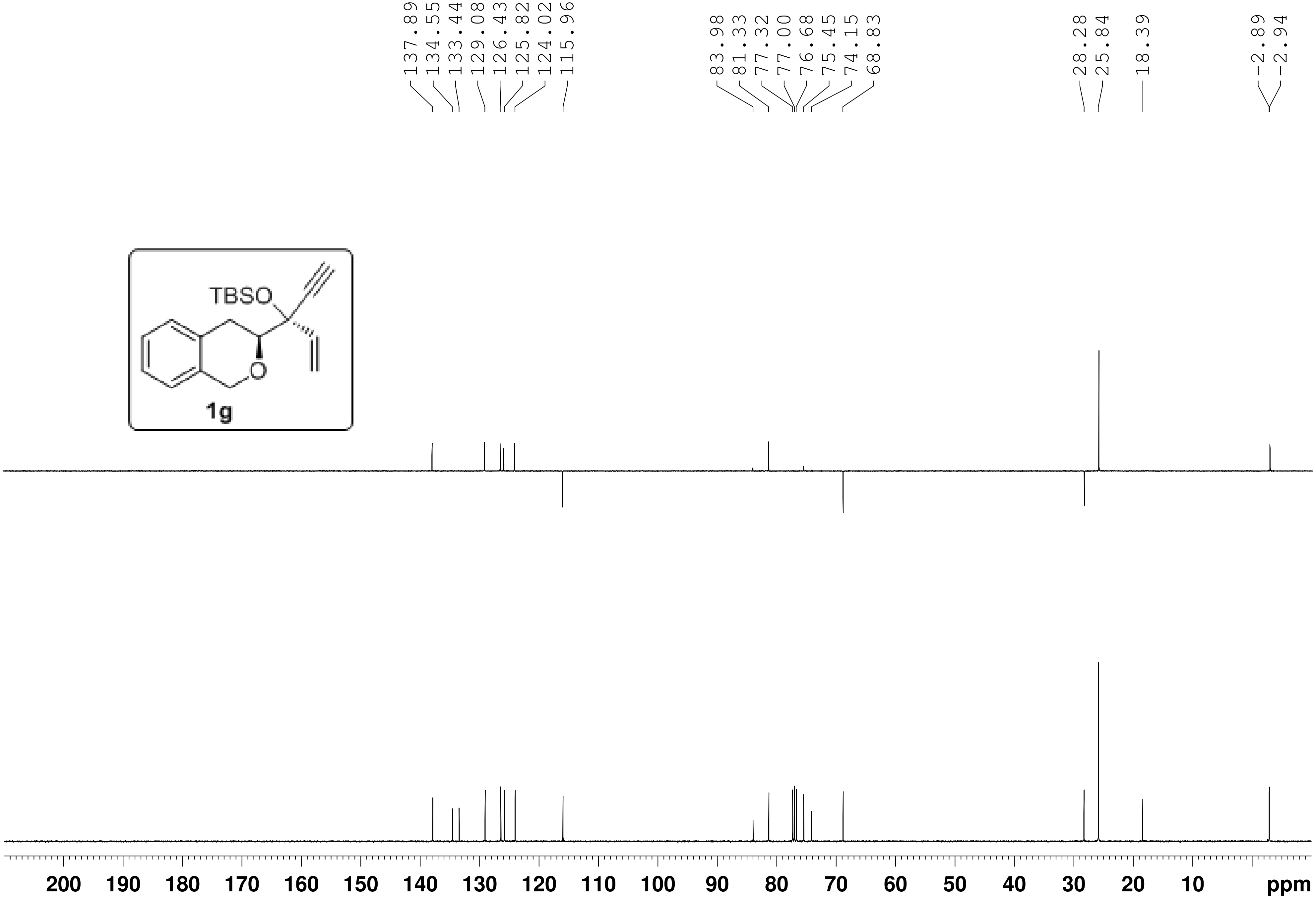

Supplementary Figure 53. ^{1}H NMR Spectrum of 2g (400 MHz, $CDCl_3$)

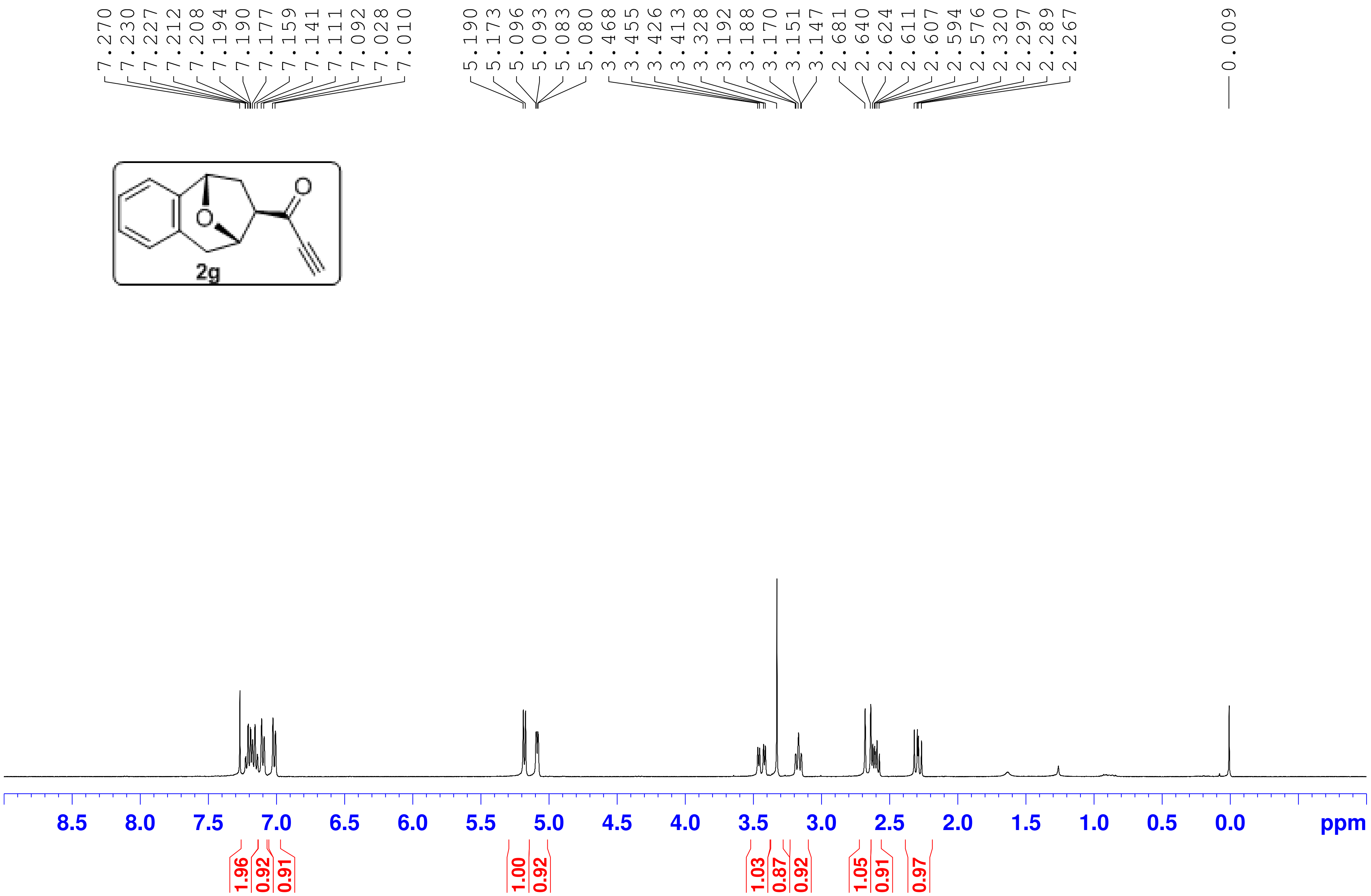

Supplementary Figure 54. ^{13}C NMR Spectrum of 2g (100 MHz, $CDCl_3$)

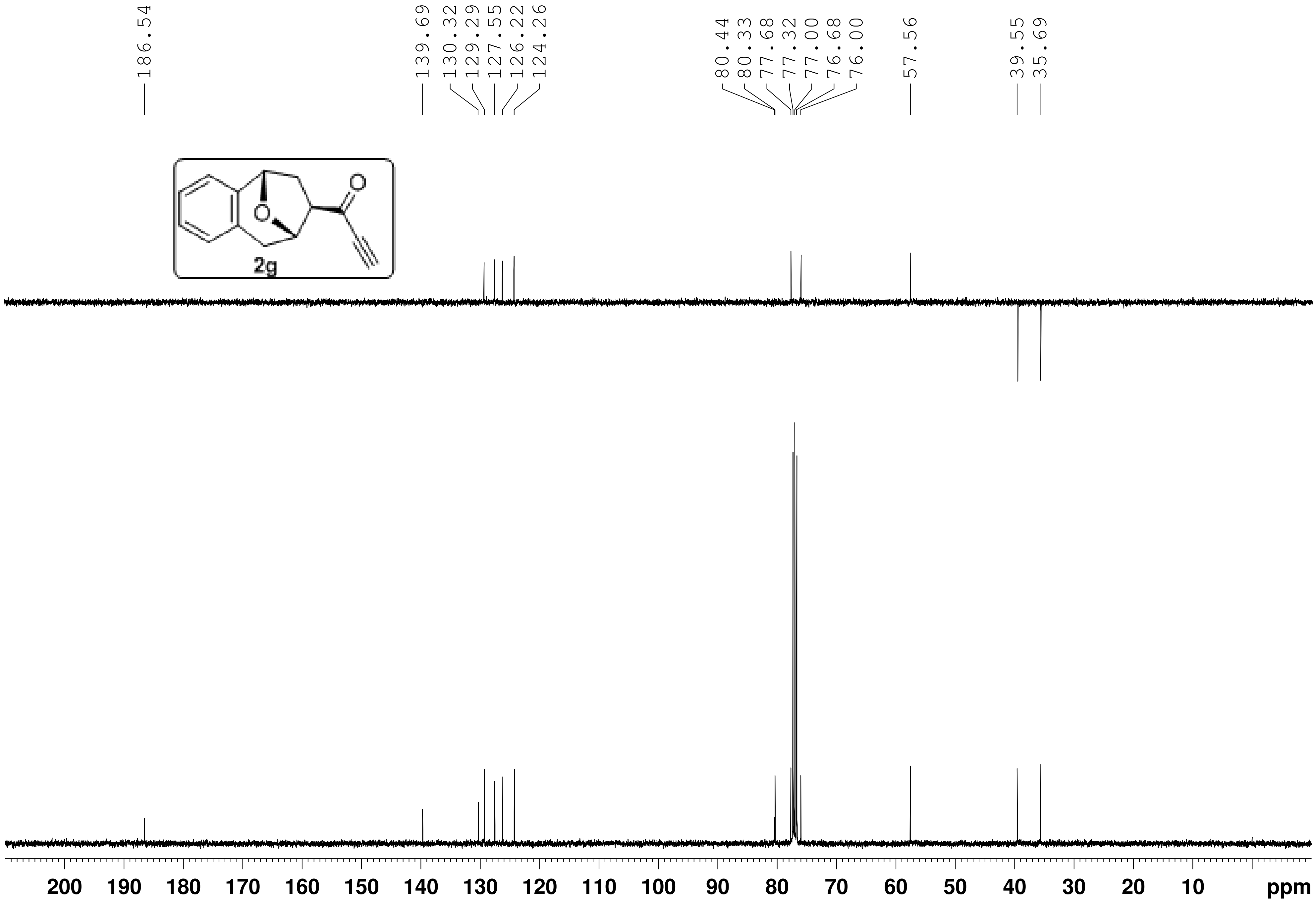

Supplementary Figure 55. ¹H NMR Spectrum of S16 (400 MHz, CDCl₃)

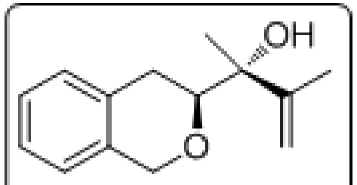

S16

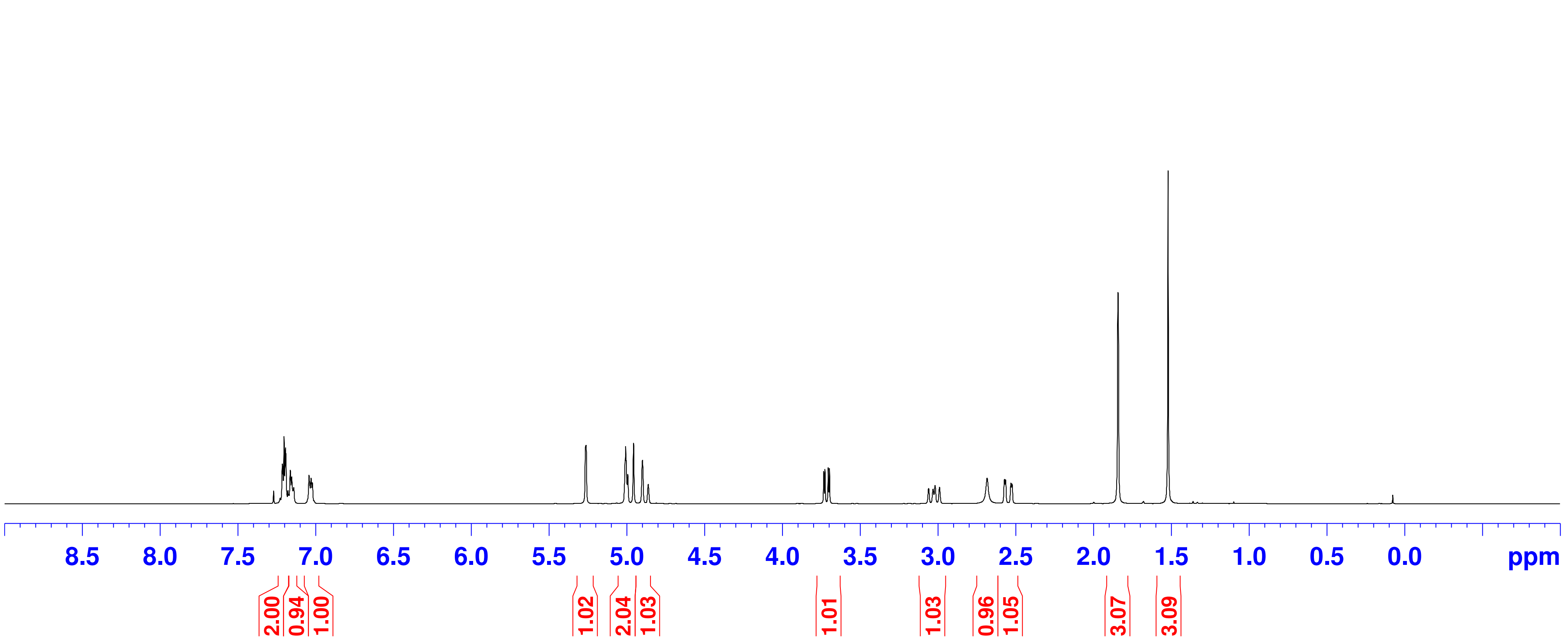

Supplementary Figure 56. ^{13}C NMR Spectrum of S16 (100 MHz, $CDCl_3$)

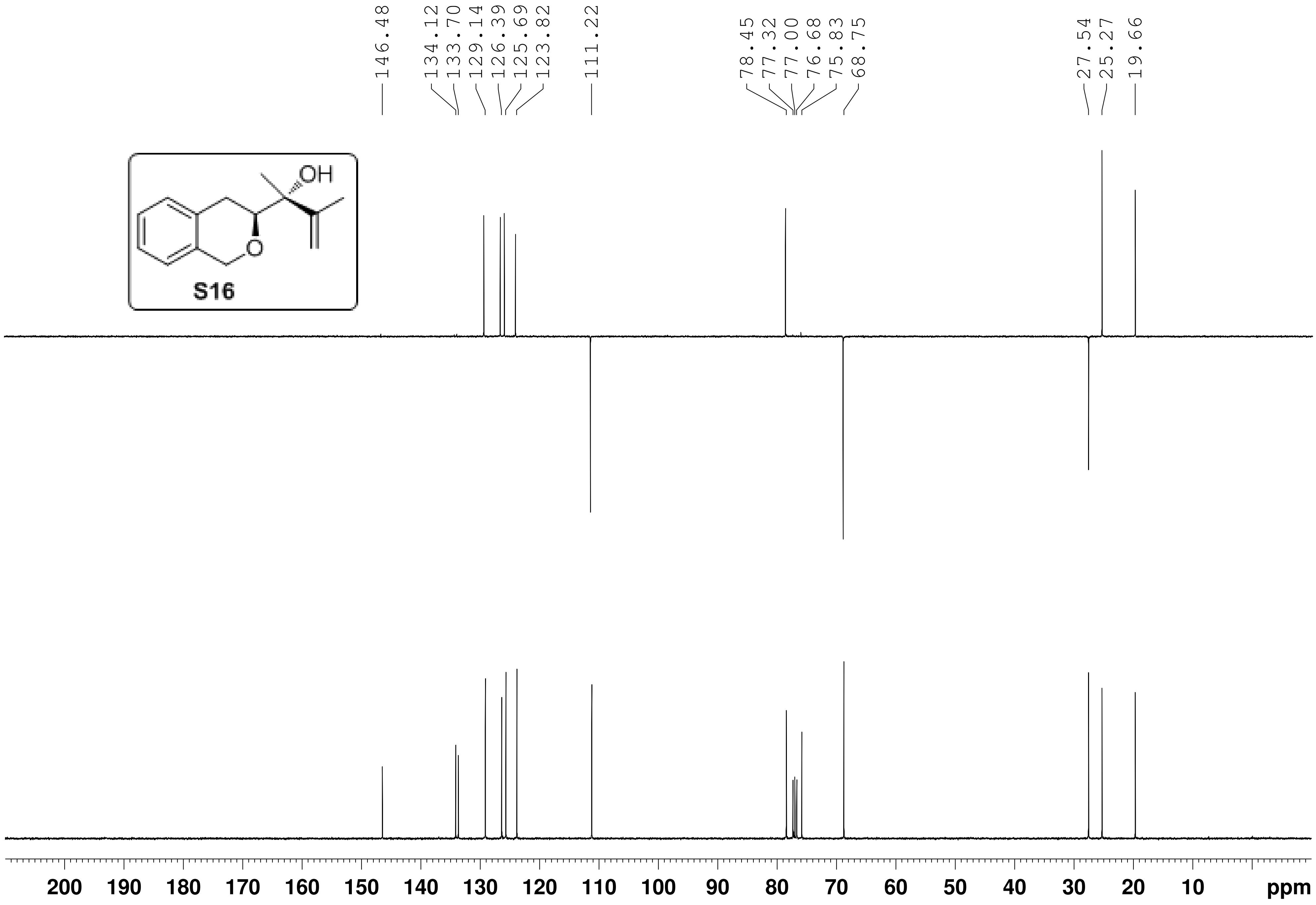

Supplementary Figure 57. ¹H NMR Spectrum of S16' (400 MHz, $CDCl_3$)

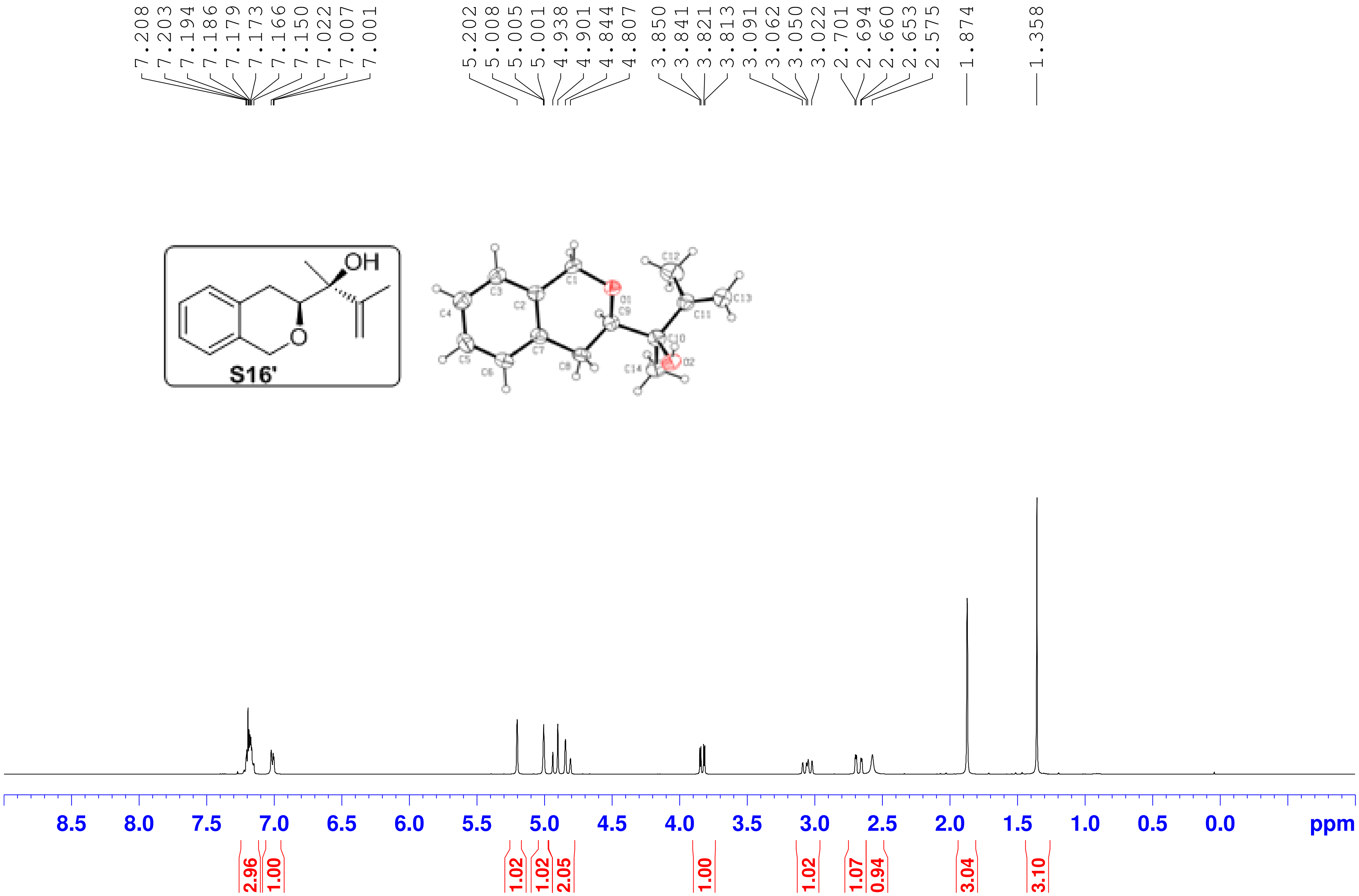

Supplementary Figure 58. ^{13}C NMR Spectrum of S16' (100 MHz, $CDCl_3$)

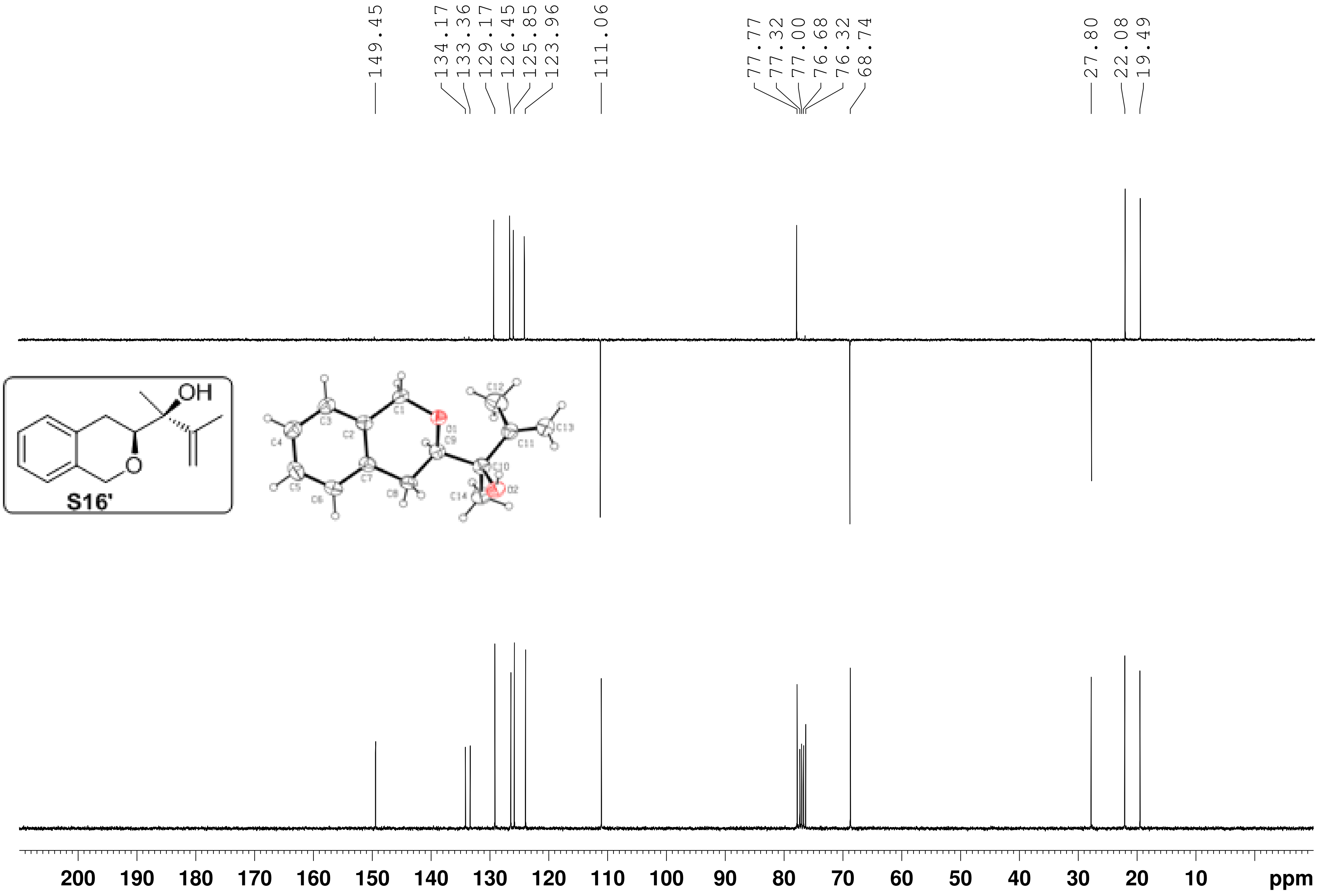

Supplementary Figure 59. ^{1}H NMR Spectrum of 1h (400 MHz, $CDCl_3$)

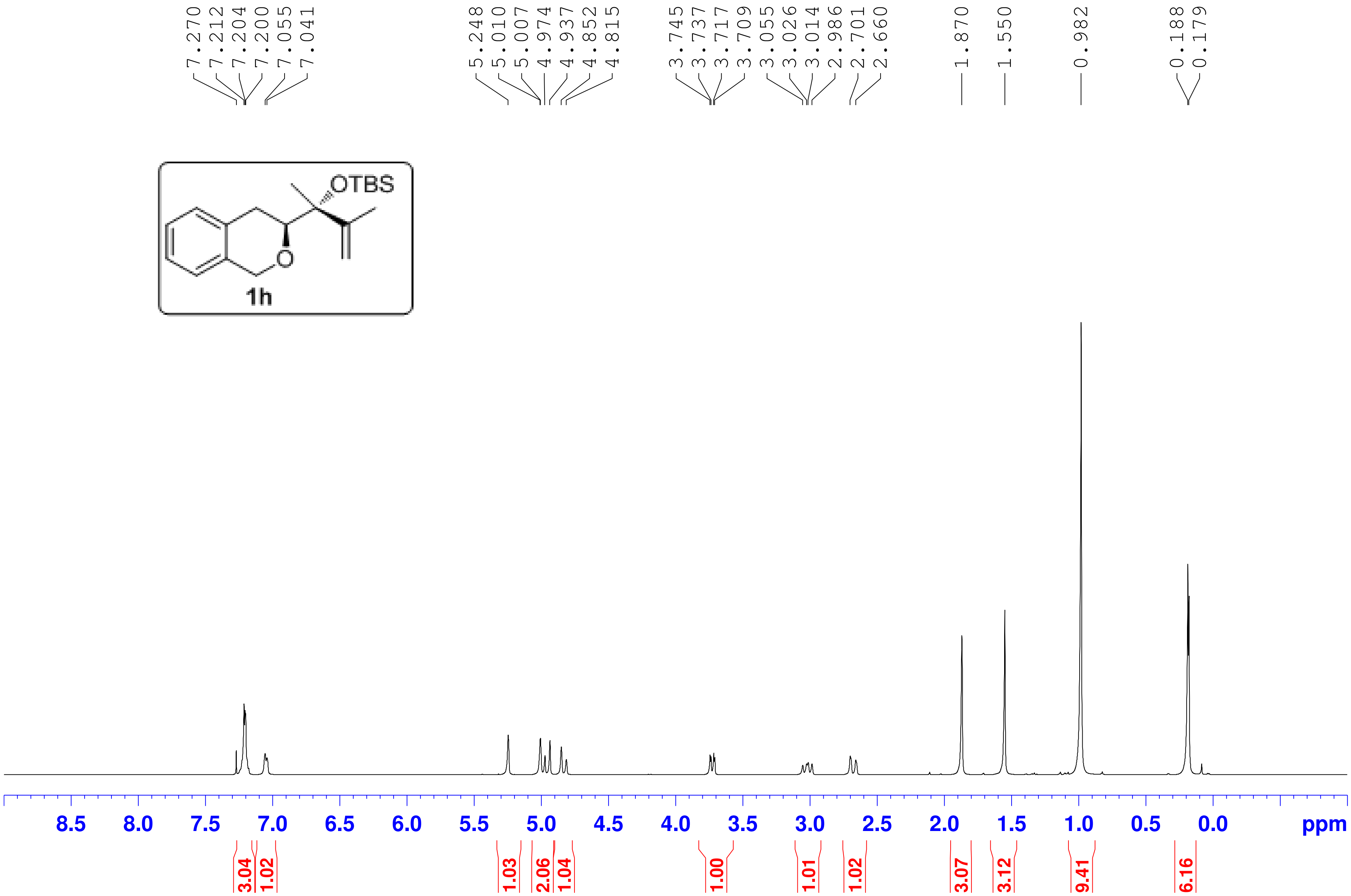

Supplementary Figure 60. ^{13}C NMR Spectrum of 1h (100 MHz, $CDCl_3$)

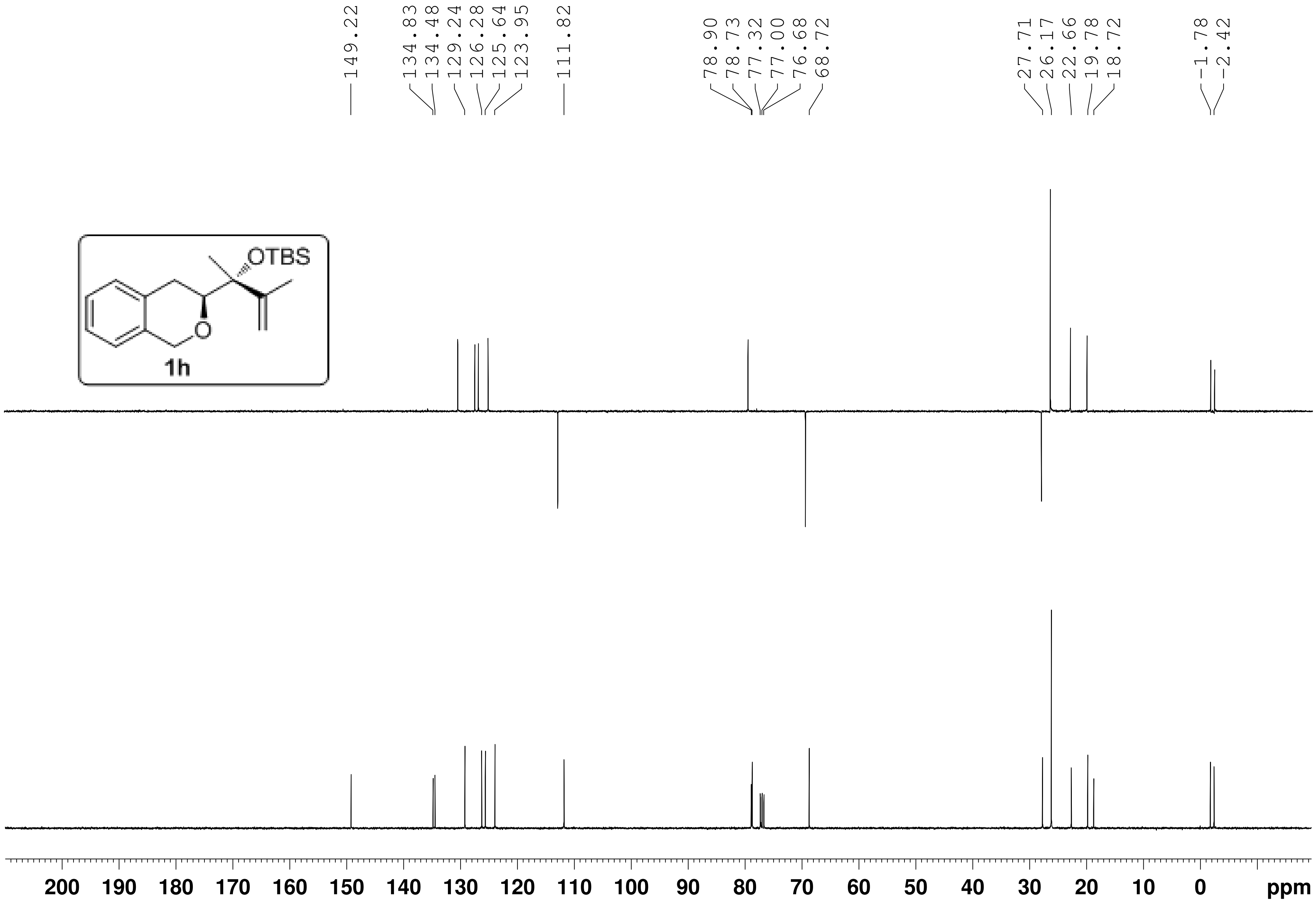

Supplementary Figure 61. ^{1}H NMR Spectrum of 1h' (400 MHz, $CDCl_3$)

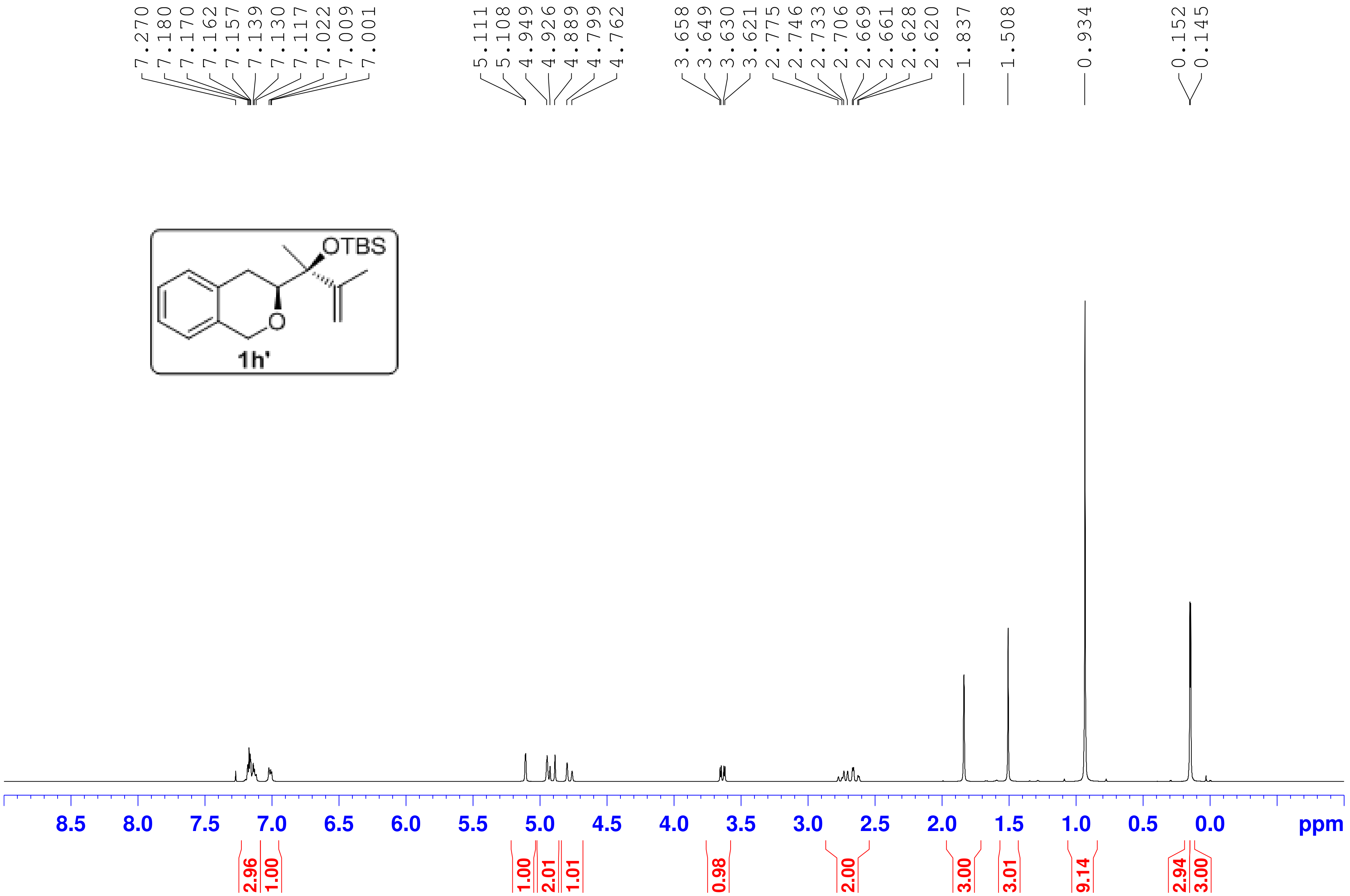

Supplementary Figure 62. ^{13}C NMR Spectrum of 1h' (100 MHz, $CDCl_3$)

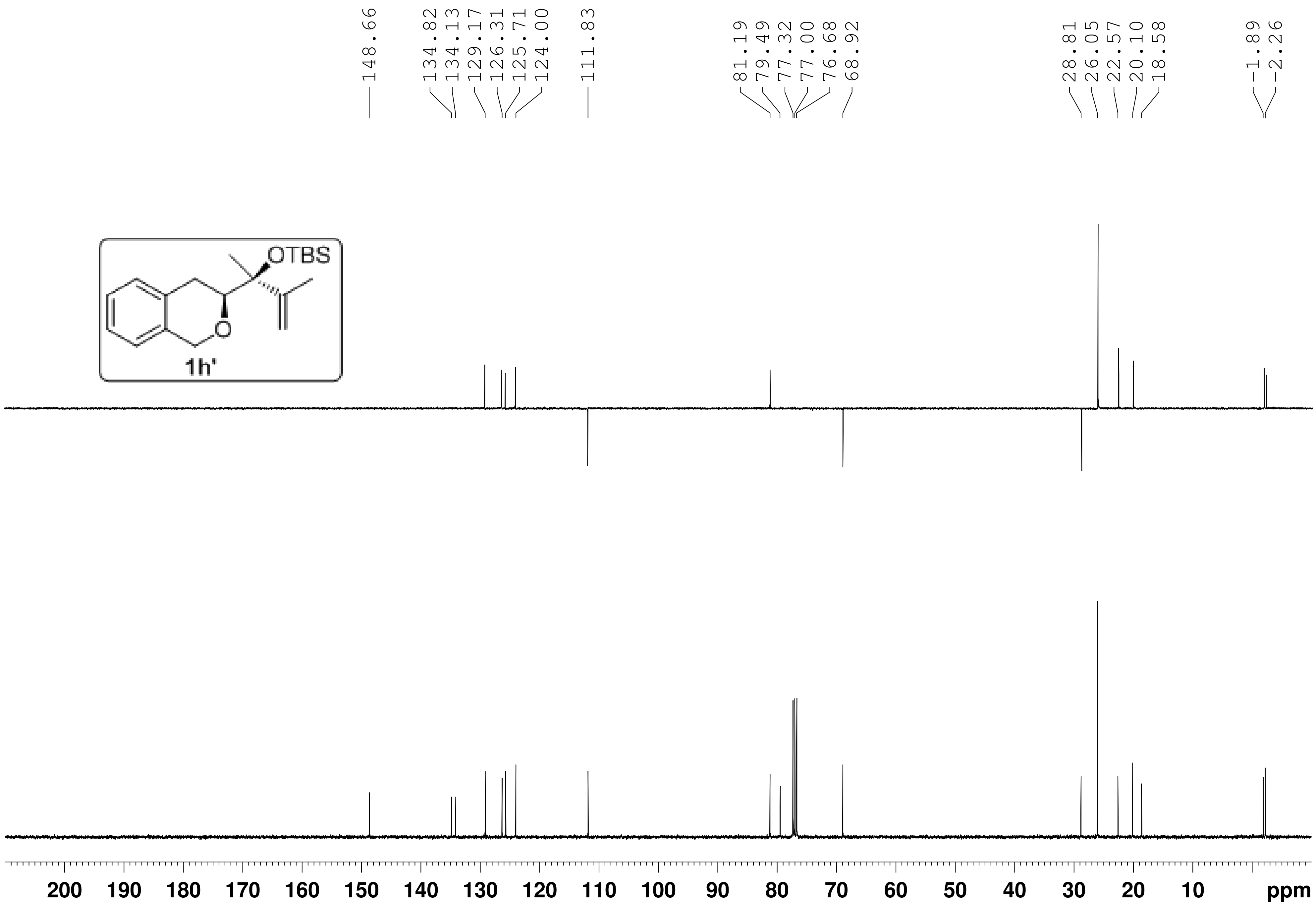

Supplementary Figure 63. ^{1}H **NMR Spectrum of 2h (400 MHz, CDCl**$_3$**)**

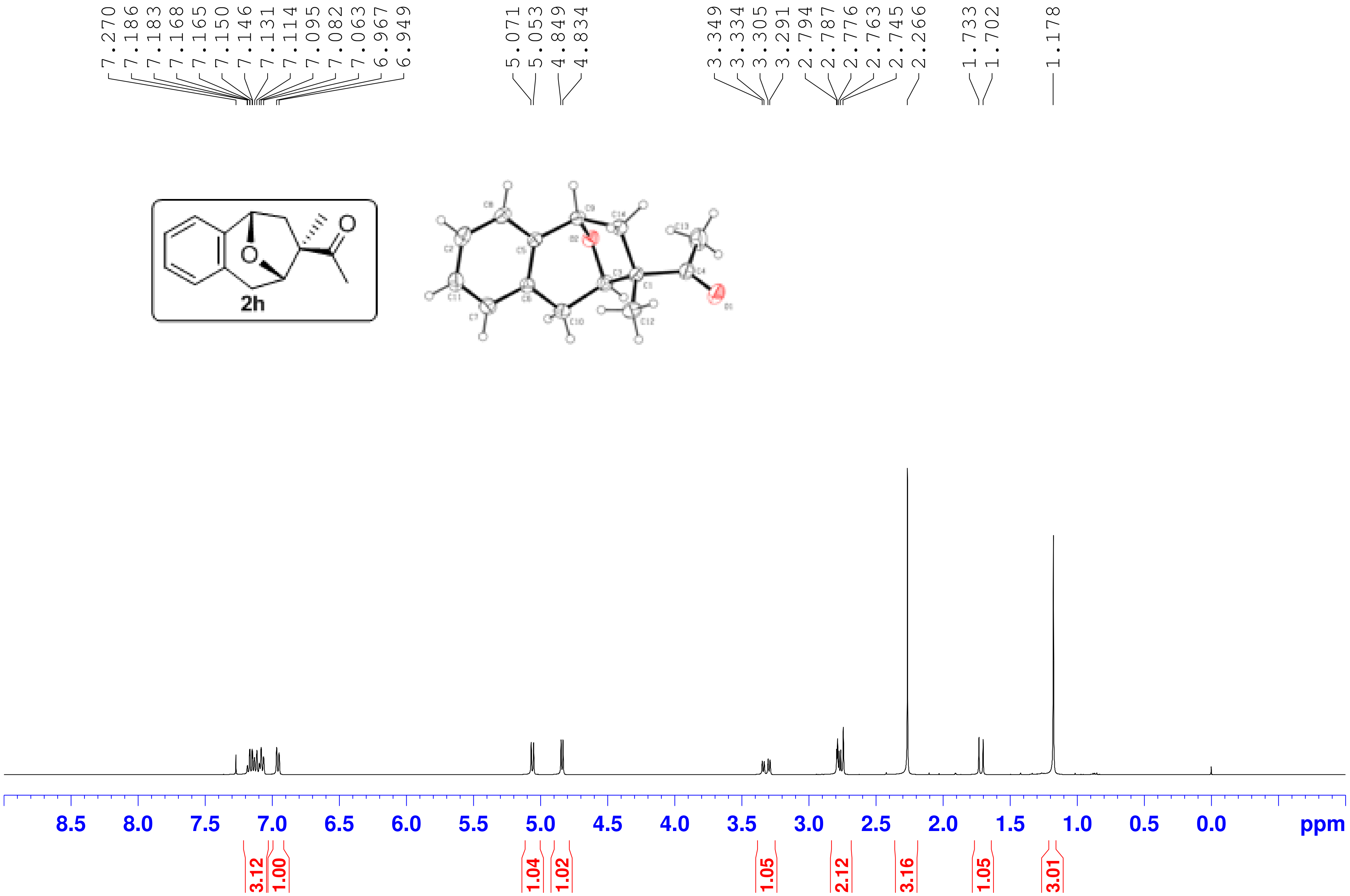

Supplementary Figure 64. ^{13}C NMR Spectrum of 2h (100 MHz, $CDCl_3$)

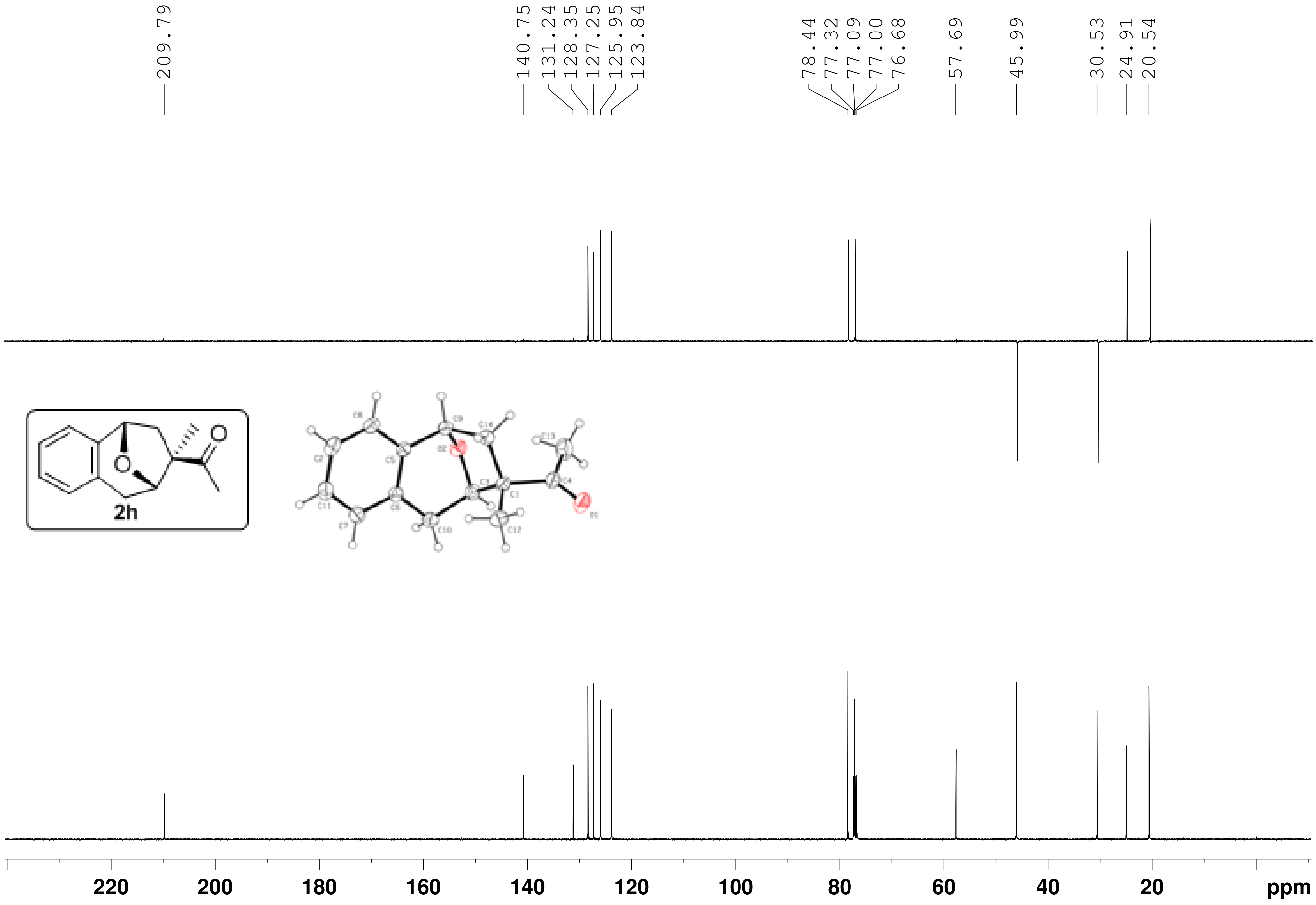

Supplementary Figure 65. ^{1}H NMR Spectrum of S18 (400 MHz, $CDCl_3$)

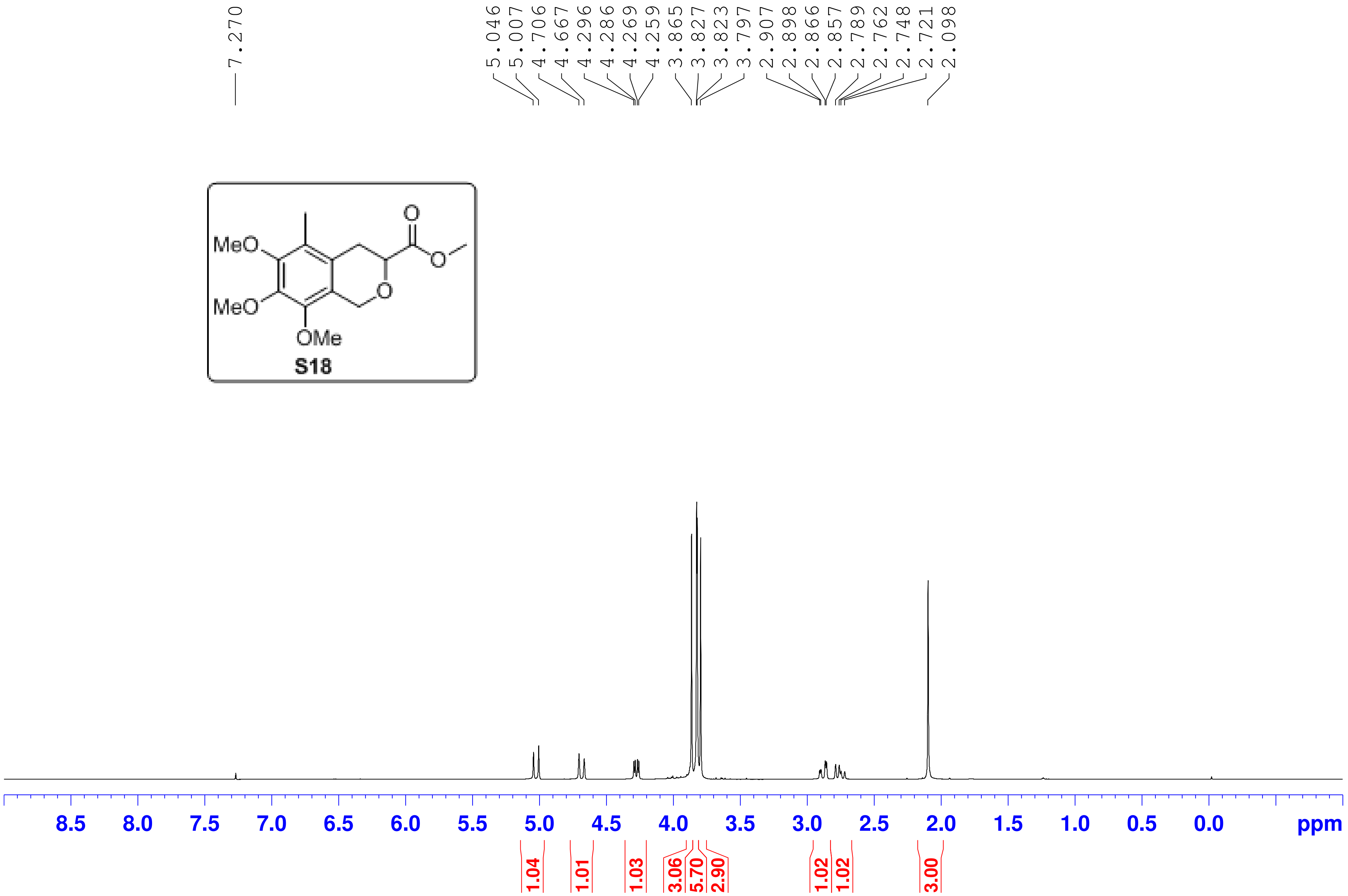

Supplementary Figure 66. ^{13}C NMR Spectrum of S18 (100 MHz, $CDCl_3$)

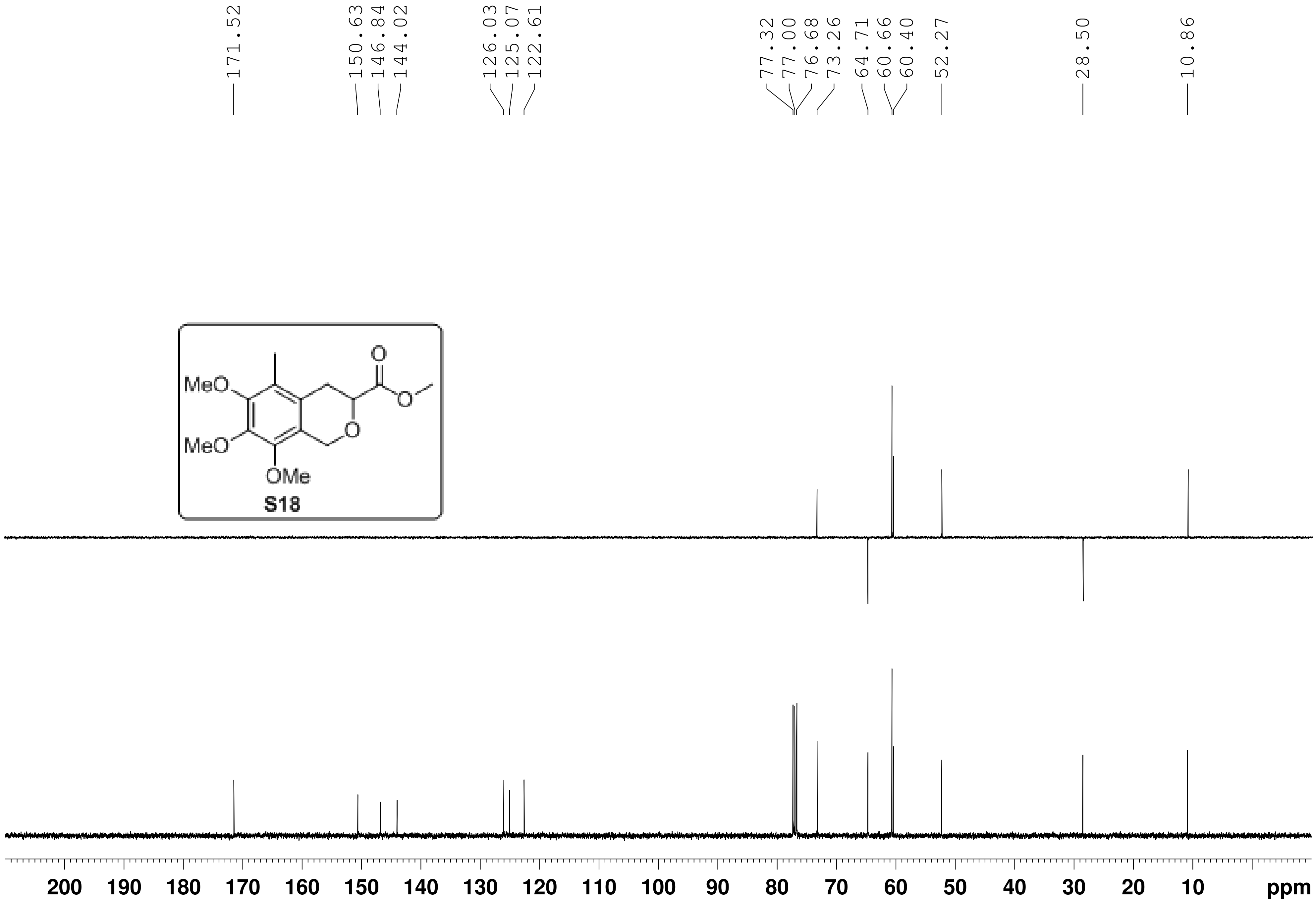

Supplementary Figure 67. ¹H NMR Spectrum of S19 (400 MHz, $CDCl_3$)

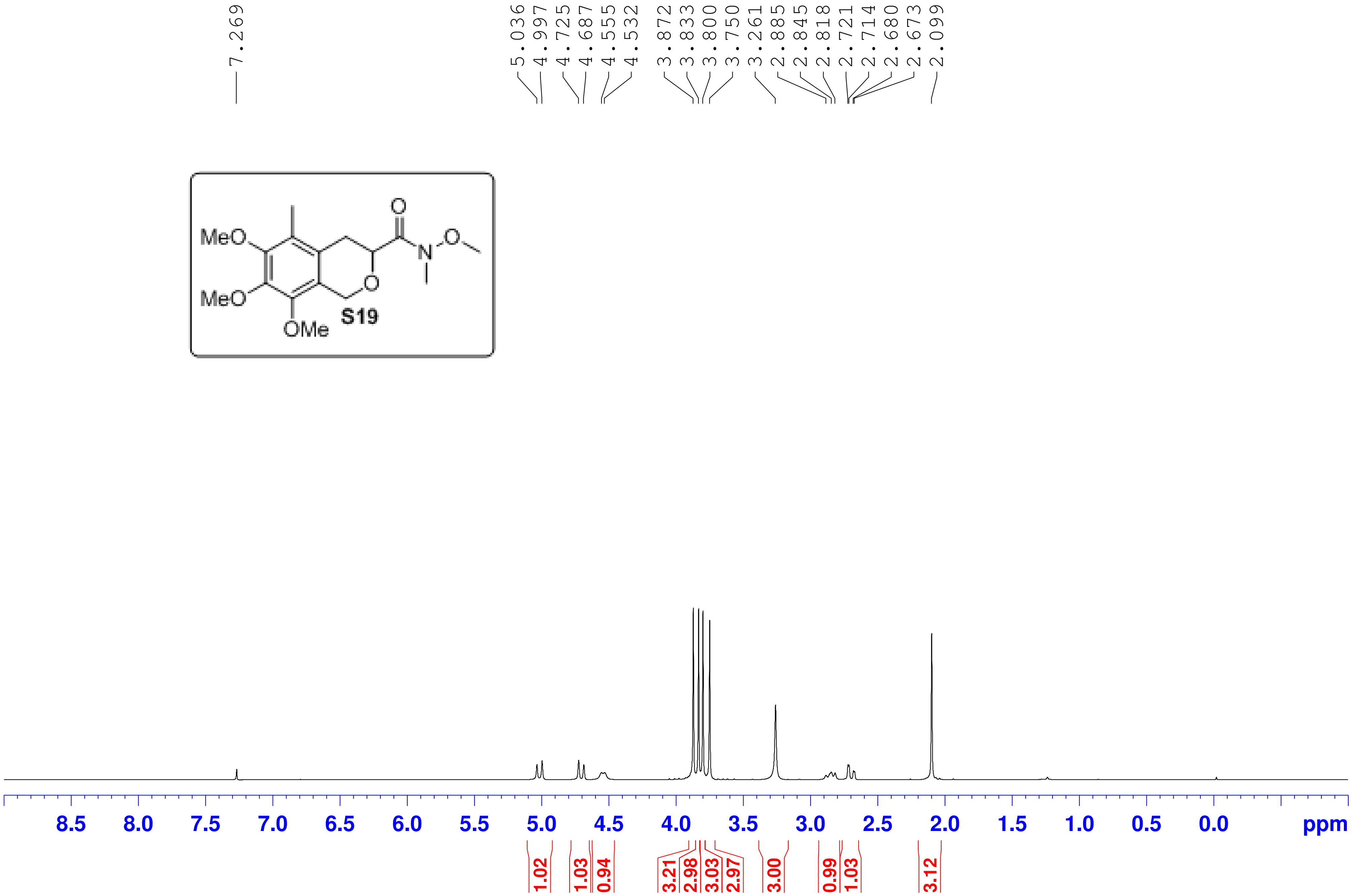

Supplementary Figure 68. ^{13}C NMR Spectrum of S19 (100 MHz, $CDCl_3$)

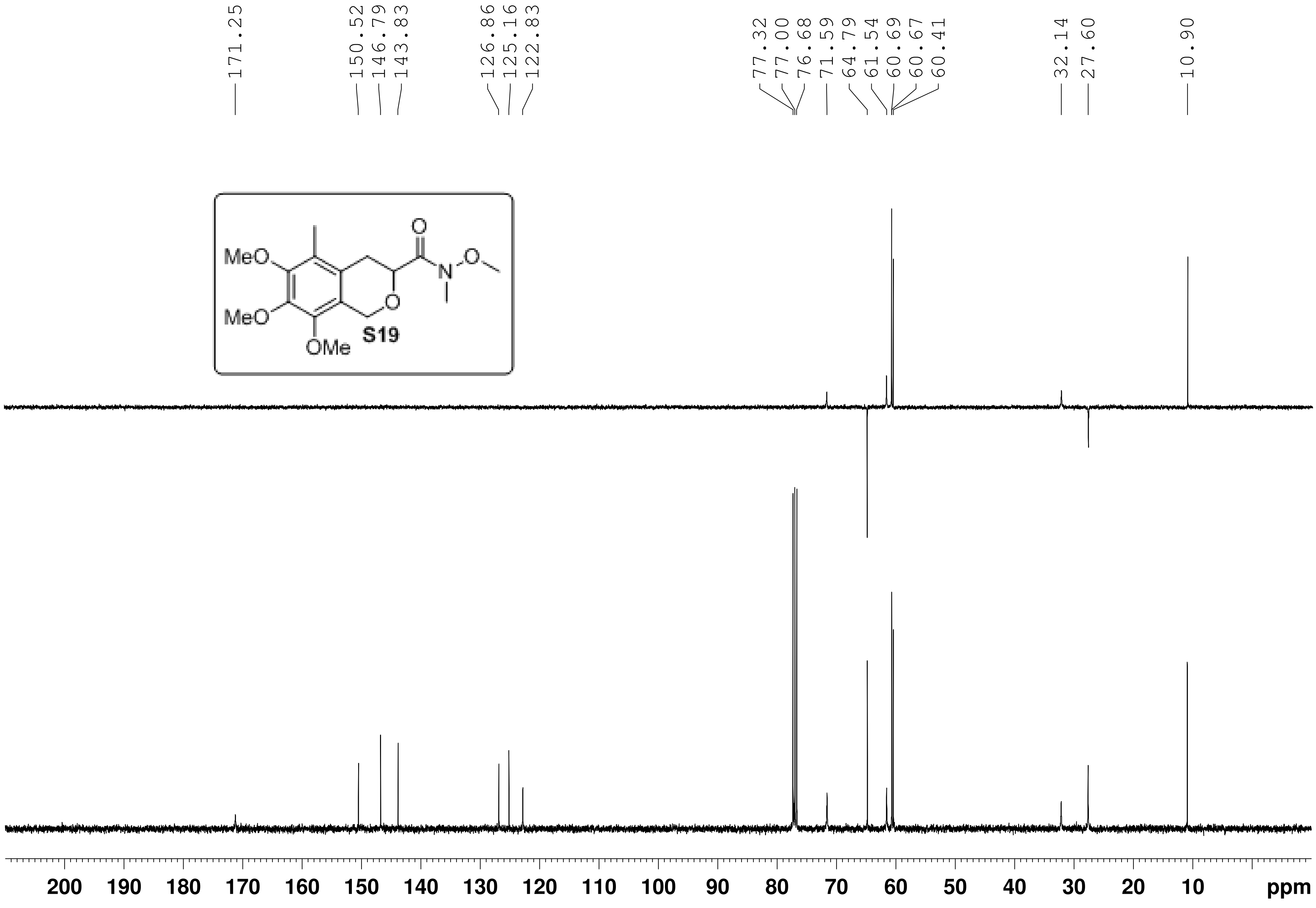

Supplementary Figure 69. ^{1}H NMR Spectrum of S20 (400 MHz, $CDCl_3$)

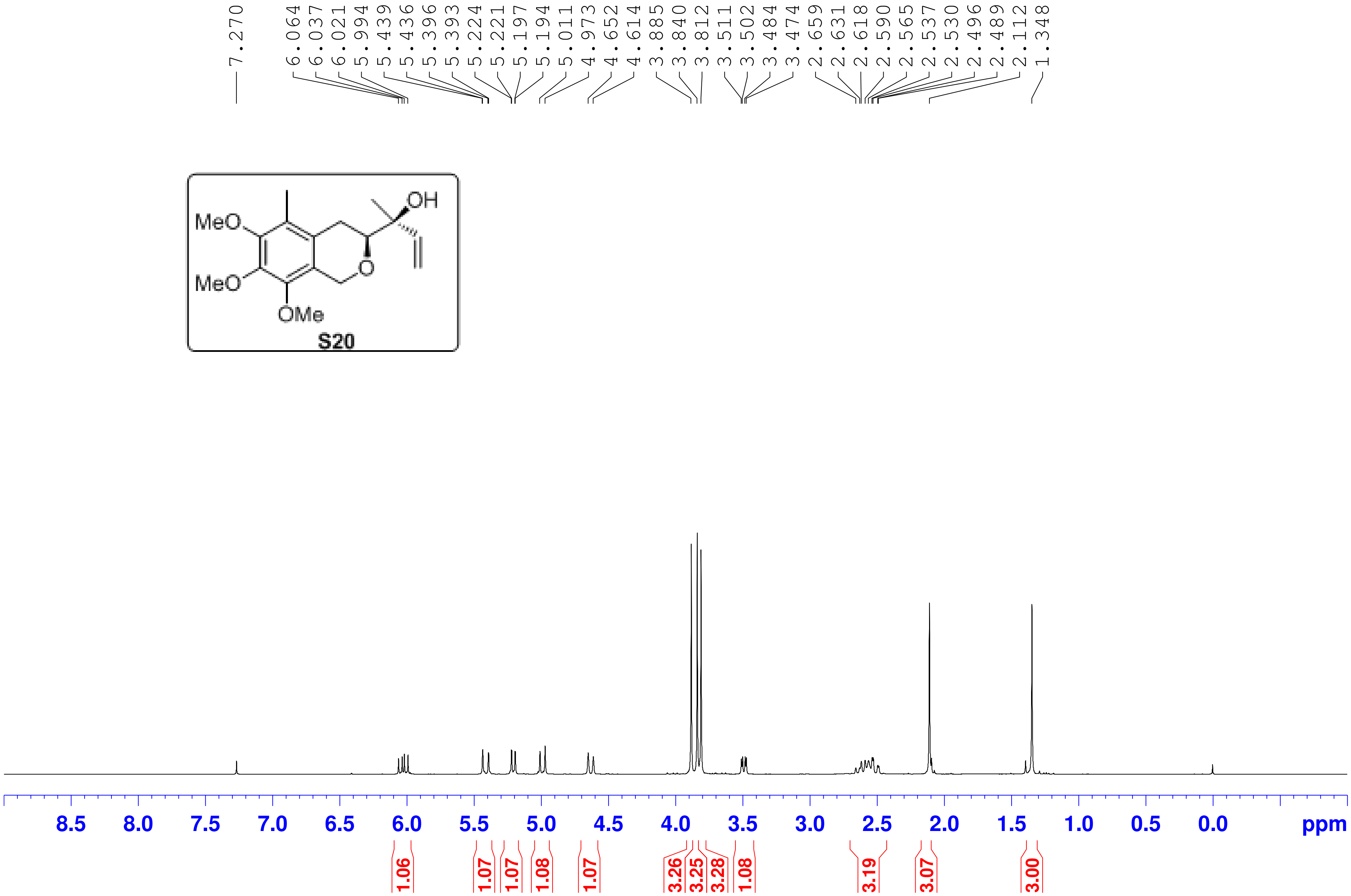

Supplementary Figure 70. ^{13}C NMR Spectrum of S20 (100 MHz, $CDCl_3$)

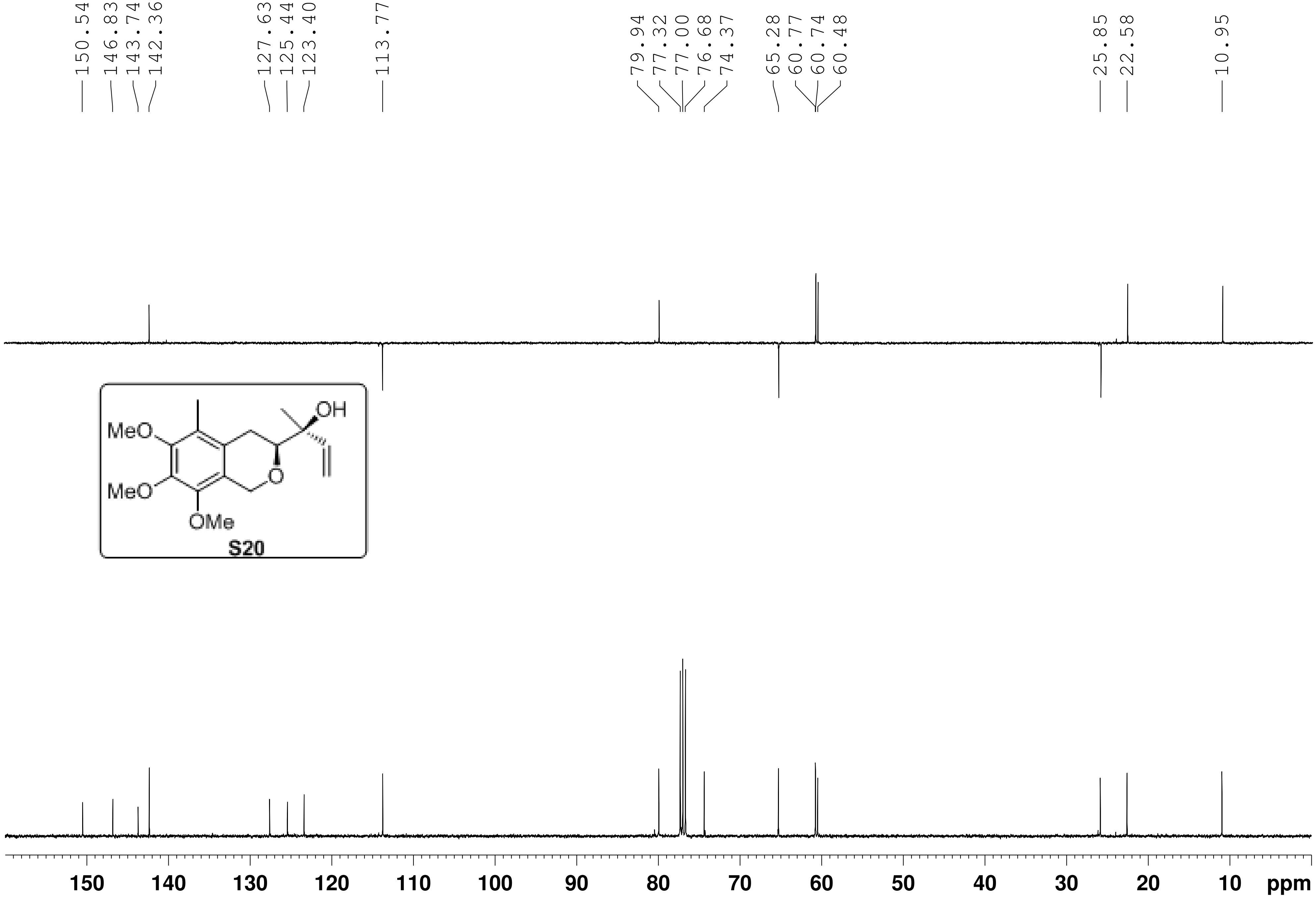

Supplementary Figure 71. ^{1}H NMR Spectrum of 1i (400 MHz, $CDCl_3$)

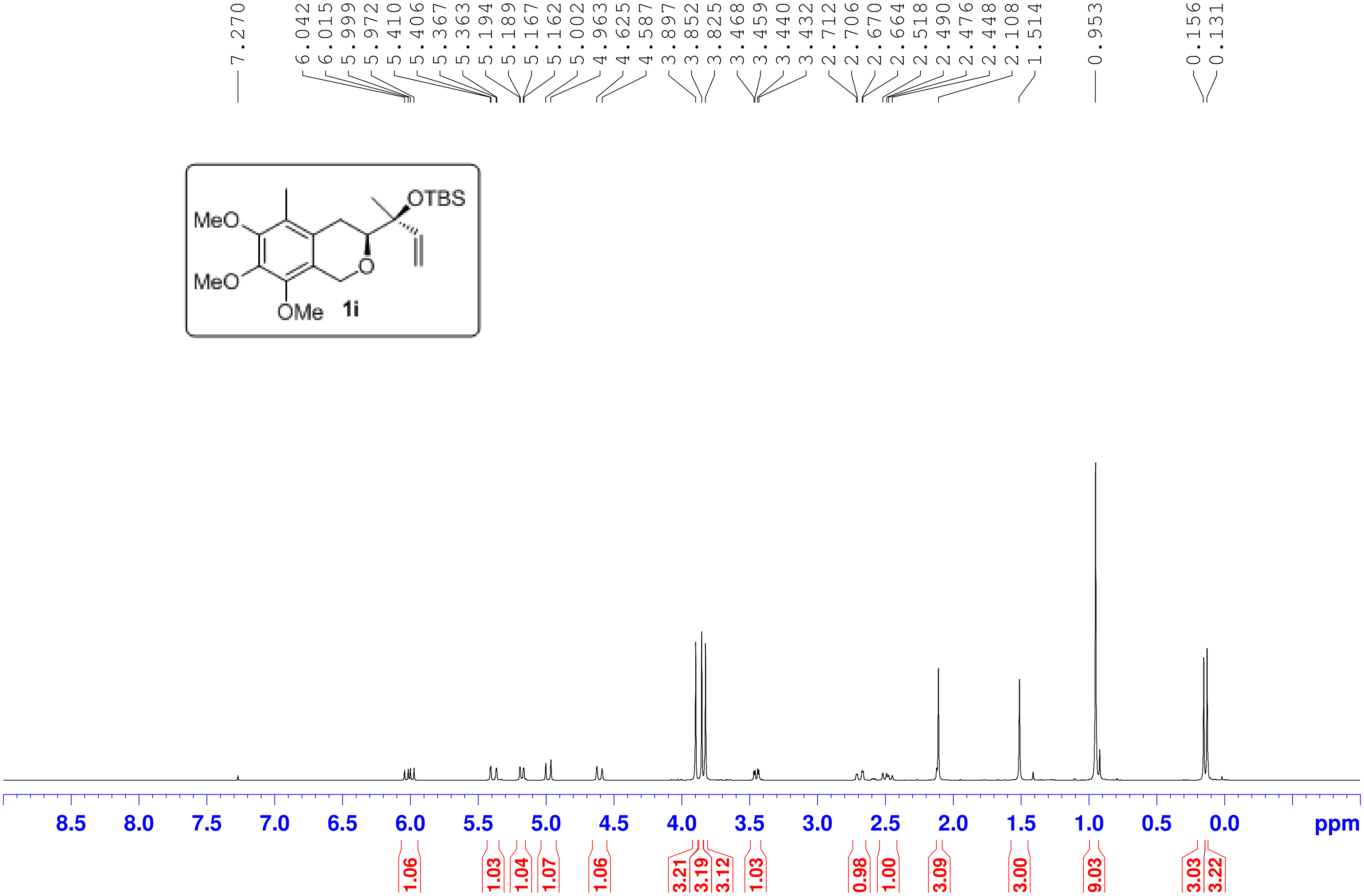

Supplementary Figure 72. ^{13}C NMR Spectrum of 1i (100 MHz, $CDCl_3$)

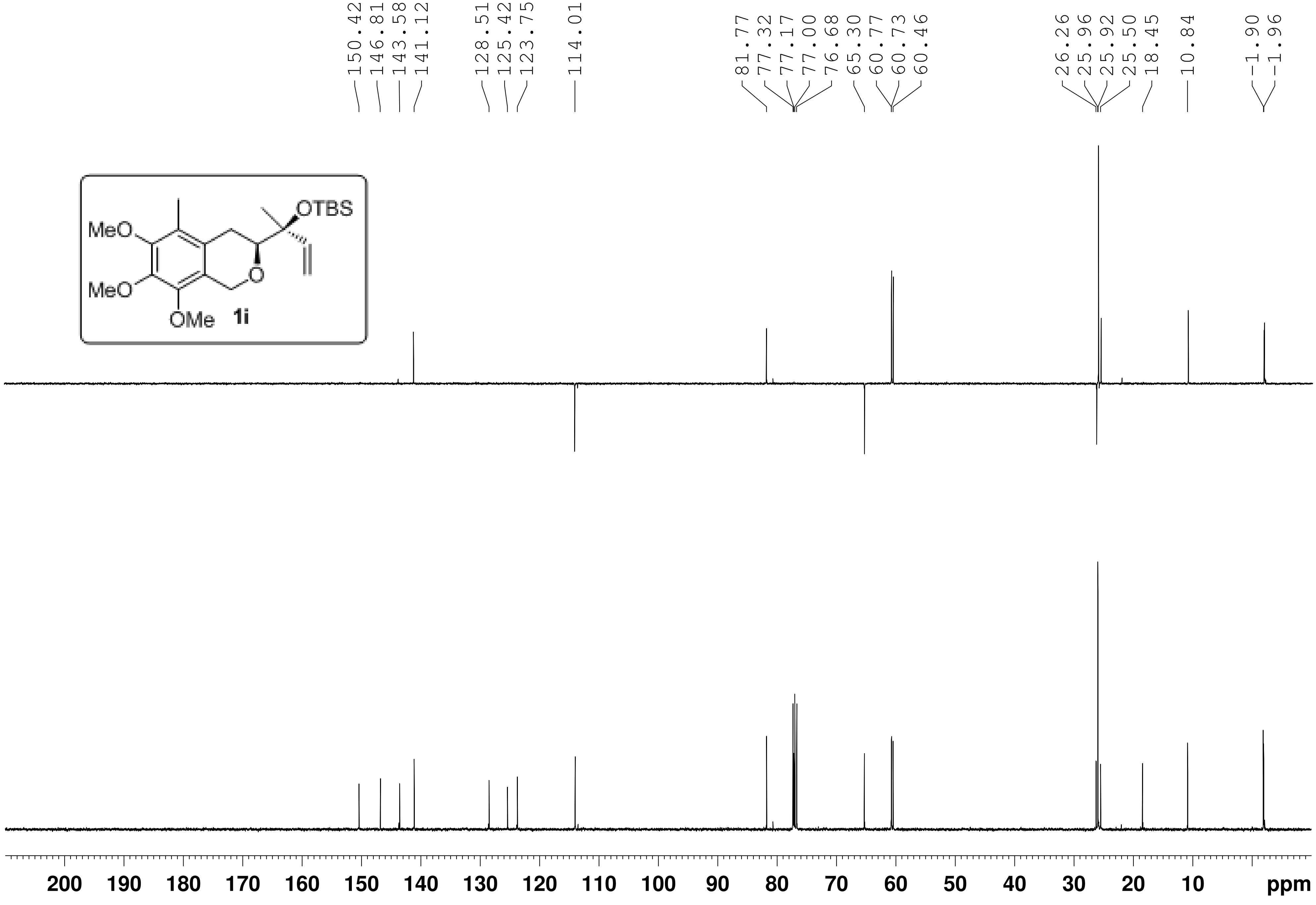

Supplementary Figure 73. ^{1}H NMR Spectrum of 2i (400 MHz, $CDCl_3$)

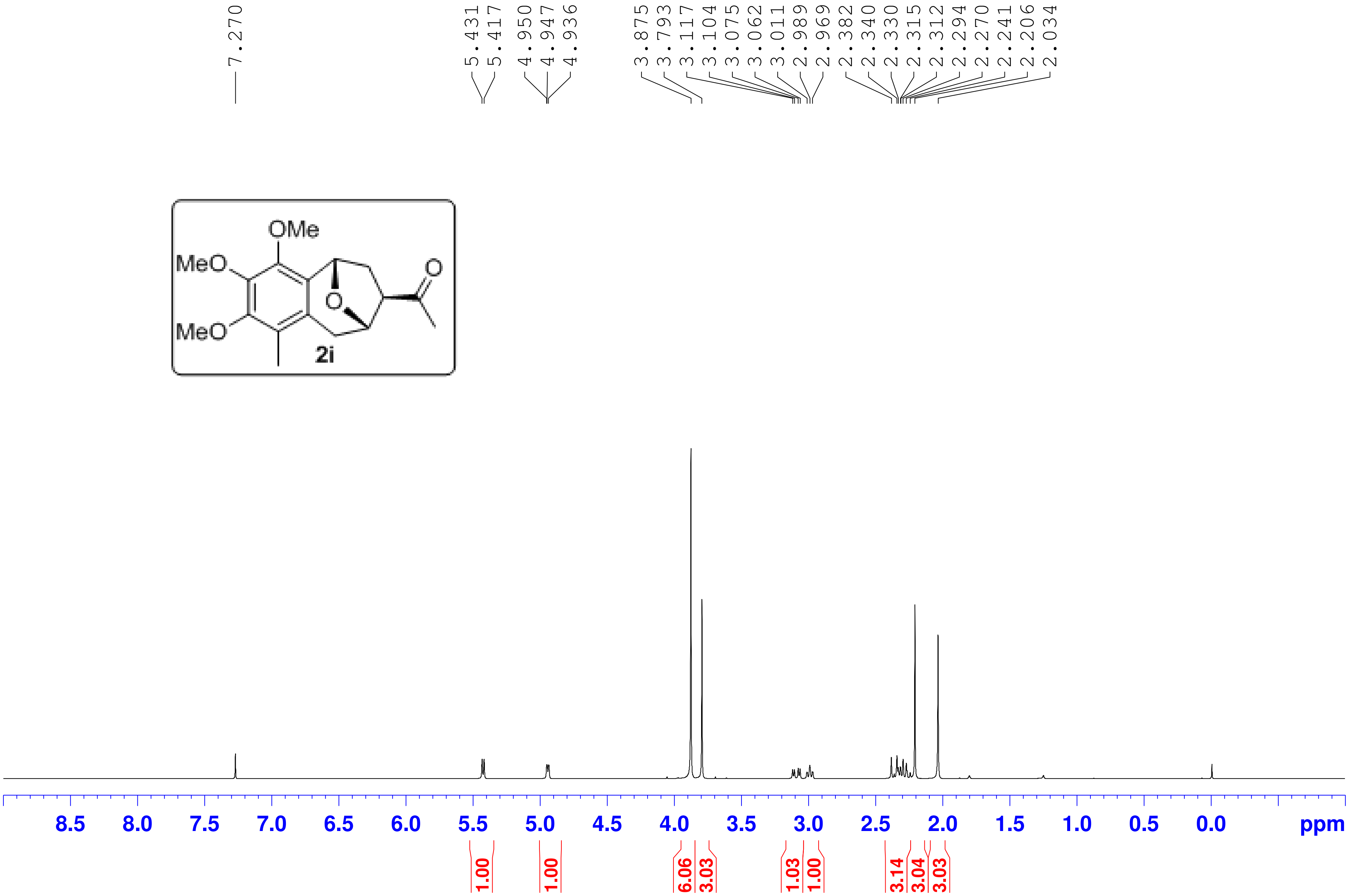

Supplementary Figure 74. ^{13}C NMR Spectrum of 2i (100 MHz, $CDCl_3$)

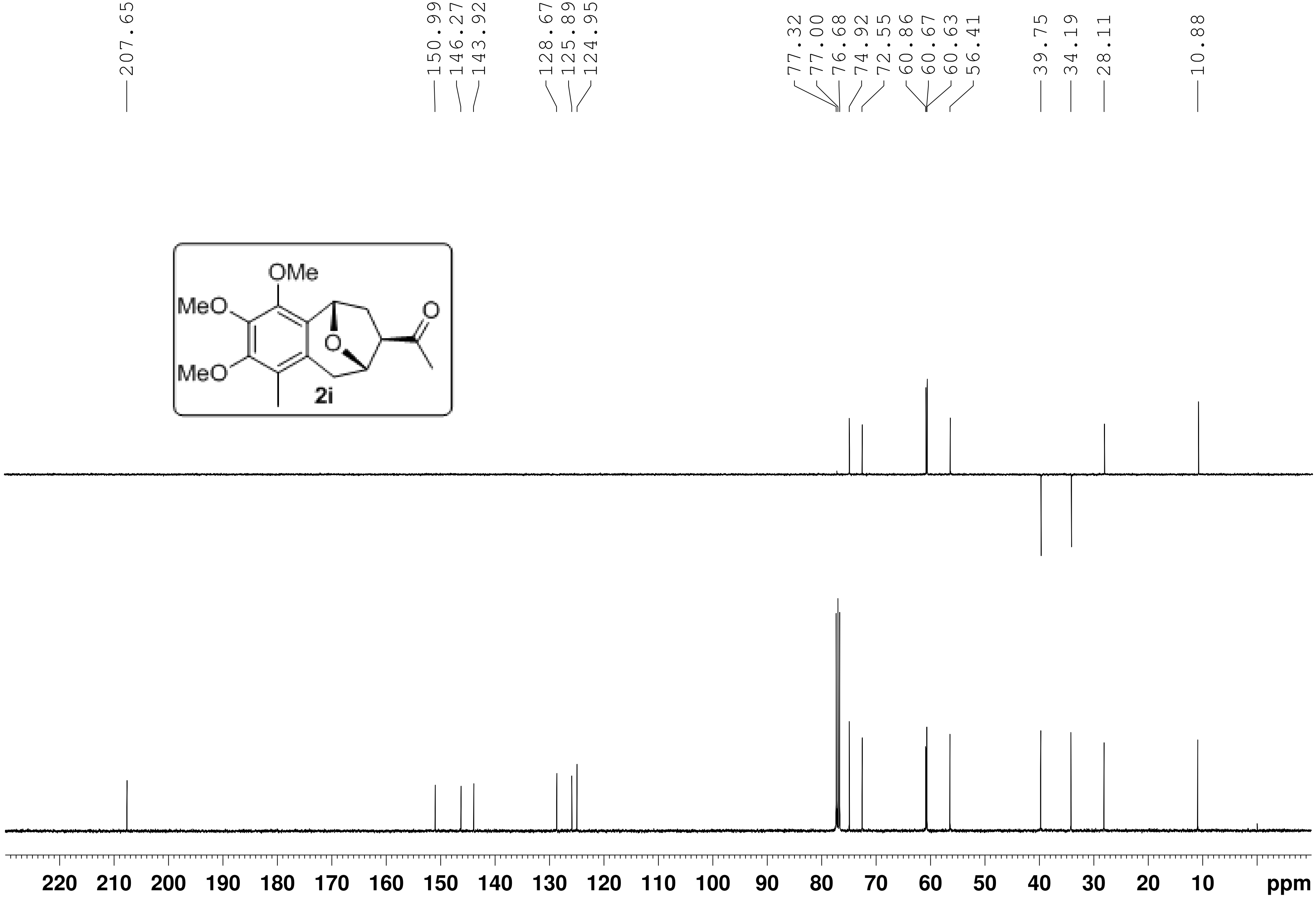

Supplementary Figure 75. ^{1}H NMR Spectrum of S21 (400 MHz, $CDCl_3$)

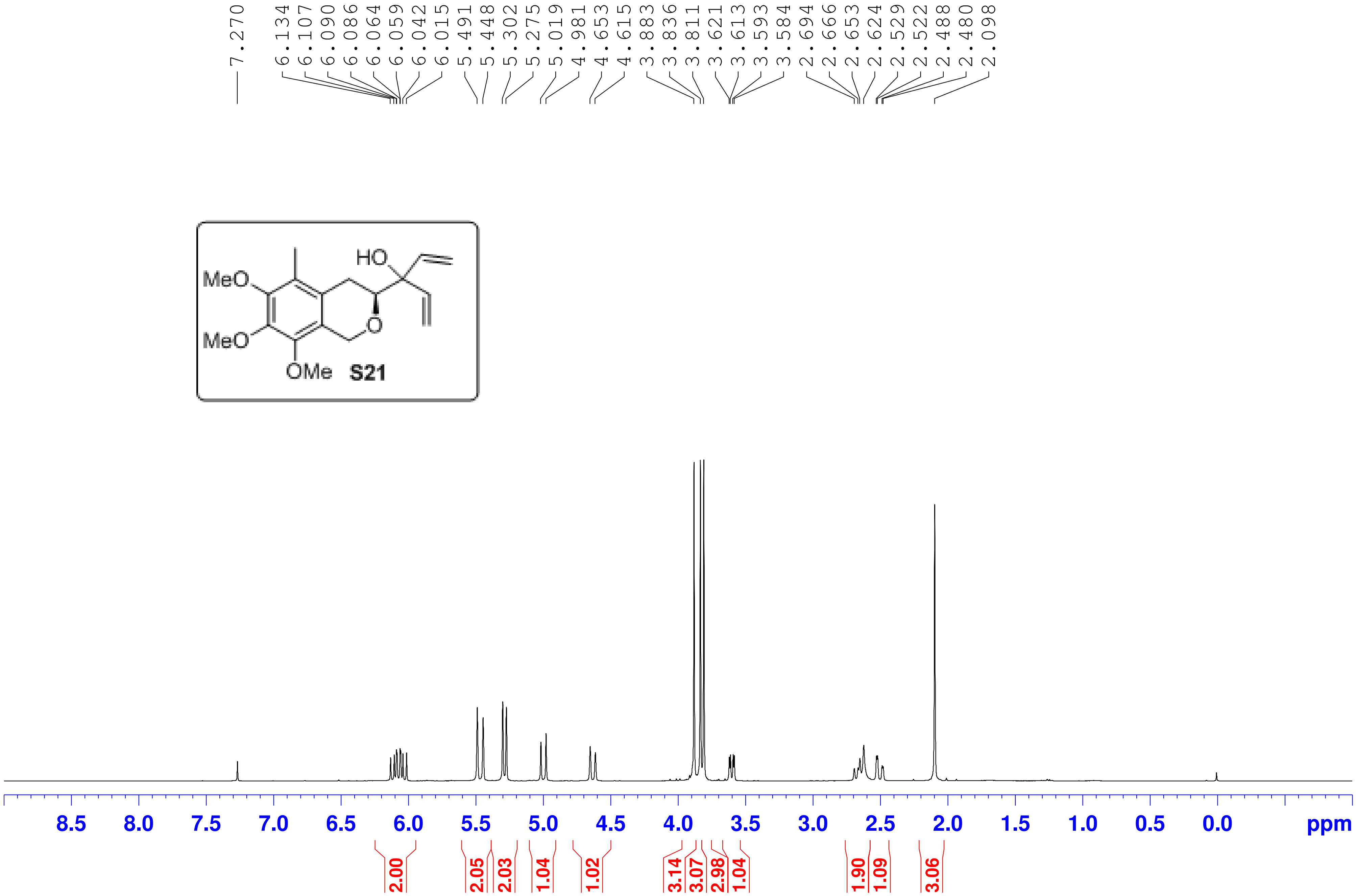

Supplementary Figure 76. ^{13}C NMR Spectrum of S21 (100 MHz, $CDCl_3$)

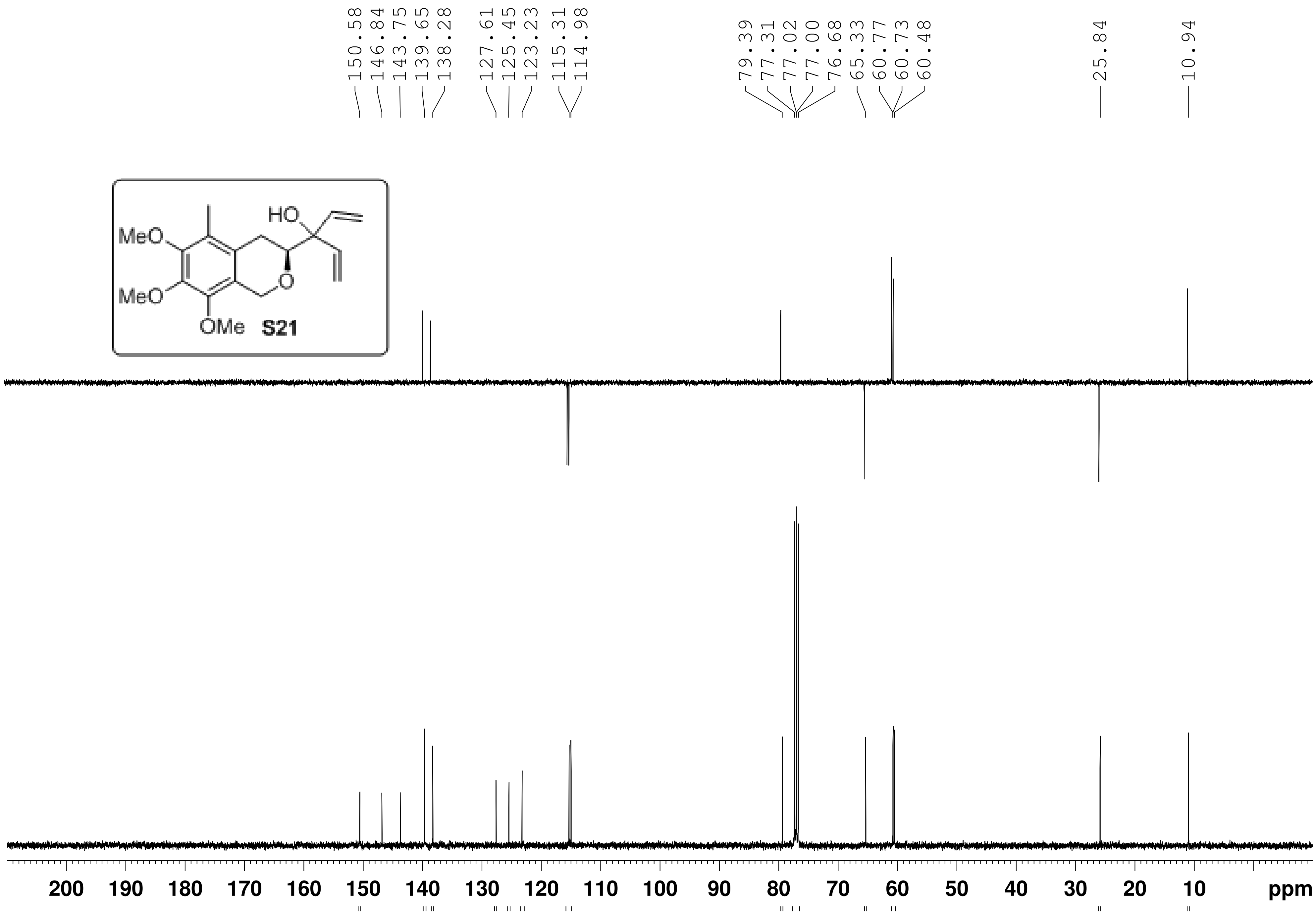

Supplementary Figure 77. ^{1}H NMR Spectrum of 1j (400 MHz, $CDCl_3$)

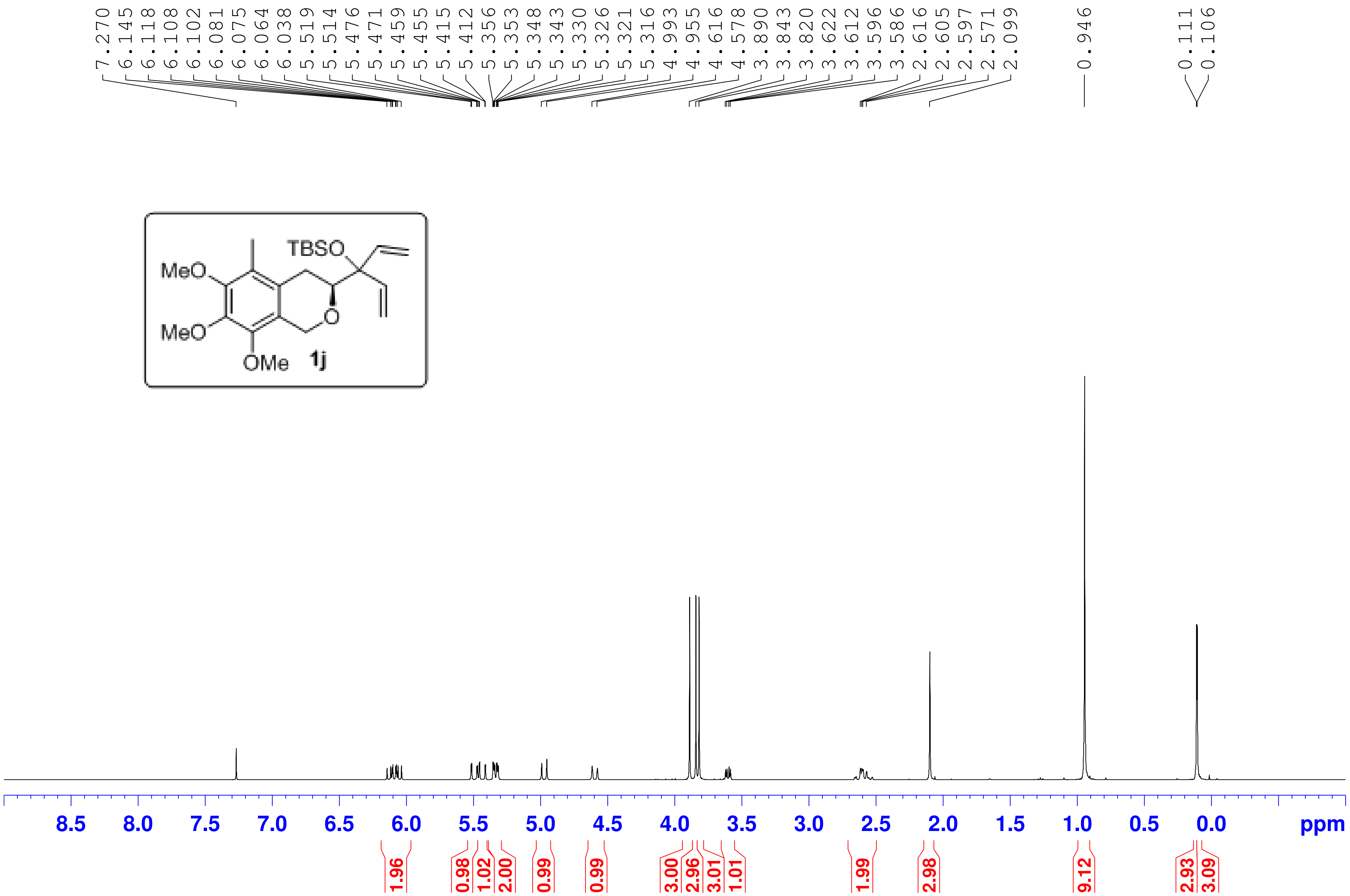

Supplementary Figure 78. ^{13}C NMR Spectrum of 1j (100 MHz, $CDCl_3$)

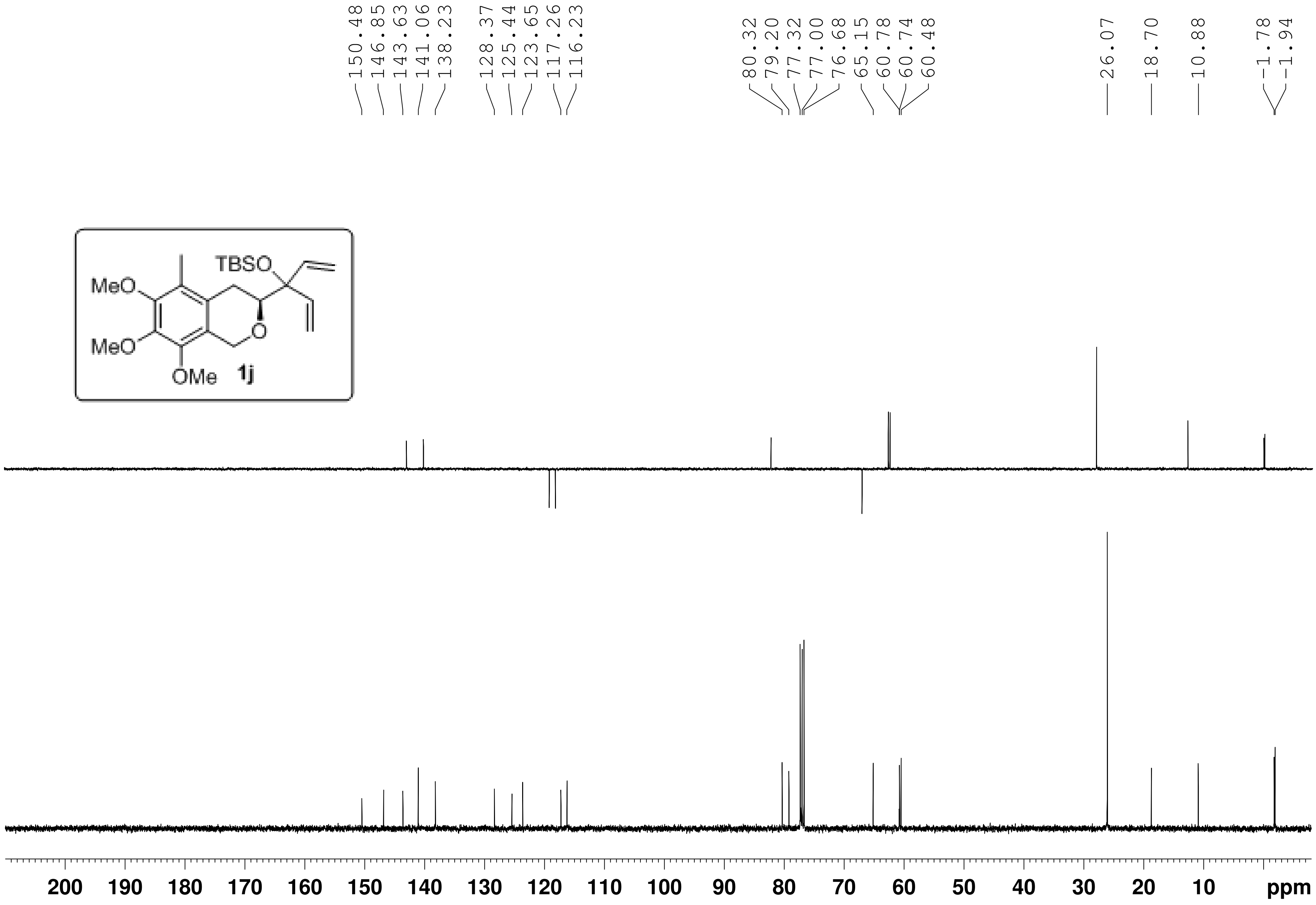

Supplementary Figure 79. ^{1}H NMR Spectrum of 2j (400 MHz, $CDCl_3$)

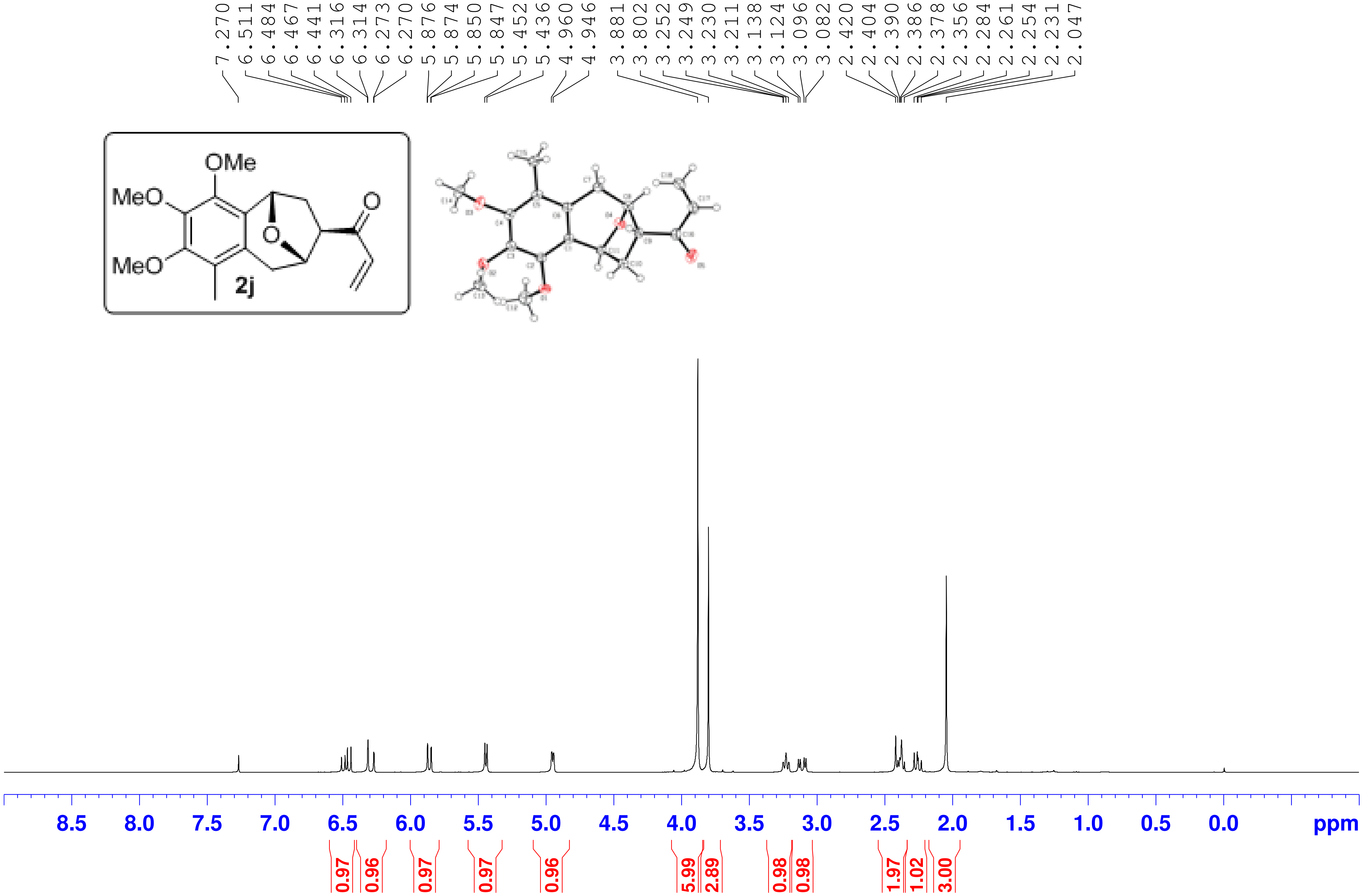

Supplementary Figure 80. ^{13}C NMR Spectrum of 2j (100 MHz, $CDCl_3$)

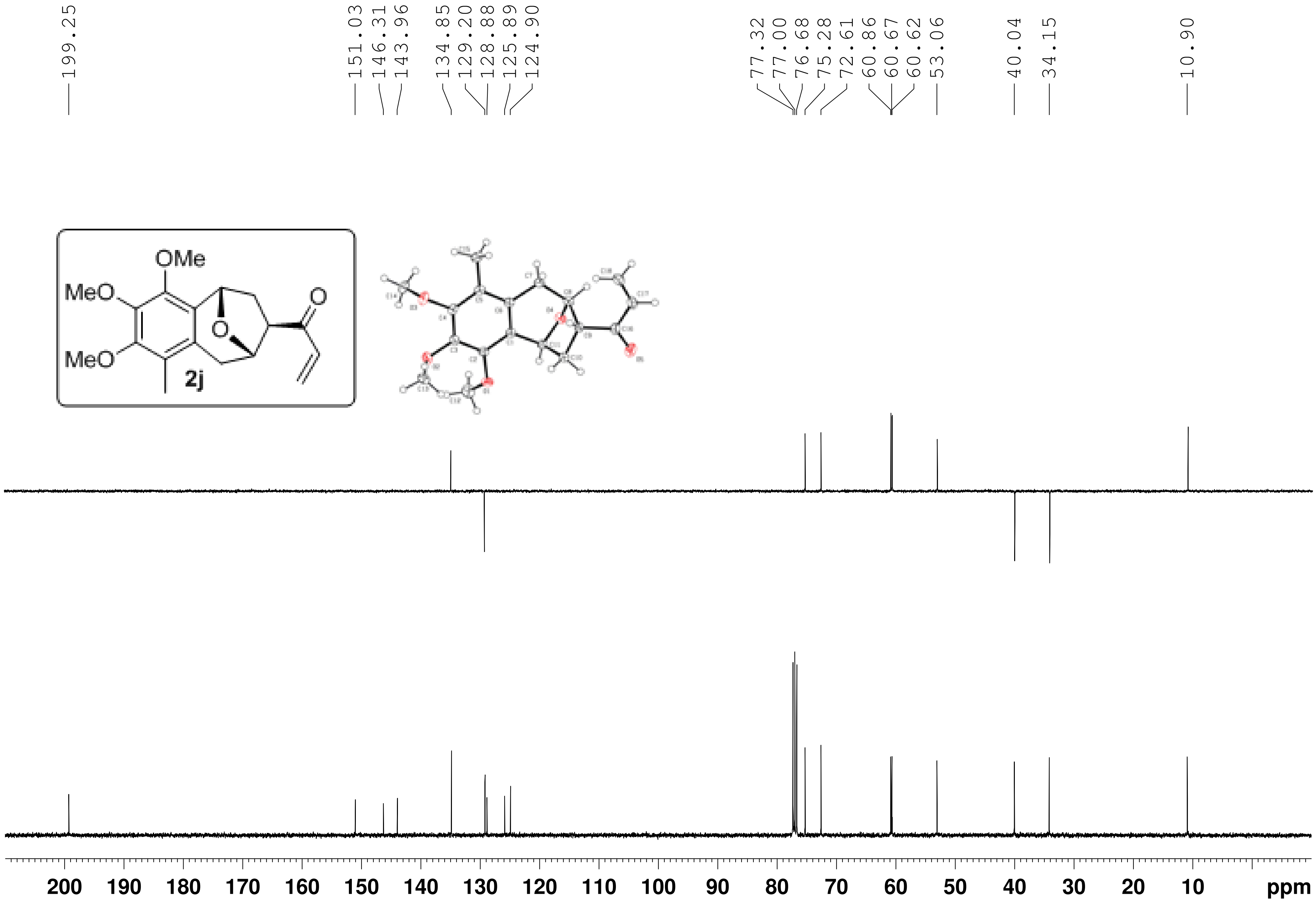

Supplementary Figure 81. ^{1}H NMR Spectrum of S23 (400 MHz, $CDCl_3$)

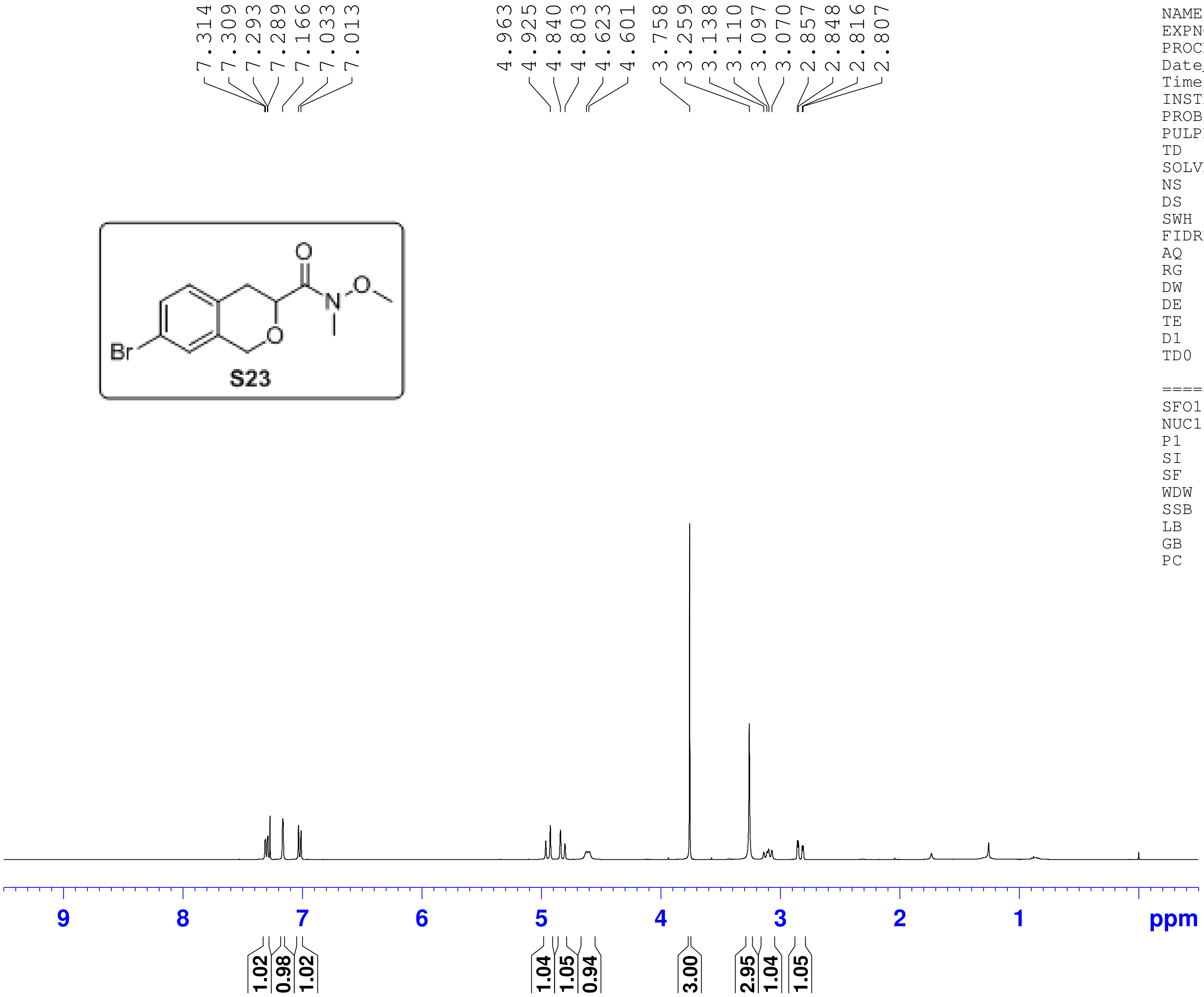


```
NAME        zhangqing A390
EXPNO                   10
PROCNO                   1
Date_             20140925
Time                  0.19
INSTRUM              spect
PROBHD     5 mm PABBO BB/
PULPROG               zg30
TD                   65536
SOLVENT              CDCl3
NS                       8
DS                       2
SWH               8012.820 Hz
FIDRES            0.122266 Hz
AQ               4.0894966 sec
RG                     128
DW                  62.400 usec
DE                    6.50 usec
TE                   295.4 K
D1              1.00000000 sec
TD0                      1

======== CHANNEL f1 ========
SFO1         400.1324710 MHz
NUC1                  1H
P1                 11.60 usec
SI                 65536
SF           400.1300049 MHz
WDW                   EM
SSB                    0
LB                  0.30 Hz
GB                     0
PC                  1.00
```

Supplementary Figure 82. ^{13}C NMR Spectrum of S23 (100 MHz, $CDCl_3$)

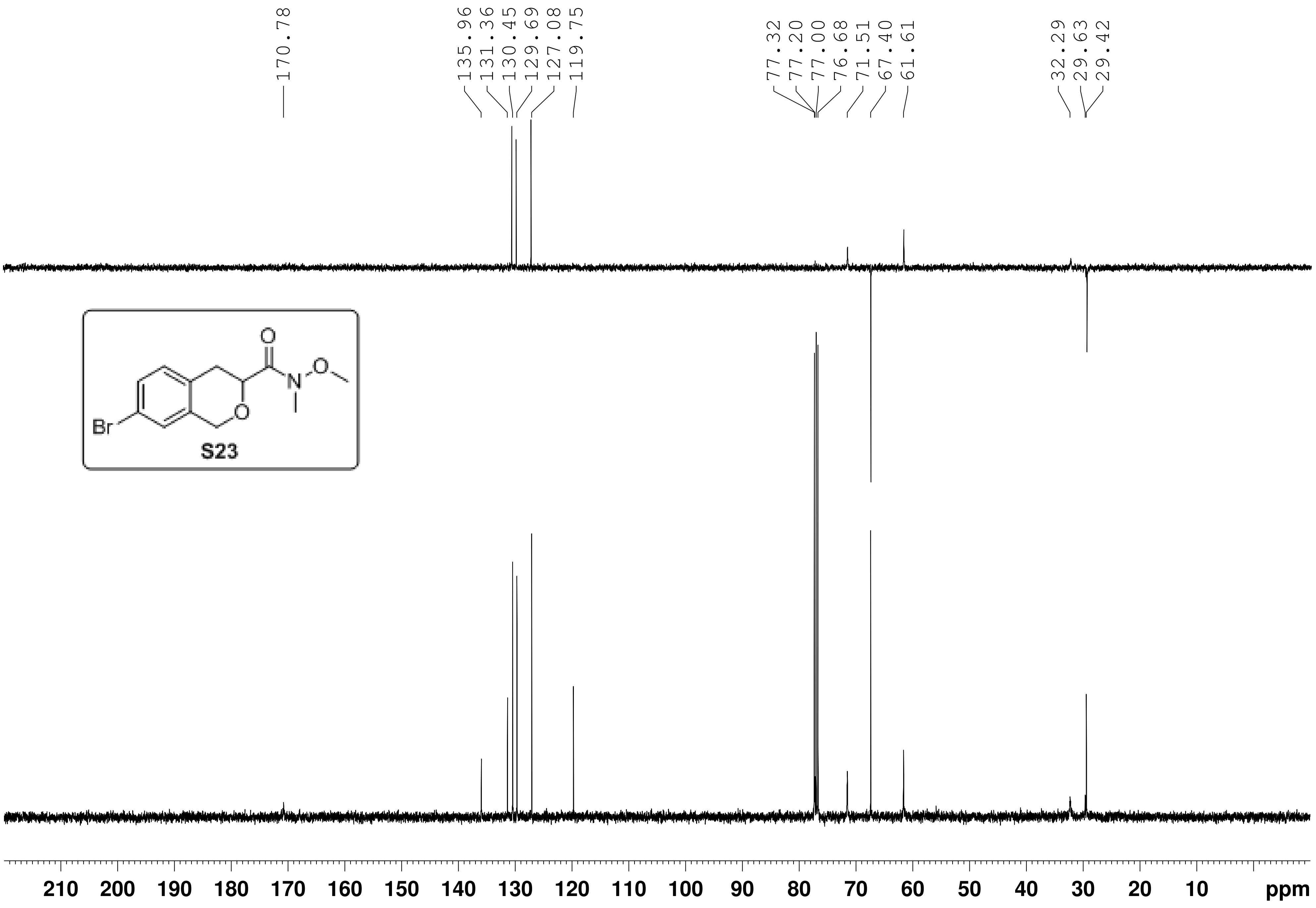

Supplementary Figure 83. ^{1}H NMR Spectrum of S24 (400 MHz, $CDCl_3$)

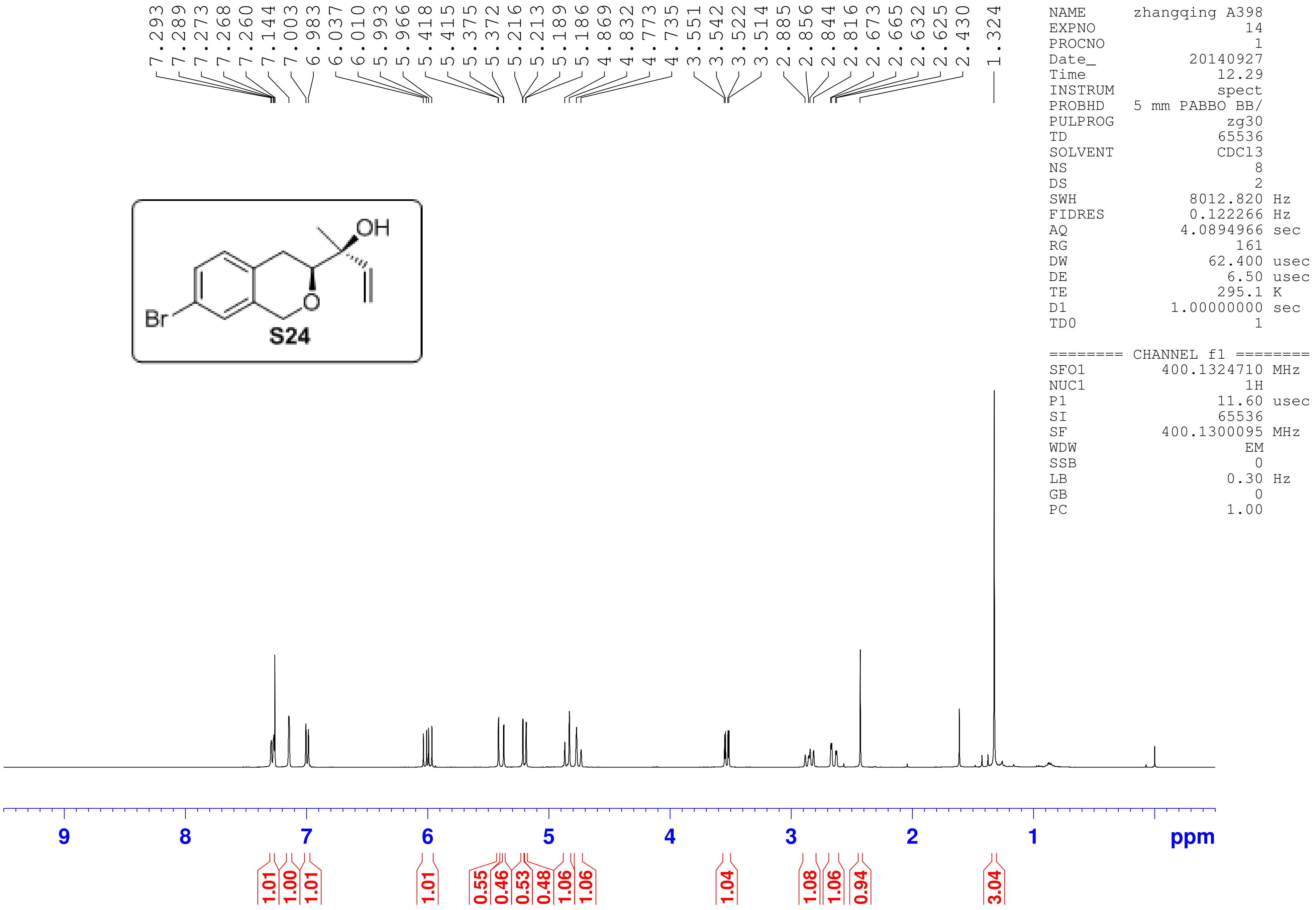

Supplementary Figure 84. ^{13}C NMR Spectrum of S24 (100 MHz, $CDCl_3$)

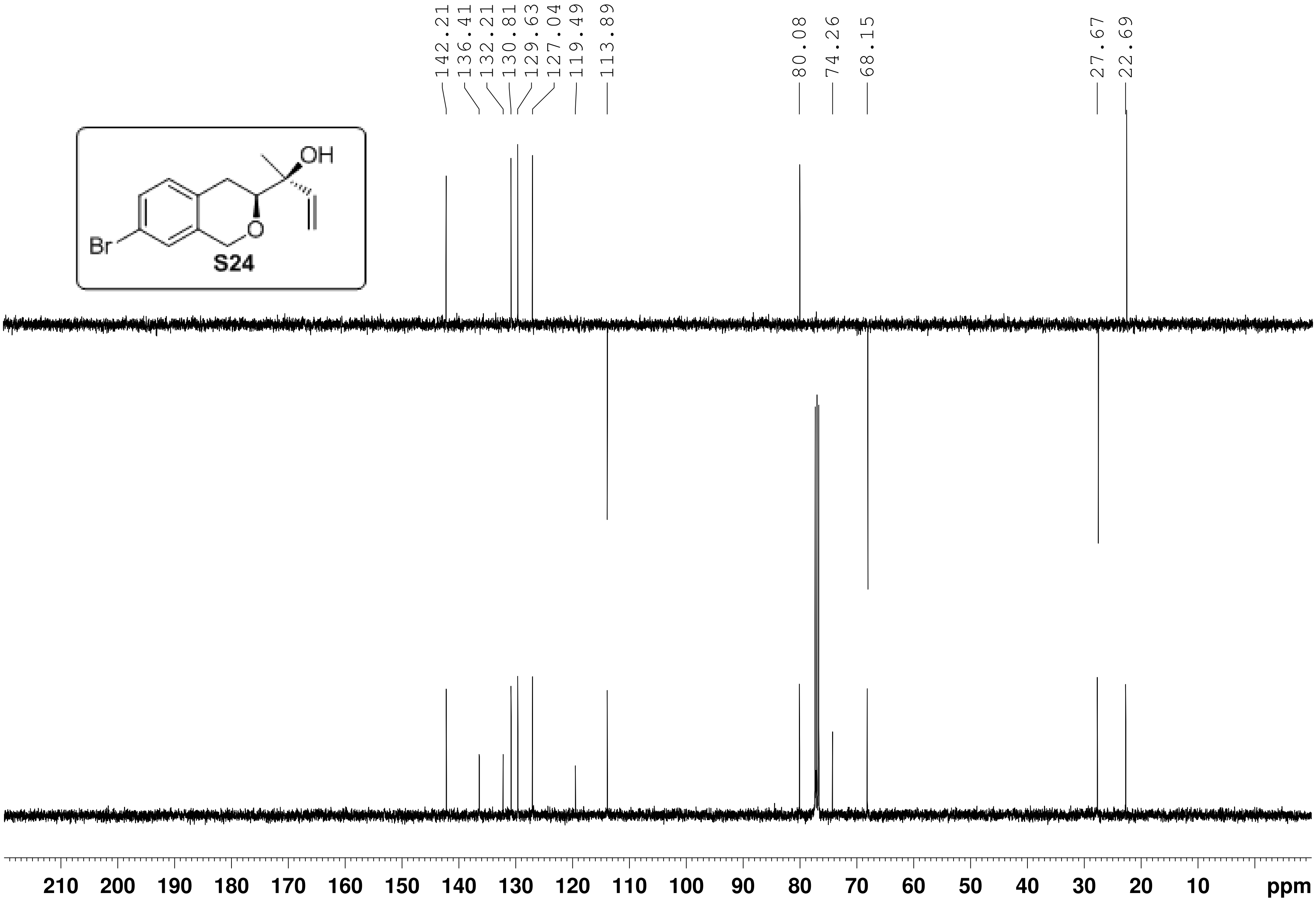

Supplementary Figure 85. ^{1}H NMR Spectrum of 1k (400 MHz, $CDCl_3$)

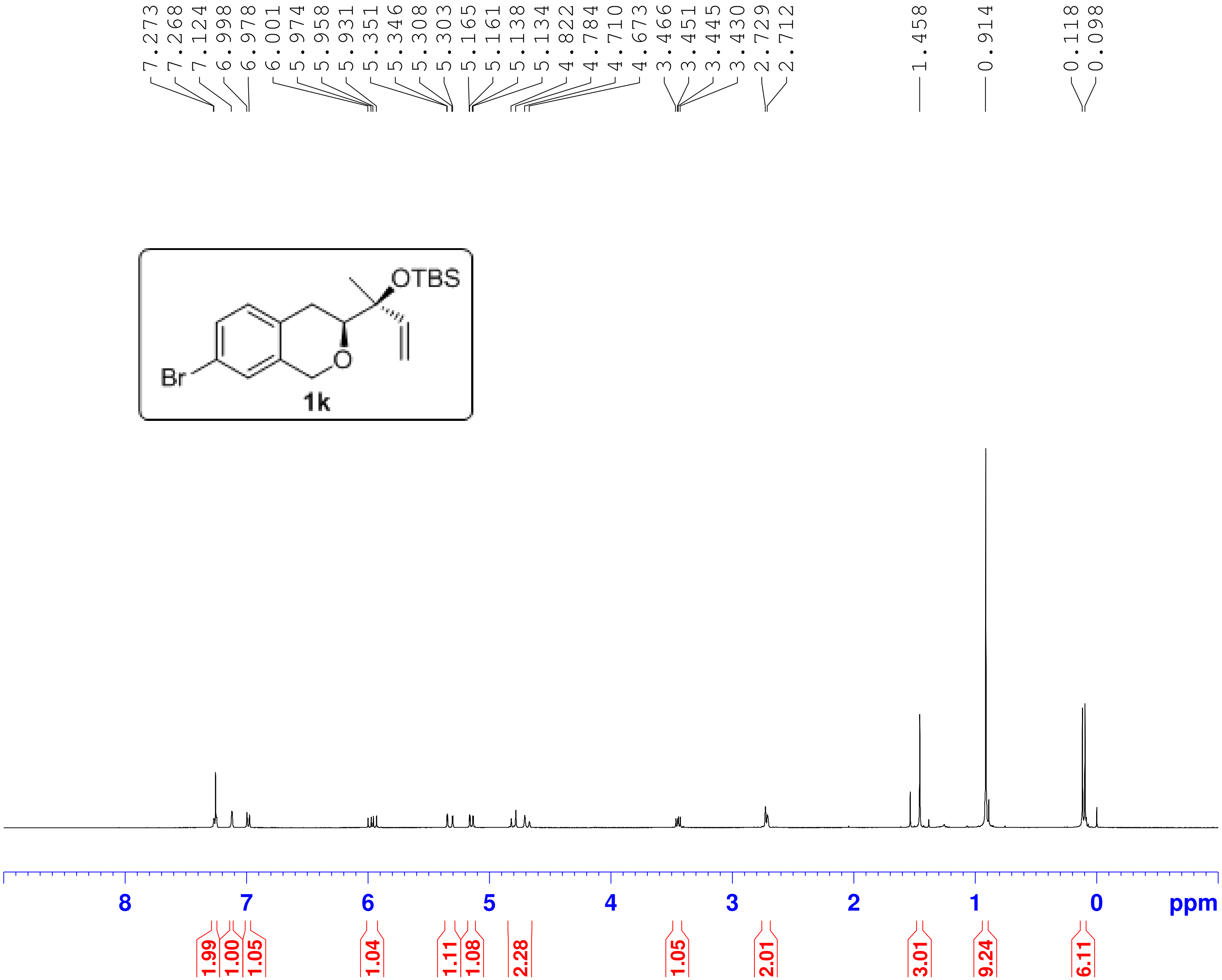

Supplementary Figure 86. ^{13}C NMR Spectrum of 1k (100 MHz, $CDCl_3$)

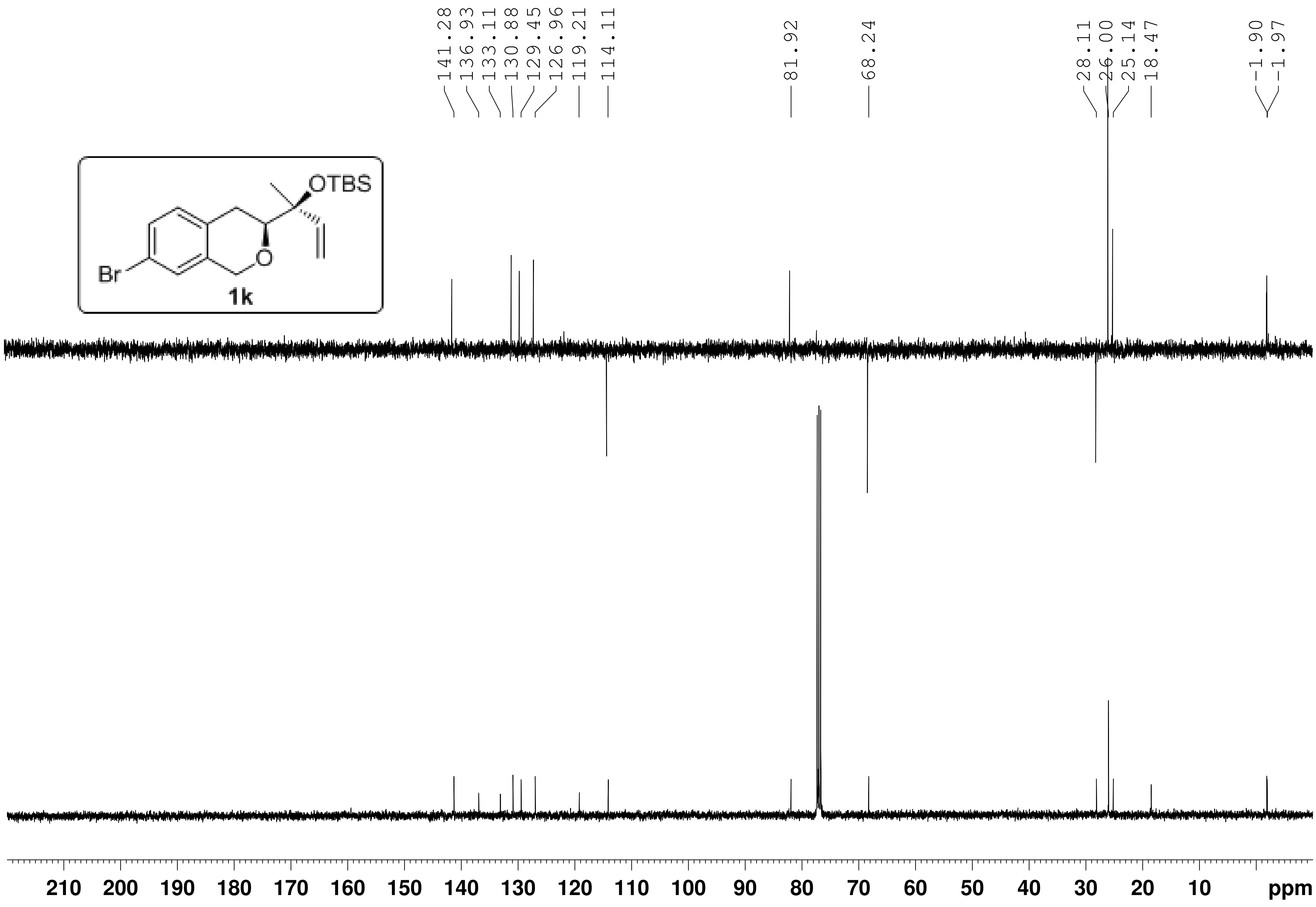

Supplementary Figure 87. ^{1}H NMR Spectrum of 2k (400 MHz, $CDCl_3$)

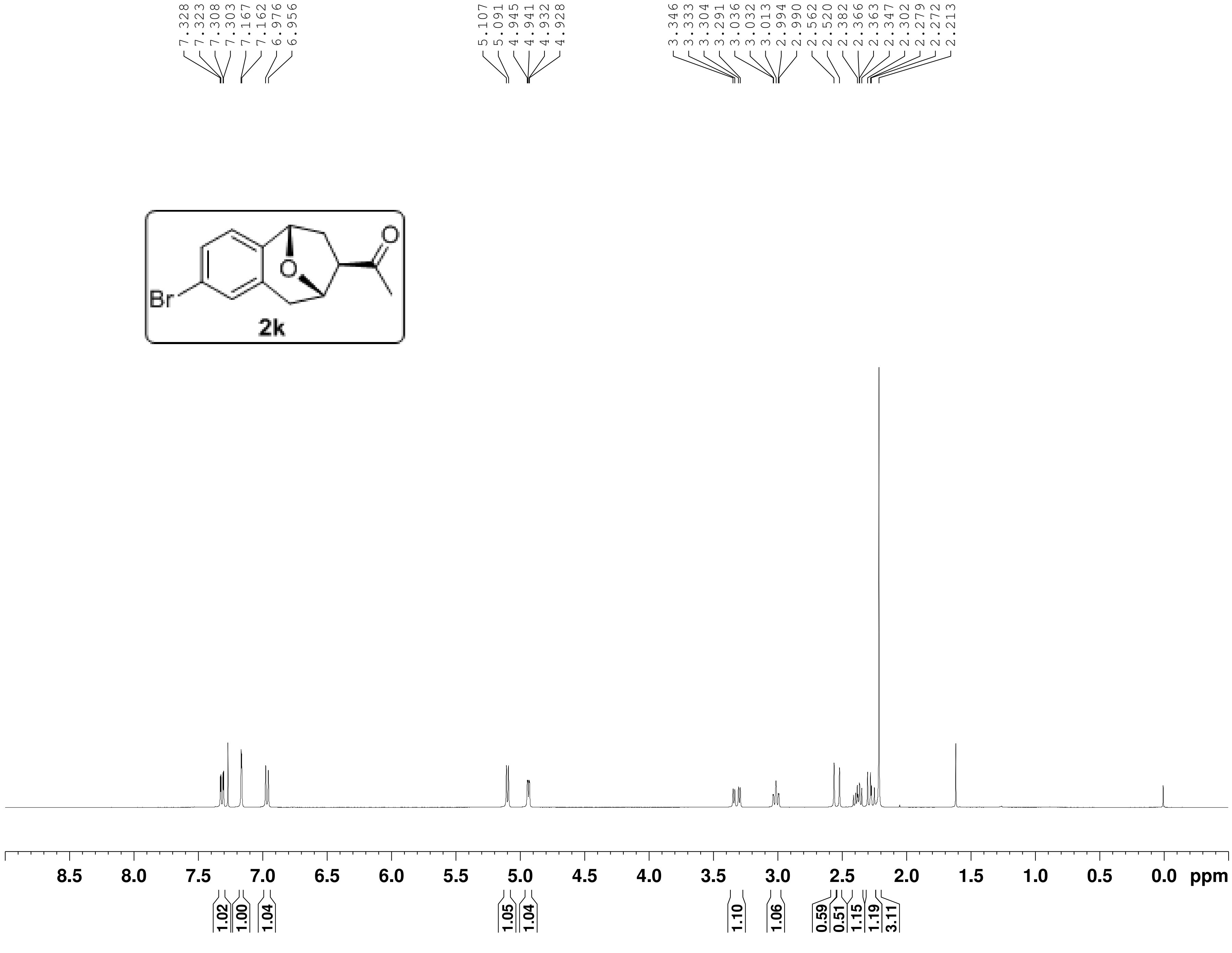

Supplementary Figure 88. ^{13}C NMR Spectrum of 2k (100 MHz, $CDCl_3$)

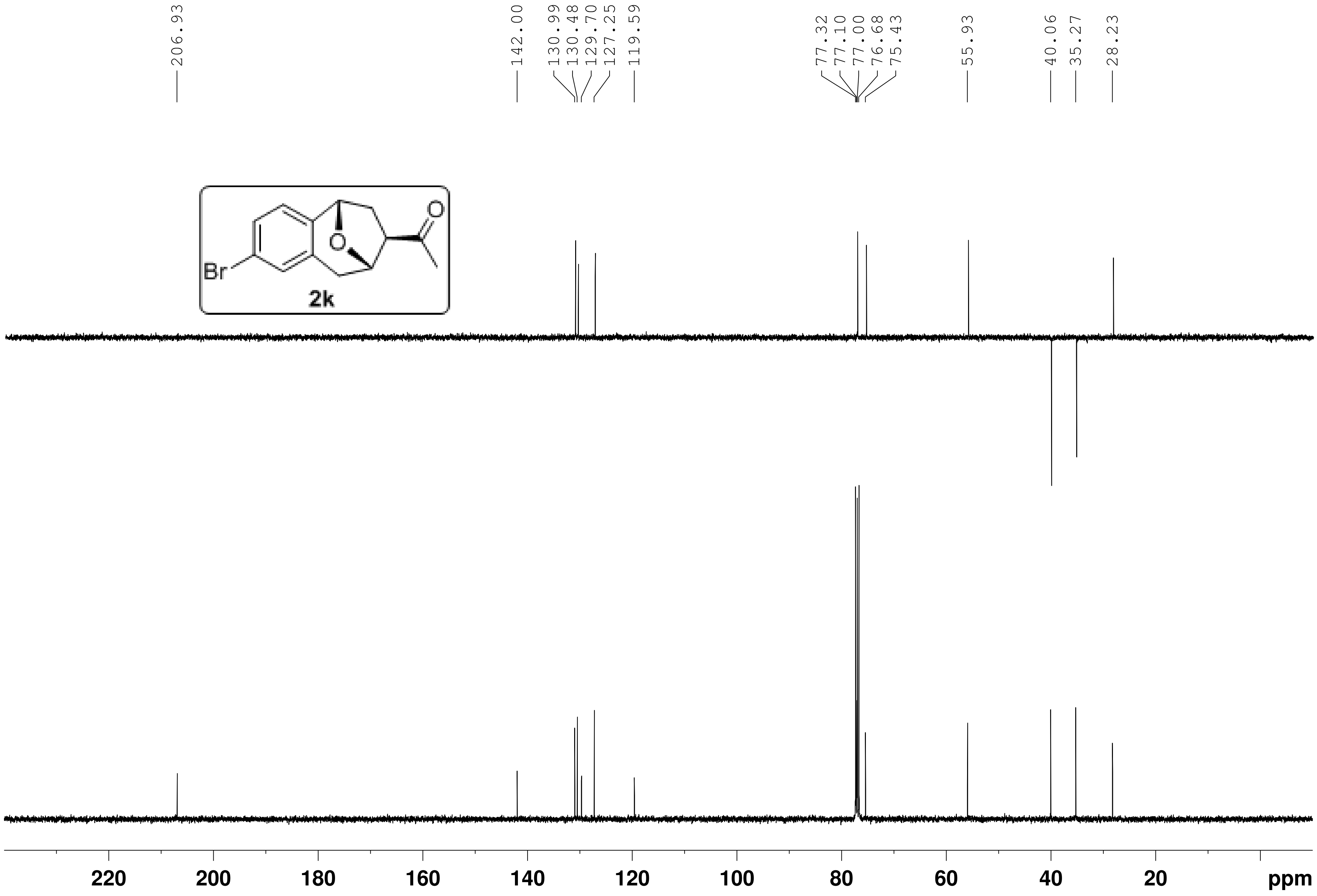

Supplementary Figure 89. ^{1}H NMR Spectrum of S26 (400 MHz, $CDCl_3$)

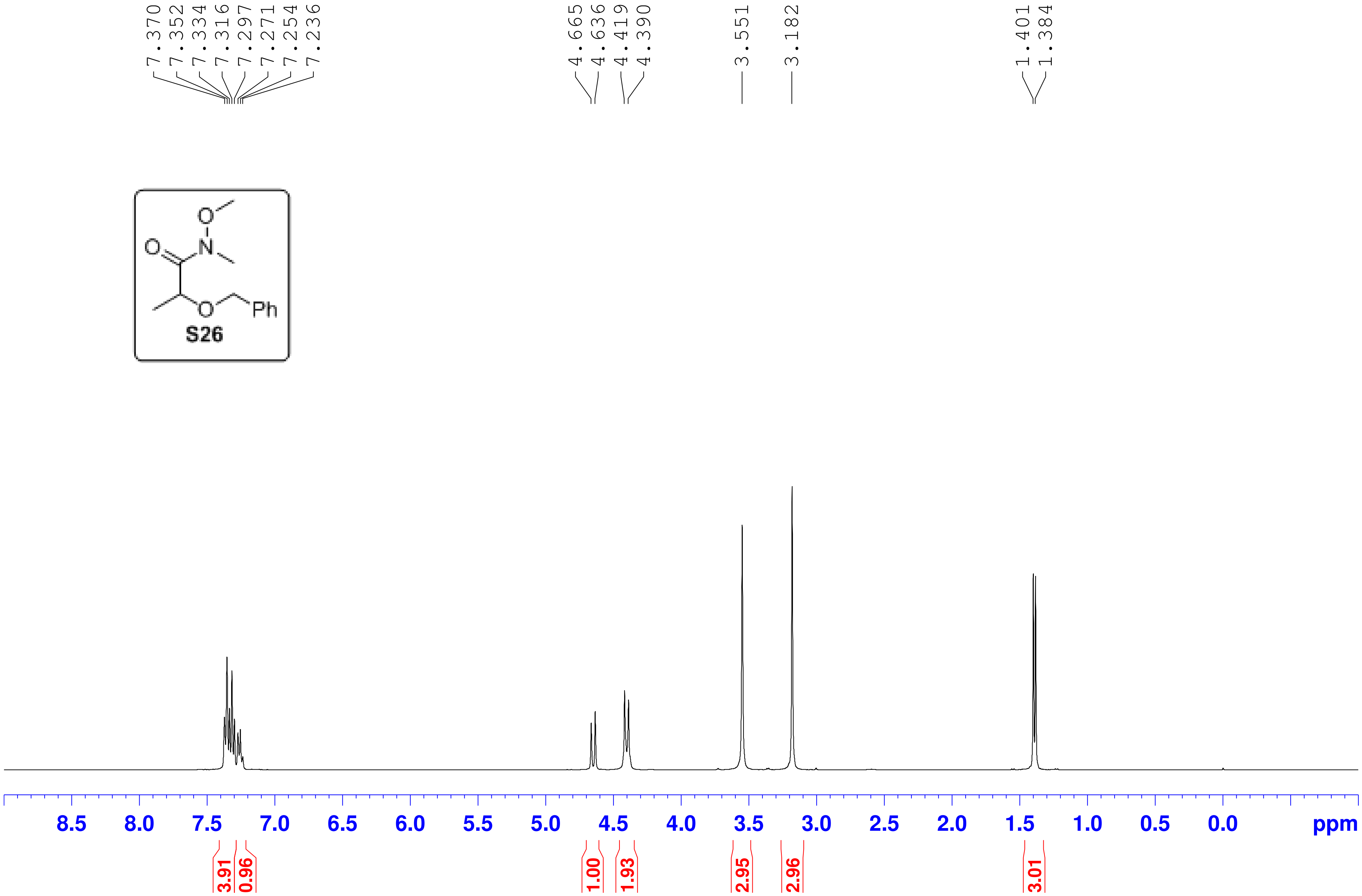

Supplementary Figure 90. ^{13}C NMR Spectrum of S26 (100 MHz, $CDCl_3$)

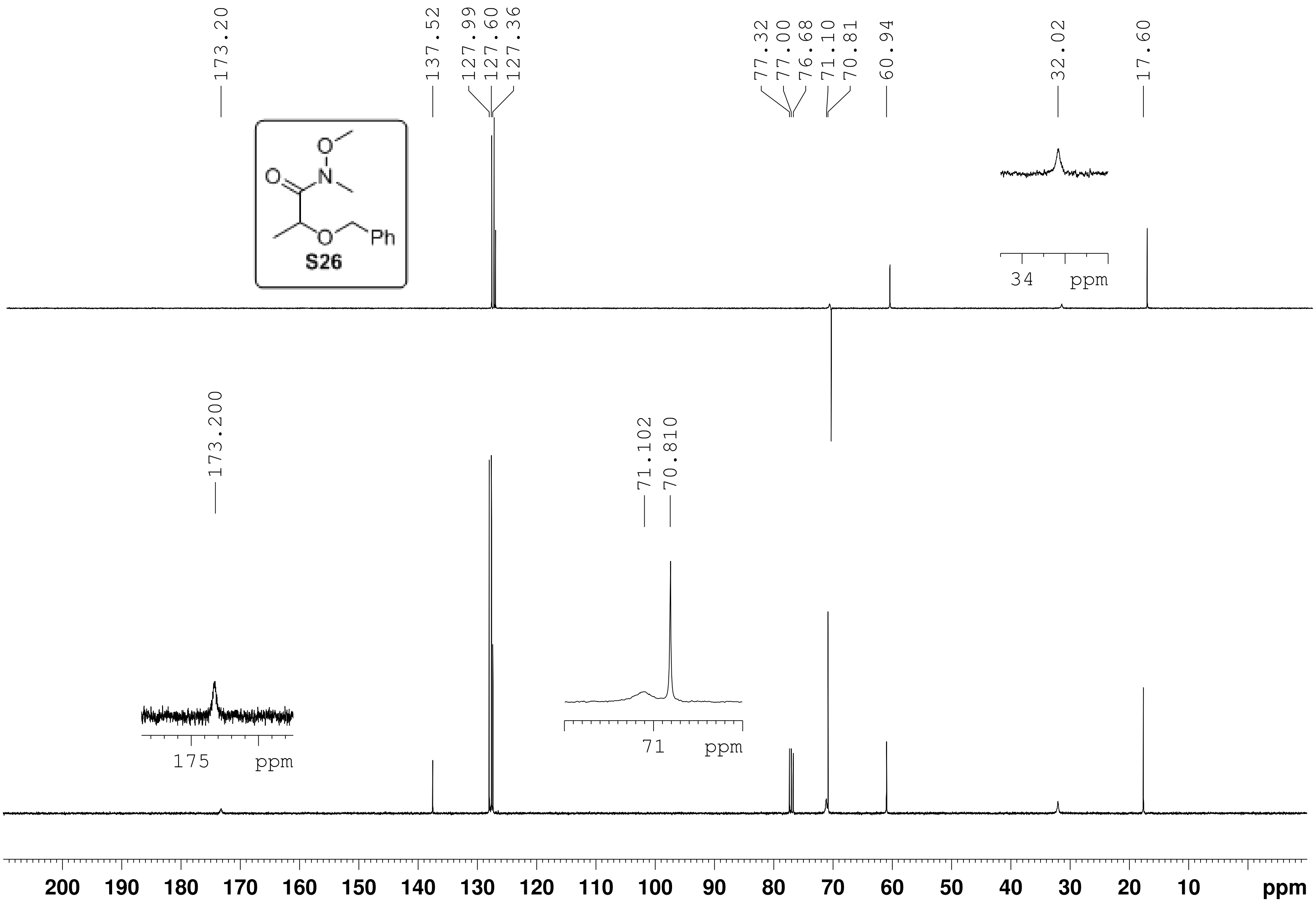

Supplementary Figure 91. ^{1}H NMR Spectrum of S27 (400 MHz, $CDCl_3$)

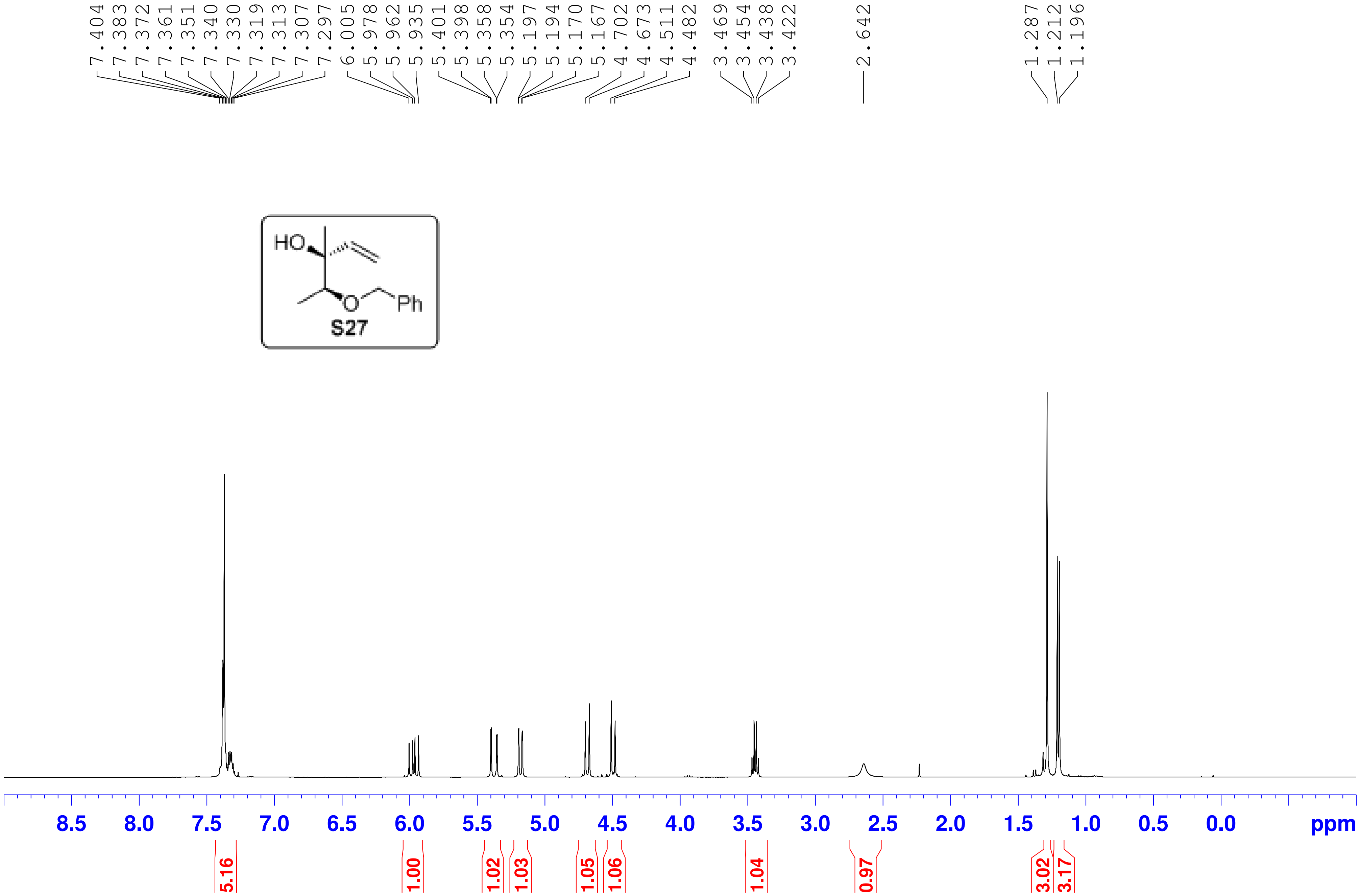

Supplementary Figure 92. ^{13}C NMR Spectrum of S27 (100 MHz, $CDCl_3$)

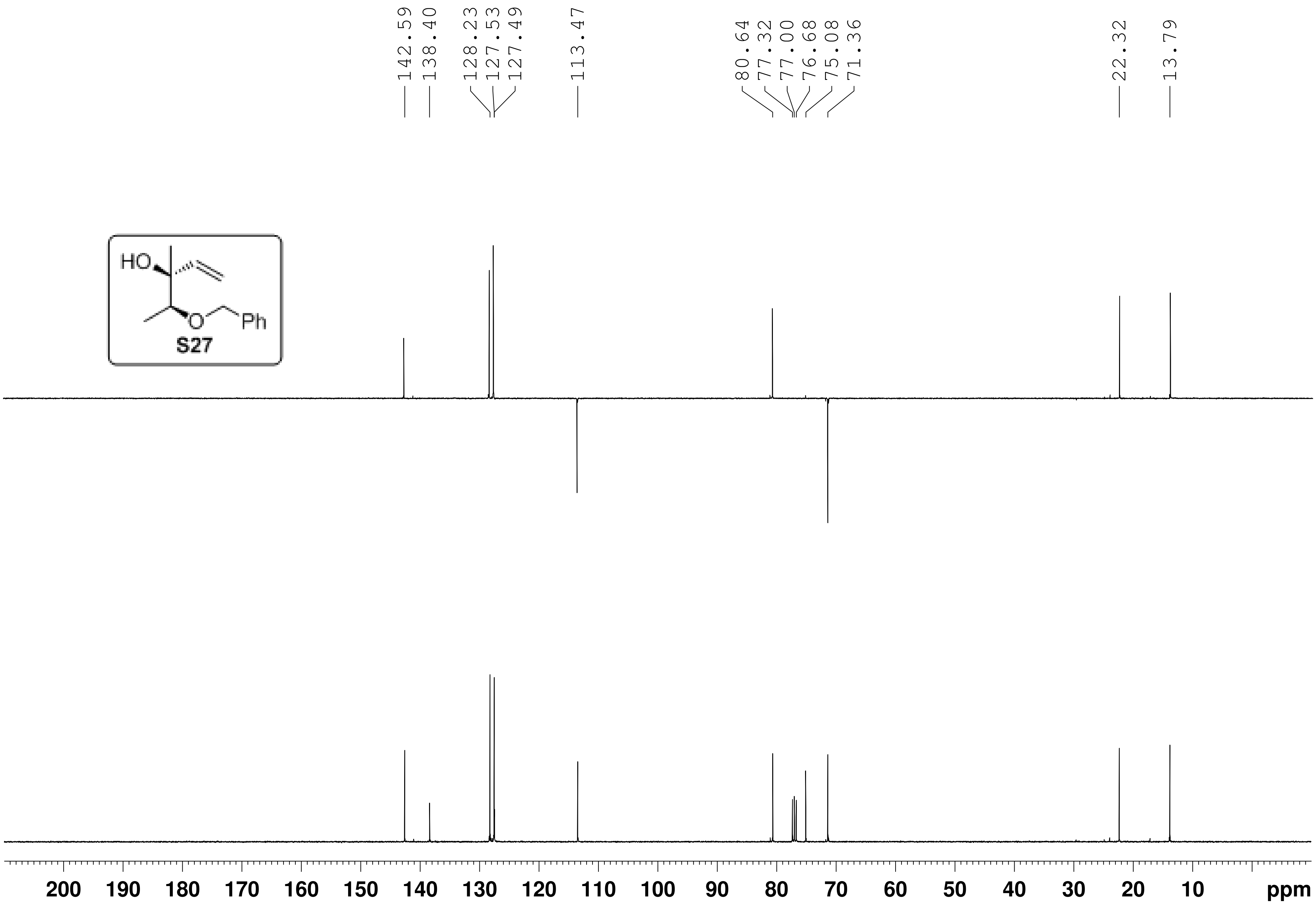

Supplementary Figure 93. ^{1}H NMR Spectrum of 1m (400 MHz, $CDCl_3$)

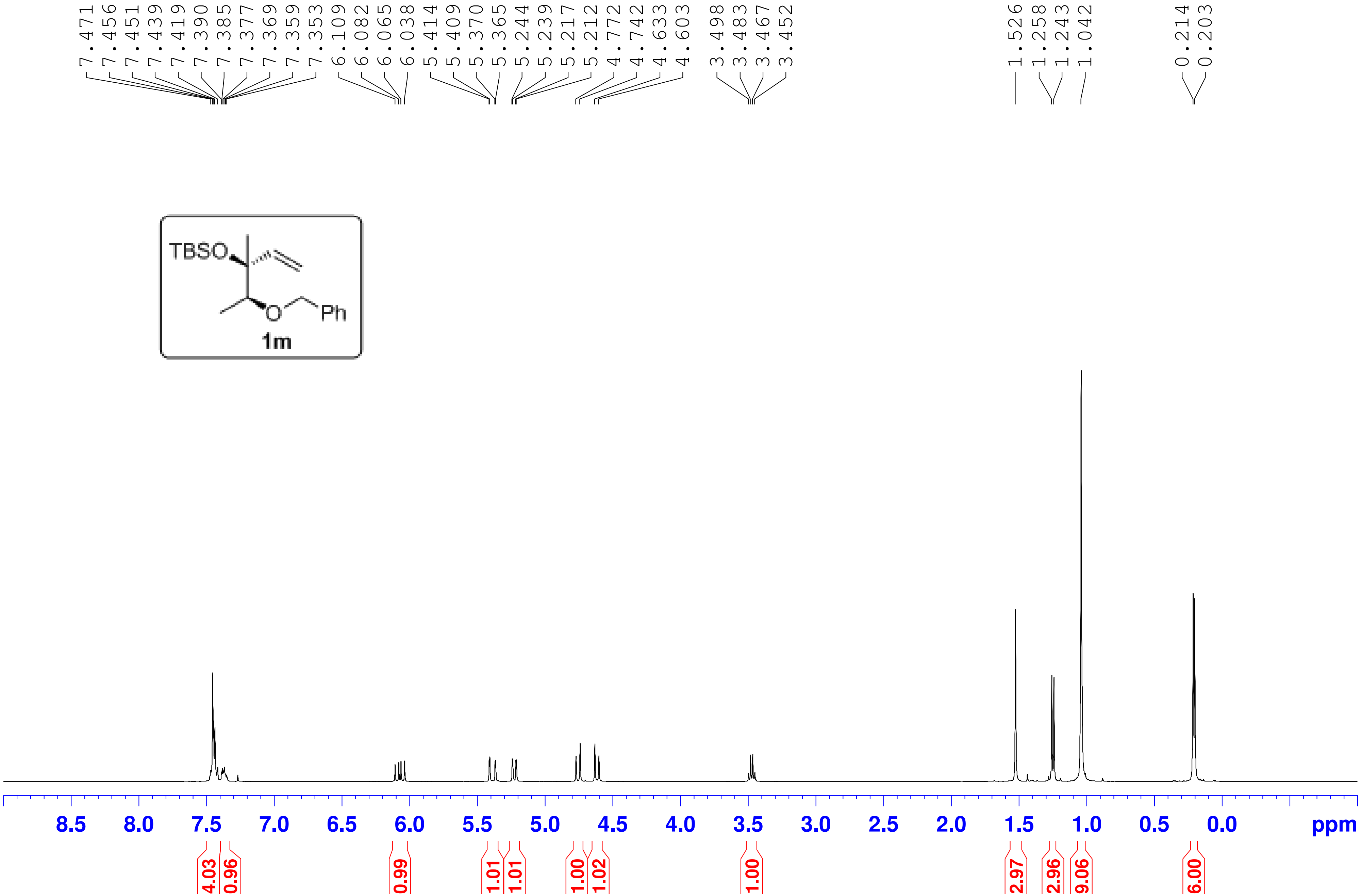

Supplementary Figure 94. ^{13}C NMR Spectrum of 1m (100 MHz, $CDCl_3$)

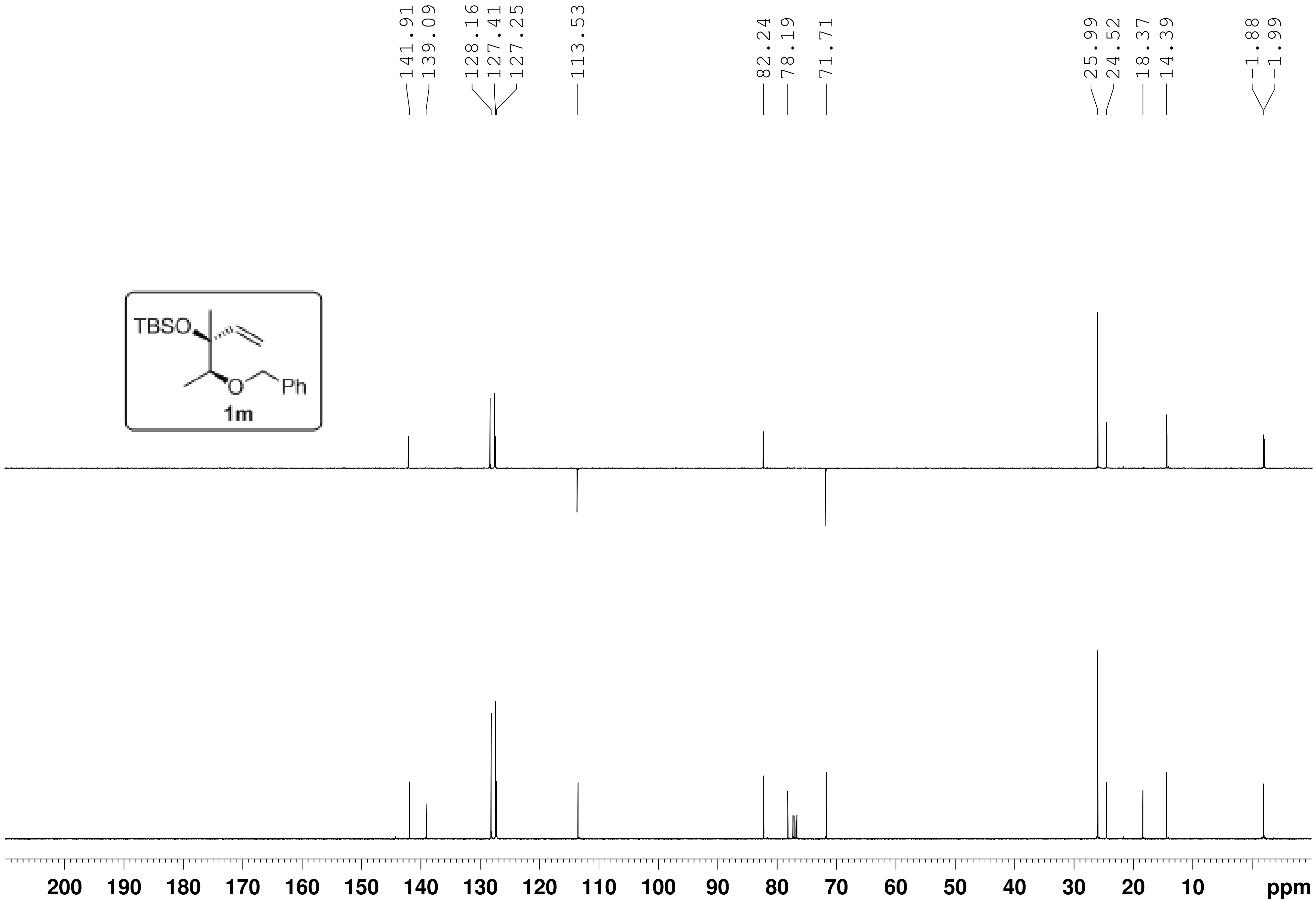

Supplementary Figure 95. ^{1}H NMR Spectrum of 2m (400 MHz, $CDCl_3$)

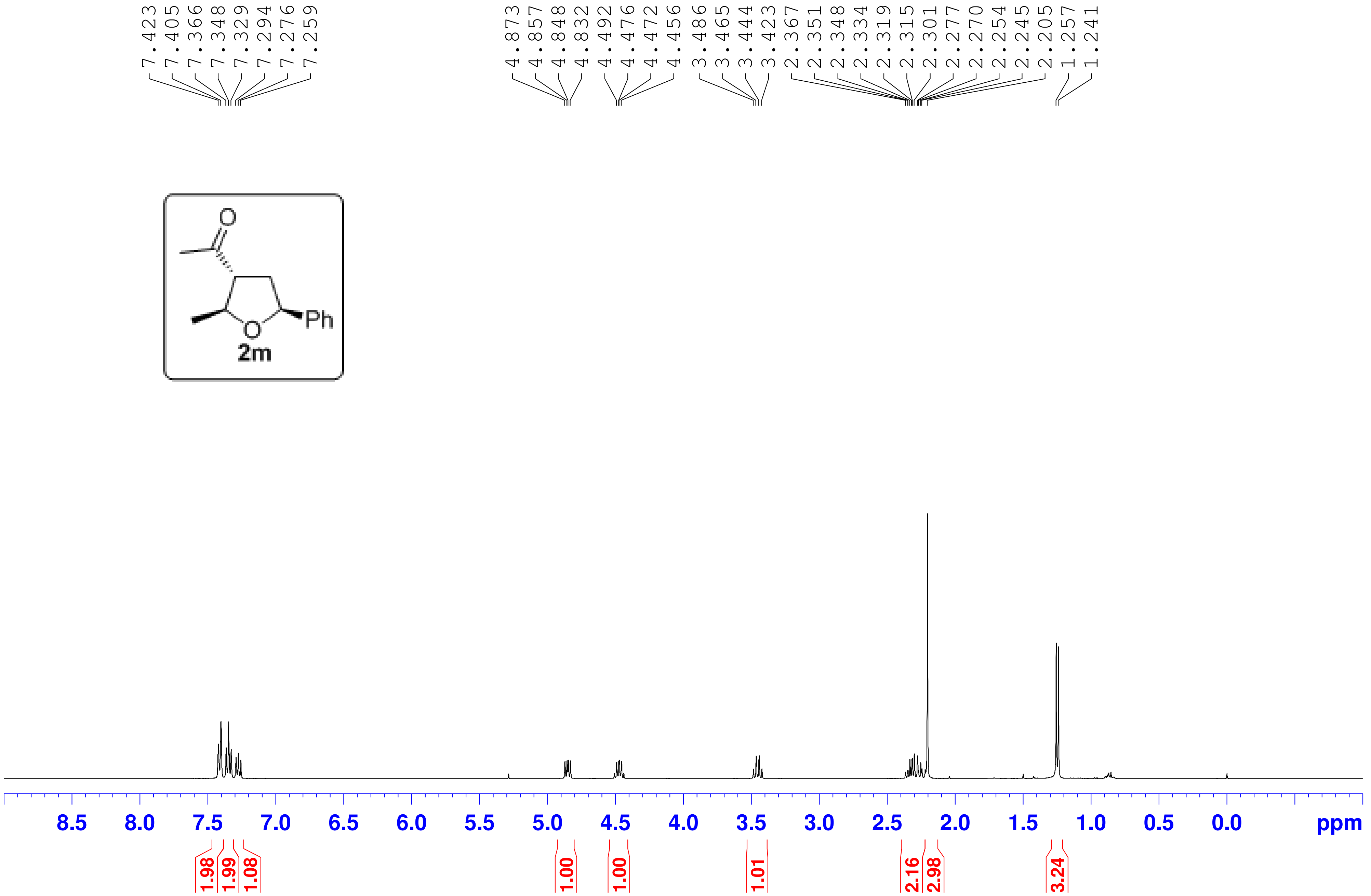

Supplementary Figure 96. ^{13}C NMR Spectrum of 2m (100 MHz, $CDCl_3$)

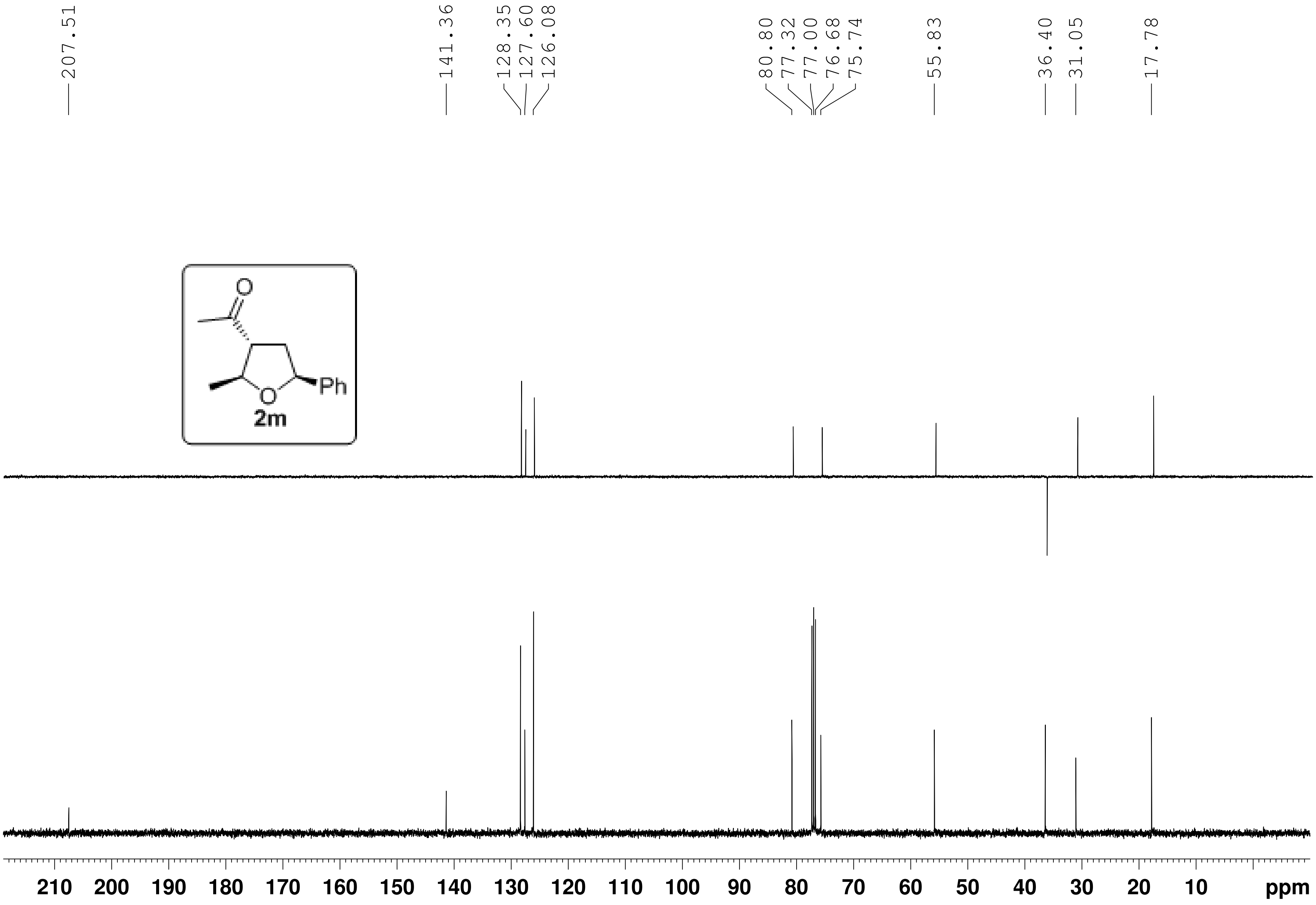

Supplementary Figure 97. ^{1}H NMR Spectrum of 2m' (400 MHz, $CDCl_3$)

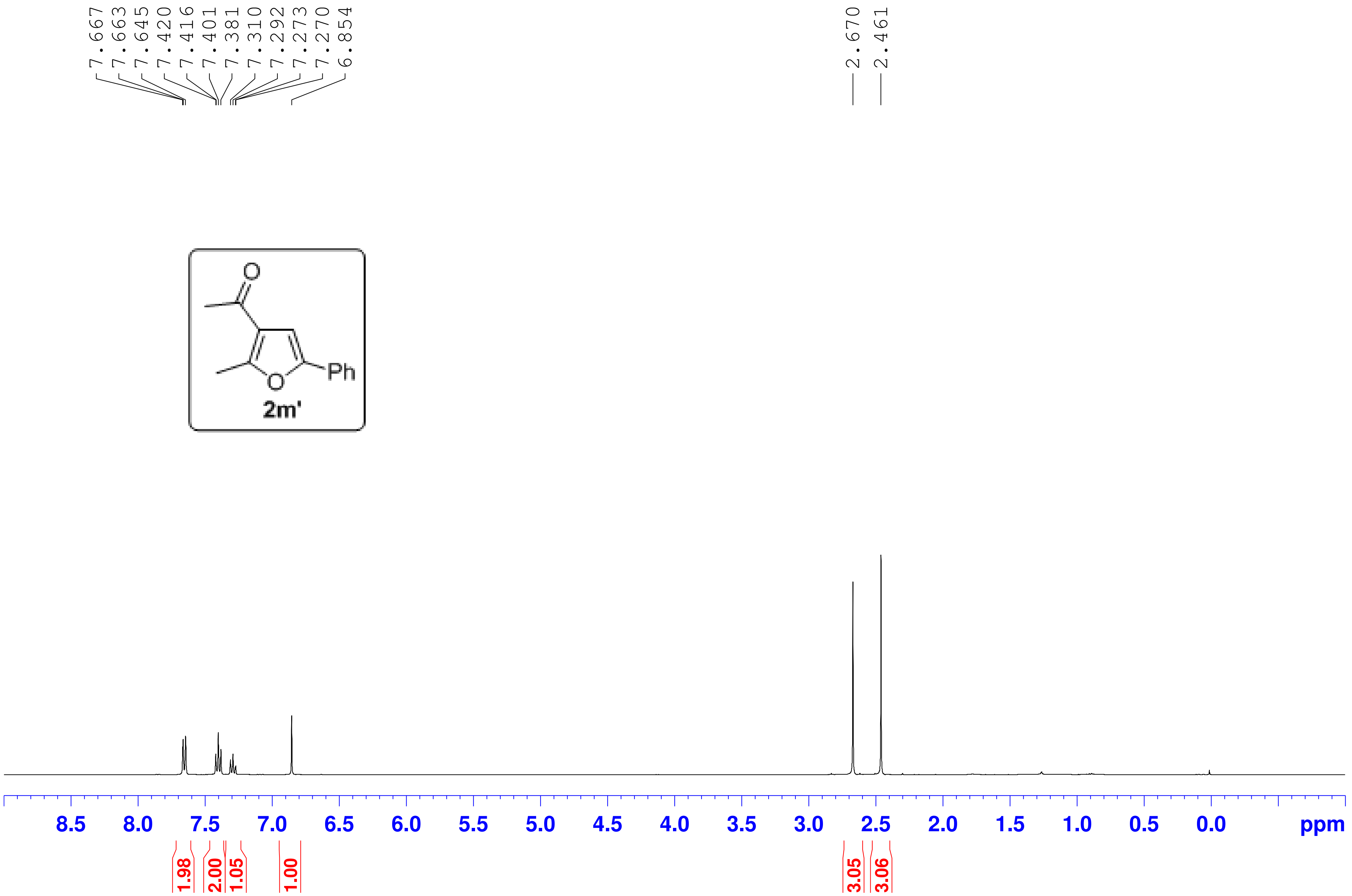

Supplementary Figure 98. ^{13}C NMR Spectrum of 2m' (100 MHz, $CDCl_3$)

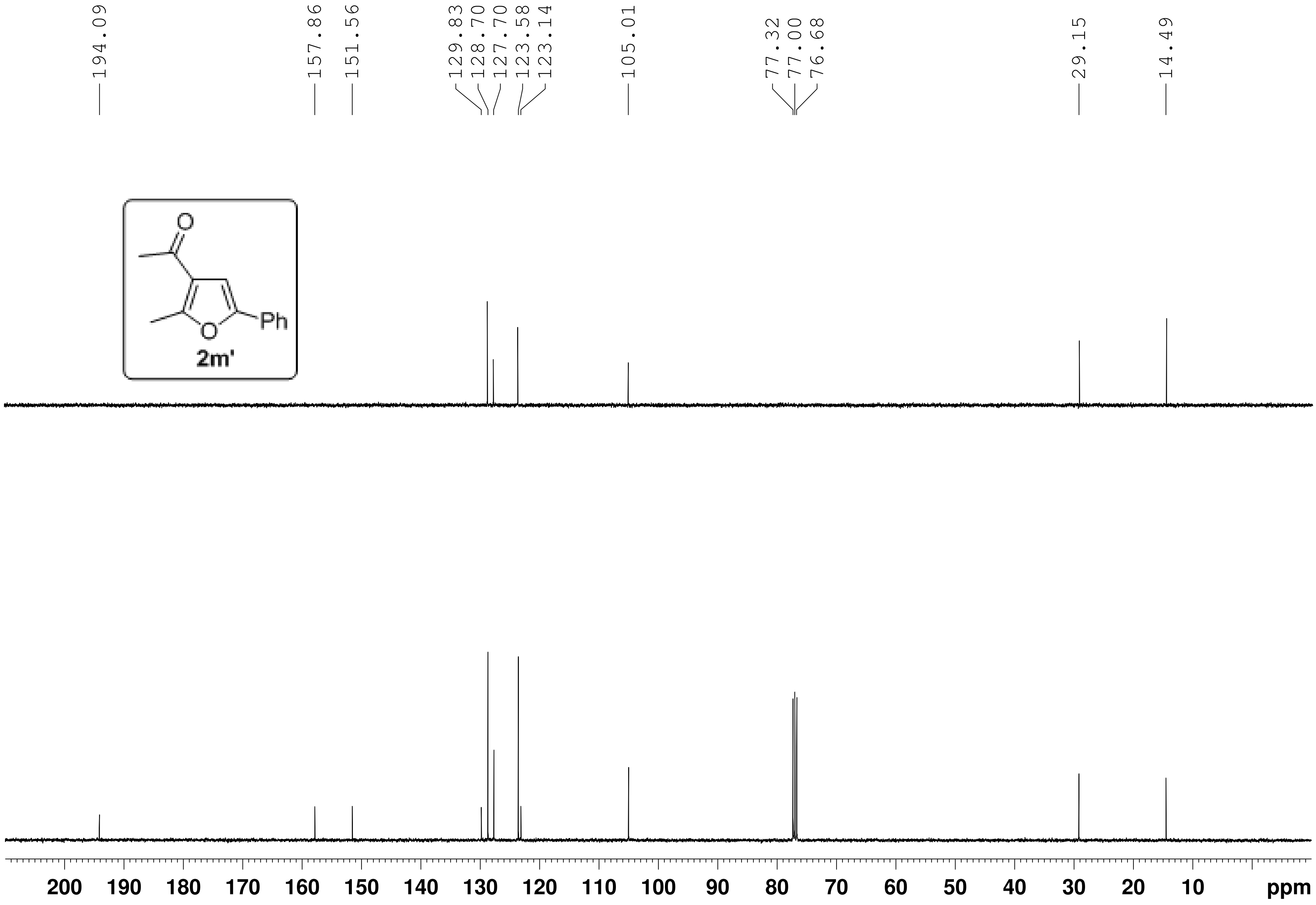

Supplementary Figure 99. ^{1}H NMR Spectrum of S29 (400 MHz, $CDCl_3$)

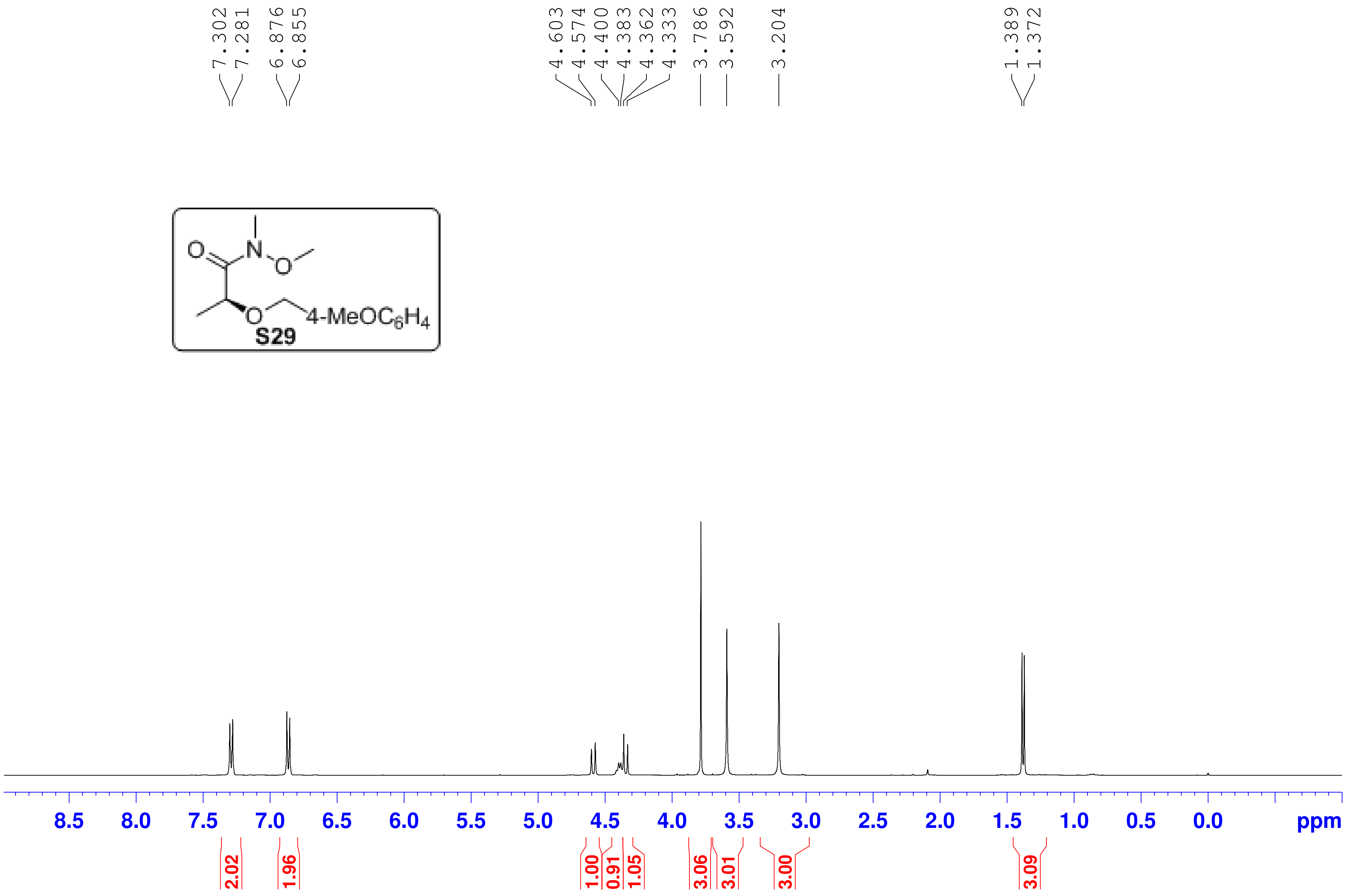

Supplementary Figure 100. ^{13}C NMR Spectrum of S29 (100 MHz, $CDCl_3$)

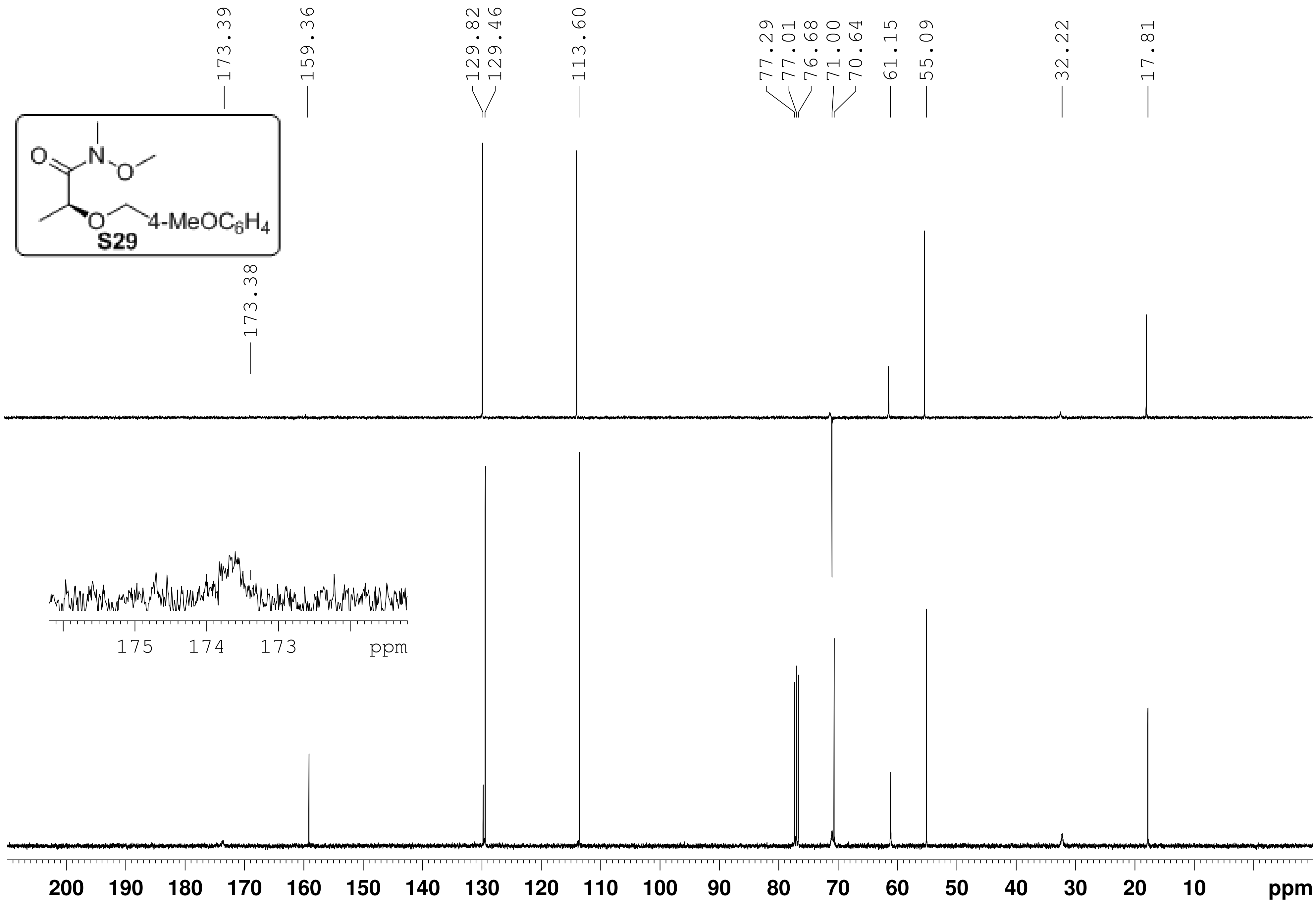

Supplementary Figure 101. ^1H NMR Spectrum of S30 (400 MHz, $CDCl_3$)

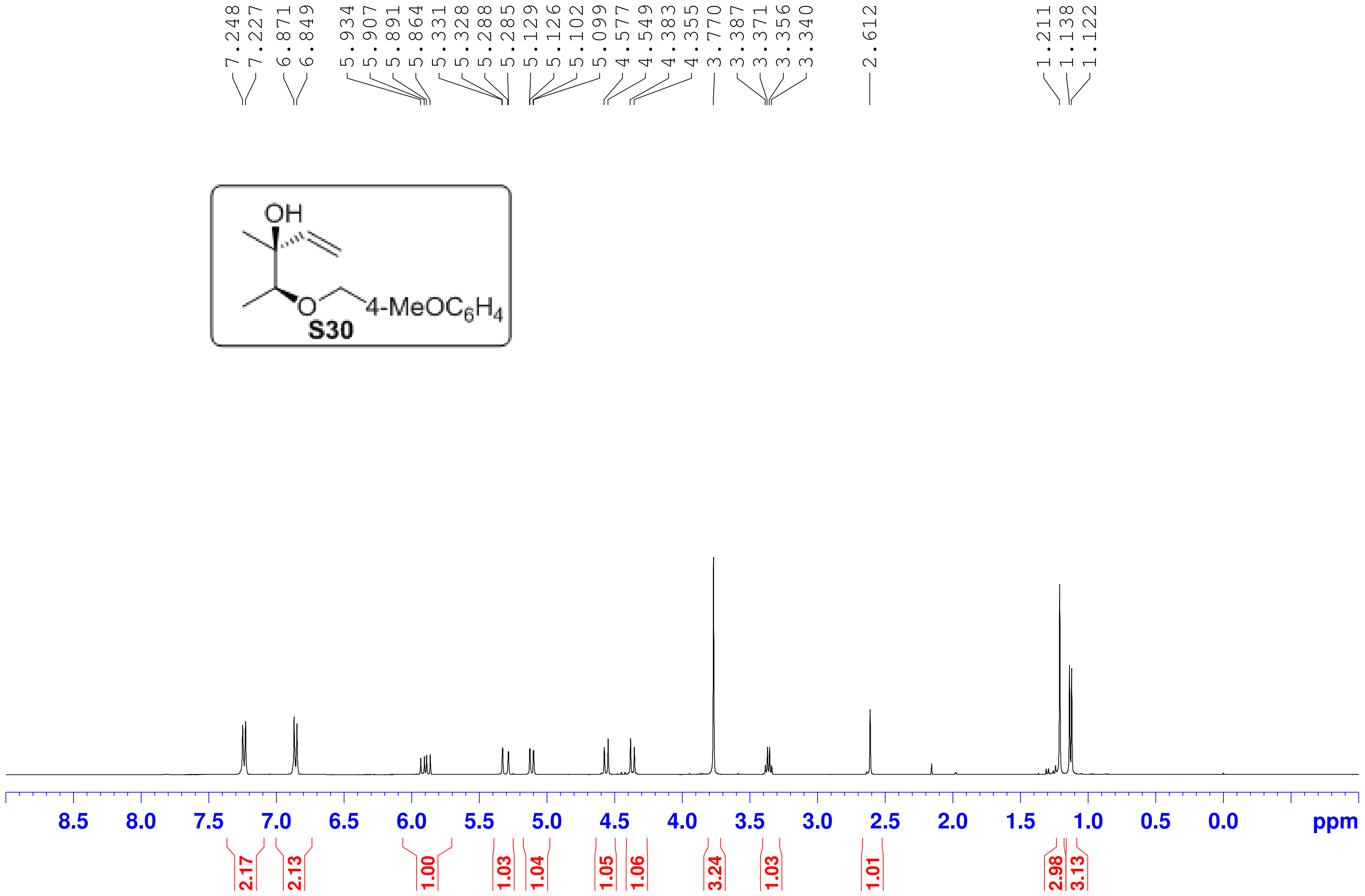

Supplementary Figure 102. ^{13}C NMR Spectrum of S30 (100 MHz, $CDCl_3$)

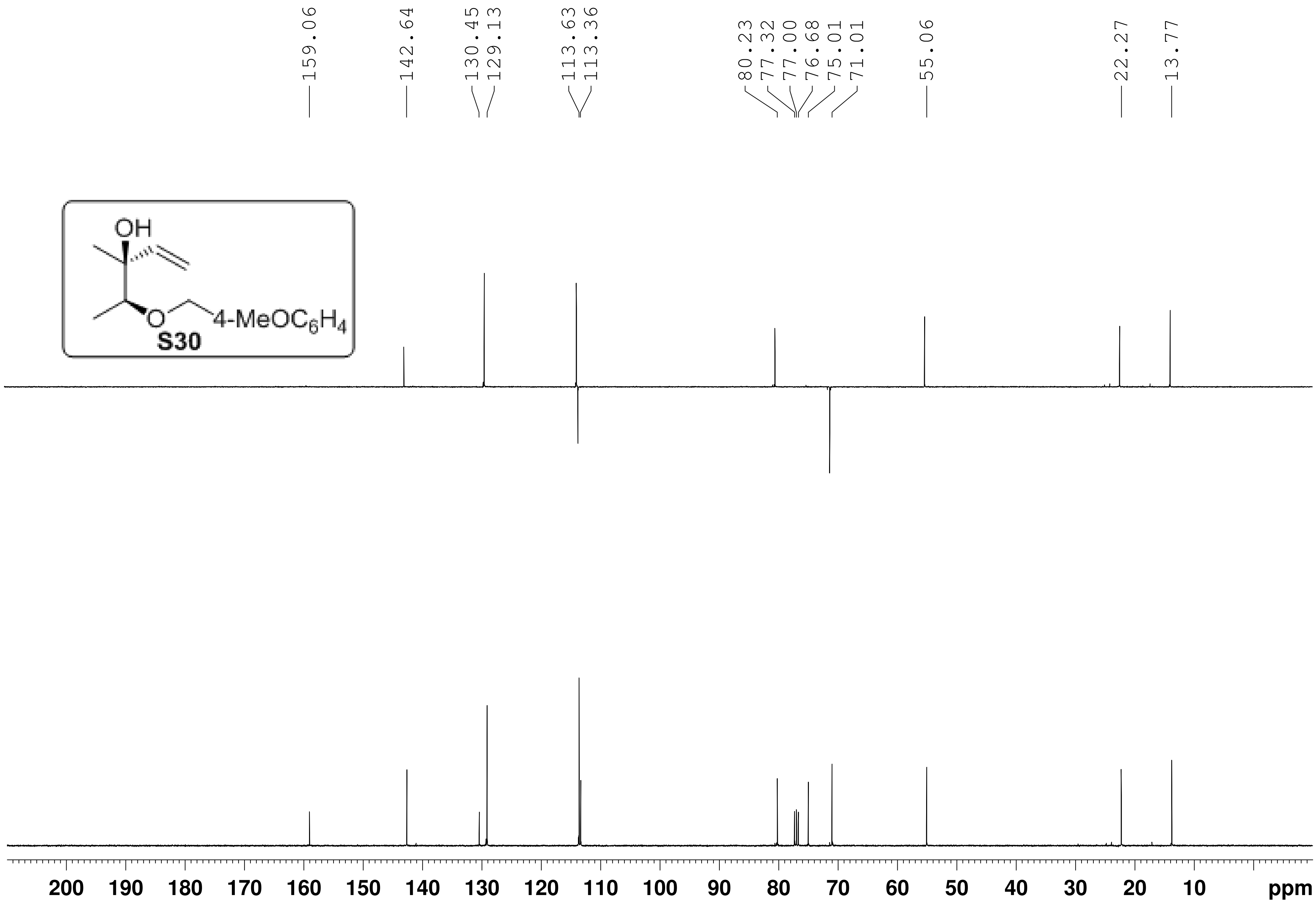

Supplementary Figure 103. ^1H NMR Spectrum of 1n (400 MHz, $CDCl_3$)

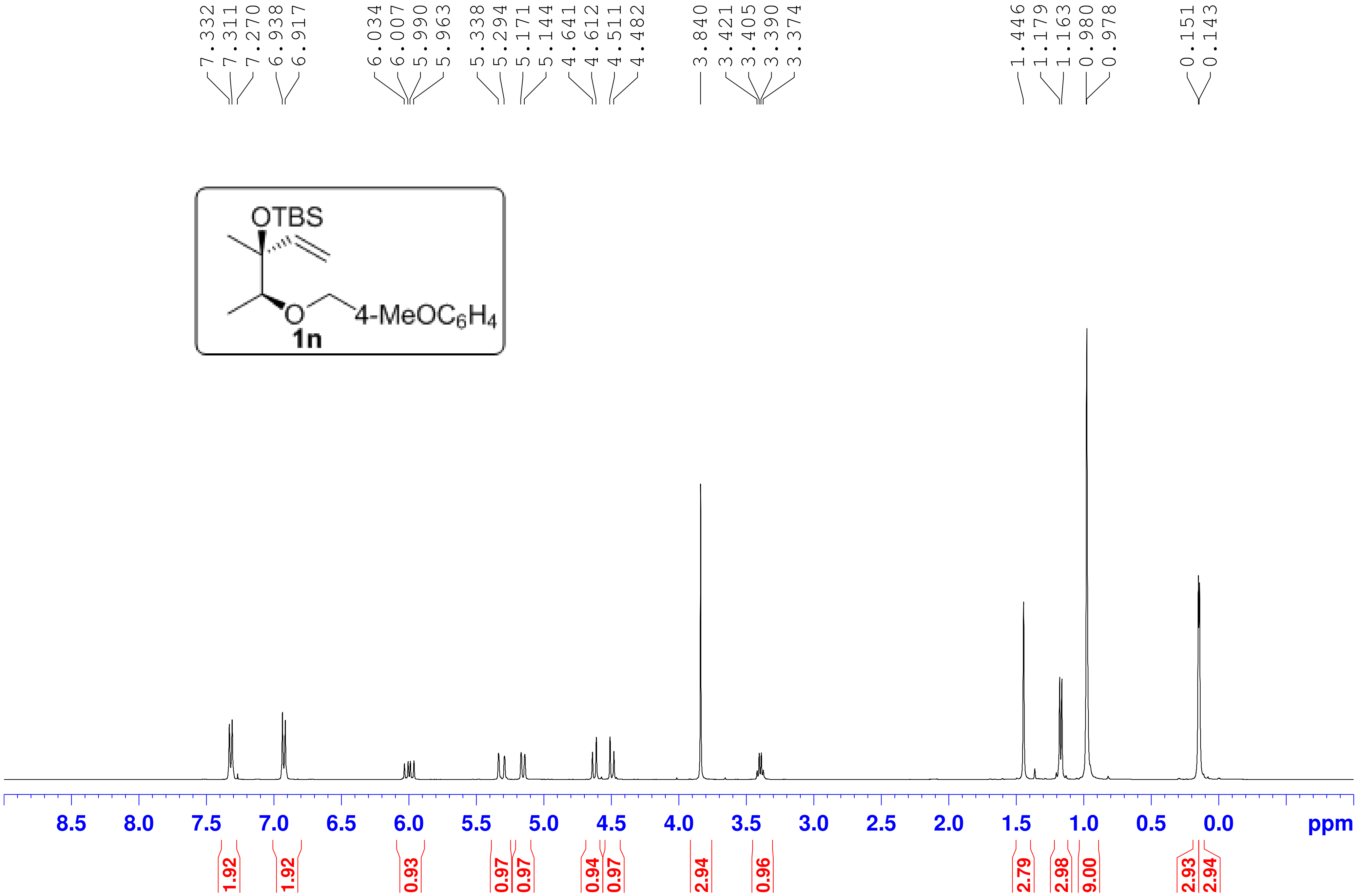

Supplementary Figure 104. ^{13}C NMR Spectrum of 1n (100 MHz, $CDCl_3$)

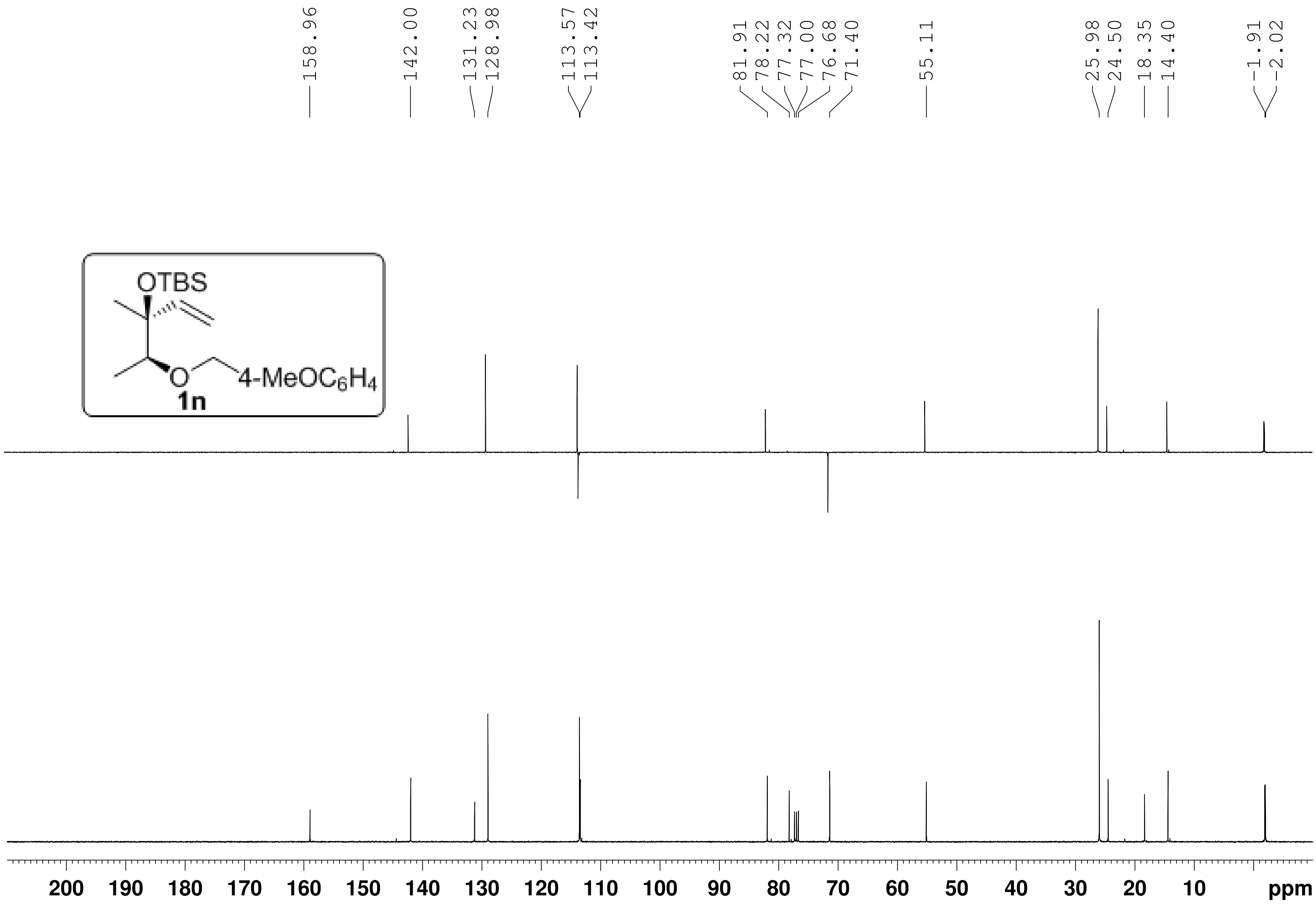

Supplementary Figure 105. ^{1}H NMR Spectrum of 2n (400 MHz, $CDCl_3$)

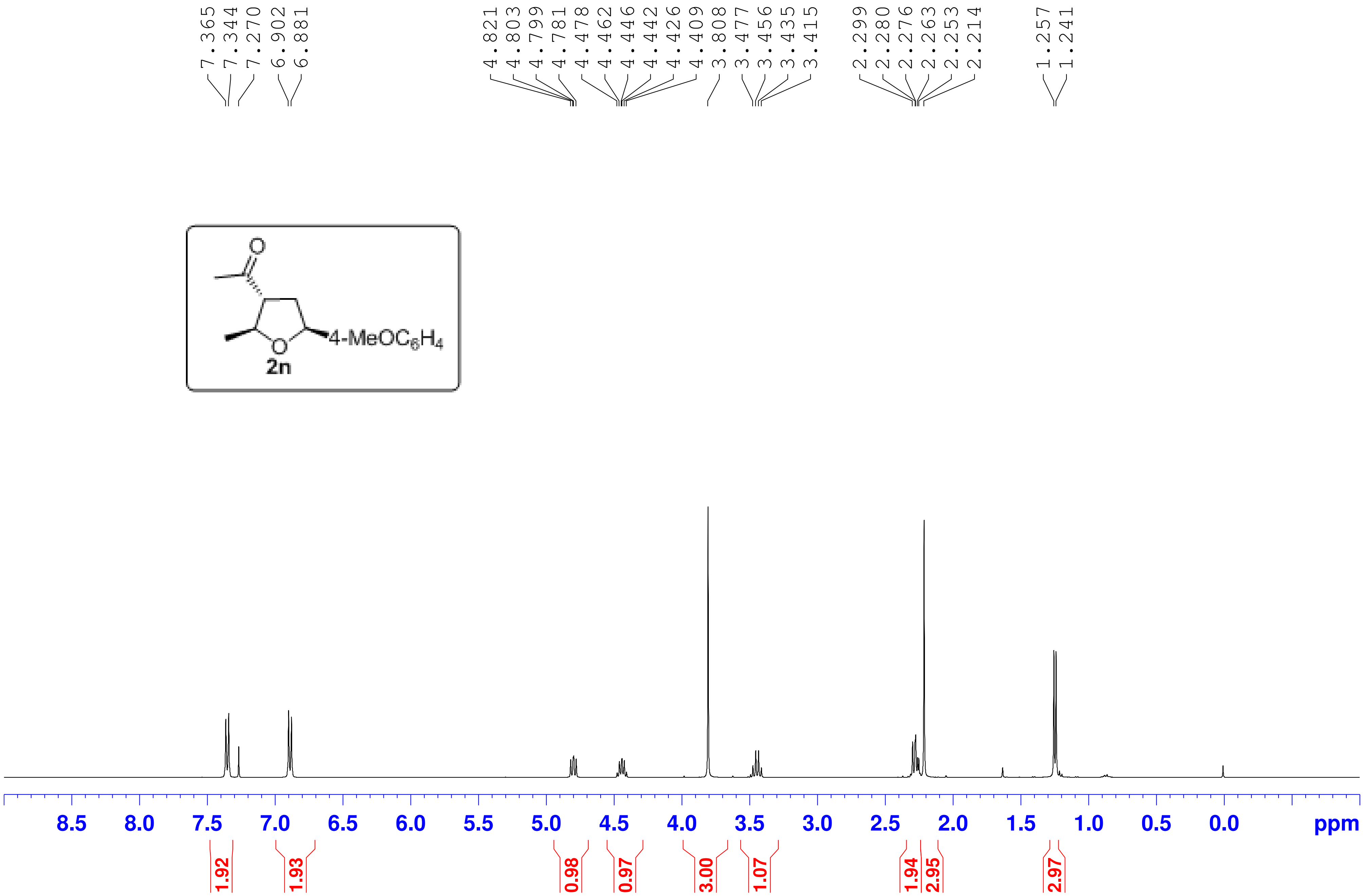

Supplementary Figure 106. ^{13}C NMR Spectrum of 2n (100 MHz, $CDCl_3$)

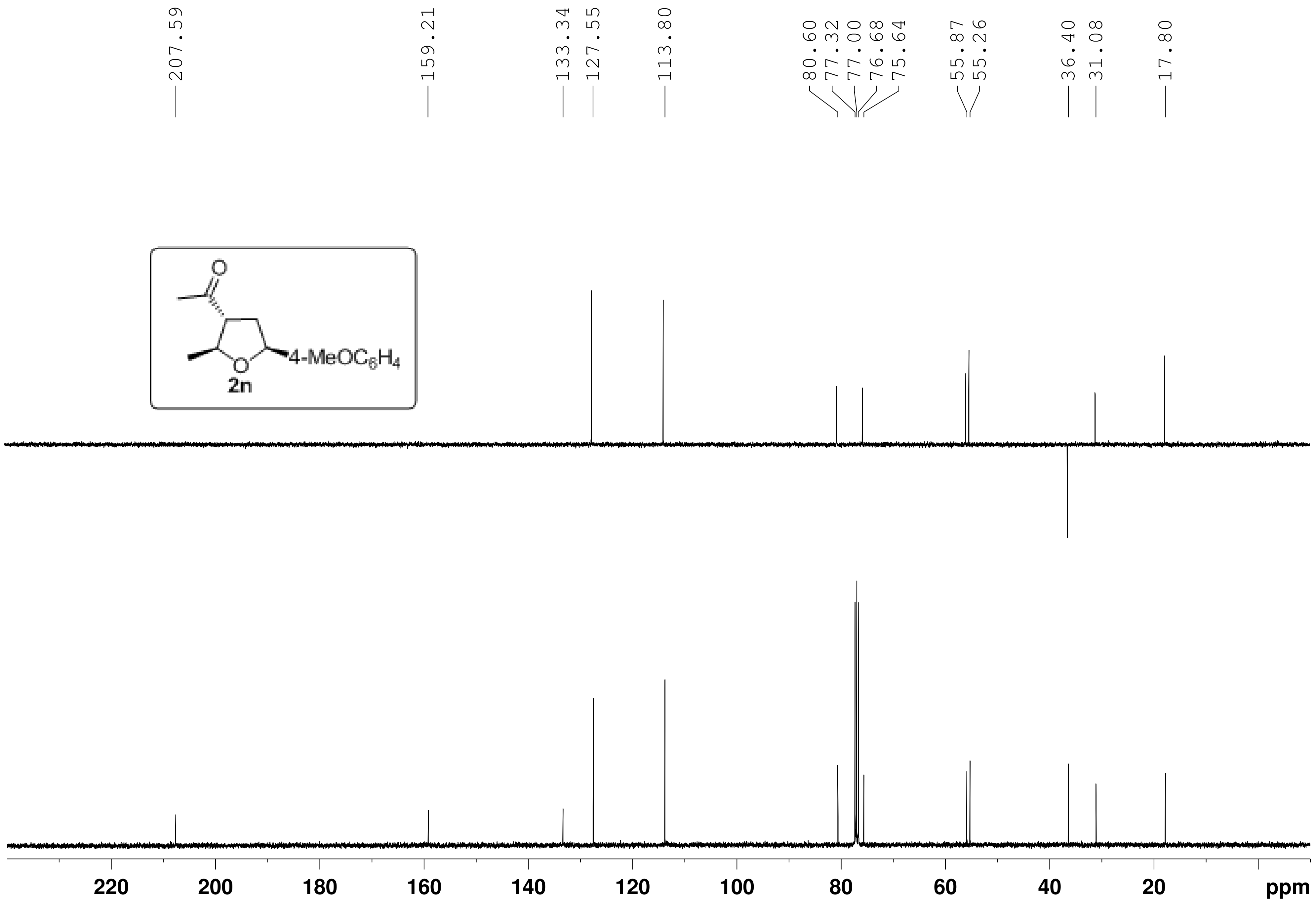

Supplementary Figure 107. ^{1}H NMR Spectrum of S31 (400 MHz, $CDCl_3$)

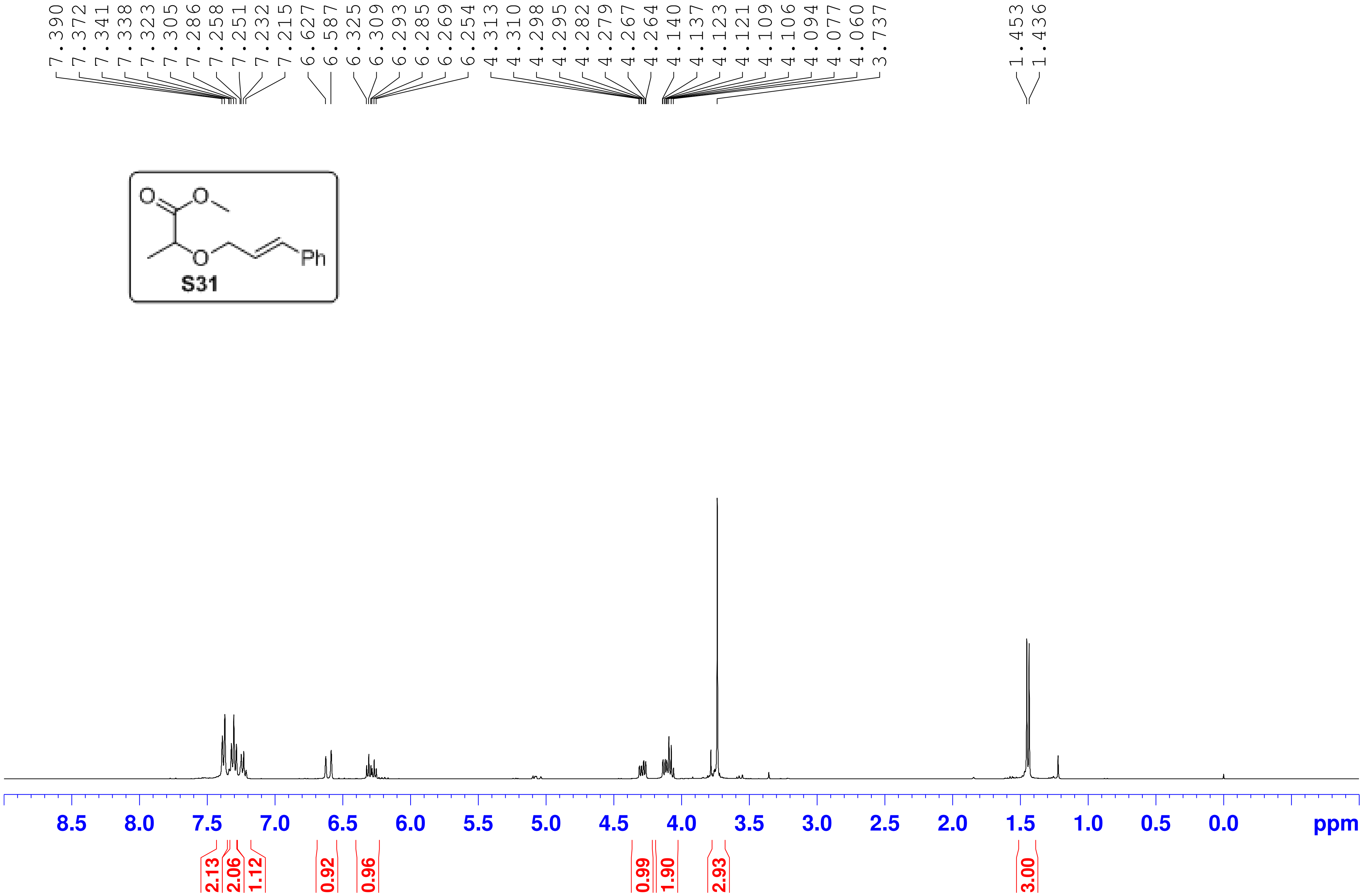

Supplementary Figure 108. ^{13}C NMR Spectrum of S31 (100 MHz, $CDCl_3$)

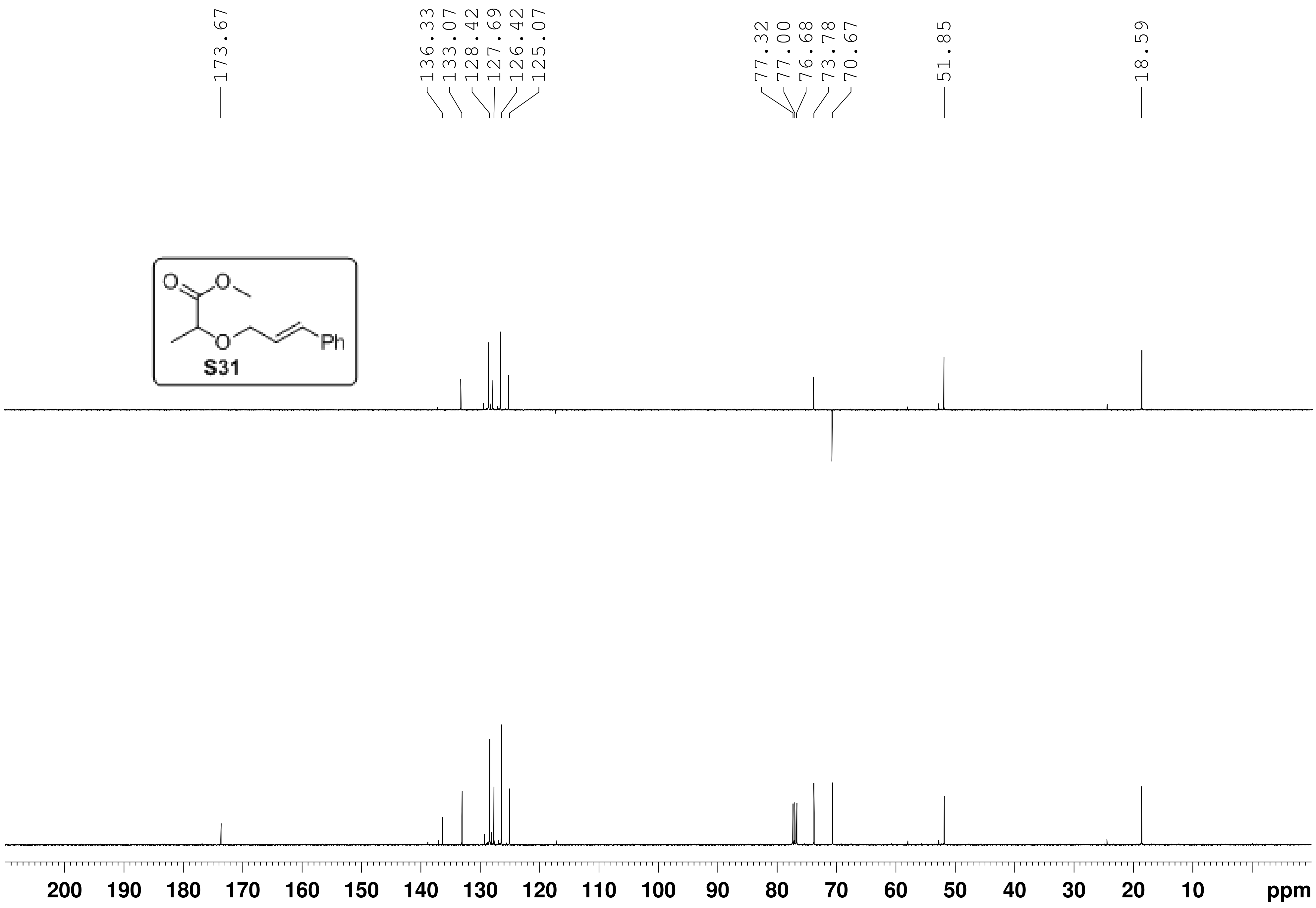

Supplementary Figure 109. ^{1}H NMR Spectrum of S32 (400 MHz, $CDCl_3$)

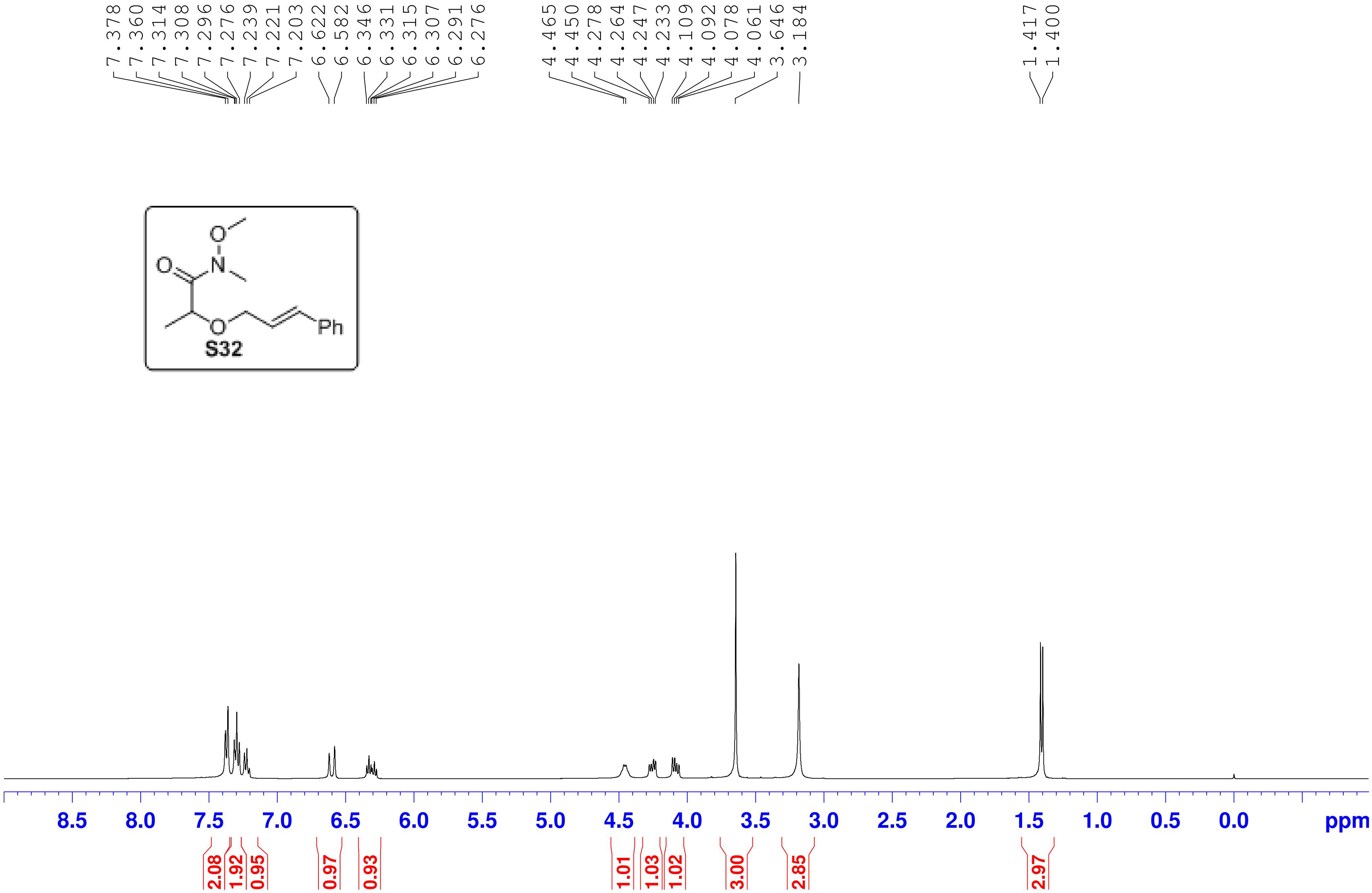

Supplementary Figure 110. ^{13}C NMR Spectrum of S32 (100 MHz, $CDCl_3$)

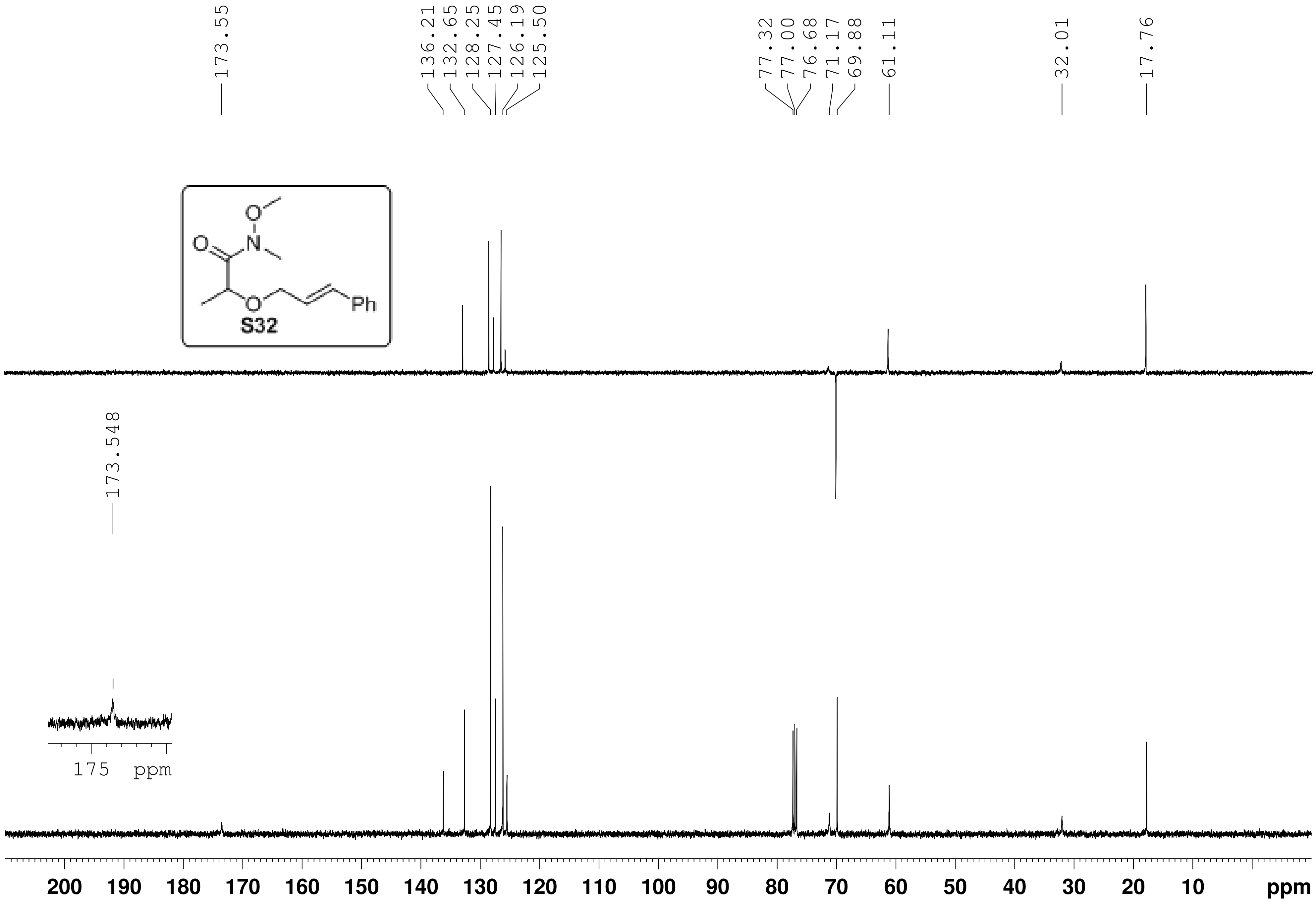

Supplementary Figure 111. ^{1}H **NMR Spectrum of S33 (400 MHz, $CDCl_3$)**

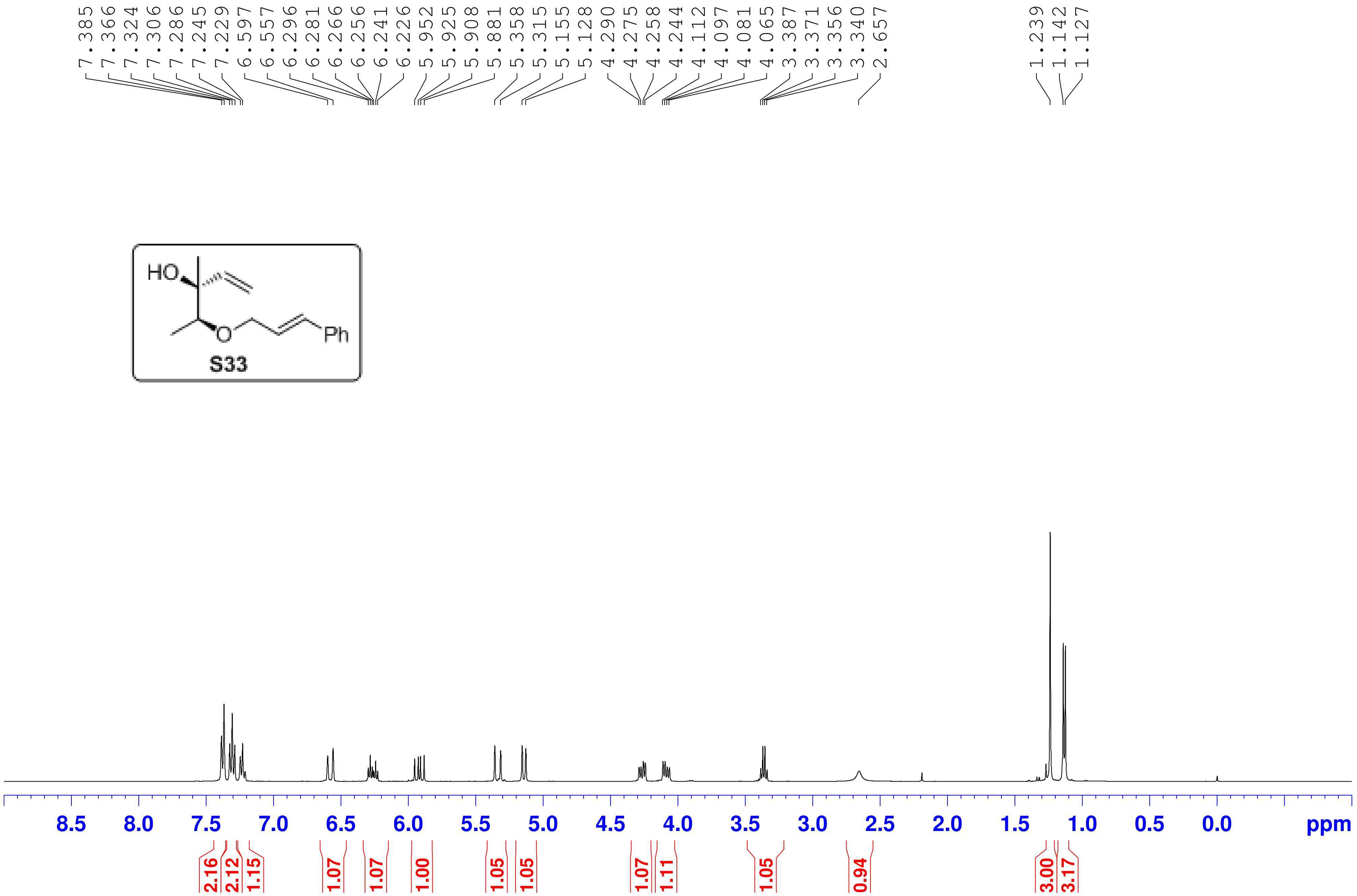

Supplementary Figure 112. ^{13}C NMR Spectrum of S33 (100 MHz, $CDCl_3$)

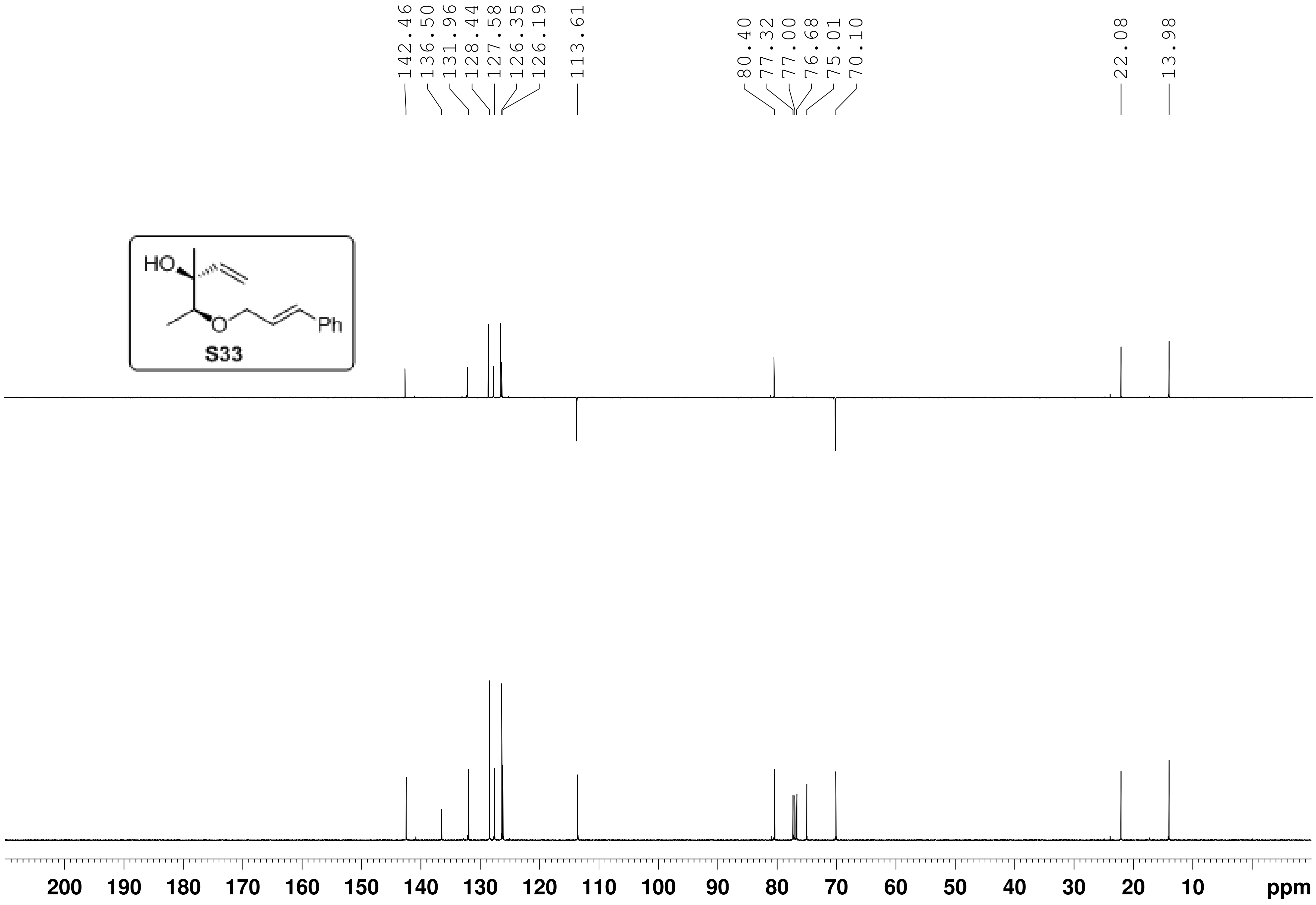

Supplementary Figure 113. ^{1}H **NMR Spectrum of 1o (400 MHz, $CDCl_3$)**

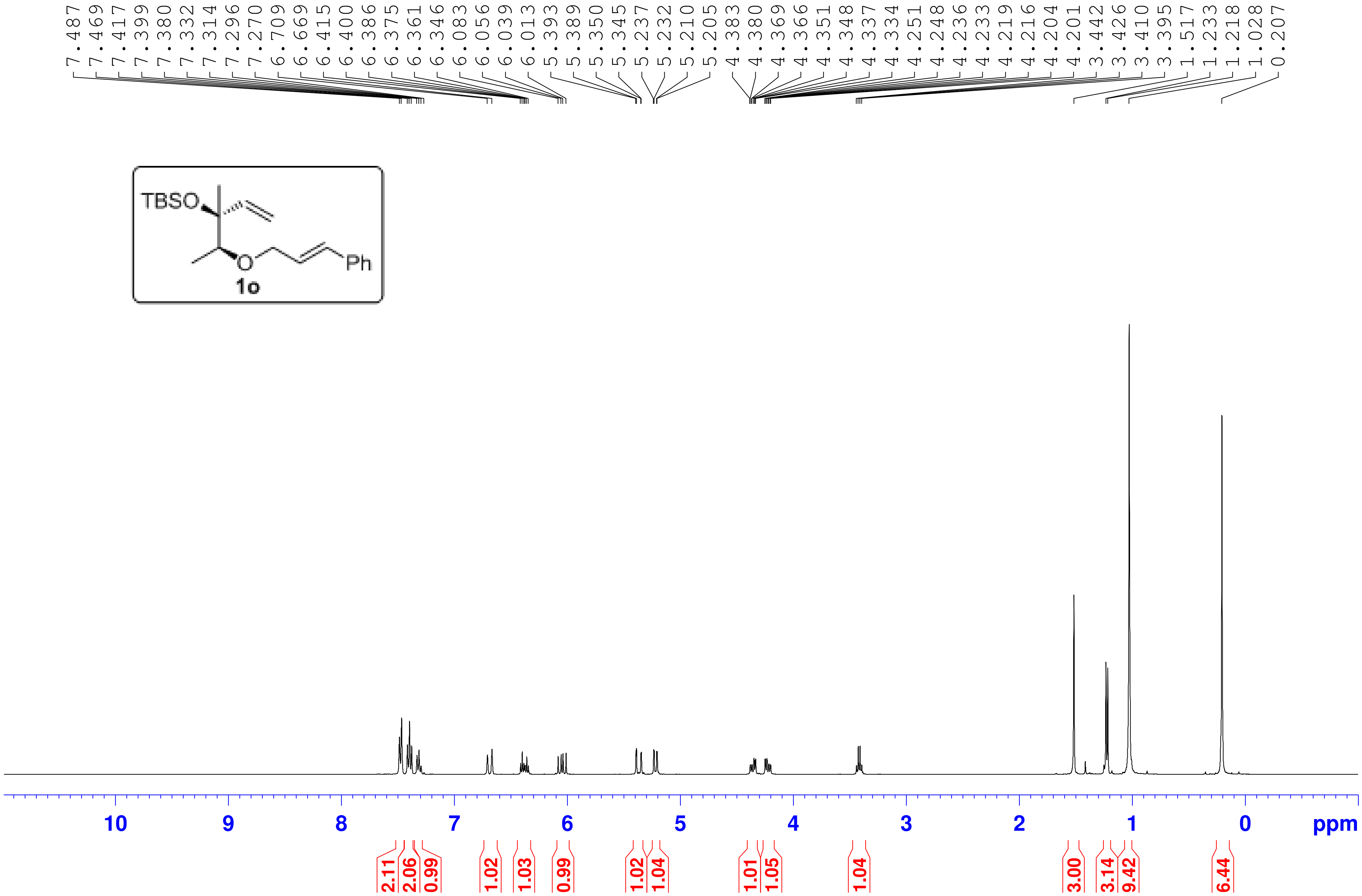

Supplementary Figure 114. ^{13}C NMR Spectrum of 1o (100 MHz, $CDCl_3$)

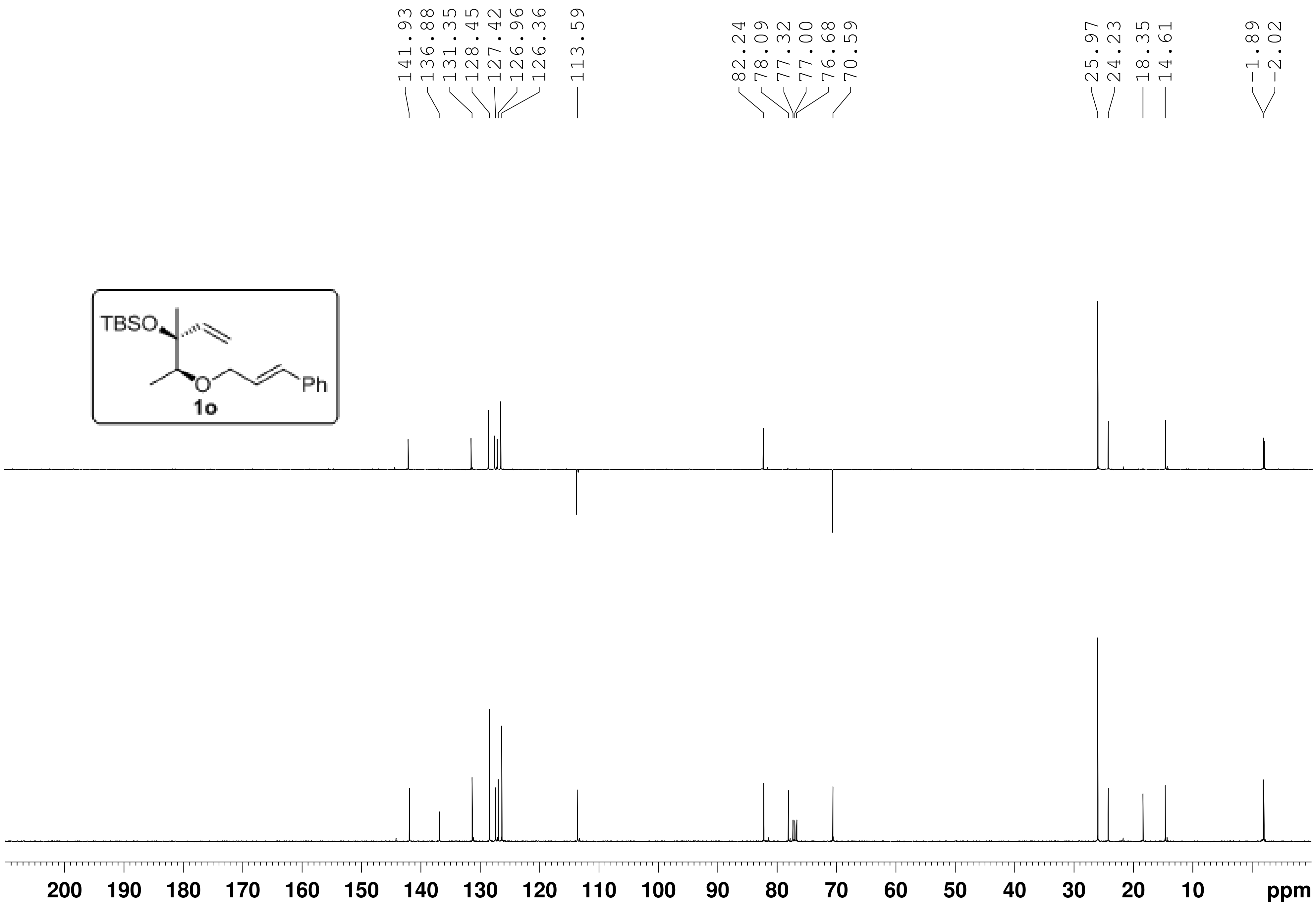

Supplementary Figure 115. ^{1}H NMR Spectrum of 2o (400 MHz, $CDCl_3$)

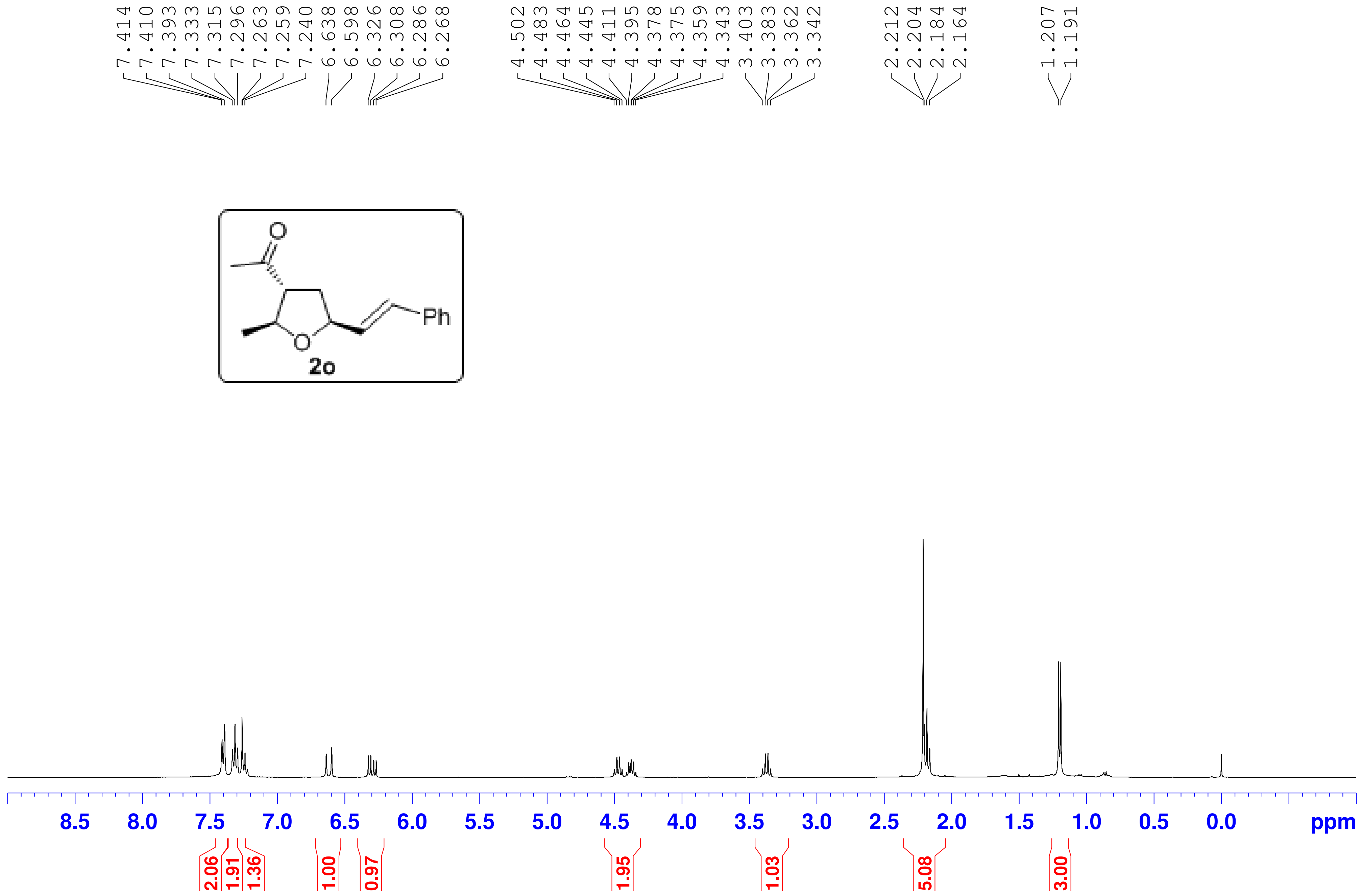

Supplementary Figure 116. ^{13}C NMR Spectrum of 2o (100 MHz, $CDCl_3$)

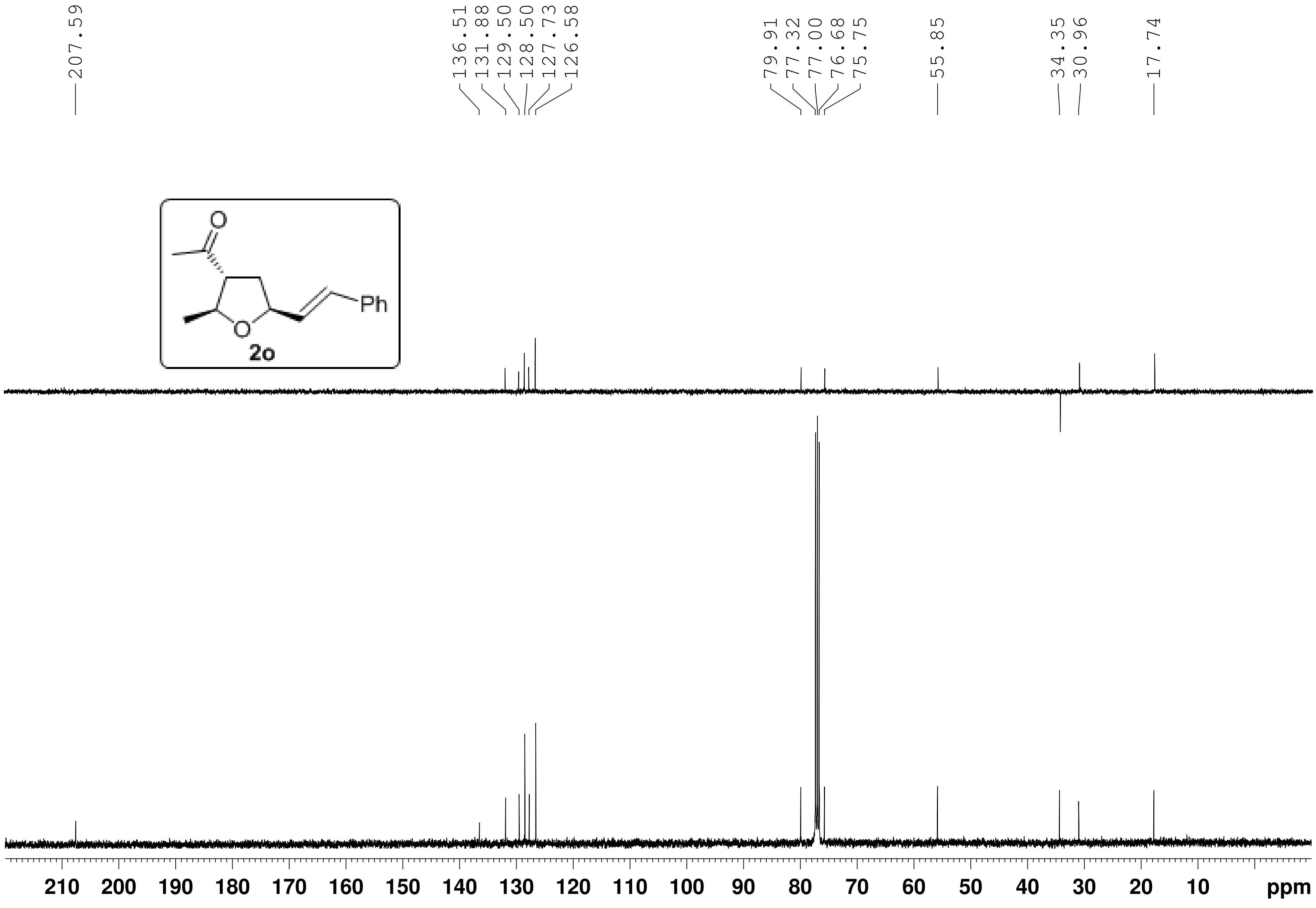

Supplementary Figure 117. ^{1}H **NMR Spectrum of S35 (400 MHz, $CDCl_3$)**

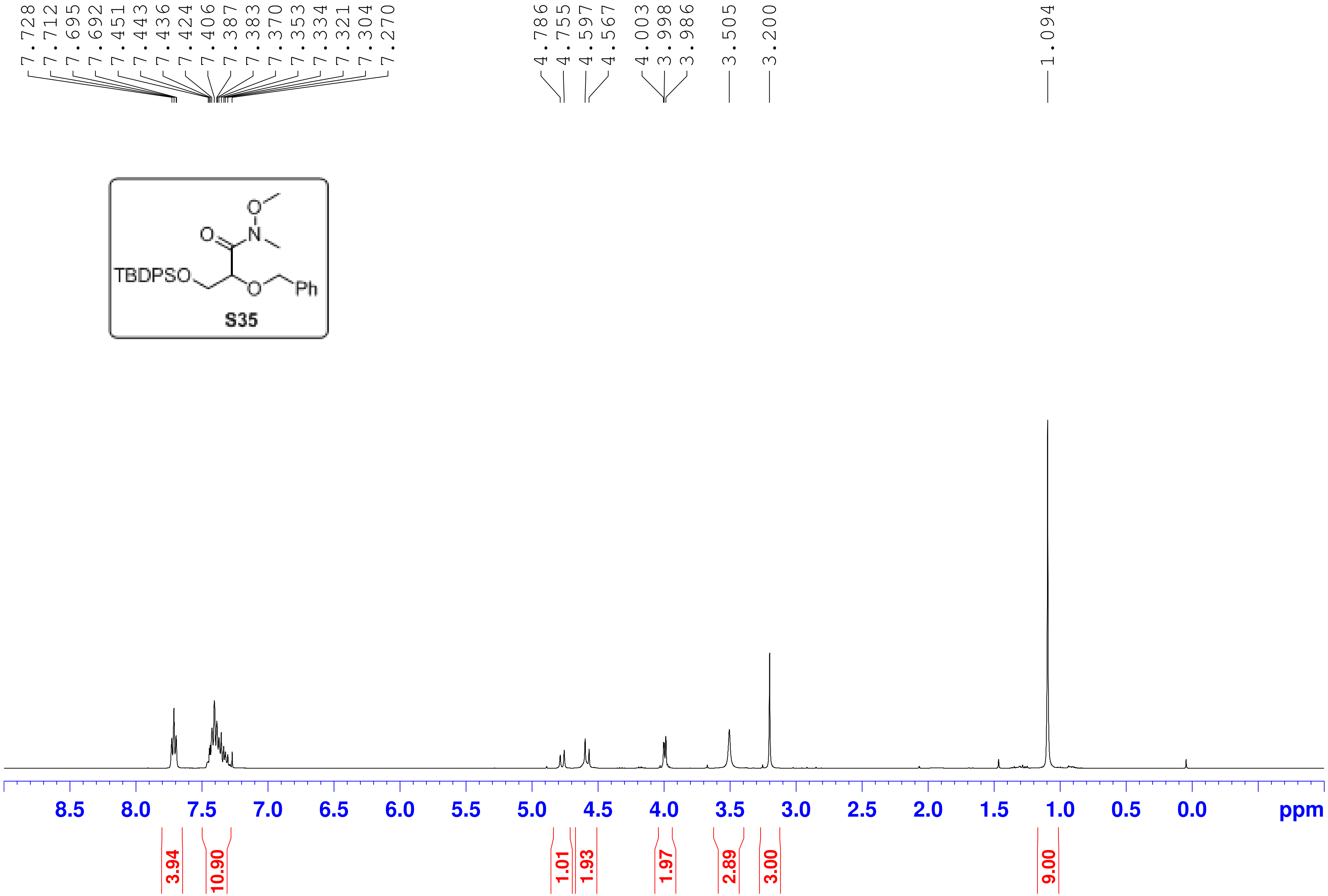

Supplementary Figure 118. ^{13}C NMR Spectrum of S35 (100 MHz, $CDCl_3$)

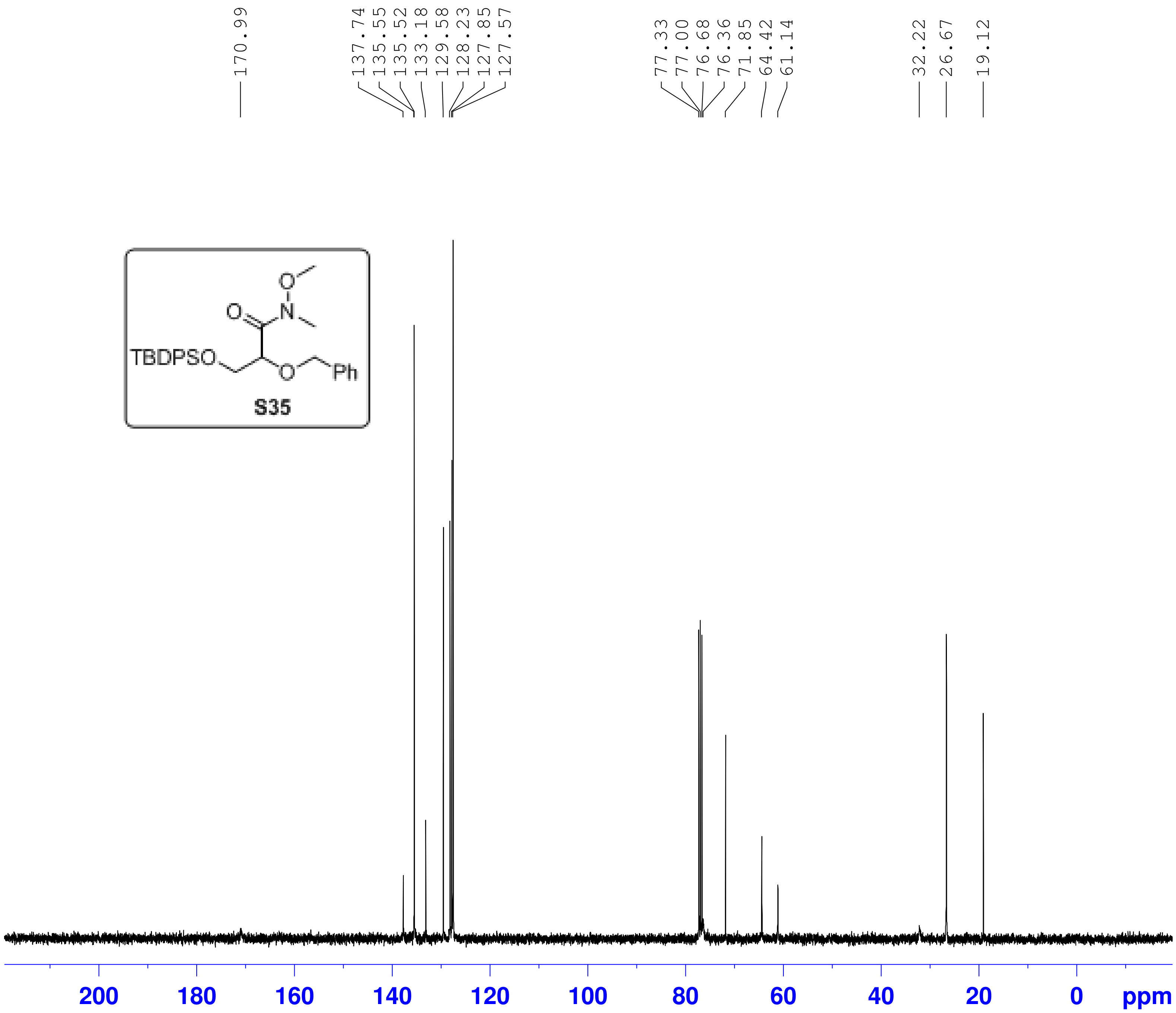

NAME	jiaozhiweiB72
EXPNO	11
PROCNO	1
Date_	20120717
Time	10.13
INSTRUM	spect
PROBHD	5 mm PABBO BB-
PULPROG	zgpg30
TD	65536
SOLVENT	CDCl3
NS	80
DS	4
SWH	24038.461 Hz
FIDRES	0.366798 Hz
AQ	1.3631988 sec
RG	2050
DW	20.800 usec
DE	6.50 usec
TE	297.7 K
D1	2.00000000 sec
D11	0.03000000 sec
TD0	1

======== CHANNEL f1 ========

NUC1	13C
P1	9.40 usec
PL1	-2.00 dB
PL1W	57.32743073 W
SFO1	100.6228298 MHz

======== CHANNEL f2 ========

CPDPRG2	waltz16
NUC2	1H
PCPD2	90.00 usec
PL2	-2.00 dB
PL12	15.50 dB
PL13	15.50 dB
PL2W	18.19349861 W
PL12W	0.32353121 W
PL13W	0.32353121 W
SFO2	400.1316005 MHz
SI	32768
SF	100.6127817 MHz
WDW	EM
SSB	0
LB	1.00 Hz
GB	0
PC	1.40

Supplementary Figure 119. ^{1}H NMR Spectrum of S36 (400 MHz, $CDCl_3$)

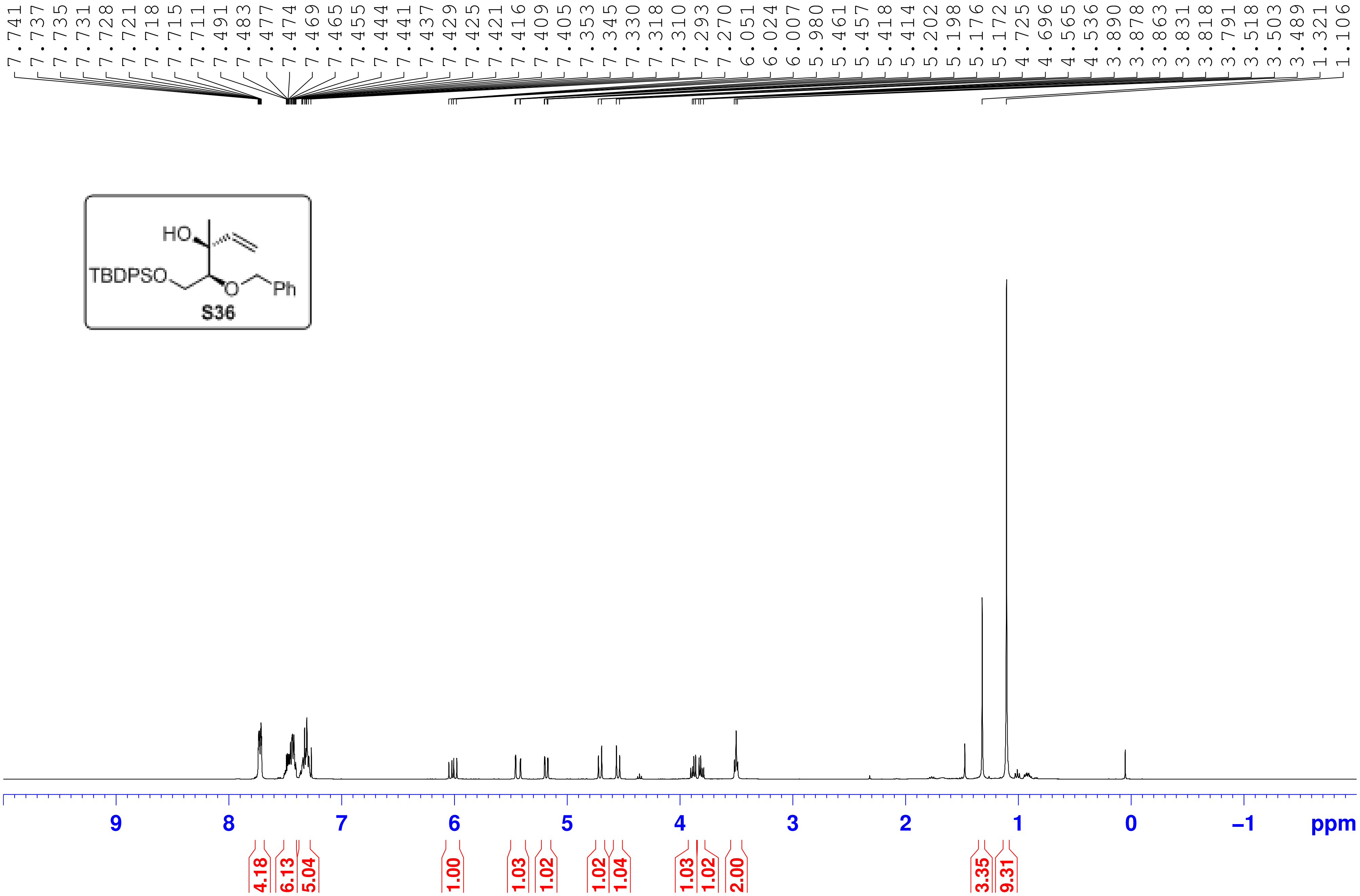

Supplementary Figure 120. ^{13}C NMR Spectrum of S36 (100 MHz, $CDCl_3$)

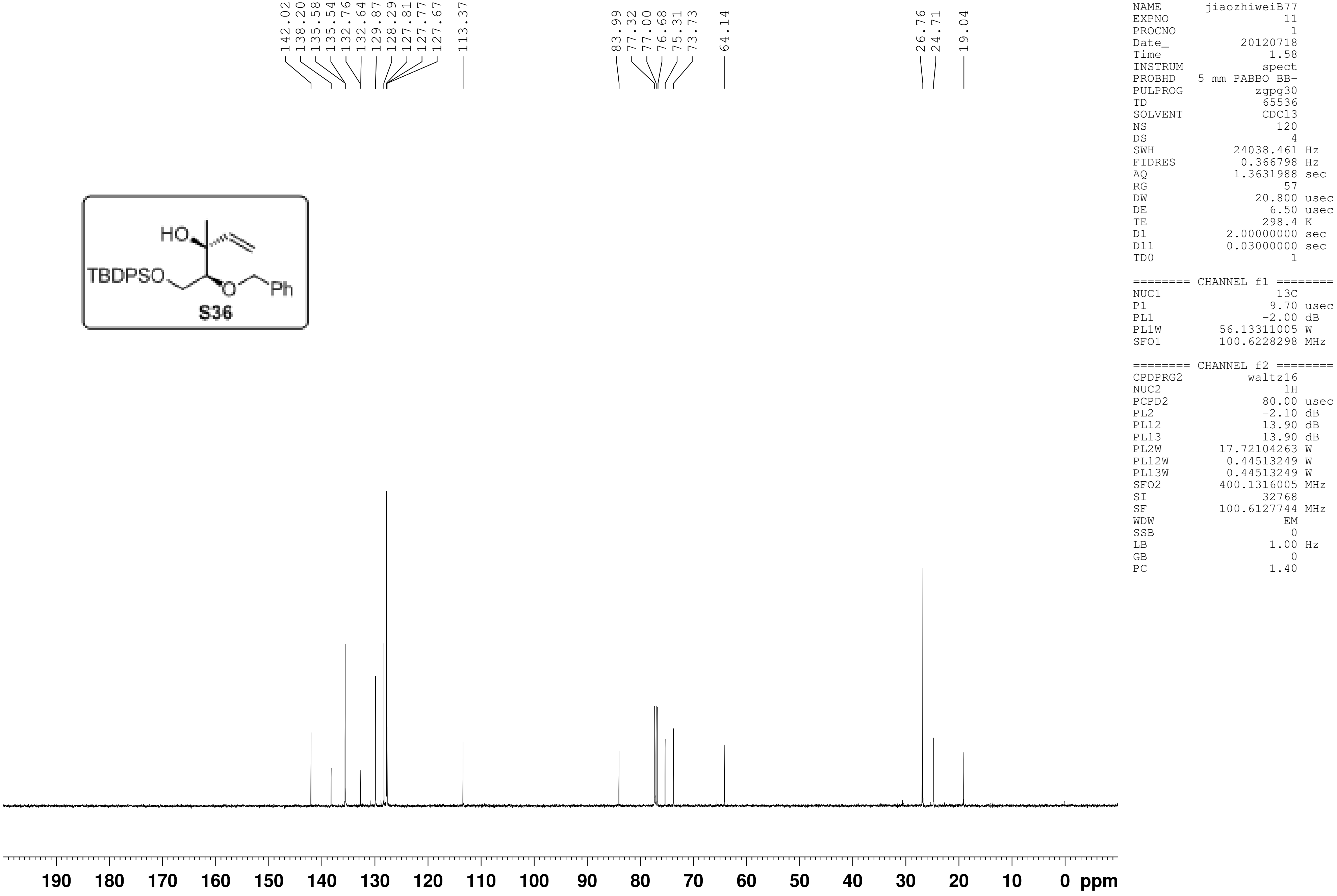

Supplementary Figure 121. ^1H NMR Spectrum of 1p (400 MHz, $CDCl_3$)

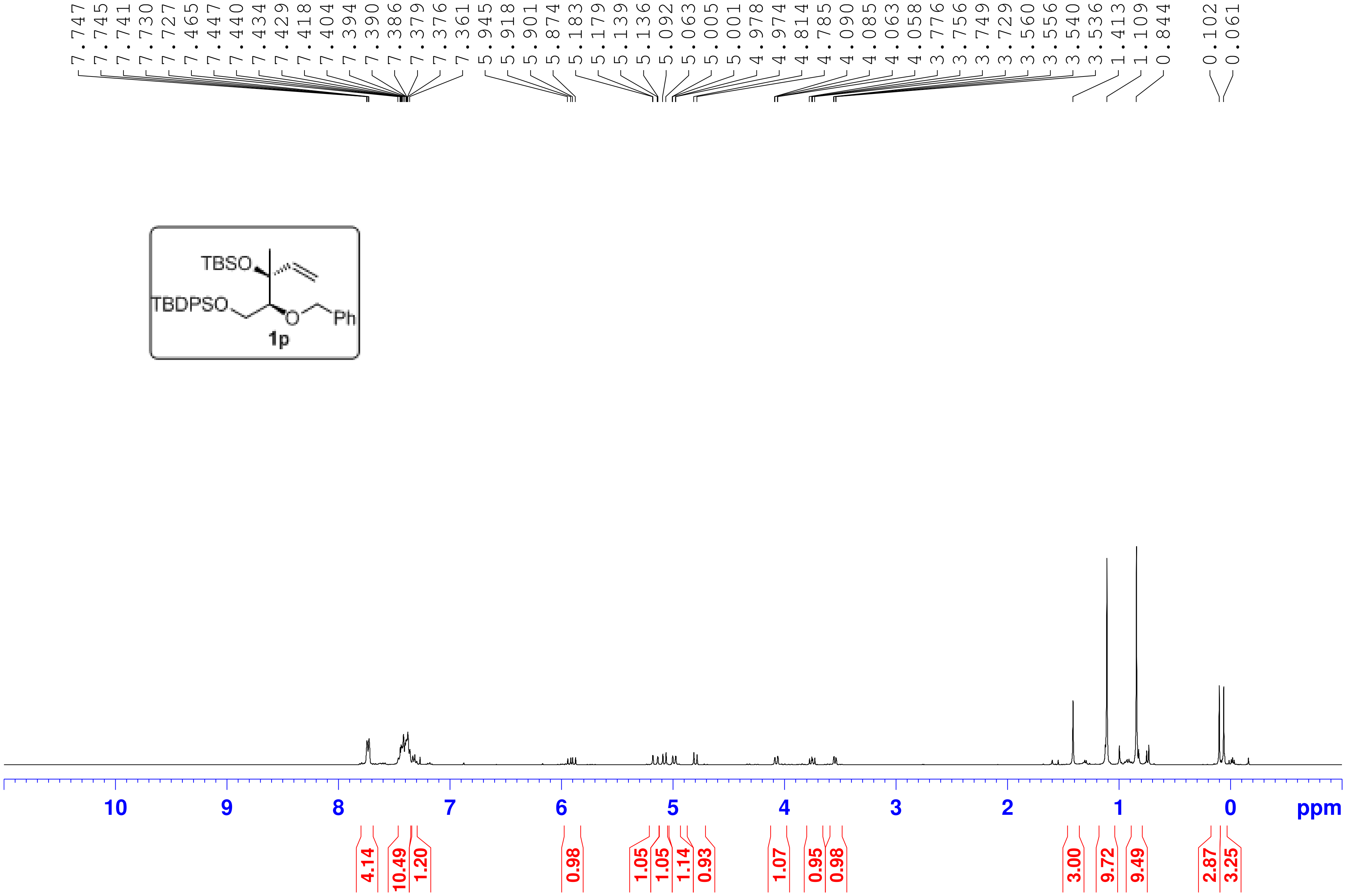

Supplementary Figure 122. ^{13}C NMR Spectrum of 1p (100 MHz, $CDCl_3$)

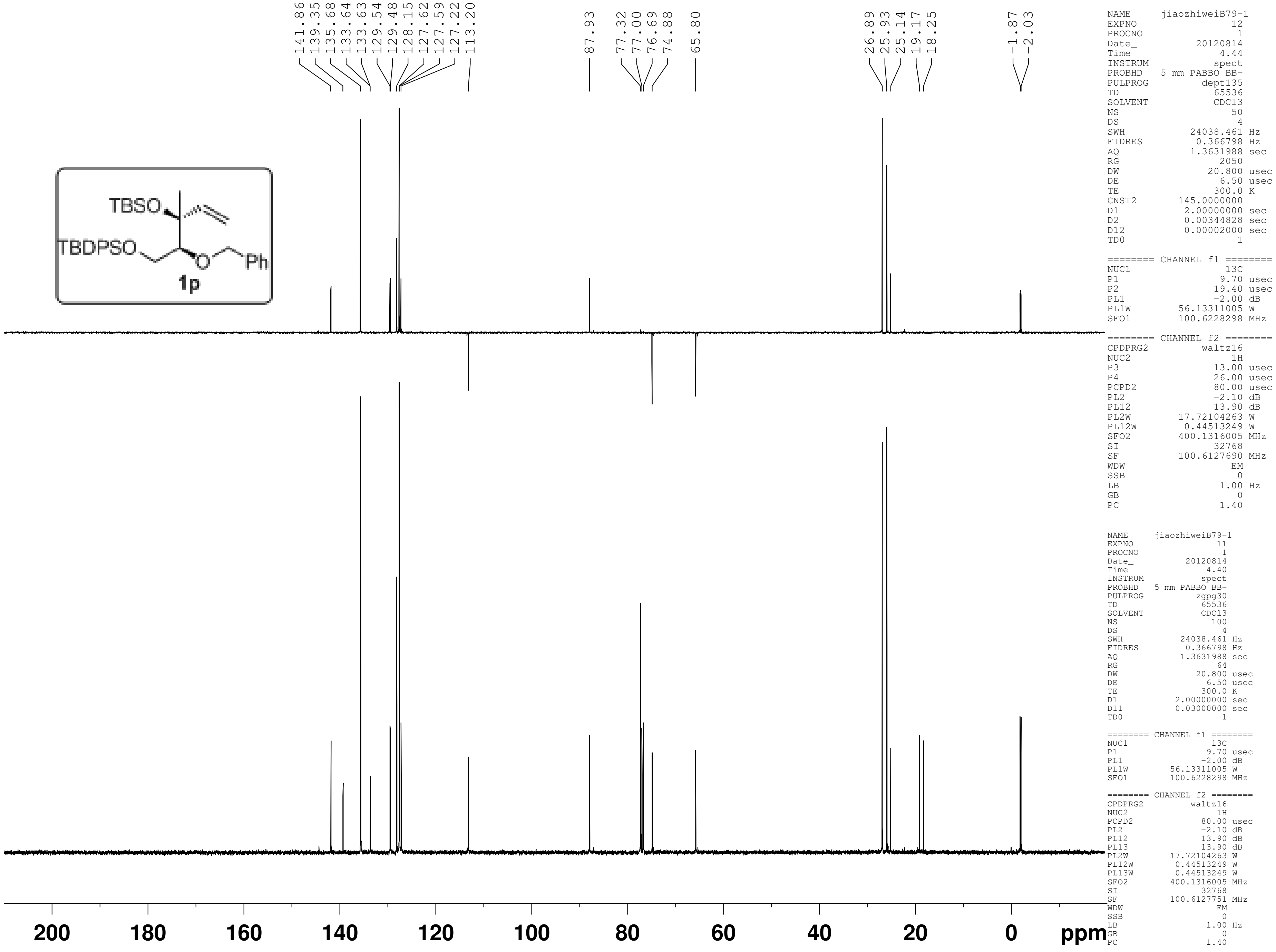

Supplementary Figure 123. ^{1}H NMR Spectrum of 2p (400 MHz, $CDCl_3$)

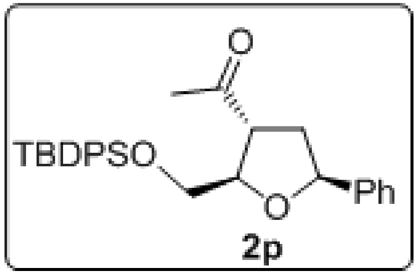

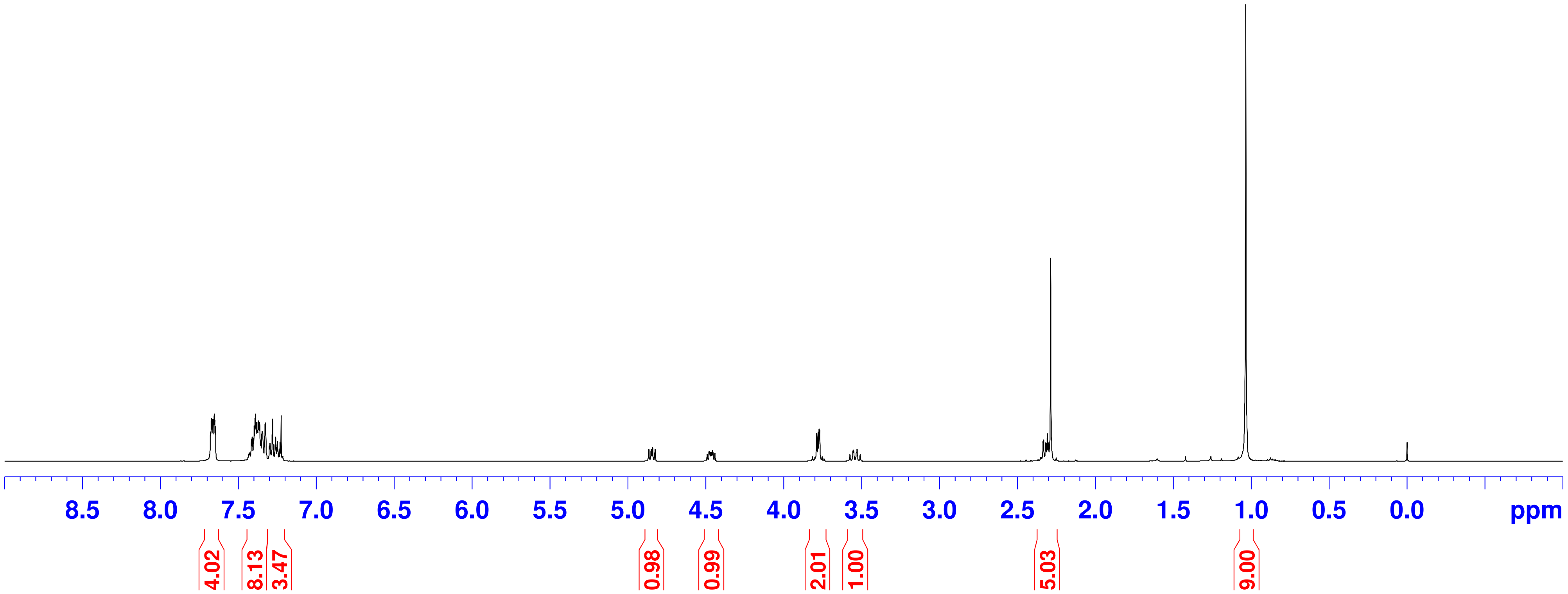

Supplementary Figure 124. ^{13}C NMR Spectrum of 2p (100 MHz, $CDCl_3$)

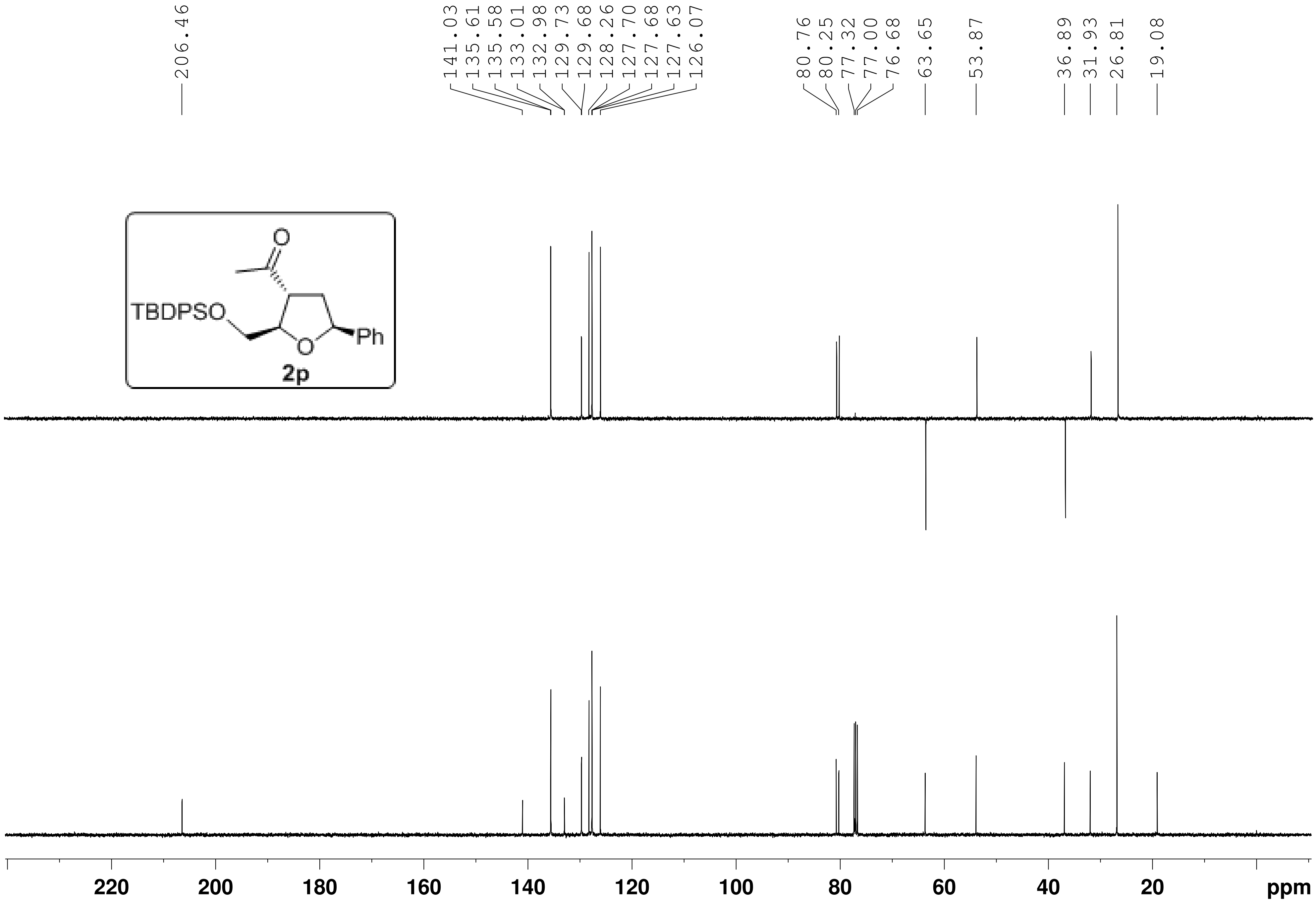

Supplementary Figure 125. ^{1}H NMR Spectrum of 3p (400 MHz, $CDCl_3$)

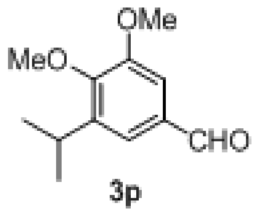

3p

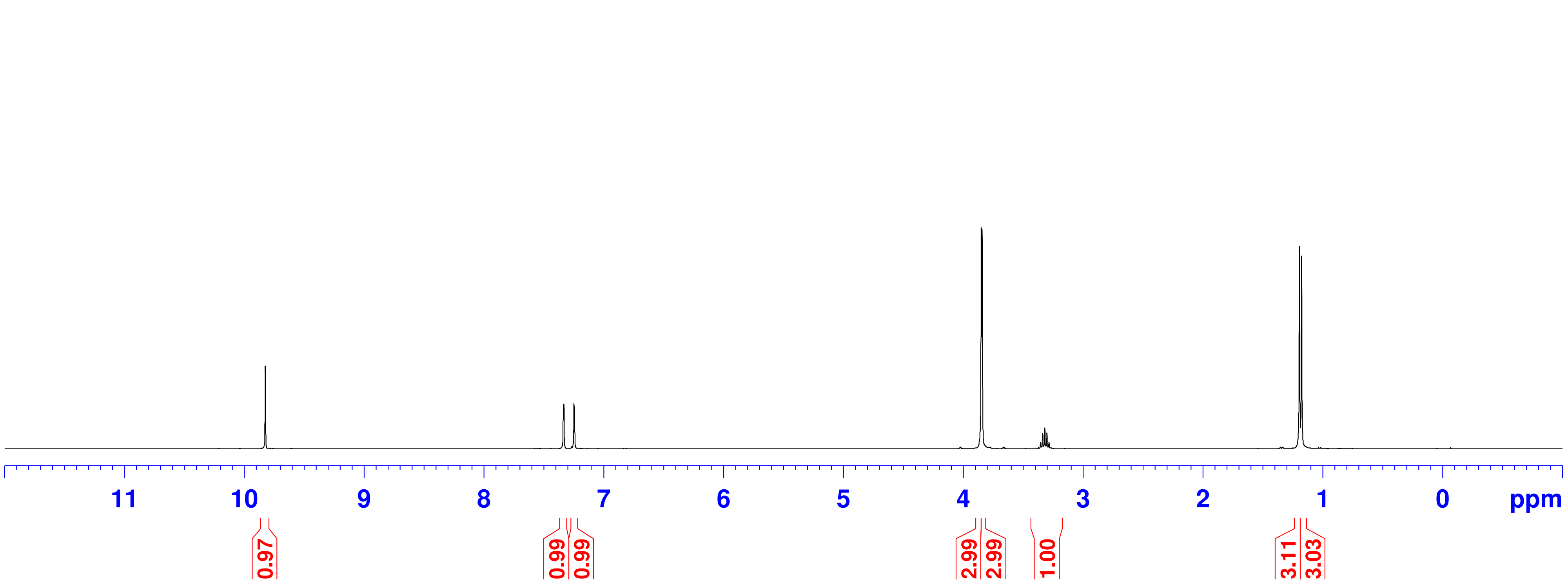

Supplementary Figure 126. ^{13}C NMR Spectrum of 3p (100 MHz, $CDCl_3$)

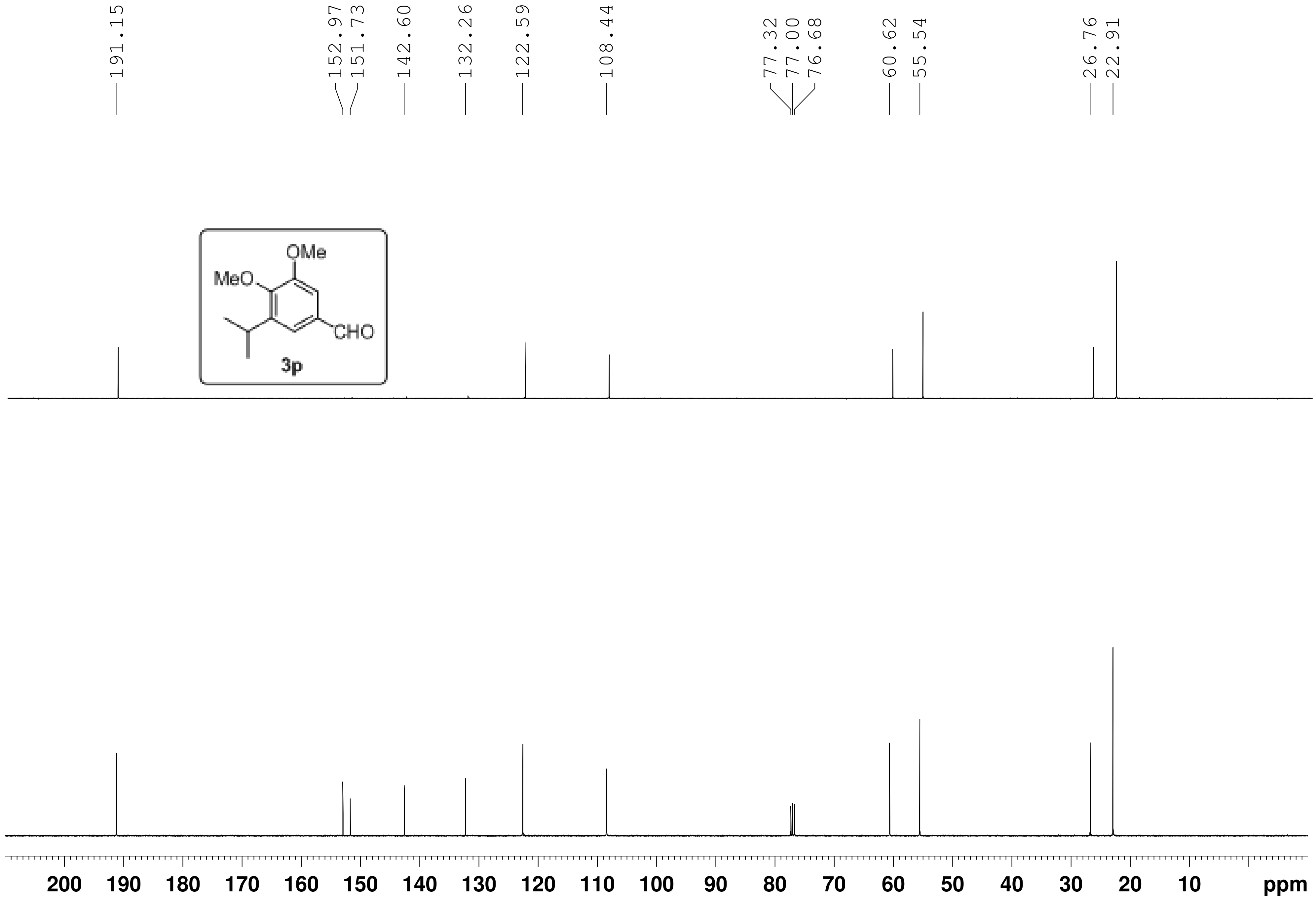

Supplementary Figure 127. ^{1}H NMR Spectrum of 3g (400 MHz, $CDCl_3$)

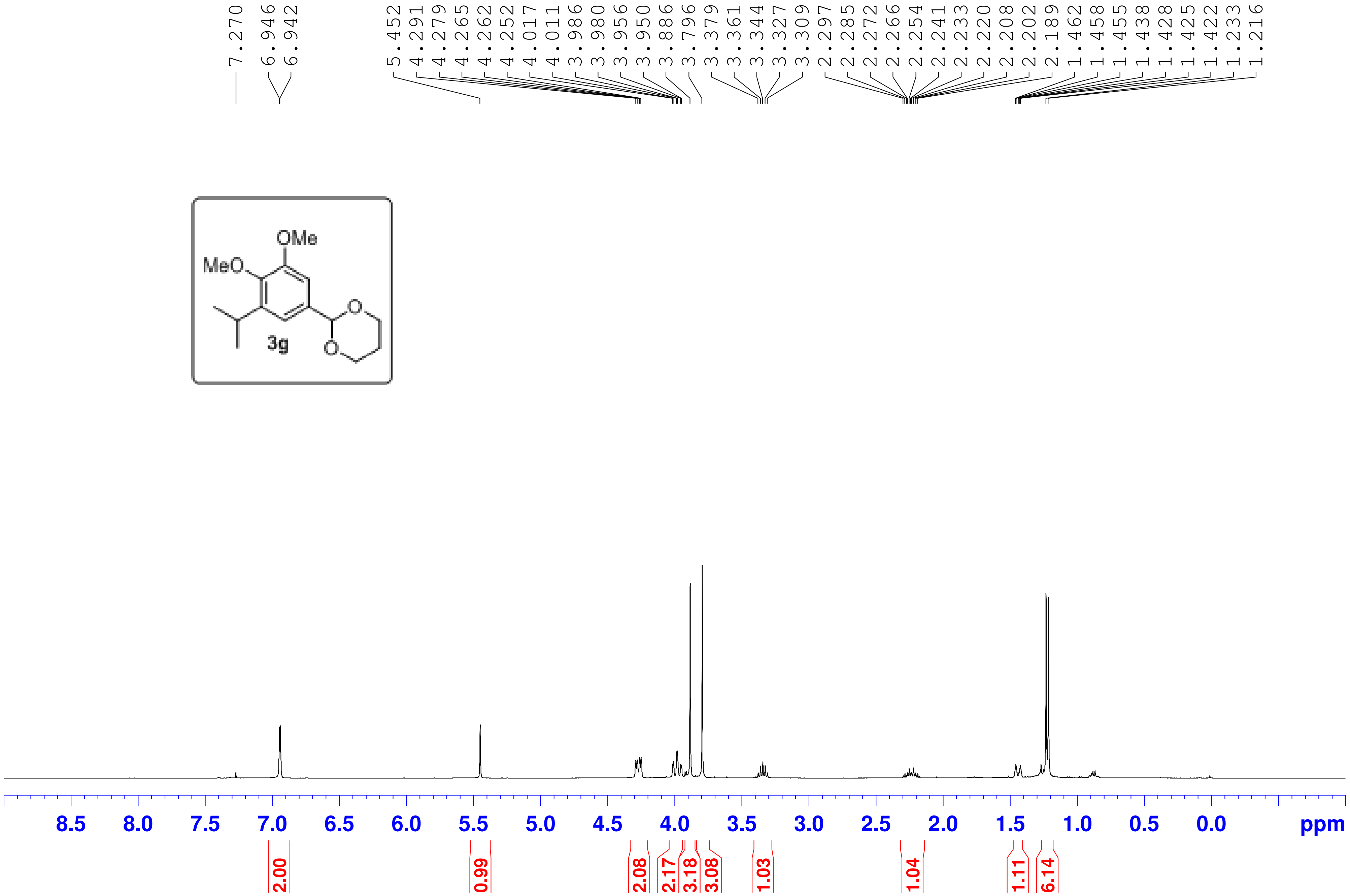

Supplementary Figure 128. ^{13}C NMR Spectrum of 3g (100 MHz, $CDCl_3$)

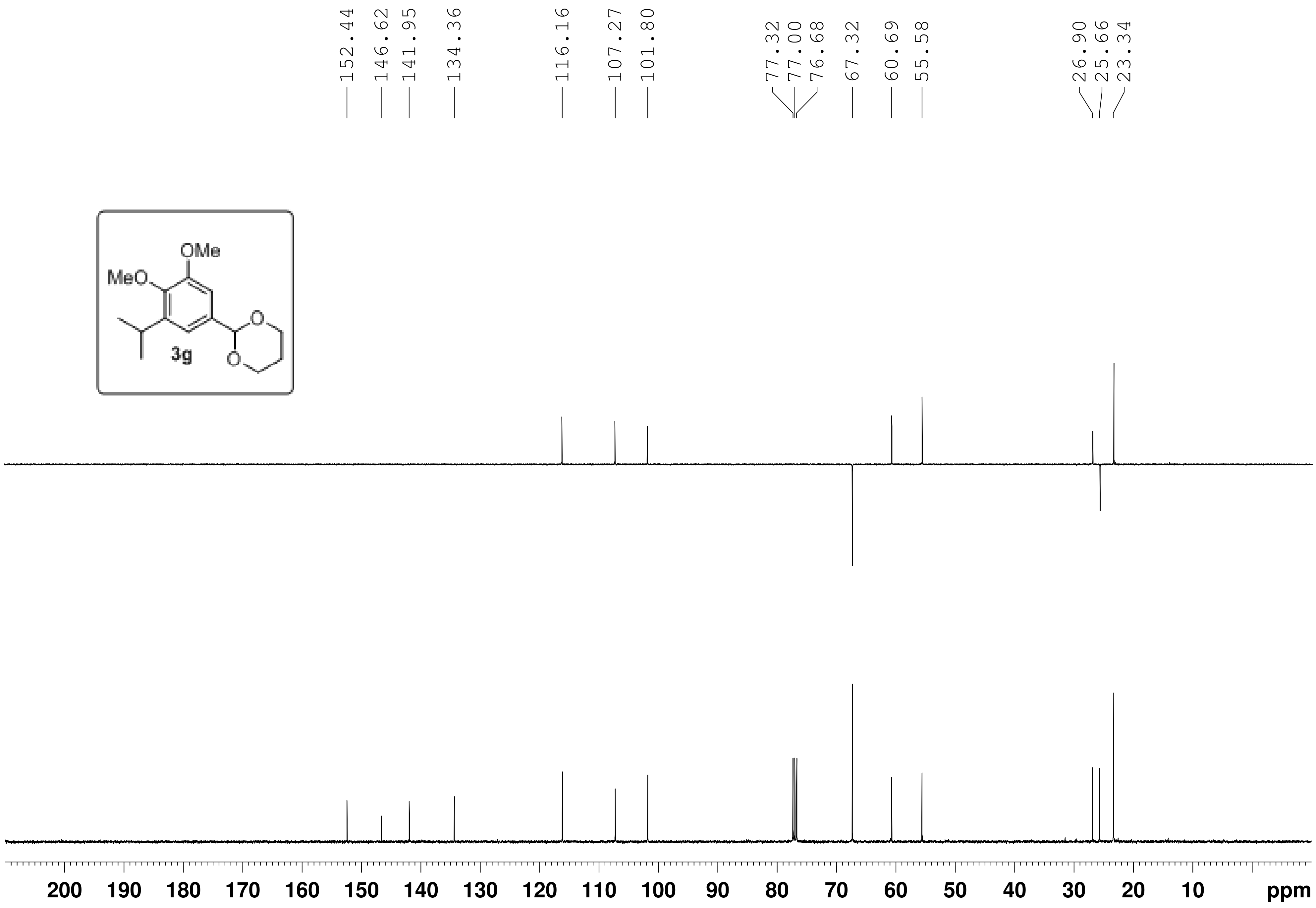

Supplementary Figure 129. ^{1}H NMR Spectrum of 3h (400 MHz, $CDCl_3$)

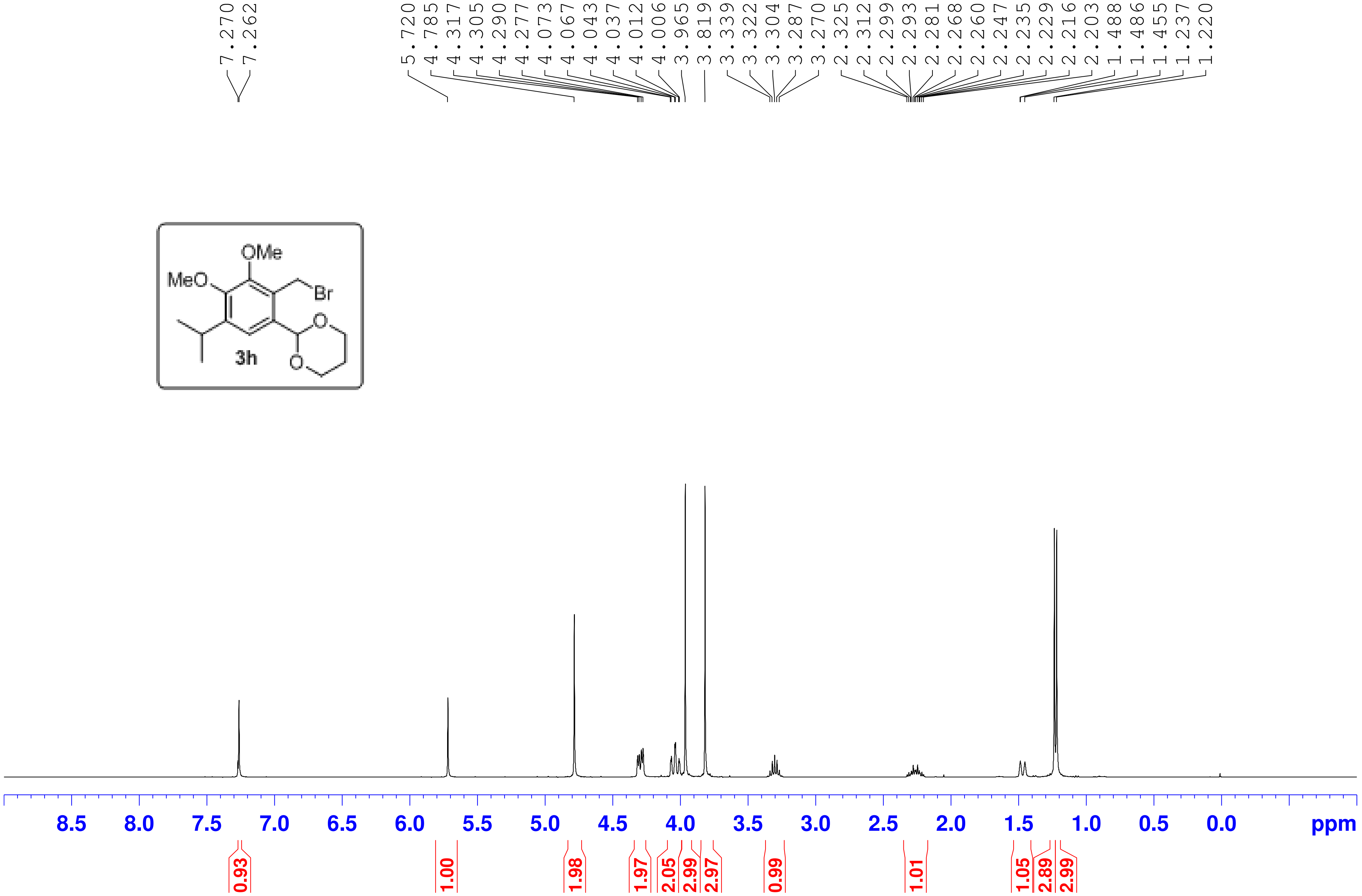

Supplementary Figure 130. ^{13}C NMR Spectrum of 3h (100 MHz, $CDCl_3$)

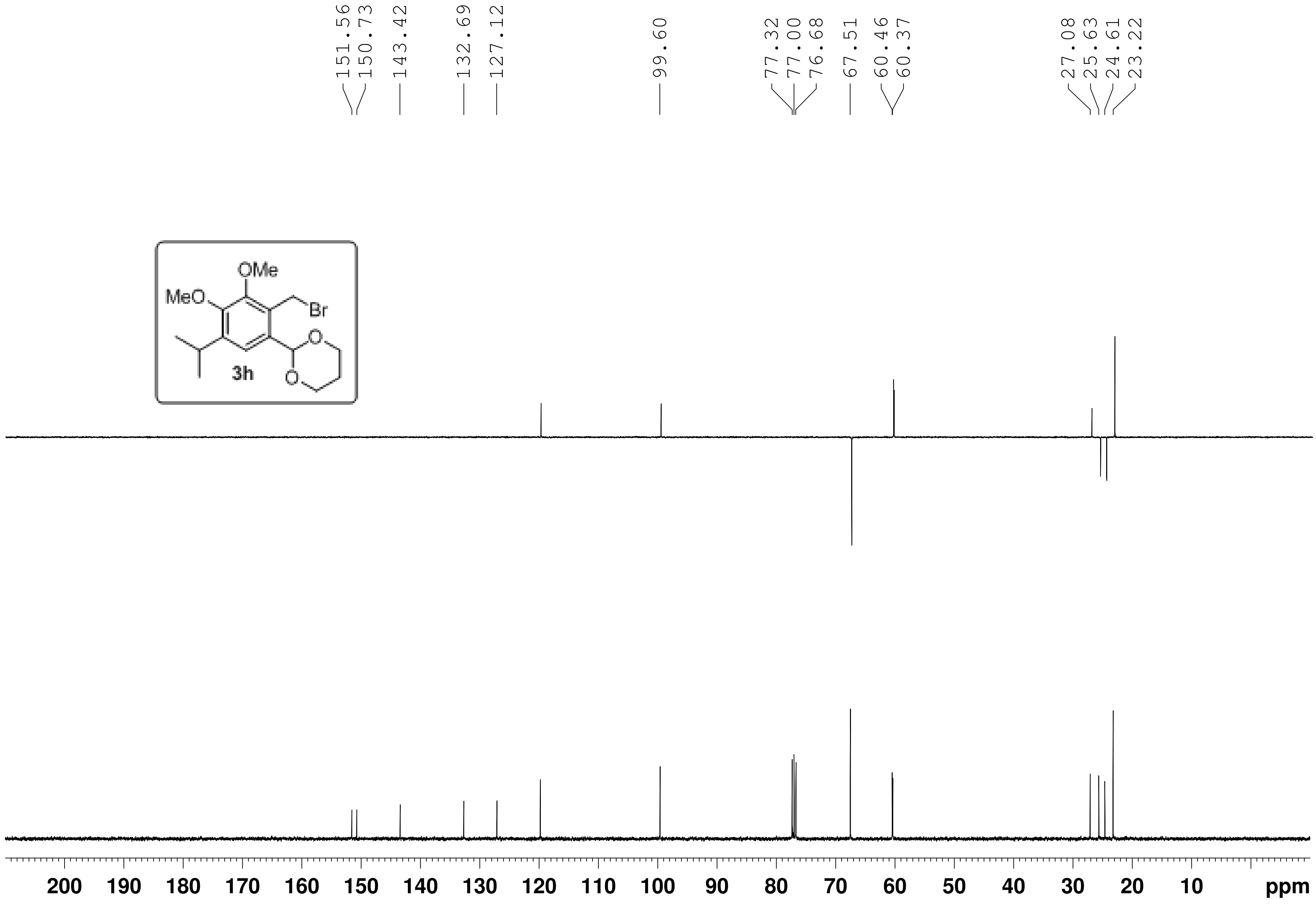

Supplementary Figure 131. ^{1}H NMR Spectrum of 3j (400 MHz, $CDCl_3$)

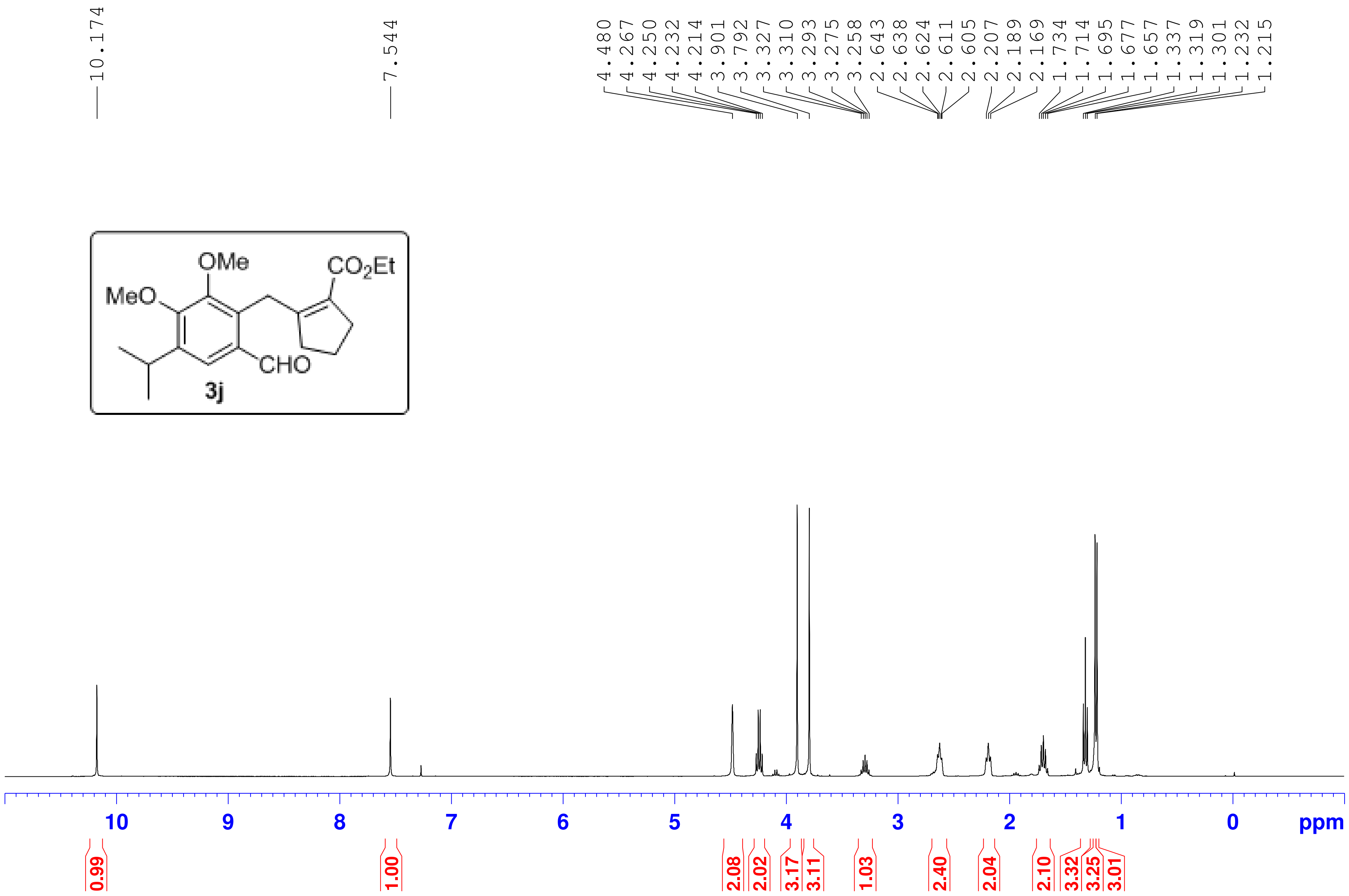

Supplementary Figure 132. ^{13}C NMR Spectrum of 3j (100 MHz, $CDCl_3$)

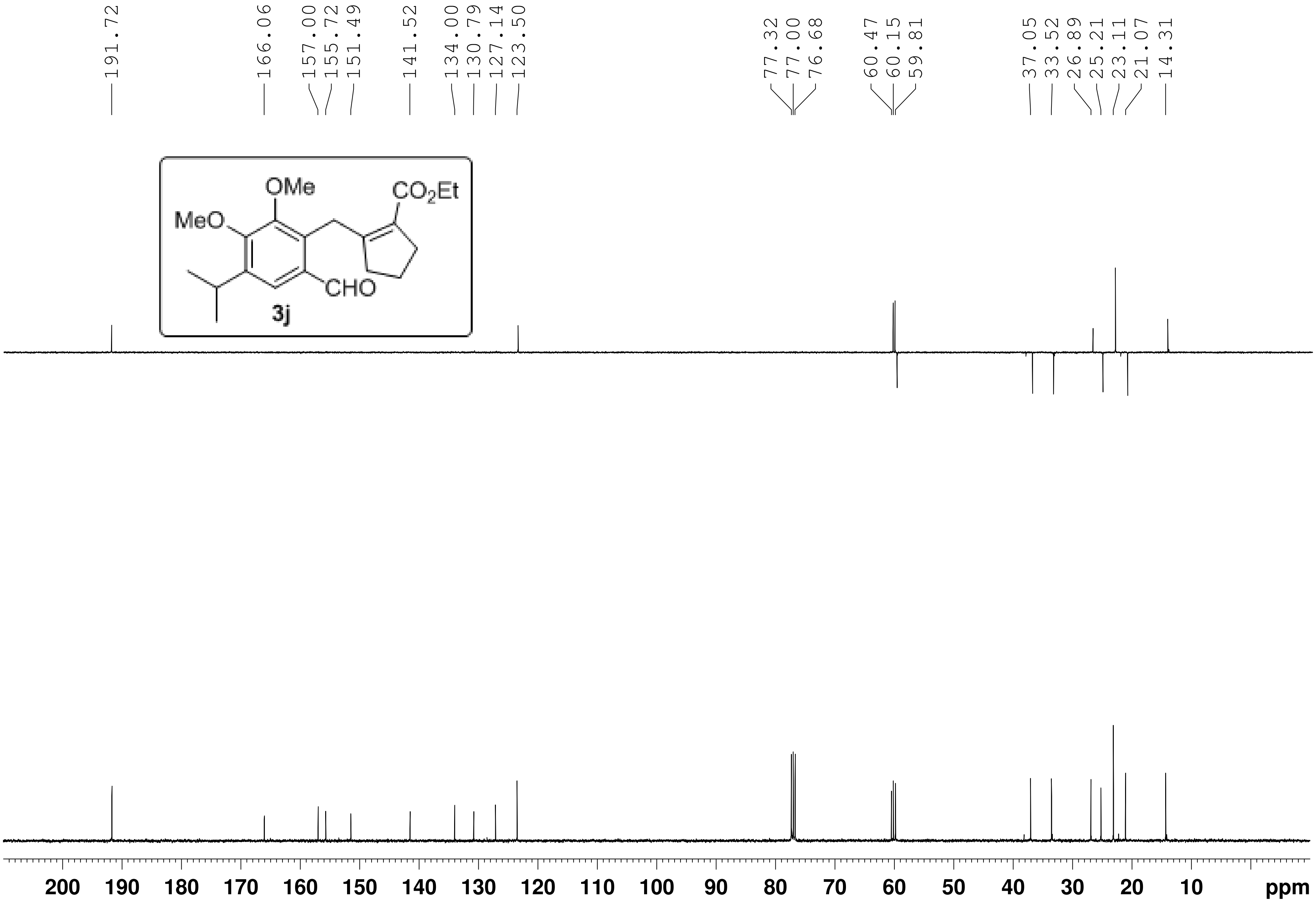

Supplementary Figure 133. ^{1}H NMR Spectrum of 3e (400 MHz, $CDCl_3$)

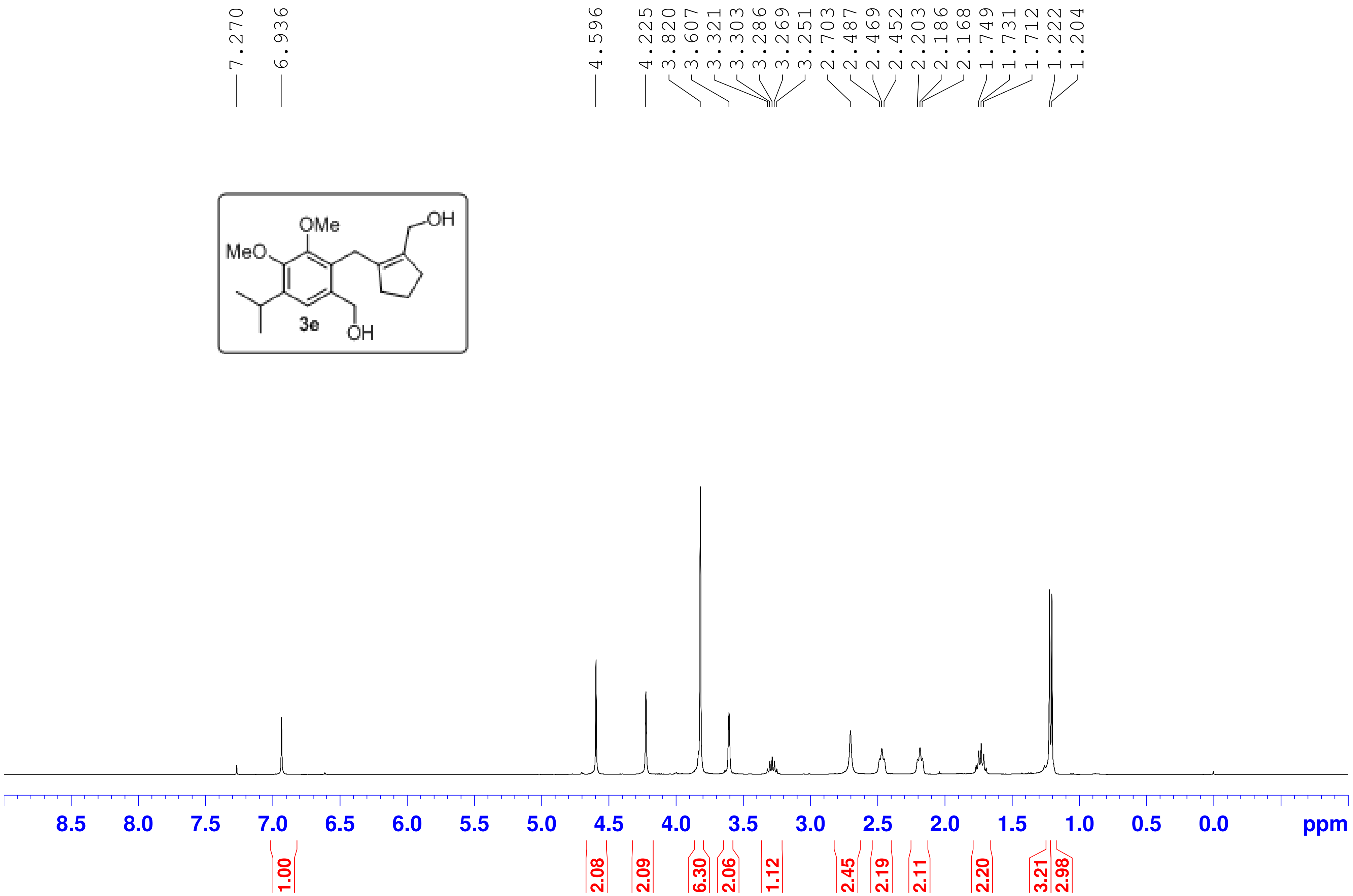

Supplementary Figure 134. ^{13}C NMR Spectrum of 3e (100 MHz, $CDCl_3$)

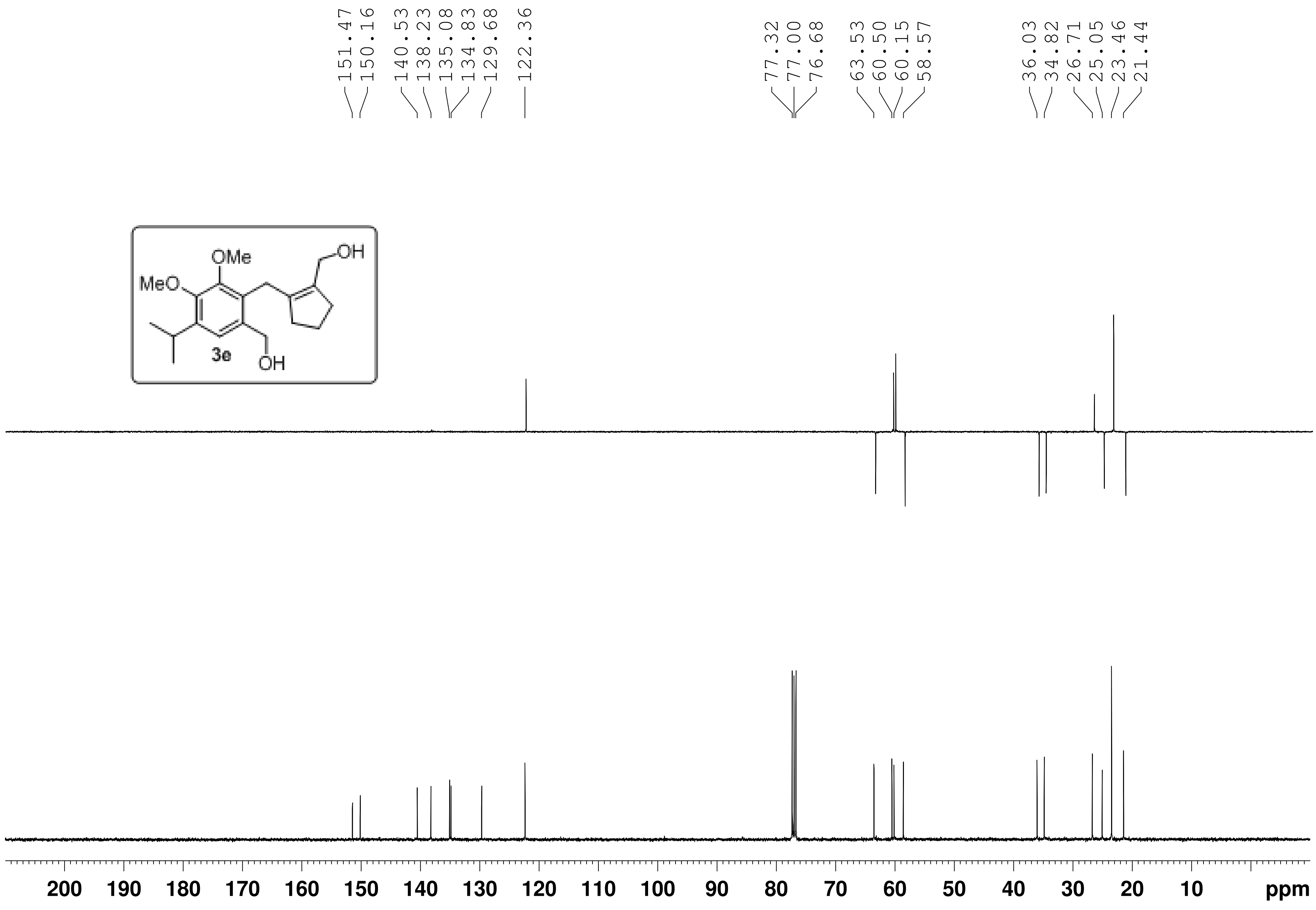

Supplementary Figure 135. ^{1}H NMR Spectrum of 3k (400 MHz, $CDCl_3$)

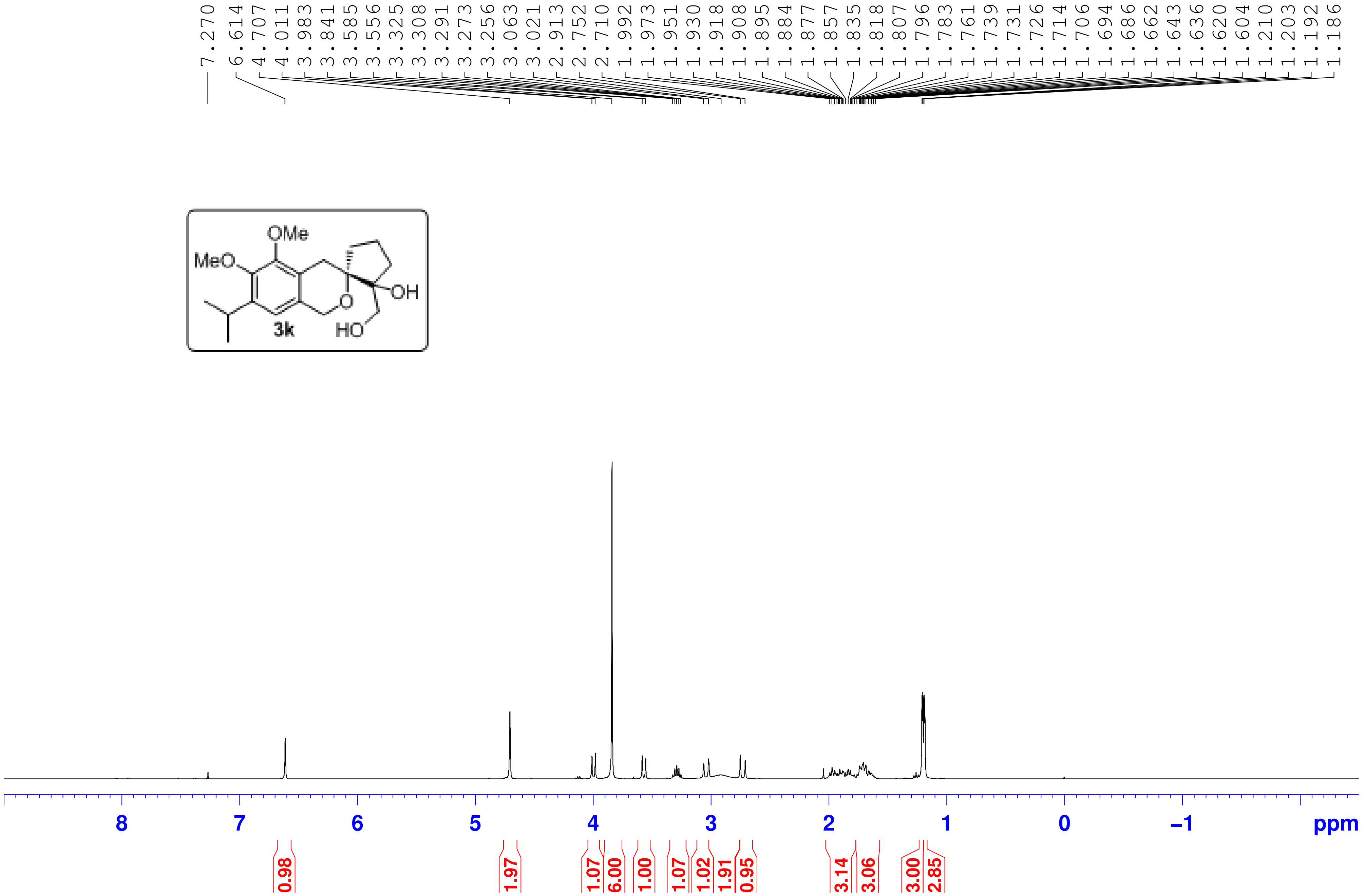

Supplementary Figure 136. ^{13}C NMR Spectrum of 3k (100 MHz, $CDCl_3$)

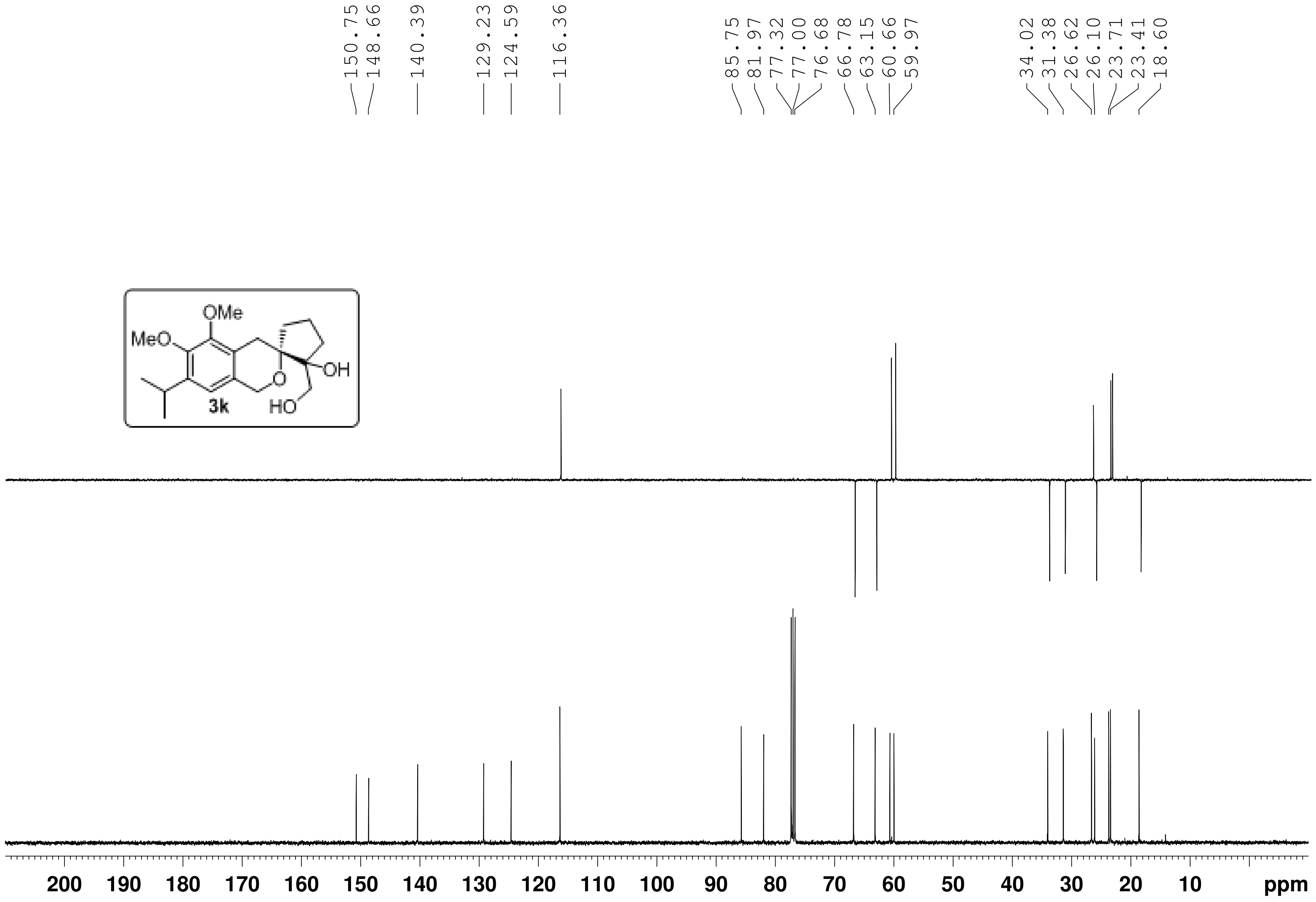

Supplementary Figure 137. HPLC Spectrum of racemic 3k

Sample Name: A570-Race

```
=====================================================================
Injection Date  : 11/5/2013 4:10:01 PM
Sample Name     : A570-Race                        Location : Vial 1
Acq. Operator   : jzw
Acq. Method     : C:\HPCHEM\1\METHODS\ZHANGQW.M
Last changed    : 11/5/2013 3:53:02 PM by jzw
                  (modified after loading)
Analysis Method : C:\HPCHEM\1\METHODS\ZHANGQW.M
Last changed    : 11/5/2013 4:35:21 PM by jzw
                  (modified after loading)
=====================================================================
```

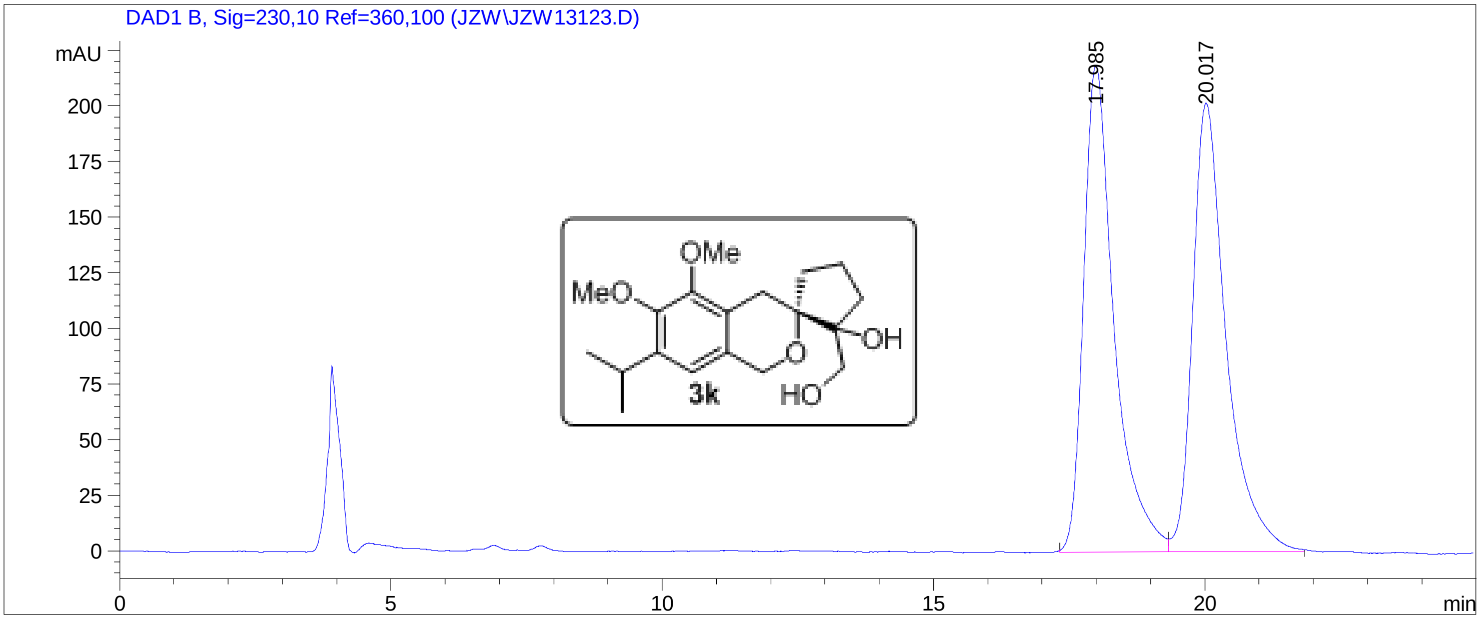


```
=====================================================================
                     Area Percent Report
=====================================================================

Sorted By             :      Signal
Multiplier            :      1.0000
Dilution              :      1.0000


Signal 1: DAD1 B, Sig=230,10 Ref=360,100

Peak RetTime Type  Width     Area      Height     Area
  #   [min]        [min]   [mAU*s]     [mAU]        %
----|-------|----|-------|----------|----------|--------|
   1  17.985 BV    0.5850 8570.99609  218.82797  49.6015
   2  20.017 VB    0.6396 8708.70703  201.76436  50.3985

Totals :                  1.72797e4   420.59233

 Results obtained with enhanced integrator!
=====================================================================
                     Summed Peaks Report
=====================================================================

Signal 1: DAD1 B, Sig=230,10 Ref=360,100
=====================================================================
                  Final Summed Peaks Report
=====================================================================

Signal 1: DAD1 B, Sig=230,10 Ref=360,100
```


PDF created with pdfFactory Pro trial version www.softwarelabs.com

Supplementary Figure 138. HPLC Spectrum of chiral 3k

Sample Name: A579-Asy

```
=====================================================================
Injection Date   : 11/5/2013 5:15:22 PM
Sample Name      : A579-Asy                       Location : Vial 1
Acq. Operator    : jzw
Method           : C:\HPCHEM\1\METHODS\ZHANGQW.M
Last changed     : 11/5/2013 4:35:21 PM by jzw
                   (modified after loading)
=====================================================================
```

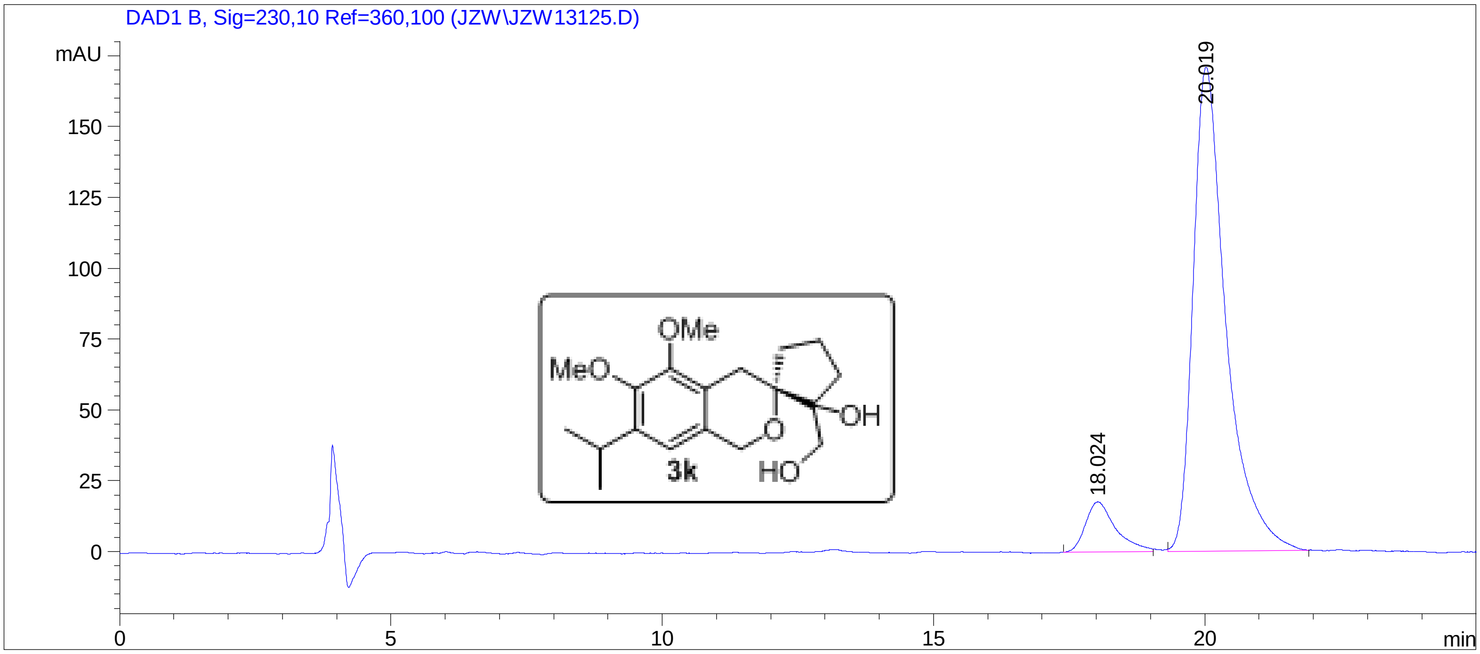


```
=====================================================================
                        Area Percent Report
=====================================================================

Sorted By            :      Signal
Multiplier           :      1.0000
Dilution             :      1.0000

Signal 1: DAD1 B, Sig=230,10 Ref=360,100
```

Peak #	RetTime [min]	Type	Width [min]	Area [mAU*s]	Height [mAU]	Area %
1	18.024	BB	0.5688	696.55188	17.79136	8.7803
2	20.019	BB	0.6381	7236.58789	170.86086	91.2197

Totals : 7933.13977 188.65221

Results obtained with enhanced integrator!

```
=====================================================================
                        Summed Peaks Report
=====================================================================

Signal 1: DAD1 B, Sig=230,10 Ref=360,100
=====================================================================
                     Final Summed Peaks Report
=====================================================================

Signal 1: DAD1 B, Sig=230,10 Ref=360,100
```


PDF created with pdfFactory Pro trial version www.softwarelabs.com

Supplementary Figure 139. ^1H NMR Spectrum of 3l (400 MHz, $CDCl_3$)

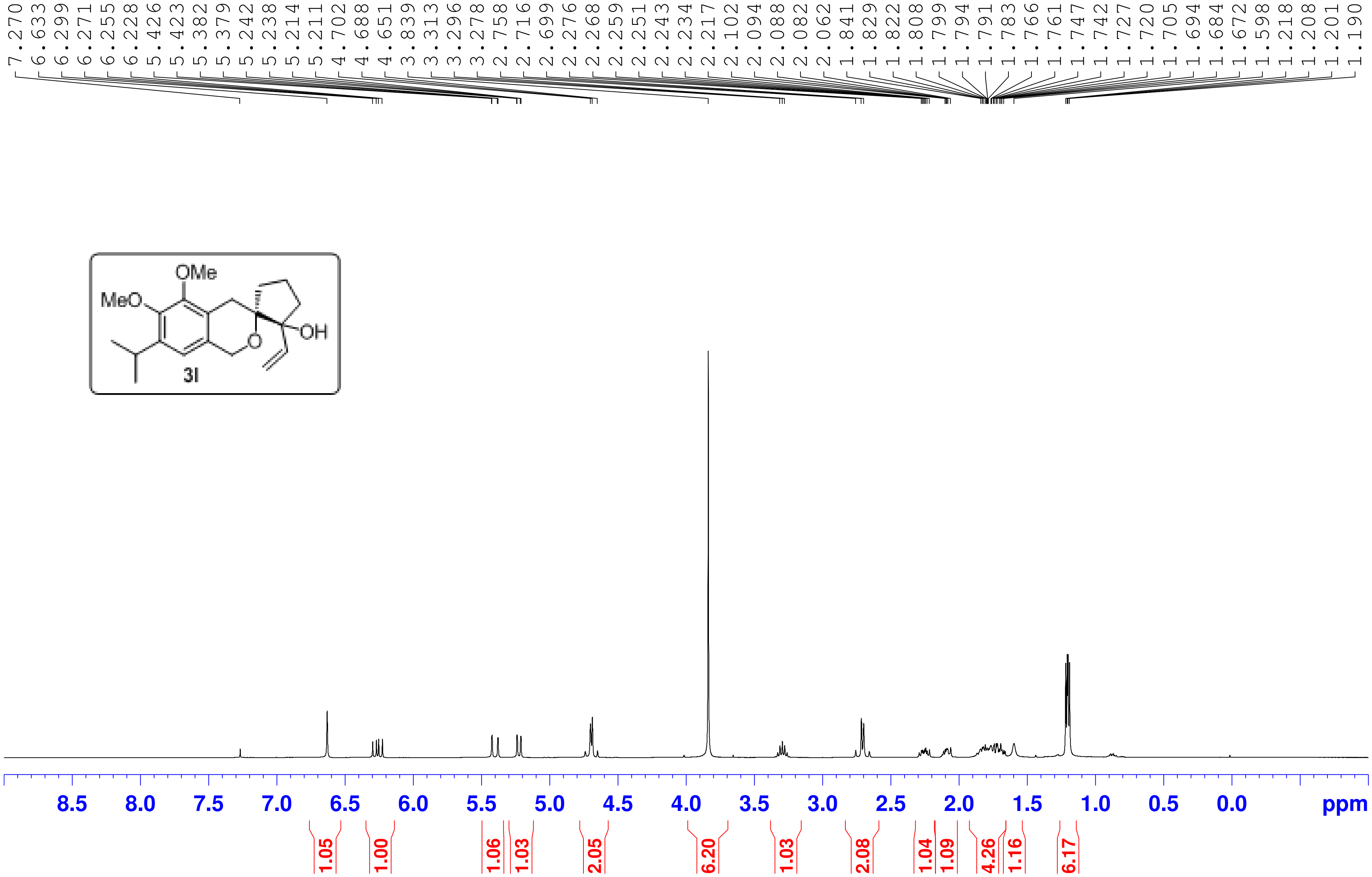

Supplementary Figure 140. ^{13}C NMR Spectrum of 3l (100 MHz, $CDCl_3$)

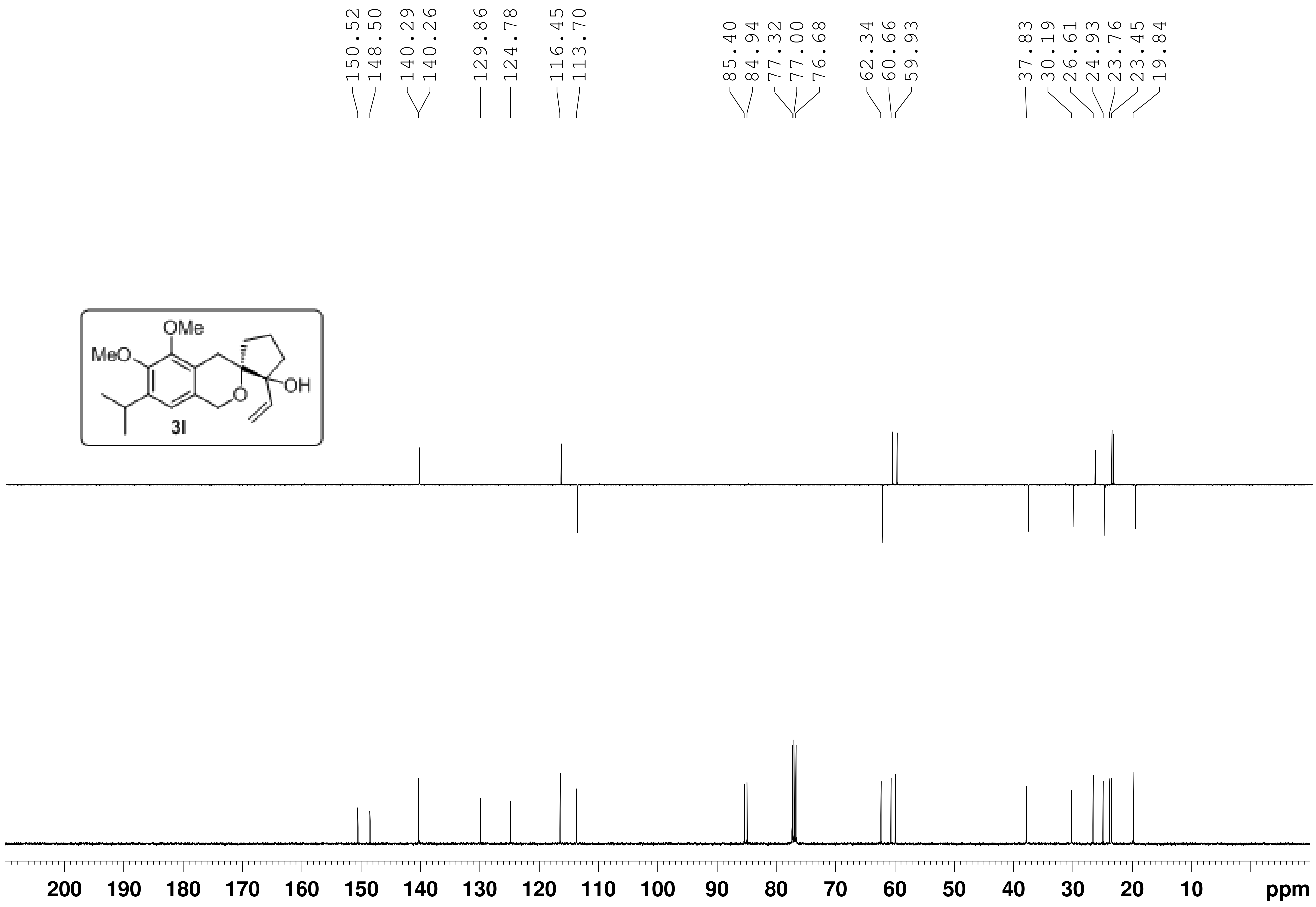

Supplementary Figure 141. ^{1}H NMR Spectrum of 3d (400 MHz, $CDCl_3$)

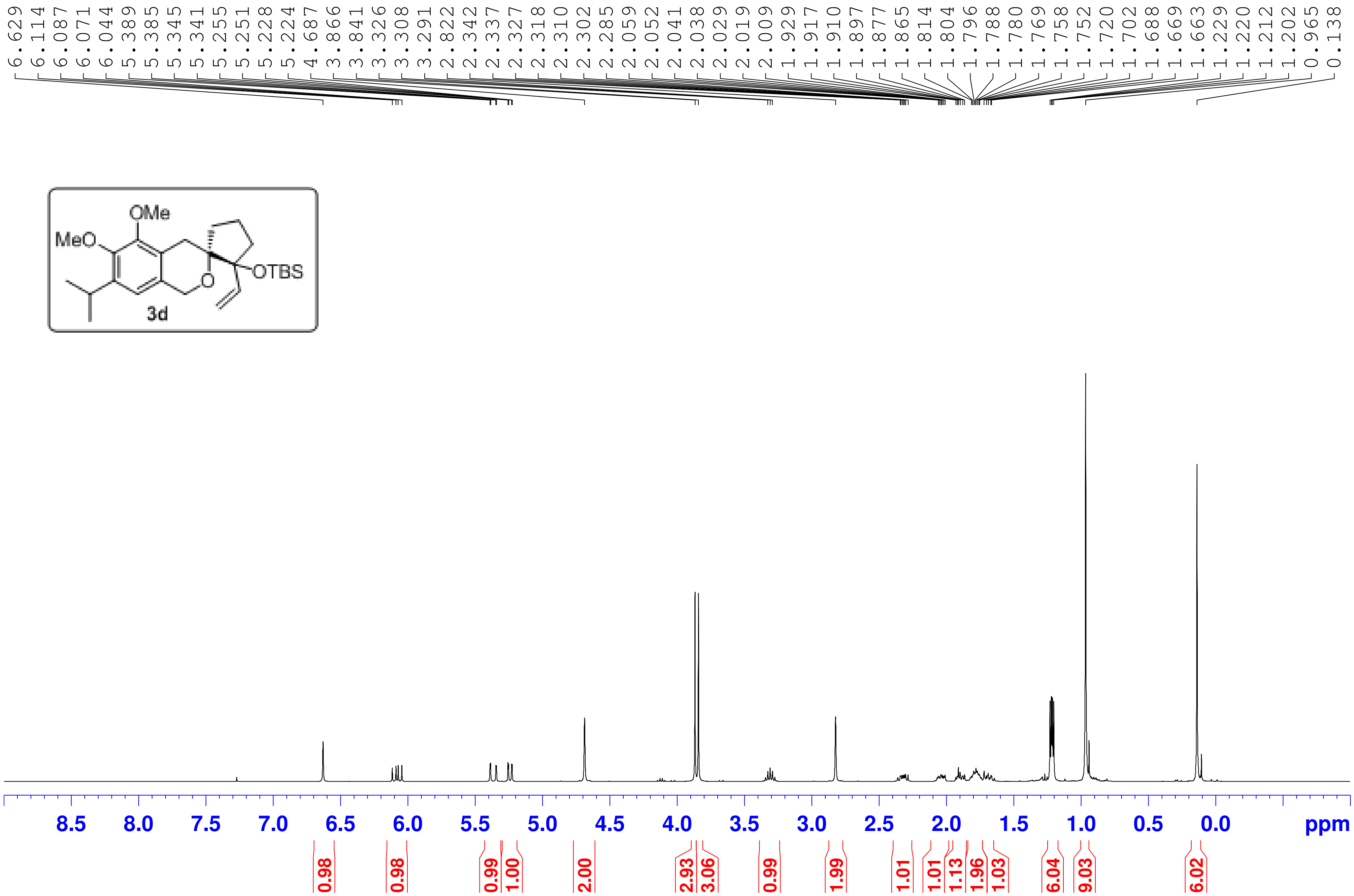

Supplementary Figure 142. ^{13}C NMR Spectrum of 3d (100 MHz, $CDCl_3$)

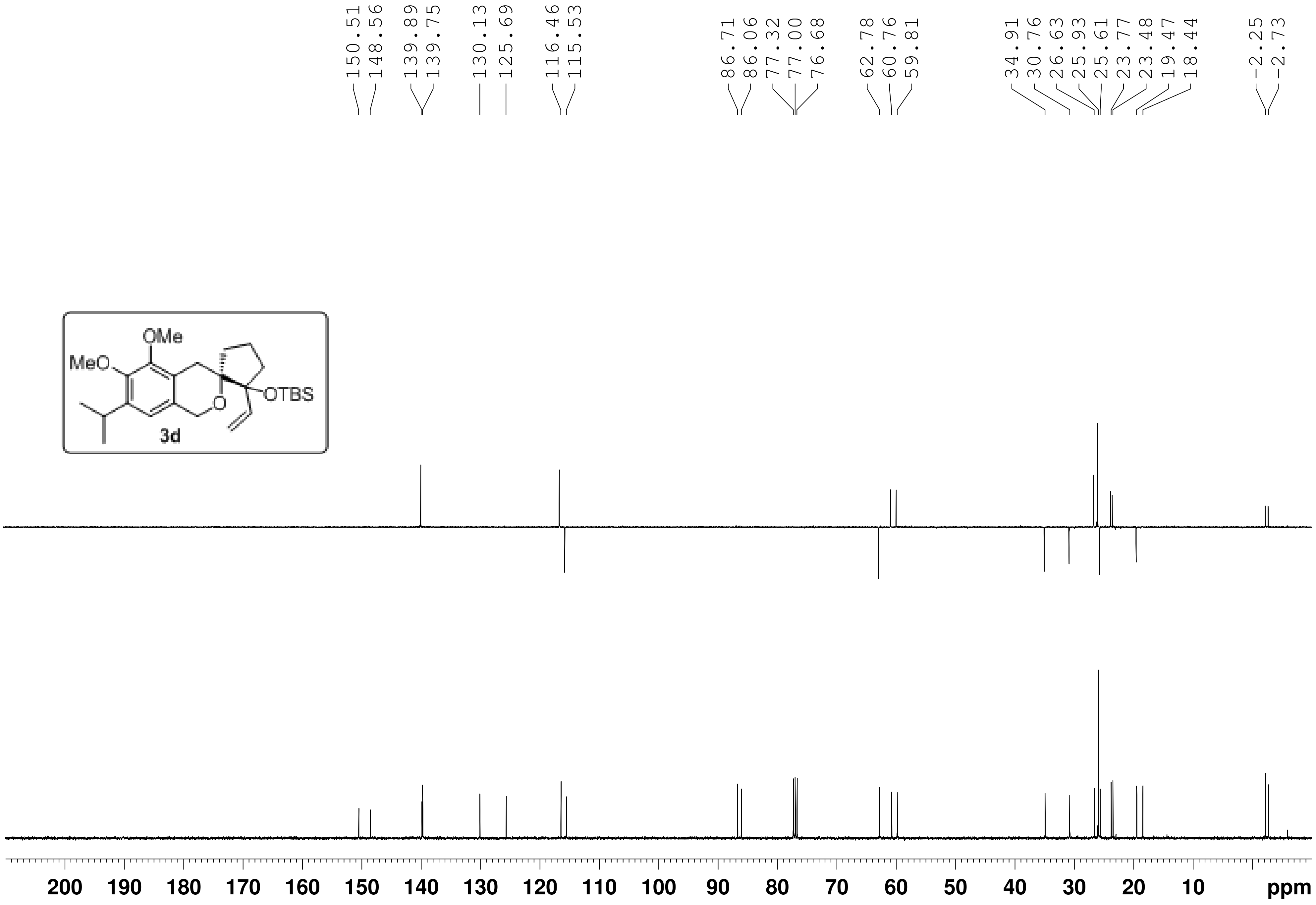

Supplementary Figure 143. ^{1}H NMR Spectrum of 3c (400 MHz, $CDCl_3$)

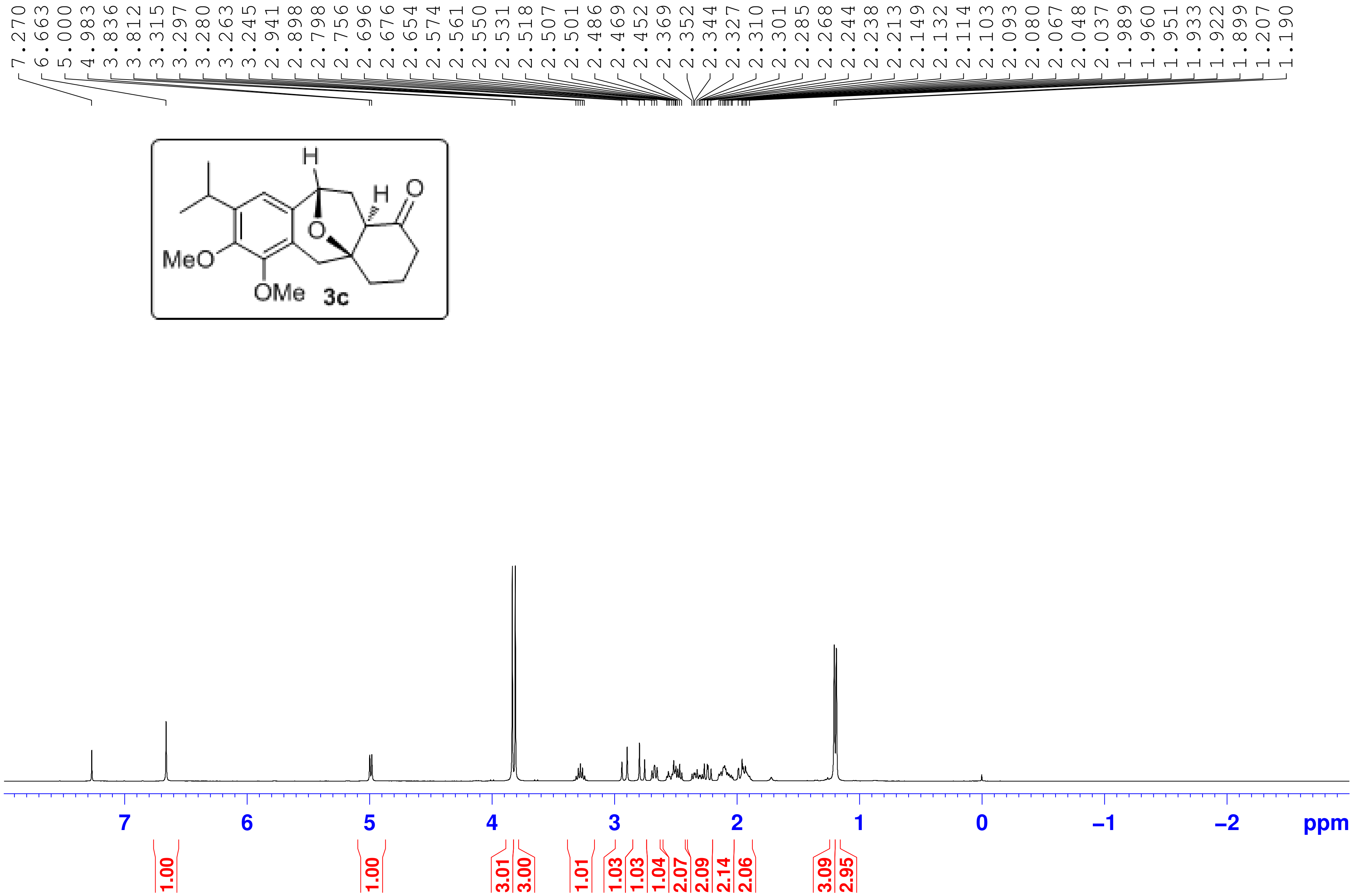

Supplementary Figure 144. ^{13}C NMR Spectrum of 3c (100 MHz, $CDCl_3$)

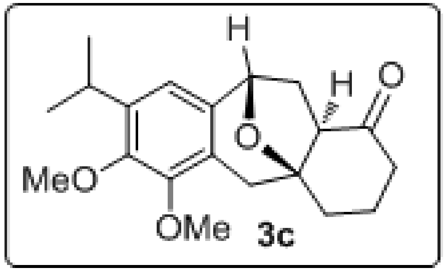

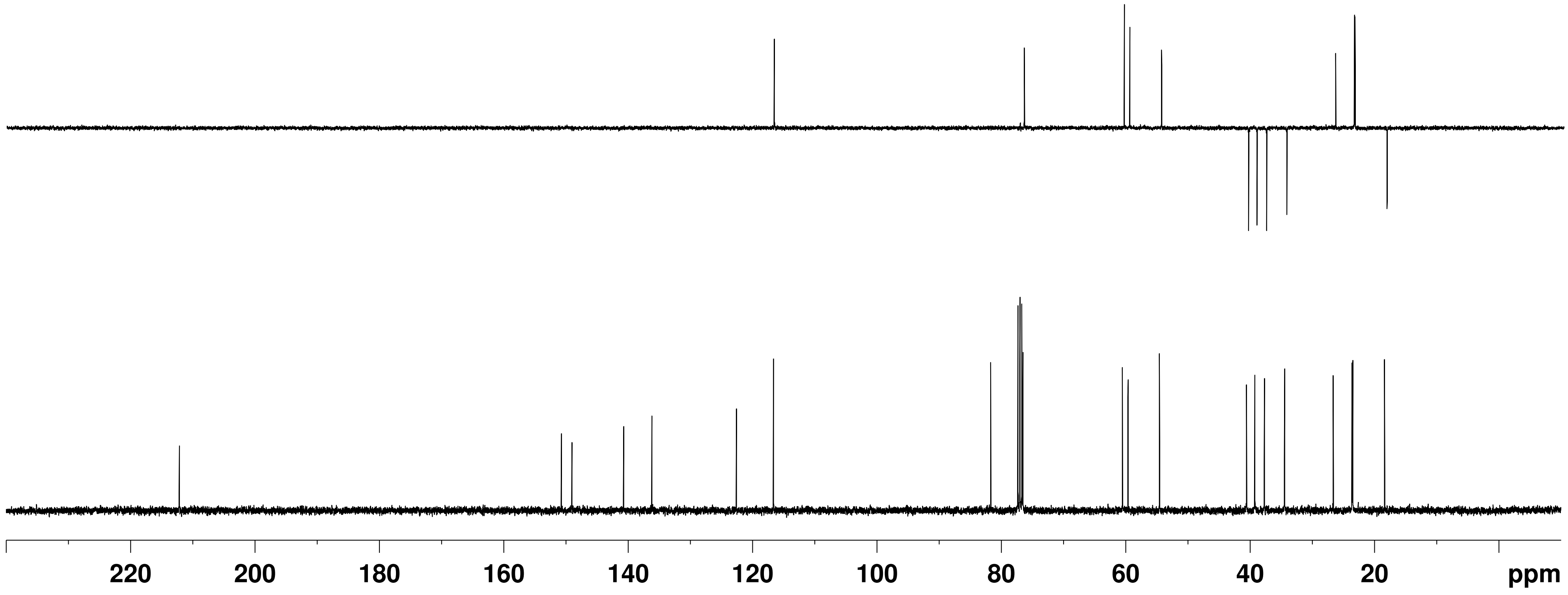

Supplementary Figure 145. ^{1}H NMR Spectrum of 3m (400 MHz, $CDCl_3$)

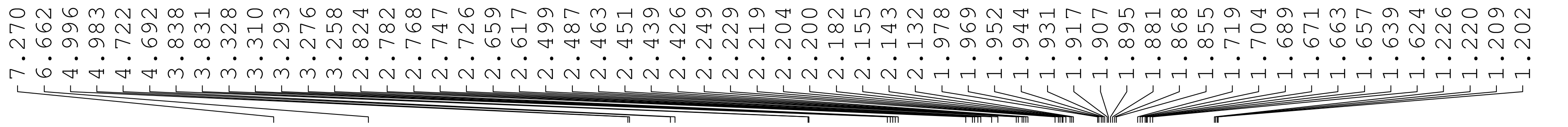

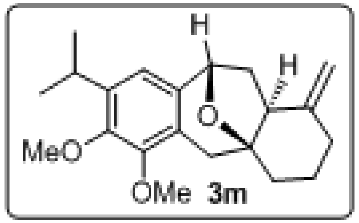

3m

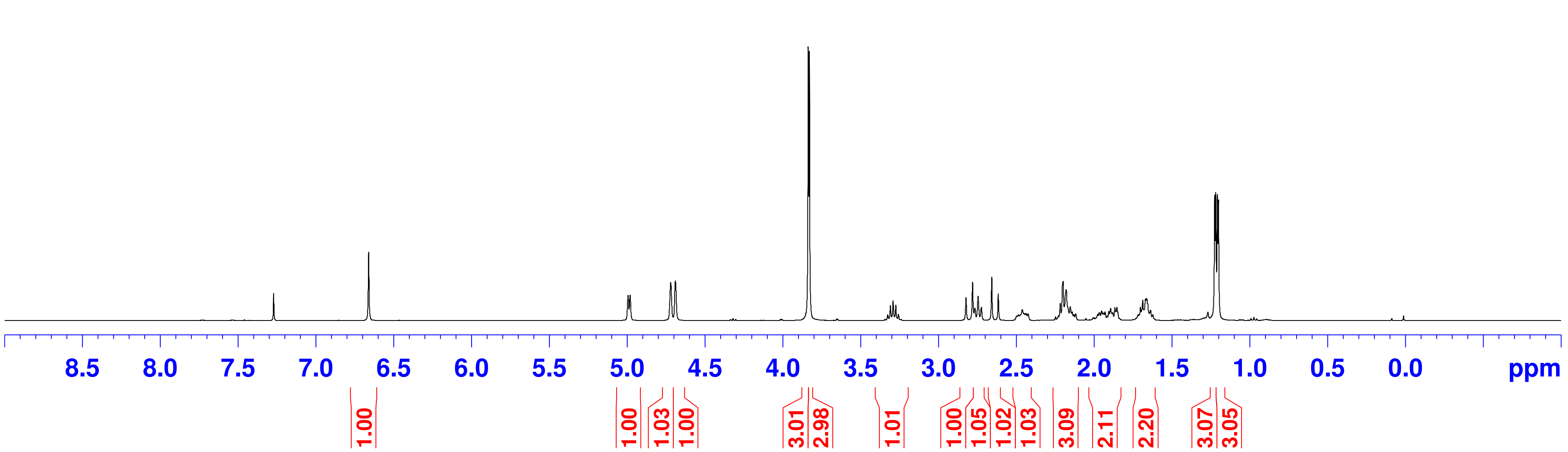

Supplementary Figure 146. ^{13}C NMR Spectrum of 3m (100 MHz, $CDCl_3$)

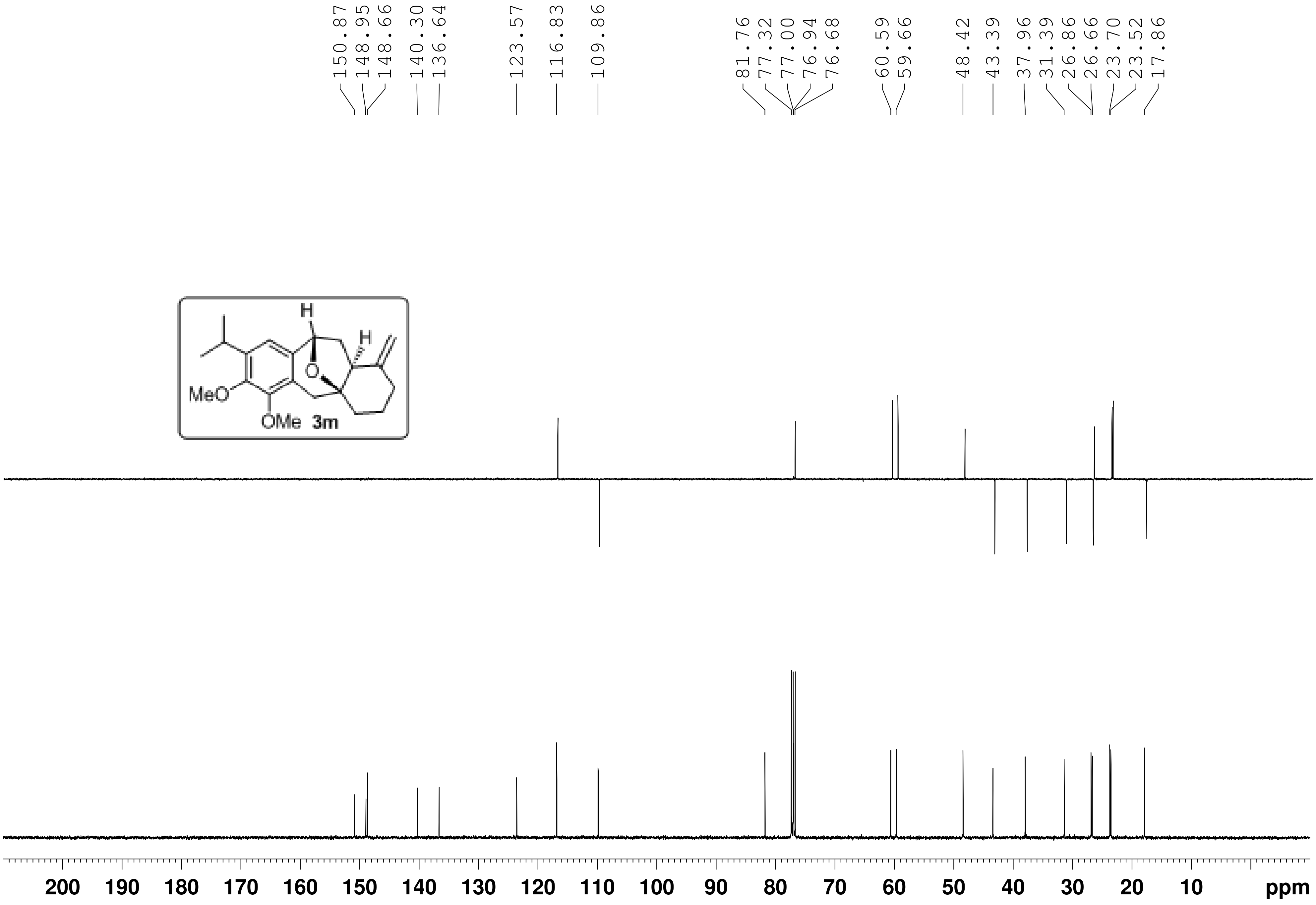

Supplementary Figure 147. ^{1}H NMR Spectrum of 3n (400 MHz, $CDCl_3$)

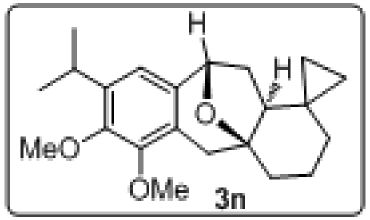

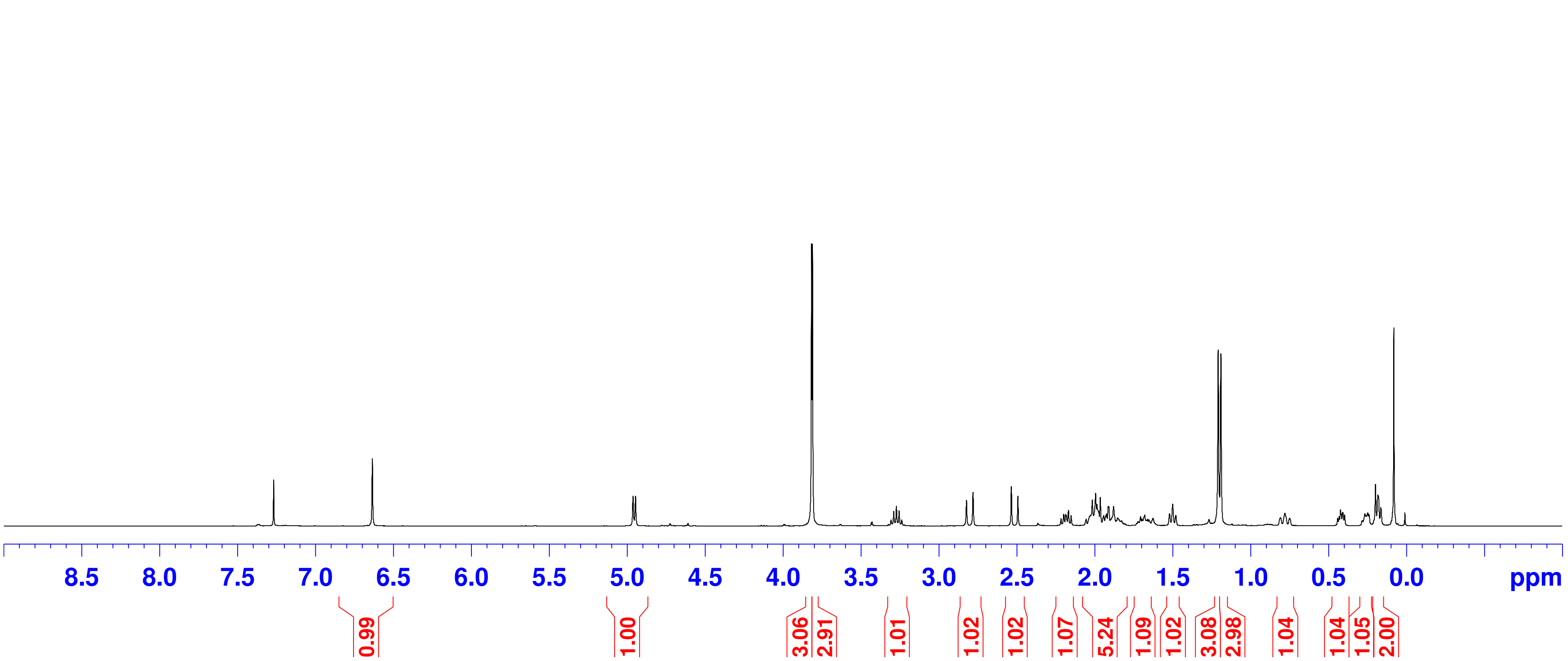

Supplementary Figure 148. ^{13}C NMR Spectrum of 3n (100 MHz, $CDCl_3$)

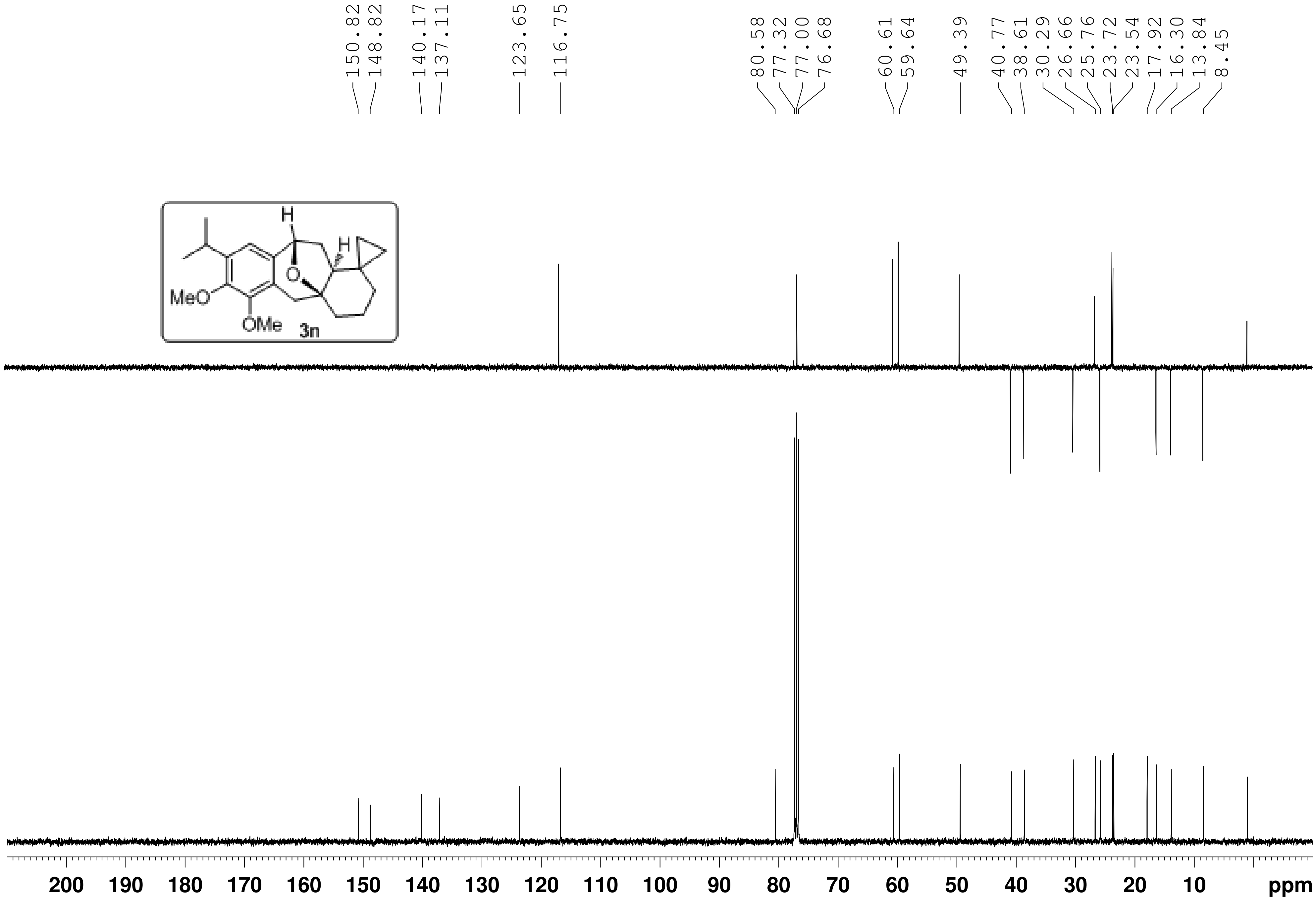

Supplementary Figure 149. ^{1}H NMR Spectrum of 3o (400 MHz, $CDCl_3$)

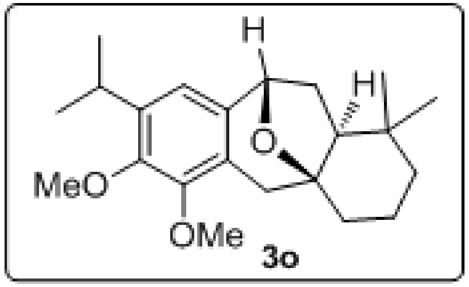

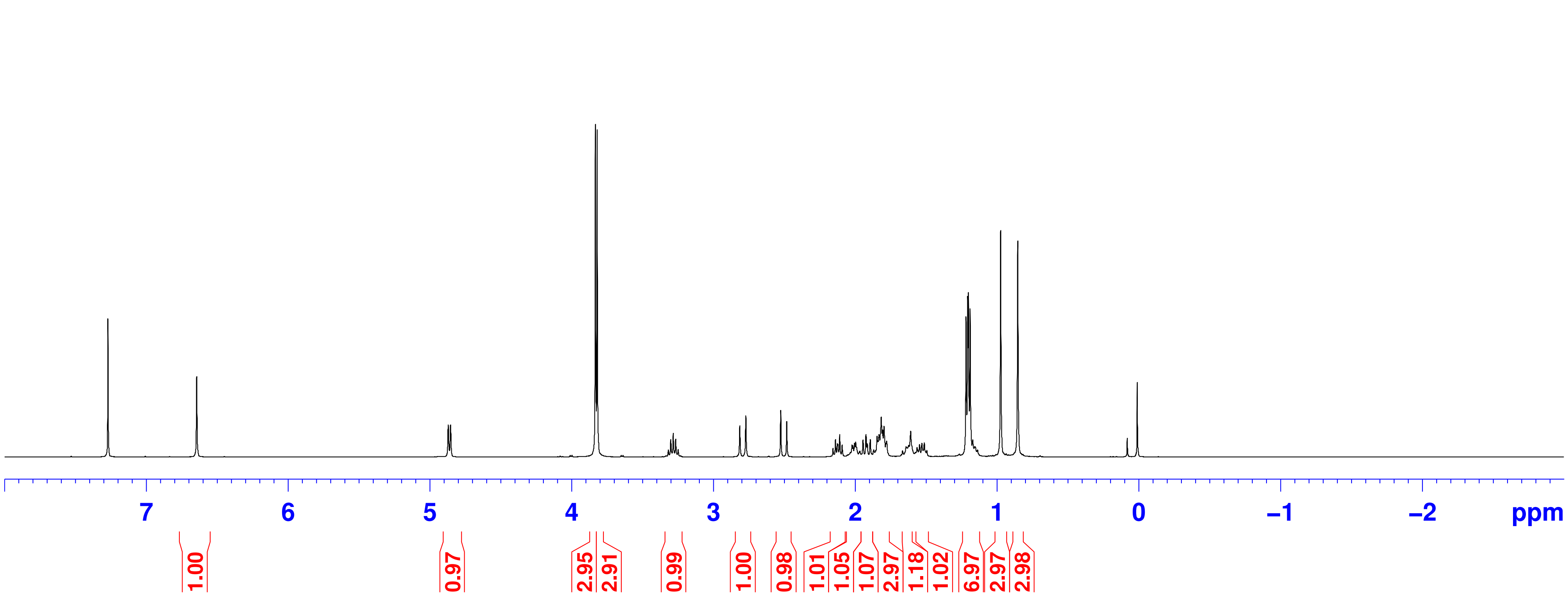

Supplementary Figure 150. ^{13}C NMR Spectrum of 3o (100 MHz, $CDCl_3$)

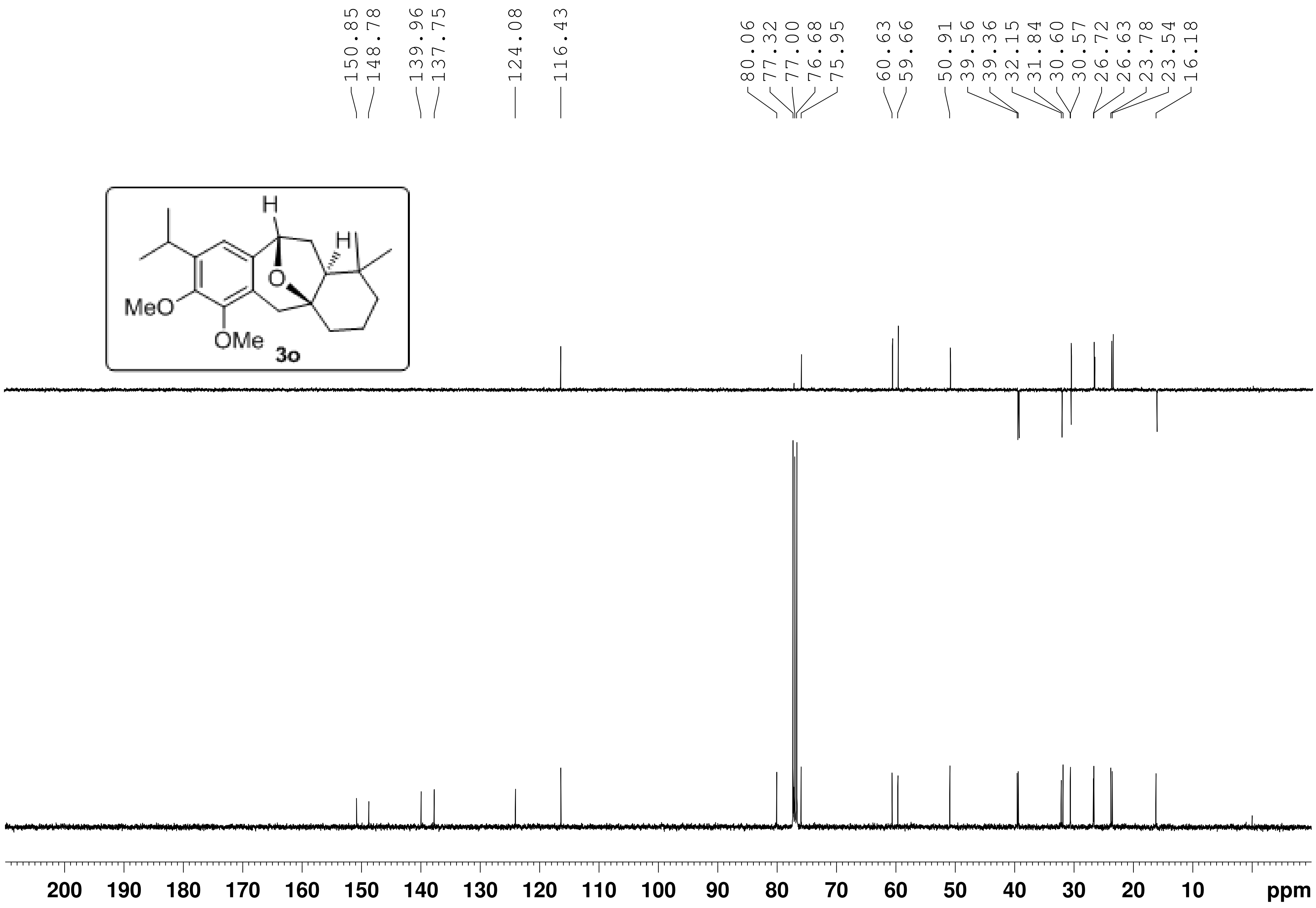

Supplementary Figure 151. ^{1}H NMR Spectrum of 3b (400 MHz, $CDCl_3$)

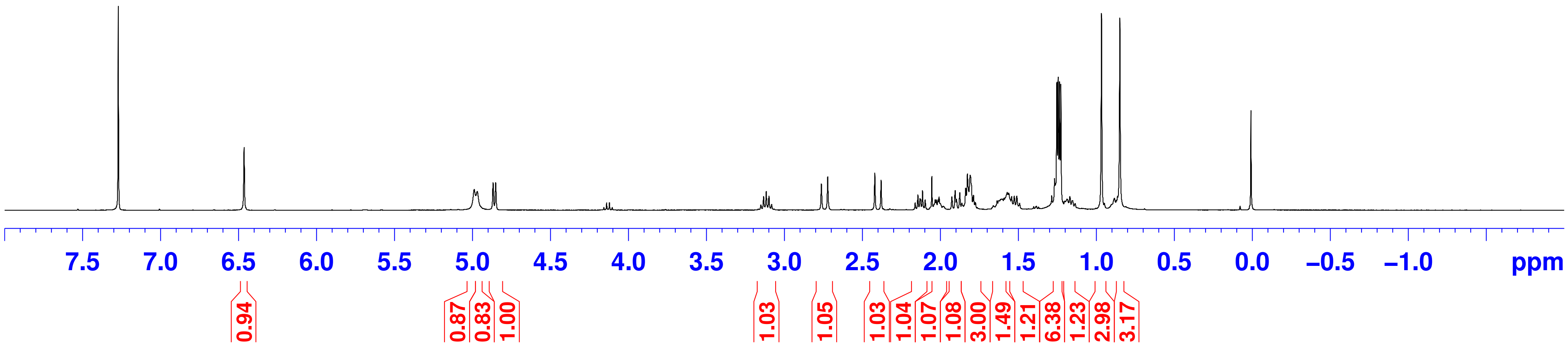

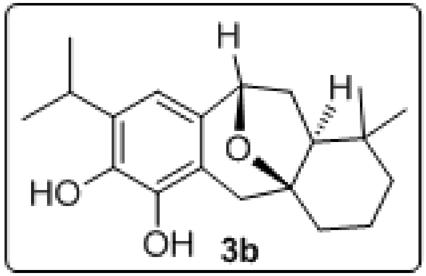

3b

Supplementary Figure 152. ^{13}C NMR Spectrum of 3b (100 MHz, $CDCl_3$)

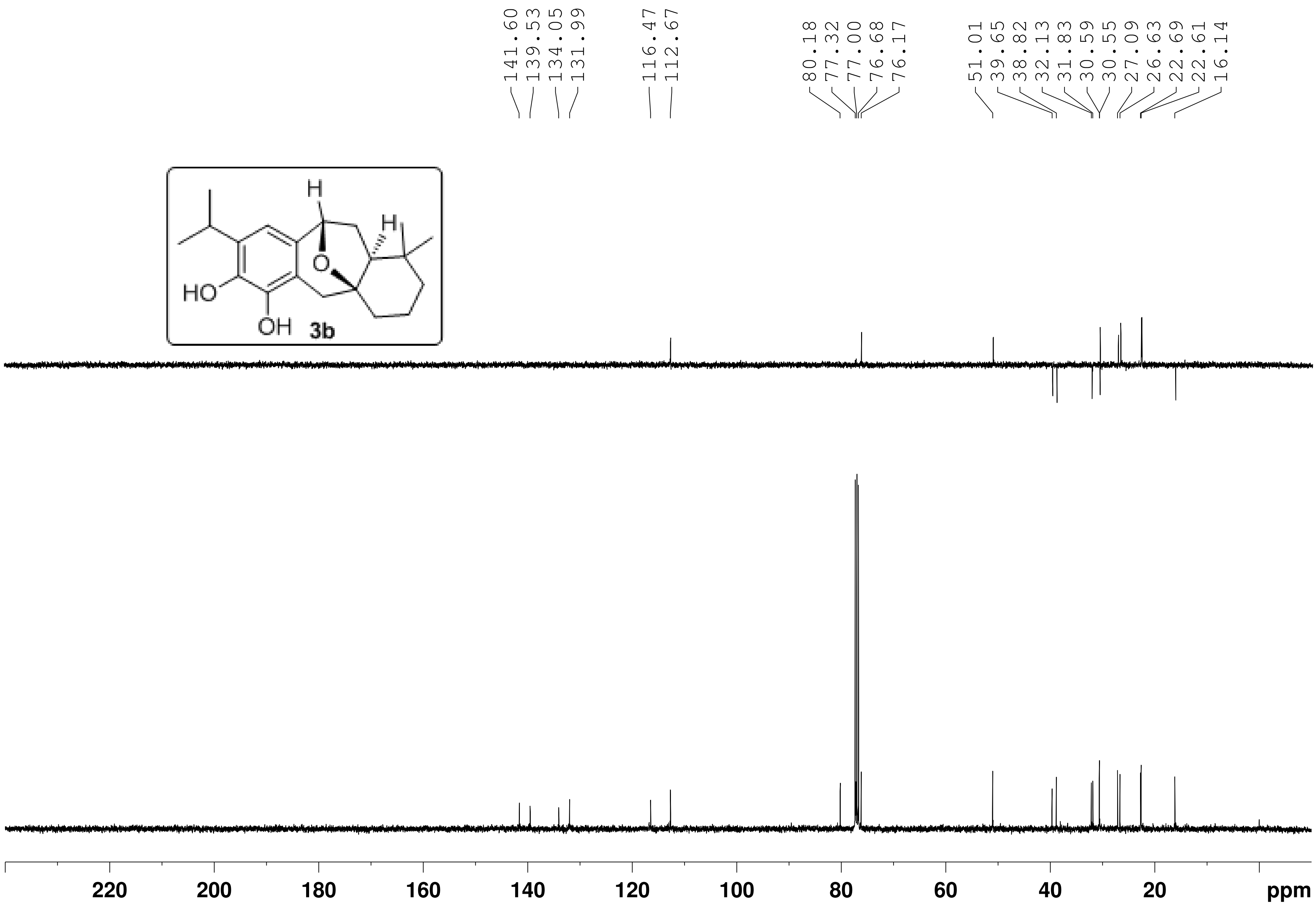

Supplementary Figure 153. ^{1}H NMR Spectrum of 3a (400 MHz, $CDCl_3$)

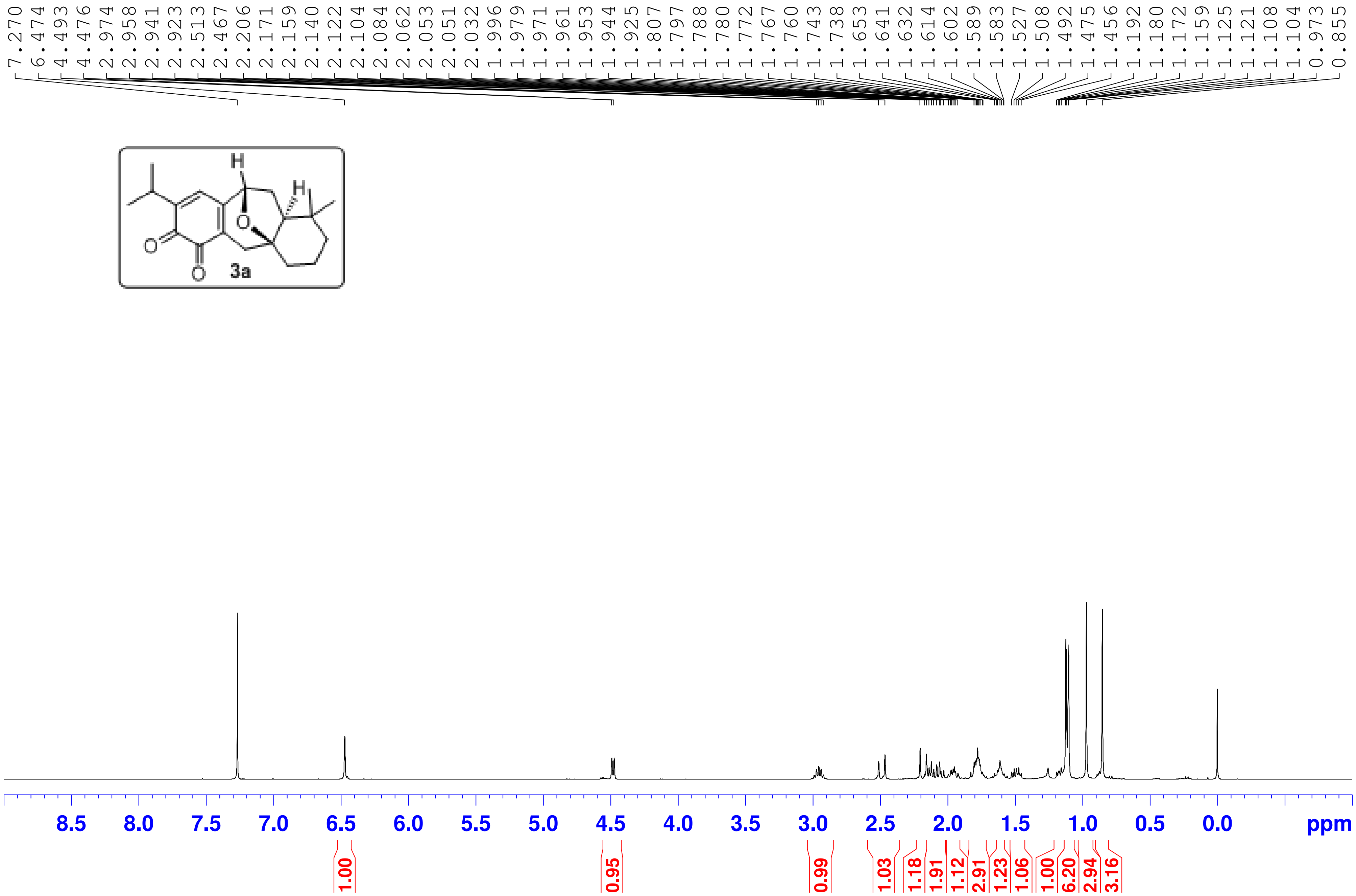

Supplementary Figure 154. ^{13}C NMR Spectrum of 3a (100 MHz, $CDCl_3$)

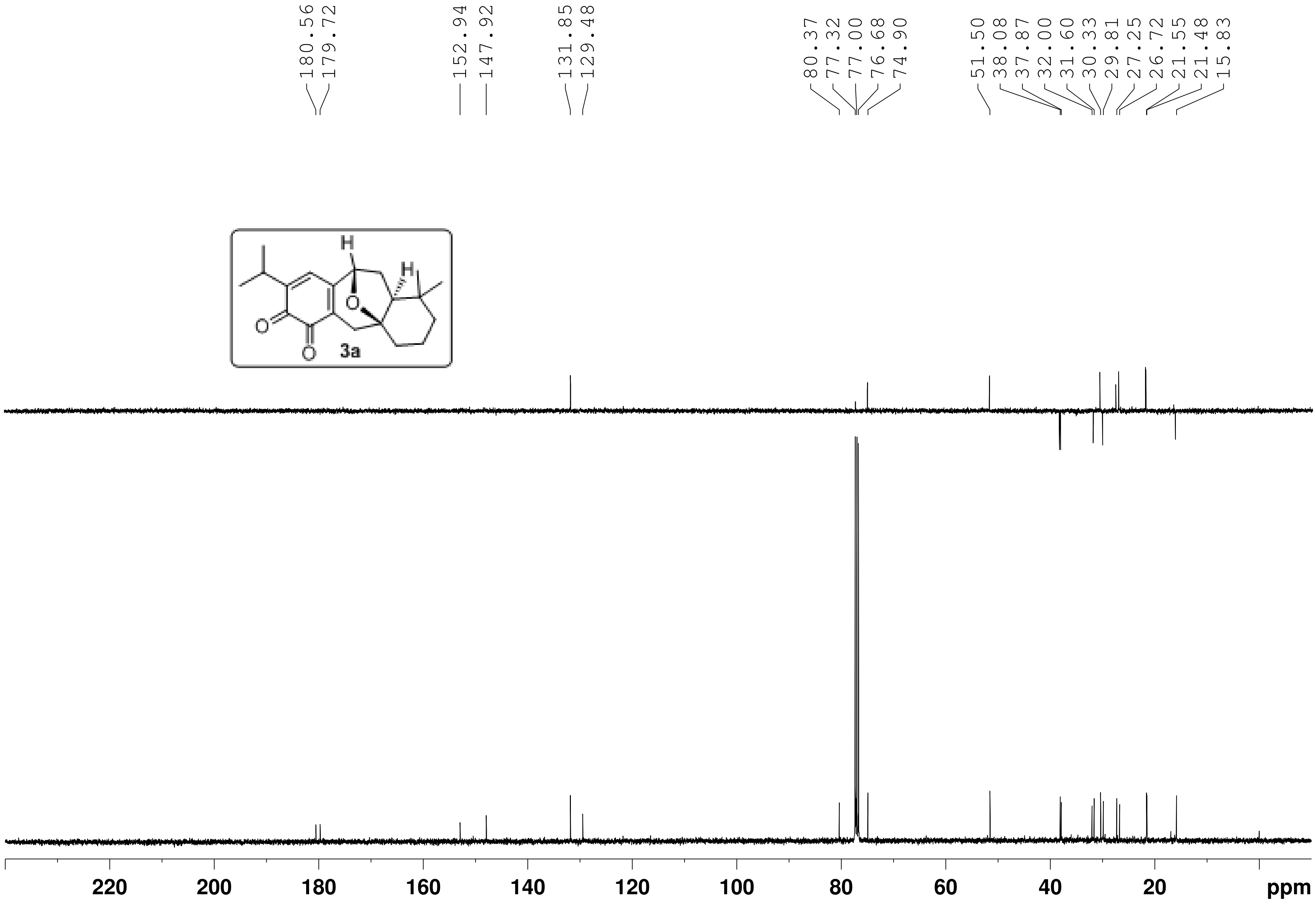

Supplementary Figure 155. ORTEP Diagram of 1e

Datablock jiaozhw0604 - ellipsoid plot

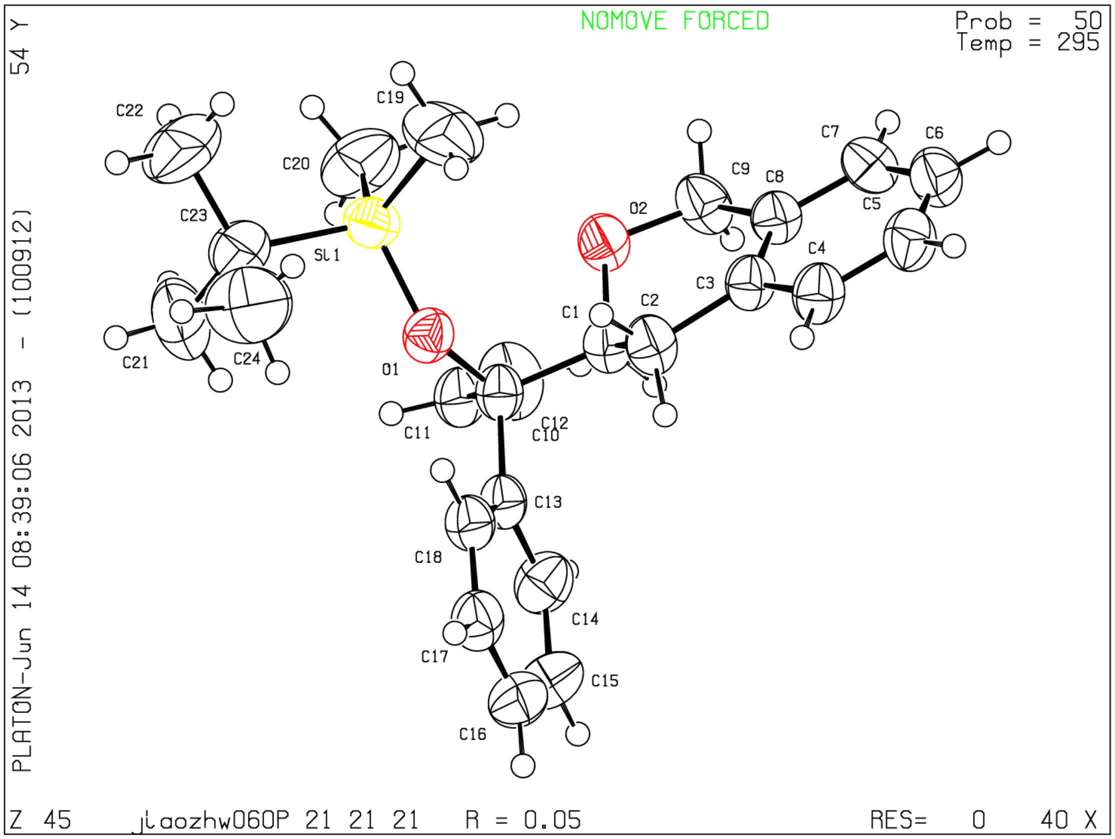

Supplementary Figure 156. ORTEP Diagram of S16'

Datablock jiaozhw0504 - ellipsoid plot

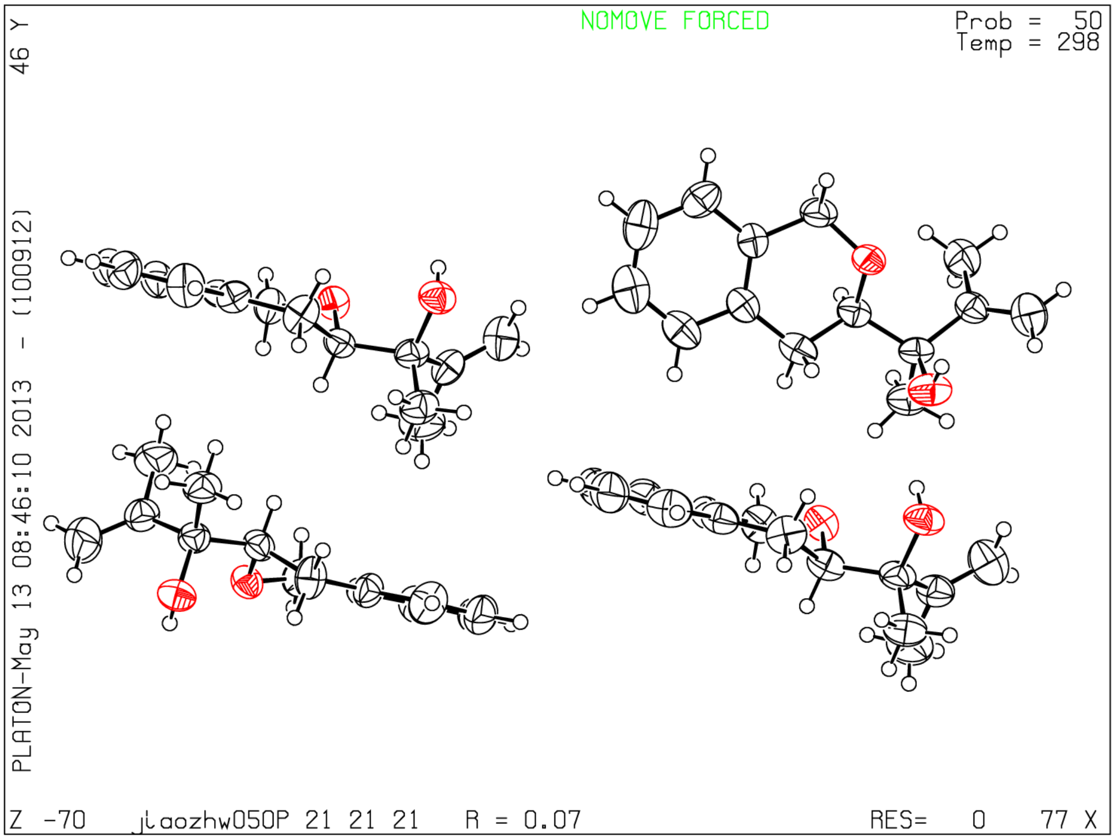

Supplementary Figure 157. ORTEP Diagram of 2h

PLATON version of 24/04/2013; check.def file version of 23/04/2013

Datablock jiaozhw0522 - ellipsoid plot

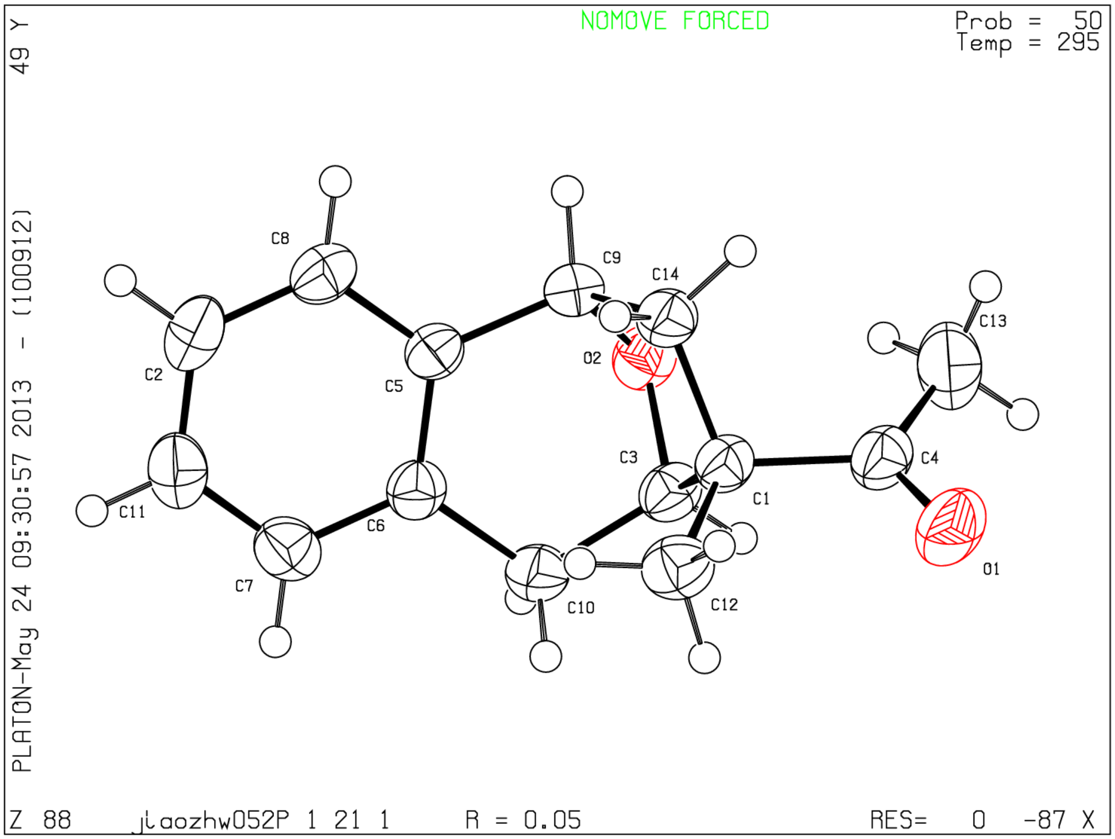

Supplementary Figure 158. ORTEP Diagram of 2j

Datablock jiaozhw1223 - ellipsoid plot

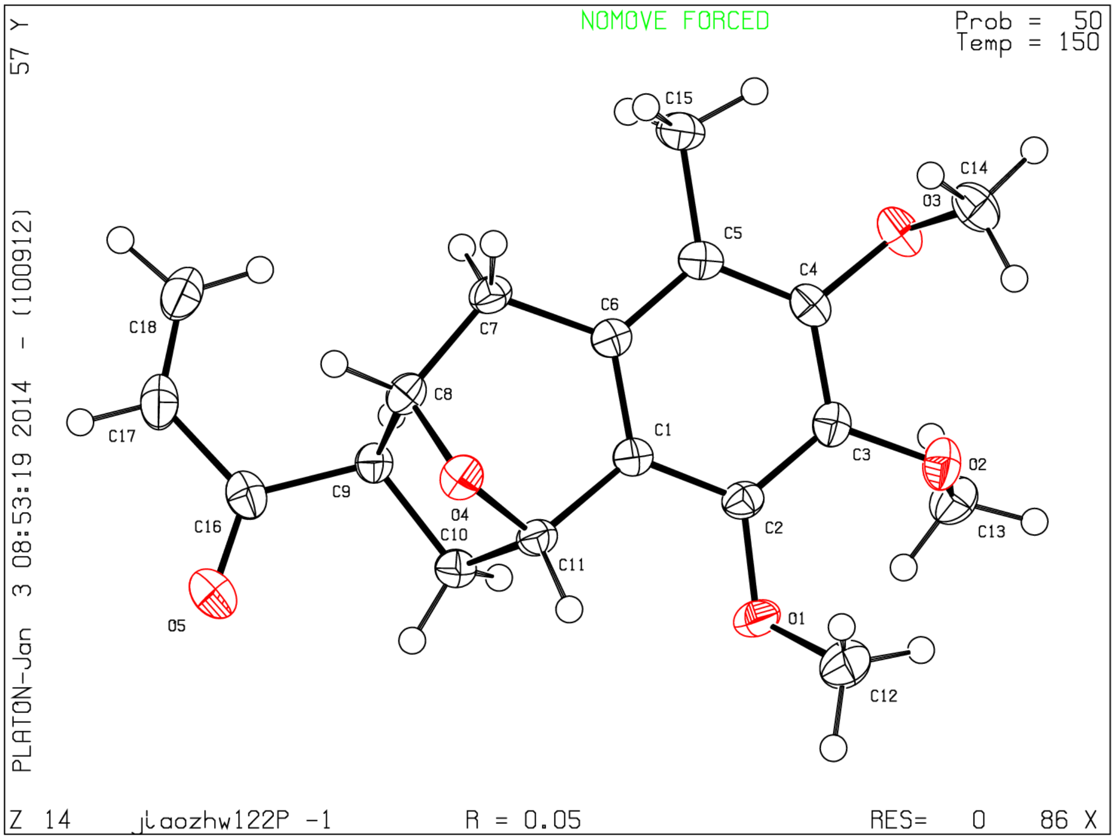

Supplementary Table 1. Comparison of the ^{1}H NMR ($CDCl_3$) data of reported (see Supplementary Reference 2) and our synthetic 3b

Reported δH [ppm, mult, *J* (Hz)] 500 MHz		Synthetic δH [ppm, mult, *J* (Hz)] 400 MHz		Err (Reported−Synthetic) ΔδH (ppm)
6.46	1 H, s	6.46	1H, s	0
4.99	1 H, s	4.99	1H, s	0
4.96	1 H, s	4.97	1H, s	−0.01
4.85	1 H, d, 6.7	4.86	1H, d, 6.4	−0.01
3.11	1 H, septet	3.15-3.08	1 H, m	−
2.73	1 H, d, 16.2	2.74	1 H, d, 16.4	−0.01
2.39	1 H, d, 16.2	2.40	1 H, d, 16.4	−0.01
2.12	1 H, dt, 12.1, 6.8	2.16-2.10	1 H, m	−
2.04-1.96	1 H, m	2.04-1.98	1 H, m	−
1.89	1 H, dd, 11.9, 8.4	1.92-1.88	1 H, m	−
1.85-1.75	3 H, m	1.87-1.77	3 H, m	−
1.65-1.58	1 H, m	1.66-1.59	1 H, m	−
1.55-1.48	1 H, m	1.57-1.49	1 H, m	−
1.24	3 H, d, 6.9	1.26	3 H, d, 6.8	−0.02
1.23	3 H, d, 6.8	1.25	3 H, d, 6.8	−0.02
1.20-1.12	1 H, m	1.24-1.23	1 H, m	−
0.96	3 H, s	0.97	3 H, s	−0.01
0.84	3 H, s	0.85	3 H, s	−0.01

Supplementary Table 2. Comparison of the ^{13}C NMR ($CDCl_3$) data of reported and our synthetic 3b

Reported δC (ppm) 125 MHz	Synthetic δC (ppm) 100 MHz	Err (Reported−Synthetic) ΔδC (ppm)
141.4	141.6	-0.2
139.3	139.5	-0.2
134.3	134.1	0.2
131.8	132.0	-0.2
116.5	116.5	0
112.7	112.7	0
80.0	80.2	-0.2
76.1	76.2	-0.1
51.1	51.0	0.1
39.7	39.7	0
38.7	38.8	-0.1
32.2	32.1	0.2
31.9	31.8	0.1
30.7	30.6	0.1

30.6	30.6	0
27.2	27.1	0.1
26.7	26.6	0.1
22.8	22.7	0.1
22.6	22.6	0
16.2	16.1	0.1

Supplementary Table 3. Comparison of the ^{1}H NMR ($CDCl_3$) data of natural (see Supplementary Reference 3) and our synthetic 3a

**Natural**<br>δH [ppm, mult, *J* (Hz)]<br>400 MHz		**Synthetic**<br>δH [ppm, mult, *J* (Hz)]<br>400 MHz		**Err**<br>(Natural−Synthetic)<br>ΔδH (ppm)
6.45	1 H, s	6.47	1H, s	−0.02
4.40	1 H, d, 6.8	4.48	1H, d, 6.4	−0.08
2.92	1 H, septet, 6.8	2.99-2.92	1H, m	−
2.46	1 H, d, 18.3	2.49	1H, d, 18.4	−0.03
2.15	1 H, d, 18.3	2.18	1 H, d, 18.4	−0.03
2.15-1.15	9 H, m	2.14-1.16	9 H, m	−
1.09	1 H, m	1.12	3 H, d, 6.8	−0.03
1.09	1 H, m	1.11	3 H, d, 6.8	−0.02
0.95	3 H, s	0.97	3 H, s	−0.02
0.83	3 H, s	0.86	3 H, s	−0.03

Supplementary Table 4. Comparison of the ^{13}CNMR ($CDCl_3$) data of reported and our synthetic 3a

**Natural**<br>δC (ppm)<br>100 MHz	**Synthetic**<br>δC (ppm)<br>100 MHz	**Err**<br>(Natural−Synthetic)<br>ΔδC (ppm)
180.3	180.6	-0.3
179.7	179.7	0
153.0	152.9	0.1
147.9	147.9	0
131.9	131.9	0
129.5	129.5	0
80.4	80.4	0
74.9	74.9	0
51.5	51.5	0
38.0	38.1	-0.1
37.9	37.9	0
32.0	32.0	0
31.6	31.6	0
30.3	30.3	0
29.8	29.8	0

27.2	27.3	-0.1
26.7	26.7	0
21.6	21.6	0
21.5	21.5	0
15.8	15.8	0

Supplementary Methods

1. **Syntheses of the substrates and Spectroscopic Data of Compounds**

2. **Experimental Procedures of the tandem reaction and Spectroscopic Data of Compounds**

3. **Total syntheses of (-)-brussonol and (-)-przewalskine *E* and Spectroscopic Data of Compounds**

General Information:

All reactions under standard conditions were monitored by thin-layer chromatography (TLC) on silica gel F254 plates. Column chromatography was performed on silica gel (200-300 meshes). Solvents for reaction were distilled prior to use, and all air- or moisture-sensitive reactions were conducted under an argon atmosphere. The melting points were measured using micro melting point apparatus. The optical rotations were measured using a 0.1-mL cell with a 1-cm path length. ^{1}H NMR and ^{13}C NMR spectra were recorded in $CDCl_3$ solution on instruments (400 MHz for ^{1}H NMR and 100 MHz for ^{13}C NMR) and spectral data were reported in ppm relative to tetramethylsilane (TMS) as internal standard. IR spectra were recorded on a fourier transform infrared spectrometer. EI-MS spectra (MS) were measured on spectrometer by direct inlet at 70 eV and signals were given in *m/z* with relative intensity (%) in brackets. High-resolution mass spectral analysis (HRMS) data were measured by means of the ESI technique on Fourier transform ion cyclotron resonance mass analyzer.

1. Syntheses of the substrates

1.1 Synthesis of the substrate 1a

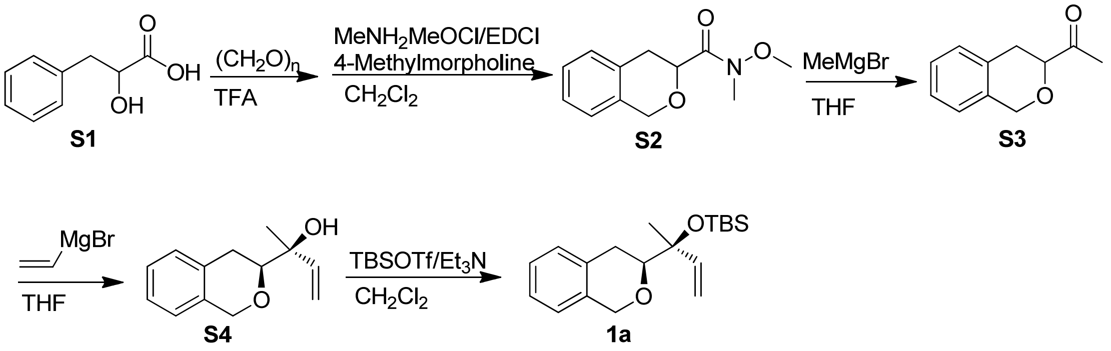

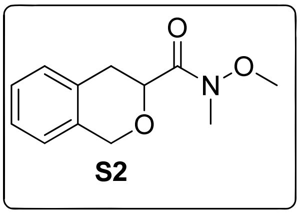

To a 150 mL round bottom flask containing 50 mL TFA (trifluoroacetic acid) was added 3-Phenyllactic acid (10.0 g, 60 mmol) and paraformaldehyde (2.0 g, 66.7 mmol, 1.1 eq.) successively. After stirring for 72 h at 90°C, the resulting reaction mixture was cooled to room temperature and the excess TFA was removed under vacuum. The obtained product was dissolved in dry CH_2Cl_2 (120 mL) and then the mixture was cooled to -15°C by ice-salt-bath, EDCI (14.0 g, 73 mmol), Me(MeO)NH·HCl (7.0 g,

72 mmol) and N-methyl morpholine (8.0 mL, 72 mmol) were added successively. The solution was stirred for 30 minutes before it was quenched with the saturated aqueous NH_4Cl (10 mL) at room temperature. The organic layer was separated and the aqueous layer was extracted with EtOAc/Et_2O (1:1) (2 × 100 mL). The combined organic layer was washed with brine (3 × 30 mL), dried over Na_2SO_4 and concentrated under vacuum. The residue was purified via column chromatography on silica gel (petroleum ether: ethyl acetate = 3:1) to give product **S2** as a faint yellow powder (10.1 g, 45.7 mmol, 76% yield). Mp: 91-92°C. ^{1}H NMR ($CDCl_3$, 400 MHz) δ 2.80 (dd, J= 16.0 Hz, 2.8 Hz, 1H), 3.07-3.19 (m, 4H), 3.66 (s, 3H), 4.57 (d, J= 8.8 Hz), 4.90 (d, J= 15.2 Hz, 1H), 4.79 (d, J= 15.2 Hz, 1H), 6.92 (t, J= 4.0 Hz, 1H), 7.05-7.12 (m, 3H); ^{13}C NMR ($CDCl_3$, 100 MHz) δ 29.5, 31.8, 61.1, 67.6, 71.3, 123.6, 125.7, 126.1, 128.3, 131.9, 133.4, 170.7. MS (EI) m/z (%): 61 (72), 77 (28), 91 (10), 105 (100), 133 (50) 161 (95).

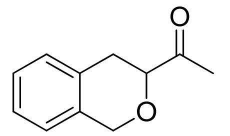

S3

To a stirred solution of **S2** (1.43 g, 6.5 mmol) in dry THF (20 mL) was added Methylmagnesium bromide (3.0 M in Et_2O, 2.4 mL) at 0°C under an argon atmosphere. After stirring at 0°C for 10 minutes, the reaction was quenched with saturated aqueous NH_4Cl (10 mL). The organic layer was separated and the aqueous layer was extracted with Et_2O (2 × 60 mL). The combined organic layer was washed with brine (3 × 10 mL), dried over Na_2SO_4 and concentrated under vacuum. The residue was purified by column chromatography on silica gel (petroleum ether: ethyl acetate = 20:1) to give product **S3** as a colorless oil (1.12 g, 6.4 mmol, 98% yield). ^{1}H NMR ($CDCl_3$, 400 MHz) δ 2.90 (s, 3H), 2.83-2.98 (m, 2H), 4.12 (dd, J= 10.8 Hz, 4.4 Hz, 1H), 4.93 (d, J= 14.8 Hz, 1H), 4.83 (dd, J= 15.2 Hz, 1H),6.97-7.01 (m, 1H), 7.10-7.19 (m, 3H); ^{13}C NMR ($CDCl_3$, 100 MHz) δ 25.7, 29.5, 67.9, 79.5, 123.9, 126.1, 126.5, 128.7, 131.6, 133.7, 208.0. MS (EI) m/z (%): 105 (100), 133 (64), 158 (53), 176 (M^+, 7).

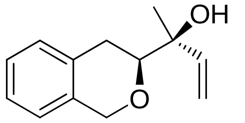

S4

To a stirred solution of **S3** (1.12 g, 6.4 mmol) in dry THF (20 mL) was added

vinylmagnesium bromide (0.7 M in THF, 11.0 mL, 77 mmol) at 0°C under an argon atmosphere. After stirring at 0°C for 10 minutes, the reaction was quenched with saturated aqueous NH_4Cl (10 mL). The organic layer was separated and the aqueous layer was extracted with Et_2O (2 × 80 mL). The combined organic layer was washed with brine (3 × 10 mL), dried over Na_2SO_4 and concentrated under vacuum. The residue was purified via column chromatography on silica gel (petroleum ether: ethyl acetate = 20:1) to give product compound **S4** as a colorless oil (773 mg, 3.8 mmol, 60% yield). ^{1}H NMR ($CDCl_3$, 400 MHz) δ 1.39 (s, 3H), 2.73 (dd, *J* = 16.4 Hz, 3.2 Hz, 1H), 2.79 (brs, 1H), 2.97 (dd, *J* = 16.4 Hz, 3.2 Hz, 1H), 3.60 (dd, *J* = 11.6 Hz, 3.2 Hz, 1H), 4.92 (d, *J* = 14.8 Hz, 1H), 4.87 (d, *J* = 15.2 Hz, 1H), 5.24 (dd, *J* = 6.8 Hz, 1.2 Hz, 1H), 5.46 (dd, *J* = 17.2 Hz, 1.2 Hz, 1H), 6.07 (dd, *J* = 17.6 Hz, 10.8 Hz, 1H), 7.00-7.02 (m, 1H), 7.14-7.21 (m, 3H); ^{13}C NMR ($CDCl_3$, 100 MHz) δ 22.6, 28.0, 68.5, 74.1, 80.1, 113.6, 123.9, 125.7, 126.3, 129.0, 133.0, 134.1, 142.2. MS (EI) m/z (%): 71 (14), 91 (12), 105 (100), 133 (28).

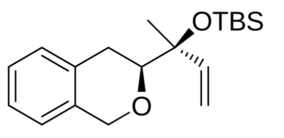

1a

To a stirred solution of **S4** (773 mg, 3.8 mmol) in dry CH_2Cl_2 (15 mL) was added Et_3N (630 μL, 4.6 mmol) and TBSOTf (952 μL, 4.0 mmol) successively at -78°C under an argon atmosphere, and then the mixture was warmed to room temperature spontaneously. The solution was stirred for 4 hours before it was quenched with the saturated aqueous $NaHCO_3$ (10 mL) at room temperature. The organic layer was separated and the aqueous layer was extracted with Et_2O (2 × 80 mL). The combined organic layer was washed with brine (3 × 10 mL), dried over Na_2SO_4 and concentrated under vacuum. The residue was purified via column chromatography on silica gel (petroleum ether: ethyl acetate = 100:1) to give product compound **1a** as a colorless oil (720 mg, 2.3 mmol, 60% yield). ^{1}H NMR ($CDCl_3$, 400 MHz) δ 0.22 (s, 3H), 0.24 (s, 3H), 1.04 (s, 9H), 1.58 (s, 3H), 2.89-2.91 (m, 2H), 3.60 (dd, *J* = 9.2 Hz, 5.6 Hz, 1H), 4.94 (d, *J* = 14.8 Hz, 1H), 4.84 (d, *J* = 14.8 Hz, 1H), 5.25 (dd, *J* = 10.4 Hz, 1.8 Hz, 1H), 5.45 (dd, *J* = 17.2 Hz, 2.2 Hz, 1H), 6.10 (dd, *J* = 17.2 Hz, 10.8 Hz, 1H), 7.04-7.06 (m, 1H), 7.19-7.24 (m, 3H); ^{13}C NMR ($CDCl_3$, 100 MHz) δ -1.93, -1.89, 18.5, 25.1, 25.2, 26.0, 26.1, 28.6, 68.8, 77.2, 82.0, 113.9 124.0, 125.7, 126.4, 129.2, 134.0, 134.8, 141.5. IR ν (cm^{-1}): 3065, 3024, 2931, 2856, 1463, 1253, 1103,

1043, 836, 775, 742. HRMS (ESI) calcd for $C_{19}H_{30}O_2SiNa$ $[M+Na]^+$: 341.1907, found 341.1914.

1.2 Synthesis of the substrate 1b

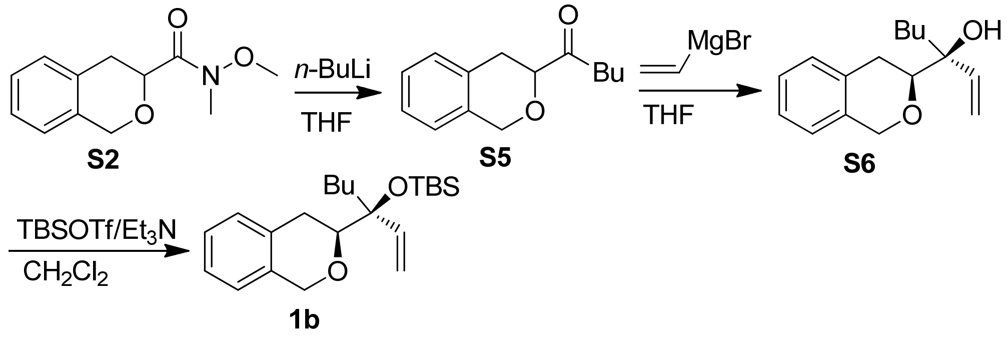

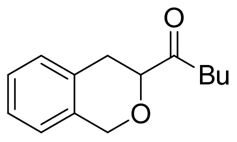

S5

Prepared according to the same procedure with **S3** from **S2** (1.96 g, 8.87 mmol) afforded **S5** as a colorless oil (1.19 g, 5.46 mmol, 62% yield).^{1}H NMR ($CDCl_3$, 400 MHz) δ 0.95 (t, J= 7.4 Hz, 3H), 1.32-1.41 (m, 2H), 1.58-1.66 (m, 2H), 2.69 (t, J= 7.2 Hz, 2H), 2.85-3.01 (m, 2H), 4.15 (dd, J= 10.8 Hz, 4.0 Hz, 1H), 4.85 (d, J= 14.8 Hz, 1H), 4.95 (d, J= 14.8 Hz, 1H), 7.00-7.02 (m, 1H), 7.13-7.20 (m, 3H); ^{13}C NMR ($CDCl_3$, 100 MHz) δ 13.8, 22.2, 25.0, 29.9, 37.7, 68.1, 79.5, 124.0, 126.2, 126.6, 128.9, 131.9, 133.9, 210.2. MS (EI) m/z (%): 57 (28), 85 (36), 105 (100), 133 (83), 162 (42).

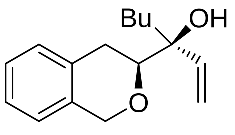

S6

Prepared according to the same procedure with **S4** from **S5** (997 mg, 4.57 mmol) afforded **S6** as a colorless oil (810 mg, 3.29 mmol, 72% yield). ^{1}H NMR ($CDCl_3$, 400 MHz) δ 0.95 (t, J= 6.8 Hz, 3H), 1.34-1.41 (m, 4H), 1.62-1.72 (m, 2H), 2.35 (brs, 1H), 2.73 (dd, J= 16.0 Hz, 3.0 Hz, 1H), 3.03 (dd, J= 16.0 Hz, 11.6 Hz, 1H), 3.64 (dd, J= 11.2 Hz, 3.2 Hz, 1H), 4.81 (dd, J= 14.8 Hz, 1H), 4.90 (dd, J= 14.8 Hz, 1H), 5.29 (dd, J= 10.8 Hz, 1.2 Hz, 1H), 5.43 (dd, J= 17.2 Hz, 1.2 Hz, 1H), 5.96 (dd, J= 17.2 Hz, 10.8 Hz, 1H), 6.99-7.01 (m, 1H), 7.14-7.19 (m, 3H); ^{13}C NMR ($CDCl_3$, 100 MHz) δ

14.0, 23.2, 25.2, 27.7, 35.6, 68.7, 76.7, 79.7, 114.3, 124.0, 125.9, 126.5, 129.2, 133.3, 134.3, 141.3. MS (EI) m/z (%): 55 (30), 91 (40), 105 (100), 119 (47), 133 (33).

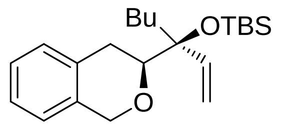

1b

Prepared according to the same procedure with **1a** from **S6** (700 mg, 2.85 mmol) afforded **1b** as a colorless oil (830 mg, 2.31 mmol, 81% yield). ^{1}H NMR ($CDCl_3$, 400 MHz) δ 0.19 (s, 3H), 0.23 (s, 3H), 1.00-1.03 (m, 12H), 1.41-1.44 (m, 4H), 1.85-1.86 (m, 2H), 2.83 (dd, J= 16.2 Hz, 2.6 Hz, 1H), 2.98 (dd, J= 16.2 Hz, 3.4 Hz, 1H), 3.69 (dd, J= 11.2 Hz, 3.2 Hz, 1H), 4.83 (d, J= 14.8 Hz, 1H), 4.92 (d, J= 14.8 Hz, 1H), 5.28 (dd, J= 10.8 Hz, 1.2 Hz, 1H), 5.45 (dd, J= 17.6 Hz, 1.2 Hz, 1H), 6.01 (dd, J= 17.2 Hz, 2.8 Hz, 1H), 7.03-7.05 (m, 1H), 7.18-7.23 (m, 3H); ^{13}C NMR ($CDCl_3$, 100 MHz) δ -2.1, 14.2, 18.9, 23.3, 25.6, 26.2, 28.5, 36.3, 68.5, 79.9, 80.0, 114.8, 124.0, 125.8, 126.4, 129.2, 133.9, 134.7, 140.7; IR ν (cm^{-1}): 3065, 3024, 2954, 2932, 2857, 1461, 1252, 1099, 1043, 835, 774, 743. HRMS (ESI) calcd for $C_{22}H_{37}O_2Si$ [M+H]$^+$: 361.2557, found 361.2550.

1.3 Synthesis of the substrate 1c

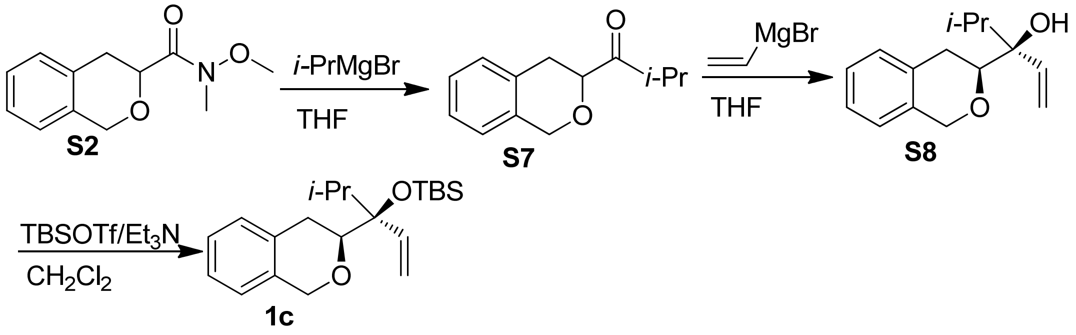

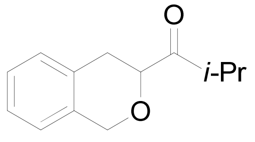

S7

Prepared according to the same procedure with **S3** from **S2** (1.92 g, 8.69 mmol) afforded **S7** as a colorless oil (1.58 g, 7.75 mmol, 89% yield). ^{1}H NMR ($CDCl_3$, 400 MHz) δ 1.15 (d, J = 6.8 Hz, 3H), 1.18 (d, J = 6.8 Hz, 3H), 2.88-3.03 (m, 2H), 3.16-3.26 (m, 1H), 4.24-4.28 (m, 1H), 4.86 (d, J= 15.2 Hz, 1H), 4.96 (d, J= 15.2 Hz, 1H), 7.01-7.02 (m, 1H), 7.13-7.20 (m, 3H); ^{13}C NMR ($CDCl_3$, 100 MHz) δ 17.8, 18.2,

30.0, 36.0, 68.2, 78.4, 124.0, 126.2, 126.6, 128.8, 132.0, 133.9, 213.4. MS (EI) m/z (%): 71 (55), 105 (100), 133 (86), 161 (50), 204 (M^+, 4).

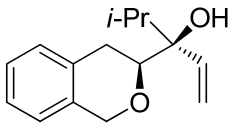

S8

Prepared according to the same procedure with **S4** from **S7** (1.33 g, 6.52 mmol) afforded **S8** as a colorless oil (1.46 g, 6.29 mmol, 96% yield). ^{1}H NMR ($CDCl_3$, 400 MHz) δ 0.91 (d, J = 6.8 Hz, 3H), 1.00 (d, J = 6.4 Hz, 3H), 2.06-2.12 (m, 1H), 2.19 (brs, 1H), 2.75 (dd, J = 16.4 Hz, 2.8 Hz, 1H), 3.10 (dd, J = 16.4 Hz, 11.2 Hz, 1H), 3.85 (dd, J = 11.2 Hz, 3.2 Hz, 1H), 4.83 (d, J = 14.8 Hz, 1H), 4.90 (d, J = 14.8 Hz, 1H), 5.37 (dd, J = 6.8 Hz, 1.6 Hz, 1H), 5.42 (dd, J = 17.4 Hz, 1.8 Hz, 1H), 5.97 (dd, J = 17.4 Hz, 11.0 Hz, 1H), 7.00-7.02 (m, 1H), 7.16-7.22 (m, 3H); ^{13}C NMR ($CDCl_3$, 100 MHz) δ 16.2, 17.2, 27.2, 32.0, 68.6, 77.4, 78.7, 115.1, 124.0, 125.8, 126.4, 129.3, 133.5, 134.3, 139.5. MS (EI) m/z (%): 55 (53), 91 (60), 105 (100), 119 (65), 131 (60), 187 (25).

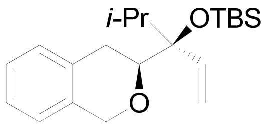

1c

Prepared according to the same procedure with **1a** from **S8** (1.36 g, 5.86 mmol) afforded **1c** as a colorless oil (1.68 g, 4.86 mmol, 83% yield). ^{1}H NMR ($CDCl_3$, 400 MHz) δ 0.15 (s, 3H), 0.23 (s, 3H), 0.94 (d, J = 6.8 Hz, 3H), 1.02 (s, 9H), 1.08 (d, J = 6.8 Hz, 3H), 2.12-2.18 (m, 1H), 2.78 (dd, J = 16.2 Hz, 3.0 Hz, 1H), 3.11 (dd, J = 16.0 Hz, 11.2 Hz, 1H), 3.94 (dd, J = 11.2 Hz, 3.4 Hz, 1H), 4.82 (d, J = 14.8 Hz, 1H), 4.92 (d, J = 14.8 Hz, 1H), 5.27-5.34 (m, 2H), 5.94 (dd, J = 17.6 Hz, 11.2 Hz, 1H), 7.06-7.07 (m, 1H), 7.20-7.25 (m, 3H); ^{13}C NMR ($CDCl_3$, 100 MHz) δ -2.0, -1.5, 17.0, 17.5, 19.2, 26.3, 27.7, 34.4, 68.1, 76.9, 82.6, 115.0, 124.0, 125.8, 126.3, 129.3, 133.9, 134.8, 139.4. IR ν (cm^{-1}): 3067, 3023, 2958, 2929, 2855, 1471, 1252, 1091, 1041, 834, 775, 741. HRMS (ESI) calcd for $C_{21}H_{35}O_2Si$ $[M+H]^+$: 347.2401, found 347.2398.

1.4 Synthesis of the substrate 1d

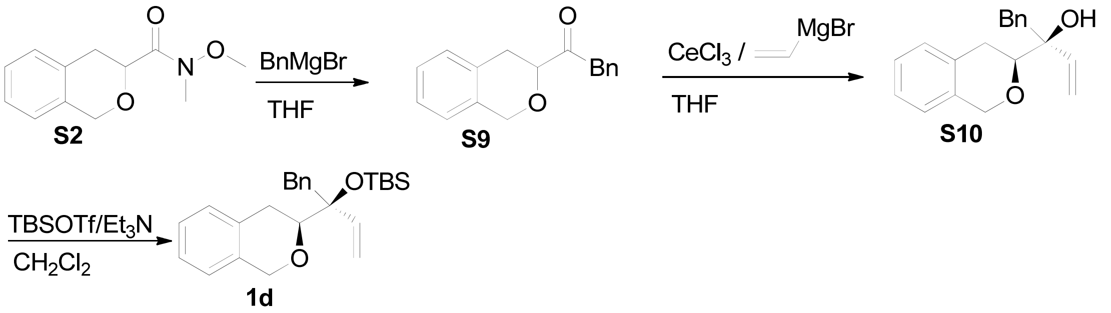

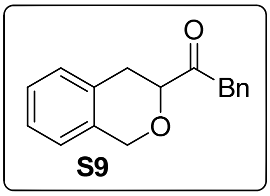

Prepared according to the same procedure with **S3** from **S2** (2.14 g, 9.68 mmol) afforded **S9** as a colorless oil (1.98 g, 7.86 mmol, 81% yield). ^{1}H NMR ($CDCl_3$, 400 MHz) δ 2.83-2.94 (m, 2H), 3.92 (d, J= 16.0 Hz, 1H), 4.00 (d, J= 16.0 Hz, 1H), 4.17 (dd, J= 10.0 Hz, 5.2 Hz, 1H), 4.79 (d, J= 14.8 Hz, 1H), 4.93 (d, J= 15.2 Hz, 1H), 6.95-6.97 (m, 1H), 7.06 -7.10(m, 1H), 7.12-7.25 (m, 7H); ^{13}C NMR ($CDCl_3$, 100 MHz) δ 29.7, 45.1, 68.1 78.8, 124.0 126.2, 126.6, 126.8, 128.4, 128.9, 129.6, 131.8, 133.6, 133.7, 207.4. MS (EI) m/z (%): 77 (22), 91 (56), 105 (100), 133 (74), 161 (67).

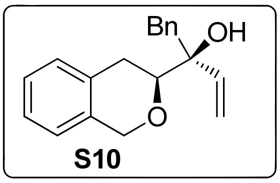

To a solution of vinylmagnesium bromide (0.7 M in THF, 10 mL, 70 mmol) was added anhydrous $CeCl_3$ (1.7 g, 6.9 mmol) at room temperature under an argon atmosphere and the mixture was stirred overnight. Then a solution of compounds **S9** (1.45 g, 5.75 mmol) in THF (2.0 mL) was added at 0°C and the mixture was stirred for 30 minutes at room temperature, then saturated aqueous NH_4Cl (5 mL) was added to quench the reaction. The organic layer was separated and the aqueous layer was extracted with Et_2O (2 × 80 mL). The combined organic layer was washed with brine (3 × 20 mL), dried over Na_2SO_4 and concentrated under vacuum. The residue was purified via column chromatography on silica gel (petroleum ether: ethyl acetate = 100:1) to give product compound **S10** as a colorless oil (720 mg, 2.6 mmol, 45% yield). ^{1}H NMR ($CDCl_3$, 400 MHz) δ 2.01 (s, 1H), 2.81 (dd, J= 16.4 Hz, 2.8 Hz, 1H), 2.99-3.06 (m, 2H), 3.23 (d, J= 13.6 Hz, 1H), 3.62 (dd, J= 11.4 Hz, 3.4 Hz, 1H), 4.86

(d, J= 14.8 Hz, 1H), 4.97 (d, J= 14.8 Hz, 1H), 5.31 (dd, J= 11.2 Hz, 1.2 Hz, 1H), 5.36 (dd, J= 17.4 Hz, 1.4 Hz, 1H), 6.17 (dd, J= 17.2 Hz, 10.8 Hz, 1H), 7.04-7.06 (m, 1H), 7.16-7.23 (m, 3H), 7.30-7.39 (m, 5H); ^{13}C NMR (CDCl$_3$, 100 MHz) δ 28.0, 43.6, 68.4, 78.4, 114.8, 124.0, 125.8, 126.4, 126.6, 128.1, 129.1, 130.7, 133.3, 134.3, 136.2, 140.1. MS (EI) m/z (%): 55 (20), 91 (53), 105 (100), 133 (42), 189 (40).

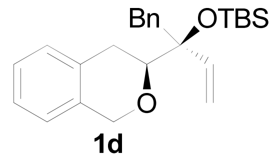

1d

Prepared according to the same procedure with **1a** from **S10** (640 mg, 2.29 mmol) afforded **1d** as a colorless oil (641 mg, 1.63 mmol, 71% yield). ^{1}H NMR (CDCl$_3$, 400 MHz) δ 0.15 (s, 3H), 0.17 (s, 3H), 0.90 (s, 9H), 2.78 (dd, J= 16.2 Hz, 2.6 Hz, 1H), 2.96-3.00 (m, 1H), 3.04 (d, J= 13.2 Hz,1H), 3.21 (d, J= 13.6 Hz, 1H), 3.58 (dd, J= 11.2 Hz, 3.2 Hz, 1H), 4.68 (d, J= 14.8 Hz, 1H), 4.83 (d, J= 15.2 Hz, 1H), 5.30 (d, J= 11.2 Hz, 1H), 5.44 (d, J = 17.6 Hz, 1H), 6.09 (dd, J = 17.6 Hz, 11.2 Hz, 1H), 6.96-6.98 (m, 1H), 7.10-7.15 (m, 3H), 7.18-7.31 (m, 5H); ^{13}C NMR (CDCl$_3$, 100 MHz) δ -1.9, -1.4, 18.8, 26.3, 28.2, 43.8, 68.1, 78.4, 79.3, 115.7, 124.0, 125.8, 126.1, 126.3, 127.5, 129.2, 131.3, 133.7, 134.7, 137.3, 140.6. IR ν(cm^{-1}): 3064, 3029, 2954, 2927, 2855, 1458, 1252, 1093, 1032, 834, 775, 744, 701. HRMS (ESI) calcd for $C_{25}H_{35}O_2Si$ [M+H]$^+$: 395.2401, found 395.2391.

1.5 Synthesis of the substrate 1e

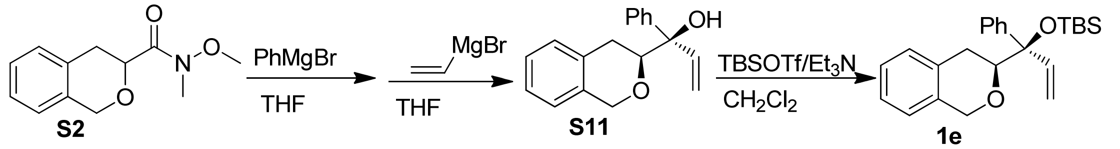

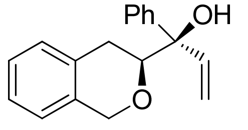

S11

To a stirred solution of **S2** (1.85 g, 8.37 mmol) in dry THF (20 mL) was added phenylmagnesium bromide (1.6 M in THF, 6.0 mL, 9.6 mmol) at 0°C under an argon atmosphere. After stirring at 0°C for 10 minutes, saturated aqueous NH$_4$Cl (10 mL) was added to quench the reaction. The organic layer was separated and the aqueous layer was extracted with Et$_2$O (2 × 80 mL). The combined organic layer was washed

with brine (3 × 10 mL), dried over Na_2SO_4 and concentrated under vacuum. The residue was dissolved in dry THF (20 mL), vinylmagnesium bromide (0.7 M in THF, 14.0 mL, 9.8 mmol) was added at 0°C under an argon atmosphere. After stirring at 0°C for 10 minutes, saturated aqueous NH_4Cl (10 mL) was added. The organic layer was separated and the aqueous layer was extracted with Et_2O (2 × 80 mL). The combined organic layer was washed with brine (3 × 10 mL), dried over Na_2SO_4 and concentrated under vacuum. The residue was purified via column chromatography on silica gel (petroleum ether: ethyl acetate = 20:1) to give compound **S11** as a colorless oil (1.65 g, 6.20 mmol, 74% yield). ^{1}H NMR ($CDCl_3$, 400 MHz) δ 2.19 (dd, J = 16.4 Hz, 2.0 Hz, 1H), 2.86 (brs, 1H), 2.98 (dd, J = 16.0 Hz, 11.6 Hz, 1H), 4.10 (dd, J = 11.2 Hz, 2.8 Hz, 1H), 4.92 (d, J = 15.2 Hz, 1H), 4.98 (d, J = 15.2 Hz, 1H), 5.29 (d, J = 10.8 Hz, 1H), 5.45 (d, J = 17.2 Hz, 1H), 6.41 (dd, J = 17.2 Hz, 10.8 Hz, 1H), 6.99-7.02 (m, 2H), 7.11-7.18 (m, 2H), 7.29-7.53 (m, 5H); ^{13}C NMR ($CDCl_3$, 100 MHz) δ 27.4, 68.7, 77.6, 79.1, 113.9, 123.9, 125.5, 125.8, 126.4, 127.0, 128.2, 129.2, 133.4, 133.8, 141.9, 142.7. MS (EI) m/z (%): 55 (46), 77 (41), 105 (100), 115 (13), 133 (33).

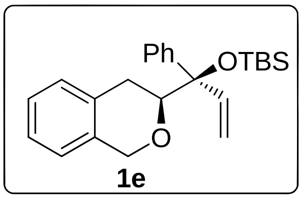

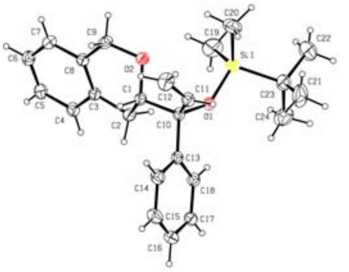

Prepared according to the same procedure with **1a** from **S11** (1.50 g, 5.64 mmol) afforded **1e** as a colorless crystal (1.23 g, 3.24 mmol, 57% yield). Mp: 58-59°C. ^{1}H NMR ($CDCl_3$, 400 MHz) δ 0.001 (s, 3H), 0.075 (s, 3H), 1.04 (s, 9H), 2.36 (dd, J = 16.6 Hz, 3.0 Hz, 1H), 3.08 (dd, J = 16.8 Hz, 11.2 Hz, 1H), 4.25 (dd, J = 11.0 Hz, 3.0 Hz, 1H), 4.92 (d, J = 14.8 Hz, 1H), 4.97 (d, J = 14.8 Hz, 1H), 5.40-5.45 (m, 2H), 6.37 (dd, J = 16.8 Hz, 11,4 Hz, 1H), 7.05-7.10 (m, 2H), 7.17-7.22 (m, 2H), 7.35-7.37 (m, 1H), 7.40-7.44 (m, 2H), 7.58-7.60 (m, 2H); ^{13}C NMR ($CDCl_3$, 100 MHz) δ -2.3, -2.2, 19.0, 26.3, 27.8, 68.2, 78.5, 80.9, 115.7, 124.0, 125.7, 126.3, 127.21, 127.24, 127.9, 129.3, 134.1, 134.5, 141.3, 142.8. IR ν (cm^{-1}): 3063, 3025, 2954, 2928, 2885, 1470, 1251, 1127, 1104, 1071, 835, 777, 742, 700. HRMS (ESI) calcd for $C_{24}H_{36}O_2SiN$ $[M+NH_4]^+$: 398.2510, found 398.2520. The crystal structure has been deposited at the Cambridge Crystallographic Data Centre and allocated the deposition number: 1000817.

1.6 Synthesis of the substrate 1f

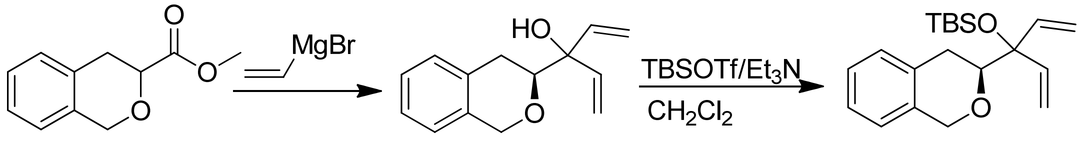

S12 S13 1f

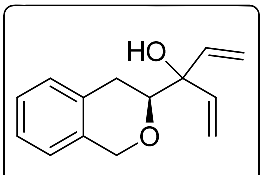

S13

To a stirred solution of **S12** (880 mg, 4.5 mmol) in dry THF (10 mL) was added vinylmagnesium bromide (0.7 M in THF, 14 mL, 9.8 mmol) at 0°C under an argon atmosphere. After stirring at 0°C for 30 minutes, saturated aqueous NH_4Cl (10 mL) was added. The organic layer was separated and the aqueous layer was extracted with Et_2O (2 × 80 mL). The combined organic layer was washed with brine (3 × 10 mL), dried over Na_2SO_4 and concentrated under vacuum. The residue was purified via column chromatography on silica gel (petroleum ether: ethyl acetate = 20:1) to give product **S13** as a colorless oil (580 mg, 2.7 mmol, 60% yield). ^{1}H NMR ($CDCl_3$, 400 MHz) δ 2.70-2.75 (m, 2H), 3.01 (dd, J= 16.4 Hz, 11.6 Hz, 1H), 3.71 (dd, J= 11.6 Hz, 3.4 Hz, 1H), 4.83 (d, J= 14.8 Hz, 1H), 4.93 (d, J= 14.8 Hz, 1H), 5.32 (dd, J= 10.8 Hz, 1.2 Hz, 2H), 5.50 (dd, J= 17.2 Hz, 1.2 Hz, 2H), 6.04-6.16 (m, 2H), 6.99-7.02 (m, 1H), 7.13-7.22 (m, 3H); ^{13}C NMR ($CDCl_3$, 100 MHz) δ 28.0, 68.6, 76.9, 79.5, 114.8, 115.1, 124.0, 125.9, 126.5, 129.1, 133.1, 134.0, 138.3, 139.6. MS (EI) m/z (%): 55 (50), 91 (69), 105 (100), 119 (90), 133 (52), 147 (45).

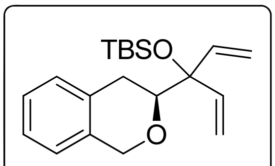

1f

Prepared according to the same procedure with **1a** from **S13** (690 mg, 3.2 mmol) afforded **1f** as a colorless oil (816 mg, 2.47 mmol, 77% yield). ^{1}H NMR ($CDCl_3$, 400 MHz) δ 0.136 (s, 3H), 0.144 (s, 3H), 0.98 (s, 9H), 2.77 (dd, J= 16.4 Hz, 3.2 Hz, 1H), 2.95 (dd, J= 16.6 Hz, 10.8 Hz, 1H), 3.71 (dd, J= 11.2 Hz, 3.2 Hz, 1H), 4.80 (d, J= 14.8 Hz, 1H), 4.91 (d, J= 14.8 Hz, 1H), 5.33-5.38 (m, 2H), 5.46 (dd, J= 10.8 Hz, 1.2 Hz, 1H), 5.51 (dd, J= 10.8 Hz, 1.4 Hz, 1H), 6.09 (dd, J= 17.4 Hz, 10.8 Hz, 1H), 6.17

(dd, J = 17.6 Hz, 10.8 Hz, 1H), 7.00-7.03 (m, 1H), 7.16-7.20 (m, 3H); ^{13}C NMR ($CDCl_3$, 100 MHz) δ -1.9, -1.8, 18.7, 26.1, 28.3, 68.6, 79.3, 80.4, 116.1, 117.1, 124.0, 125.7, 126.4, 129.2, 133.9, 134.6, 138.6, 140.9. IR ν (cm^{-1}): 3066, 3024, 2955, 2929, 2855, 1460, 1251, 1103, 1040, 925, 836, 776, 743. HRMS (ESI) calcd for $C_{20}H_{31}O_2Si$ $[M+H]^+$: 331.2088, found 331.2077.

1.7 Synthesis of the substrate 1g

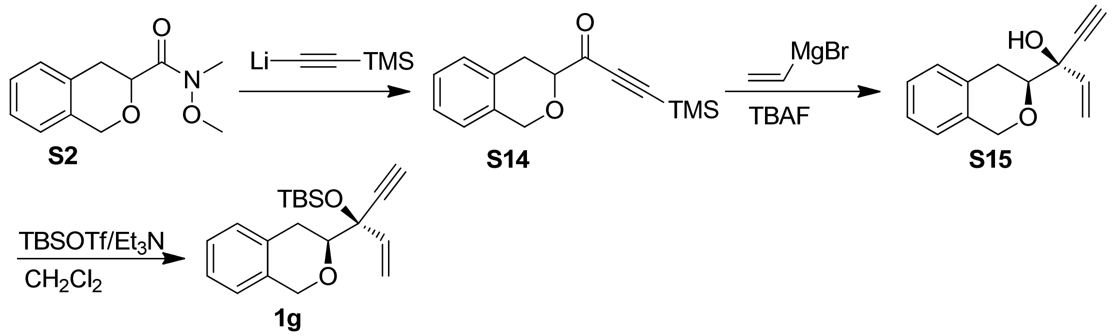

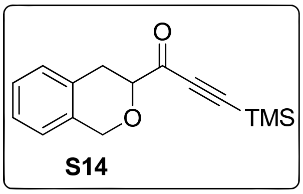

To a stirred solution of Trimethylsilylacetylene (586 μL, 4.5 mmol) in dry THF (10 mL) was added *n*-BuLi (2.5 M, 1.4 mL, 3.5 mmol) at -78°C under an argon atmosphere, and then the mixture was warmed to room temperature. After stirring at room temperature for 30 minutes, the system was cooled to -78°C again and **S2** (665 mg, 3.0 mmol) was added, then the mixture was warmed to RT spontaneously before it was quenched with saturated aqueous $NaHSO_4$ (20 mL). The organic layer was separated and the aqueous layer was extracted with Et_2O (2 × 60 mL). The combined organic layer was washed with brine (3 × 10 mL), dried over Na_2SO_4 and concentrated under vacuum. The residue was purified via column chromatography on silica gel (petroleum ether: ethyl acetate = 20:1) to give product **S14** as a colorless oil (410 mg, 1.59 mmol, 53% yield). ^1H NMR ($CDCl_3$, 400 MHz) δ 0.28 (s, 9H), 3.03 (dd, J= 16.0 Hz, 10.4 Hz, 1H), 3.13 (dd, J= 16.0 Hz, 4.0 Hz, 1H), 4.37 (dd, J= 10.4 Hz, 4.4 Hz, 1H), 4.87 (d, J= 15.2 Hz, 1H), 5.02 (d, J= 14.8 Hz, 1H), 7.00-7.03 (m, 1H), 7.15-7.21 (m, 3H); ^{13}C NMR ($CDCl_3$, 100 MHz) δ -1.0, 29.9, 67.7, 79.4, 99.8, 102.3, 124.1, 126.4, 126.6, 128.7, 131.2, 133.6, 185.0. MS (EI) m/z (%): 73 (19), 97 (20), 105 (100), 125 (25), 133 (52).

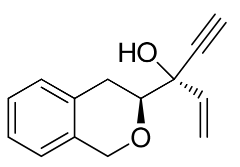

S15

To a stirred solution of **S14** (470 mg, 1.82 mmol) in dry THF (6 mL), vinylmagnesium bromide (0.7 M, 3.1 mL, 2.17 mmol) was added at 0°C under an argon atmosphere. After stirring at 0°C for 10 minutes, saturated aqueous NH_4Cl (10 mL) was added. The organic layer was separated and the aqueous layer was extracted with Et_2O (2 × 80 mL). The combined organic layer was washed with brine (3 × 10 mL), dried over Na_2SO_4 and concentrated under vacuum. The residue was dissolved in dry THF (6.0 mL), TBAF (523 mg, 2.0 mmol) was added at room temperature under an argon atmosphere. After stirring at 0°C for 10 minutes, saturated aqueous NaCl (10 mL) was added. The organic layer was separated and the aqueous layer was extracted with EtOAc/Et_2O (1:1) (2 × 80 mL). The combined organic layer was washed with brine (3 × 10 mL), dried over Na_2SO_4 and concentrated under vacuum. The residue was purified by column chromatography on silica gel (petroleum ether: ethyl acetate = 20:1) to give product **S15** as a colorless oil (362 mg, 1.69 mmol, 93% yield, dr = 5:1). (major product) ^{1}H NMR ($CDCl_3$, 400 MHz) δ 2.66 (s, 1H), 2.75 (dd, *J* = 16.4 Hz, 2.8 Hz, 1H), 3.11 (dd, *J* = 16.4 Hz, 11.6 Hz, 1H), 3.48 (brs, 1H), 3.68 (dd, *J* = 11.6 Hz, 3.2 Hz, 1H), 4.85-5.03 (m, 2H), 5.42 (dd, *J* = 11.4 Hz, 1.0 Hz, 1H), 5.81 (dd, *J* = 16.8 Hz, 1.0 Hz, 1H), 5.96 (dd, *J* = 16.8 Hz, 10.0 Hz, 1H), 7.01-7.03 (m, 1H), 7.13-7.20 (m, 3H); ^{13}C NMR ($CDCl_3$, 100 MHz) δ 28.9, 68.8, 73.8, 75.3, 79.8, 81.7, 118.2, 124.0, 124.1, 126.0, 126.1, 126.6, 126.7, 129.0, 129.1, 132.4, 133.8, 136.2. MS (EI) m/z (%): 77 (15), 105 (97), 117 (25), 133 (100), 149 (14).

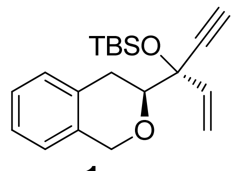

1g

Prepared according to the same procedure with **1a** from **S15** (290 mg, 1.36 mmol) afforded **1g** as a colorless oil (332 mg, 1.01 mmol, 75% yield). ^{1}H NMR ($CDCl_3$, 400 MHz) δ 0.24 (s, 3H), 0.27 (s, 3H), 0.96 (s, 9H), 2.69 (s, 1H), 2.83 (dd, *J* = 16.4 Hz, 2.8 Hz, 1H), 2.99 (dd, *J* = 16.4 Hz, 11.2 Hz, 1H), 3.75 (dd, *J* = 11.2 Hz, 3.2 Hz, 1H), 4.84 (d, *J* = 14.8 Hz, 1H), 4.97 (d, *J* = 14.4 Hz, 1H), 5.33 (dd, *J* = 10.4 Hz, 1.2 Hz,

1H), 5.67 (dd, J = 17.2 Hz, 1.2 Hz, 1H), 6.08 (dd, J = 17.2 Hz, 10.4 Hz, 1H), 7.01-7.03 (m, 1H), 7.17-7.20 (m, 3H); ^{13}C NMR ($CDCl_3$, 100 MHz) δ -2.94, -2.89, 18.4, 25.8, 28.3, 68.8, 74.2, 75.5, 81.3, 84.0, 116.0, 124.0, 125.8, 126.4, 129.1, 133.4, 134.6, 137.9. IR ν(cm^{-1}): 3305, 3065, 2954, 2929, 2856, 1461, 1252, 1098, 1033, 929, 839, 779, 745. HRMS (ESI) calcd for $C_{20}H_{29}O_2Si$ [M+H]$^+$: 329.1931, found 329.1925.

1.8 Synthesis of the substrate 1h/1h′

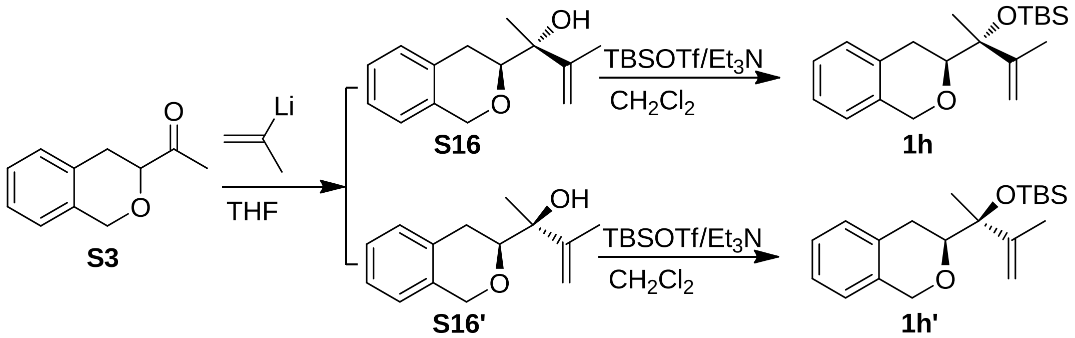

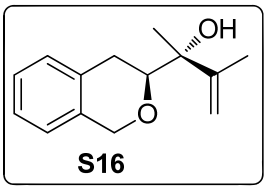

To a stirred solution of 2-bomopropylene (1.7 mL, 19.5 mmol) in dry THF (20 mL), *n*-BuLi (1.6 M, 9.8 mL, 15.7 mmol) was added at -78°C under an argon atmosphere. After stirring at -78°C for 30 minutes, **S3** (2.30 g, 13.0 mmol) in THF (2.0 mL) was added in one portion. After stirring for 10 minutes, saturated aqueous NH_4Cl (10 mL) was added to quench the reaction. The organic layer was separated and the aqueous layer was extracted with EtOAc/Et_2O (1:1) (2 × 100 mL). The combined organic layer was washed with brine (3 × 10 mL), dried over Na_2SO_4 and concentrated under vacuum. The residue was purified via column chromatography on silica gel (petroleum ether: ethyl acetate = 20:1) to give product **S16** as a colorless oil (1.01 g, 4.6 mmol, 35% yield), **S16′** as a colorless crystal (960 mg, 4.4 mmol, 34% yield). ^{1}H NMR ($CDCl_3$, 400 MHz) δ 1.52 (s, 3H), 1.84 (s, 3H), 2.55 (dd, J= 16.8 Hz, 3.0 Hz, 1H), 2.69 (brs, 1H), 3.03 (dd, J= 16.4 Hz, 11.6 Hz, 1H), 3.72 (dd, J= 11.2 Hz, 3.2 Hz, 1H), 4.86-5.01 (m, 3H), 5,26 (d, J= 0.8 Hz, 1H), 7.02-7.04 (m, 1H), 7.14-7.23 (m, 3H); ^{13}C NMR ($CDCl_3$, 100 MHz) δ 19.7, 25.3, 27.5, 68.8, 75.8, 78.5, 111.2, 123.8, 125.7, 126.4, 129.1, 133.7, 134.1, 146.5. MS (EI) m/z (%): 57 (22), 77 (19), 85 (23),

105 (100), 133 (32), 147 (9).

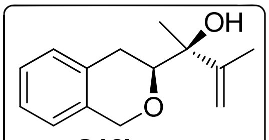

S16'

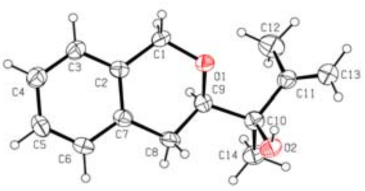

Mp: 57-59°C. ^{1}H NMR ($CDCl_3$, 400 MHz) δ 1.36 (s, 3H), 1.87 (s, 3H), 2.58 (s, 1H), 2.68 (dd, J= 16.4 Hz, 2.8 Hz, 1H), 3.06 (dd, J= 16.4 Hz, 11.4 Hz, 1H), 3.83 (dd, J= 11.4 Hz, 3.4 Hz, 1H), 4.83 (d, J= 14.8 Hz, 1H), 4.92 (d, J= 14.8 Hz, 1H), 5.01 (s, 1H), 5.20 (s, 1H), 7.00-7.02 (m, 1H), 7.15-7.21 (m, 3H); ^{13}C NMR ($CDCl_3$, 100 MHz) δ 19.5, 22.1, 27.8, 68.7, 76.3, 77.8, 111.1, 124.0, 125.9, 126.5, 129.2, 133.4, 134.2, 149.5. MS (EI) m/z (%): 57 (20), 77 (15), 85 (16), 105 (100), 133 (61). The crystal structure has been deposited at the Cambridge Crystallographic Data Centre and allocated the deposition number: 1000816.

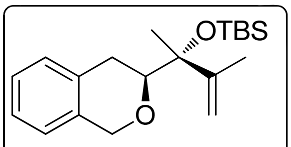

1h

Prepared according to the same procedure with **1a** from **S16** (364 mg, 1.67 mmol) afforded **1h** as a colorless oil (447 mg, 1.35 mmol, 81% yield). ^{1}H NMR ($CDCl_3$, 400 MHz) δ 0.18 (s, 3H), 0.19 (s, 3H), 0.98 (s, 9H), 1.55 (s, 3H), 1.87 (s, 3H), 2.68 (d, J= 16.4 Hz, 1H), 3.02 (dd, J= 16.2 Hz, 11.4 Hz, 1H), 3.73 (dd, J= 11.2 Hz, 3.2 Hz, 1H), 4.83 (d, J= 14.8 Hz, 1H), 4.96 (d, J= 14.8 Hz, 1H), 5.01 (d, J= 1.2 Hz, 1H), 5.25 (s, 1H), 7.04-7.05 (m, 1H), 7.20-7.21 (m, 3H); ^{13}C NMR ($CDCl_3$, 100 MHz) δ -2.4, -1.8, 18.7, 19.8, 22.7, 26.2, 27.7, 68.7, 78.7, 78.9, 111.8, 124.0, 125.6, 126.3, 129.2, 134.5, 134.8, 149.2. IR ν (cm^{-1}): 2951, 2932, 2856, 1734, 1459, 1252, 1122, 1098, 1034, 835, 775, 743. HRMS (ESI) calcd for $C_{20}H_{32}O_2SiK$ $[M+K]^+$: 371.1803, found 371.1812.

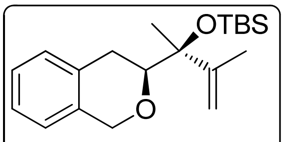

1h'

Prepared according to the same procedure with **1a** from **S16′** (960 mg, 4.4 mmol) afforded **1h′** as colorless oil (1.17 g, 3.5 mmol, 80% yield). ^{1}H NMR ($CDCl_3$, 400 MHz) δ 0.145 (s, 3H), 0.152 (s, 3H), 0.93 (s, 9H), 1.51 (s, 3H), 1.84 (s, 3H), 2.64 (dd, J= 16.4 Hz, 3.2 Hz, 1H), 2.74 (dd, J= 16.4 Hz, 11.2 Hz, 1H), 3.64 (dd, J= 11.2 Hz, 3.6 Hz, 1H), 4.78 (d, J= 14.8 Hz, 1H), 4.91 (d, J= 14.8 Hz, 1H), 4.95 (s, 1H), 5.11 (d,

J= 1.2 Hz, 1H), 7.00-7.02 (m, 1H), 7.12-7.18 (m, 3H); ^{13}C NMR ($CDCl_3$, 100 MHz) δ -2.3, -1.9, 18.6, 20.1, 22.6, 26.1, 28.8, 68.9, 79.5, 81.2, 111.8, 124.0, 125.7, 126.3, 129.2, 134.1, 134.8, 148.7. IR ν (cm^{-1}): 3062, 2953, 2931, 2855, 1459, 1144, 1101, 1032, 834, 774, 742. HRMS (ESI) calcd for $C_{20}H_{32}O_2SiNa$ $[M+Na]^+$: 355.2064, found 355.2076.

1.9 Synthesis of the substrate 1i

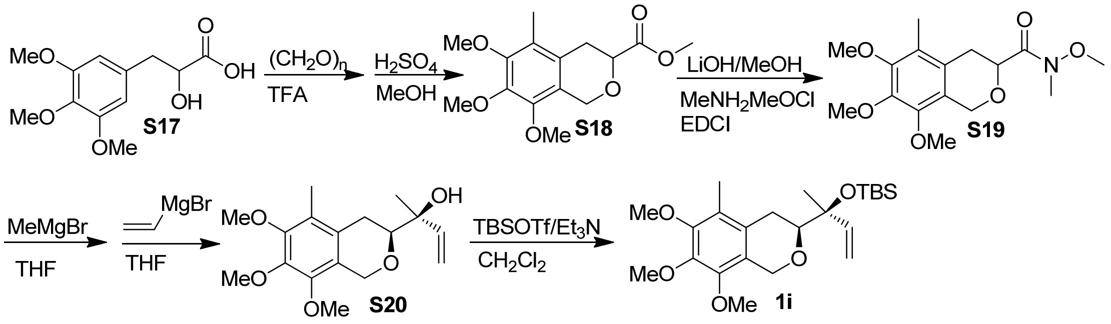

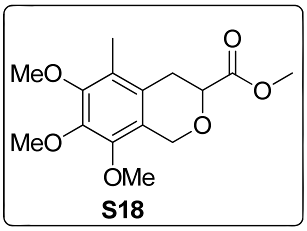

To a 100 mL round bottom flask containing 30 mL TFA (trifluoroacetic acid) was added **S17** (4.32 g, 16.9 mmol) and paraformaldehyde (558 mg, 18.6 mmol) successively. After stirring for 72 h at 90°C, the resulting reaction mixture was cooled to RT and the excess TFA was removed in vacuum. The obtained product was dissolved in 30 mL absolute MeOH and catalytic H_2SO_4 was added, then the mixture was heated to 80°C overnight, excess MeOH was removed under vacuum. Saturated aqueous $NaHCO_3$ (10 mL) and 30 mL Et_2O was added. The organic layer was separated and the aqueous layer was extracted with EtOAc/Et_2O (1:1) (2 × 100 mL). The combined organic layer was washed with brine (3 × 30 mL), dried over Na_2SO_4 and concentrated under vacuum. The residue was purified via column chromatography on silica gel (petroleum ether: ethyl acetate = 3:1) to give compound **S18** as a white solid (1.42 g, 4.80 mmol, 28% yield). Mp: 51-53°C. ^{1}H NMR ($CDCl_3$, 400 MHz) δ 2.10 (s, 3H), 2.76 (dd, J= 16.4 Hz, 10.8 Hz, 1H), 2.88 (dd, J= 16.4 Hz, 3.6 Hz, 1H), 3,80 (s, 3H), 3.82 (s, 3H), 3.83 (s, 3H), 3.87 (s, 3H), 4.28 (dd, J= 10.8 Hz, 4.0 Hz, 1H), 4.69 (d, J = 15.6 Hz, 1H), 5.03 (d, J = 15.6 Hz, 1H); ^{13}C NMR ($CDCl_3$, 100 MHz) δ 10.9, 28.5, 52.3, 60.4, 60.7, 64.7, 73.3, 122.6, 125.1, 126.0,

144.0, 146.8, 150.6, 171.5. MS (EI) m/z (%): 59 (85), 77 (62), 121 (50), 135 (28), 165 (43), 193 (30), 209 (100).

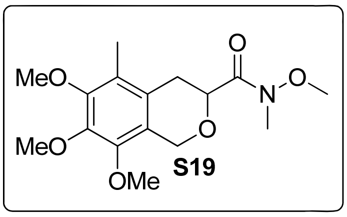

S19

To a stirred solution of **S18** (596 mg, 2.01 mmol) in absolute MeOH (10.0 mL), $LiOH\cdot H_2O$ (253 mg, 6.03 mmol) was added at room temperature under an argon atmosphere. After stirring at 0°C for 1 hour, aqueous HCl (2 M/L, 10 mL) was added. The organic layer was separated and the aqueous layer was extracted with EtOAc (2 × 60 mL). The combined organic layer was washed with brine (3×10 mL), dried over Na_2SO_4 and concentrated under vacuum. The obtained product was dissolved in 20 mL dry CH_2Cl_2 and the mixture was cooled to -15°C by ice-salt-bath, EDCI (460 mg, 2.4 mmol), Me(MeO)NH·HCl (234 mg, 2.4 mmol) and N-methyl morpholine (266 μL, 2.4 mmol) were added successively, 30 minutes later, saturated aqueous NH_4Cl (10 mL) was added. The organic layer was separated and the aqueous layer was extracted with EtOAc/Et_2O (1:1) (3 × 50 mL). The combined organic layer was washed with brine (3 × 20 mL), dried over Na_2SO_4 and concentrated under vacuum. The residue was purified via column chromatography on silica gel (petroleum ether: ethyl acetate = 20:1) to give product compound **S19** as a colorless oil (333 mg, 1.02 mmol, 51% yield). ^{1}H NMR ($CDCl_3$, 400 MHz) δ 2.10 (s, 3H), 2.70 (dd, J= 16.4 Hz, 2.8 Hz, 1H), 2.82-2.89 (m, 1H), 3.26 (s, 3H), 3.75 (s, 3H), 3.80 (s, 3H), 3.83 (s, 3H), 3.87 (s, 3H), 4.54 (d, J= 9.2 Hz, 1H), 4.71 (d, J= 15.2 Hz, 1H), 5.02 (d, J= 15.6 Hz, 1H); ^{13}C NMR ($CDCl_3$, 100 MHz) δ 10.9, 27.6, 32.1, 60.4, 60.67, 60.69, 61.5, 64.8, 71.6, 122.8, 125.2, 126.9, 143.8, 146.8, 150.5, 171.3. MS (EI) m/z (%): 195 (8), 209 (27), 235 (38), 265 (100).

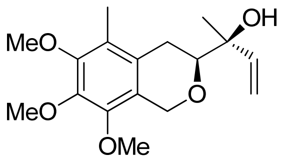

S20

Prepared according to the same procedure with **S11** from **S19** (330 mg, 1.02 mmol) afforded **S20** as a colorless oil (195 mg, 0.63 mmol, 62% yield). ^{1}H NMR ($CDCl_3$, 400 MHz) δ 1.35 (s, 3H), 2.11 (s, 3H), 2.49-2.66 (m, 3H), 3.49 (dd, J= 10.8 Hz, 3.6

Hz, 1H), 3.81 (s, 3H), 3.84 (s, 3H), 3.89 (s, 3H), 4.63 (d, J= 15.2 Hz, 1H), 4,99 (d, J= 15.2 Hz, 1H), 5.21 (dd, J= 10.8 Hz, 1.2 Hz, 1H), 5.42 (dd, J= 17.2 Hz, 1.2 Hz, 1H), 6.03 (dd, J= 17.2 Hz, 10.8 Hz, 1H); ^{13}C NMR ($CDCl_3$, 100 MHz) δ 11.0, 22.6, 25.9, 60.5, 60.7, 60.8, 65.3, 74.4, 79.9, 113.8, 123.4, 125.4, 127.6, 142.4, 143.7, 146.8, 150.5. MS (EI) m/z (%): 71 (10), 209 (100), 237 (15), 308 (M^+, 9).

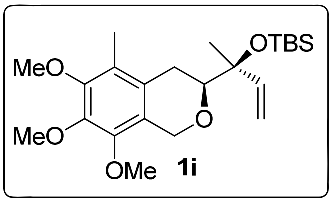

Prepared according to the same procedure with **1a** from **S20** (190 mg, 0.62 mmol) afforded **1i** as a colorless oil (236 mg, 0.56 mmol, 91% yield). ^{1}H NMR ($CDCl_3$, 400 MHz) δ 0.13 (s, 3H), 0.16 (s, 3H), 0.95 (s, 9H), 1.51 (s, 3H), 2.11 (s, 3H), 2,48 (dd, J = 16.8 Hz, 11.2 Hz, 1H), 2.69 (dd, J= 16.8 Hz, 2.4 Hz, 1H), 3.45 (dd, J= 11.2 Hz, 3.4 Hz, 1H), 3.83 (s, 3H), 3.85 (s, 3H), 3.90 (s, 3H), 4.61 (d, J= 15.2 Hz, 1H), 4.98 (d, J= 15.6 Hz, 1H), 5.18 (dd, J= 10.8 Hz, 2.00 Hz, 1H), 5.39 (dd, J= 17.2 Hz, 1.6 Hz, 1H), 6.01 (dd, J= 17.2 Hz, 10.8 Hz, 1H); ^{13}C NMR ($CDCl_3$, 100 MHz) δ -2.0, -1.9, 10.8, 18.5, 25.5, 25.9, 26.0, 26.3, 60.5, 60.7, 60.8, 65.3, 77.2, 81.8, 114.0, 123.8, 125.4, 128.5, 141.1, 143.6, 146.8, 150.4. IR ν (cm^{-1}): 3089, 3051, 2934, 2855, 1586, 1467, 1344, 1255, 1106, 1050, 1002, 836, 775, 740. HRMS (ESI) calcd for $C_{23}H_{38}O_5SiNa$ $[M+Na]^+$: 445.2381, found 445.2378.

1.10 Synthesis of the substrate 1j

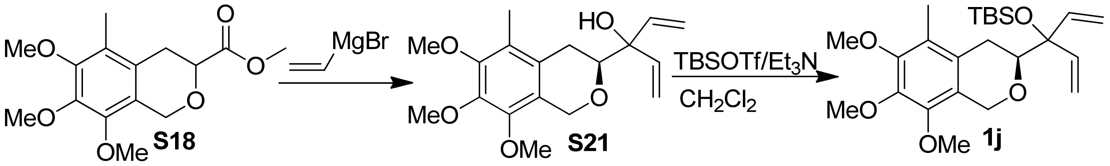

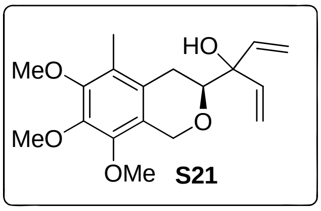

Prepared according to the same procedure with **S13** from **S18** (489 mg, 1.65 mmol) afforded **S21** as a colorless oil (255 mg, 0.80 mmol, 48% yield). ^{1}H NMR ($CDCl_3$, 400 MHz) δ 2.10 (s, 3H), 2.50 (dd, J= 16.6 Hz, 3.0 Hz, 1H), 2.62-2.69 (m, 2H), 3.60 (dd, J= 11.6 Hz, 3.4 Hz, 1H), 3.81 (s, 3H), 3.84 (s, 3H), 3.88 (s, 3H), 4.63 (d, J= 15.2 Hz, 1H), 5.00 (d, J= 15.2 Hz, 1H), 5.29 (d, J= 10.8 Hz, 2H), 5.47 (d, J= 17.2 Hz,

2H), 6.02-6.13 (m, 2H); ^{13}C NMR ($CDCl_3$, 100 MHz) δ 10.9, 25.8, 60.5, 60.7, 60.8, 65.3, 77.0, 79.4, 115.0, 115.3, 123.2, 125.5, 127.6, 138.3, 139.7, 143.8, 146.8, 150.6. MS (EI) m/z (%): 209 (100), 237 (35), 267 (9), 320 (M^+, 10).

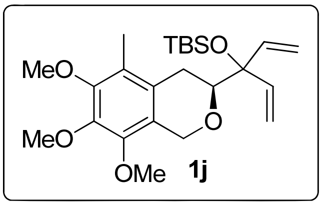

Prepared according to the same procedure with **1a** from **S21** (187 mg, 0.58 mmol) afforded **1j** as a colorless oil (183 mg, 0.42 mmol, 72% yield). ^{1}H NMR ($CDCl_3$, 400 MHz) δ 0.106 (s, 3H), 0.111 (s, 3H), 0.95 (s, 9H), 2.10 (s, 3H), 2.57-2.62 (m, 2H), 3.60 (dd, J= 10.40 Hz, 4.00 Hz, 1H), 3.82 (s, 3H), 3.84 (s, 3H), 3.89 (s, 3H), 4.60 (d, J= 15.2 Hz, 1H), 4.97 (d, J= 15.2 Hz, 1H), 5.32 (dd, J= 4.0 Hz, 1.8 Hz, 1H), 5.35 (dd, J= 3.6 Hz, 1.6 Hz, 2H), 5.44 (dd, J= 17.4 Hz, 1.4 Hz, 1H), 5.50 (dd, J= 17.2 Hz, 2.0 Hz, 1H), 6.06 (dd, J= 6.8 Hz, 2.4 Hz, 1H), 6.12 (dd, J= 14.8 Hz, 2.6 Hz, 1H); ^{13}C NMR ($CDCl_3$, 100 MHz) δ -1.9, -1.8, 10.9, 18.7, 26.1, 60.5, 60.7, 60.8, 65.2, 79.2, 80.3, 116.2, 117.3, 123.7, 125.4, 128.4, 138.2, 141.1, 143.6, 146.9, 150.5. IR ν(cm^{-1}): 3091, 2929, 2855, 1589, 1467, 1345, 1252, 1109, 1049, 1004, 931, 836, 777, 740. HRMS (ESI) calcd for $C_{24}H_{42}O_5SiN$ [M+ NH_4]$^+$: 452.2827, found 452.2824.

1.11 Synthesis of the substrate 1k

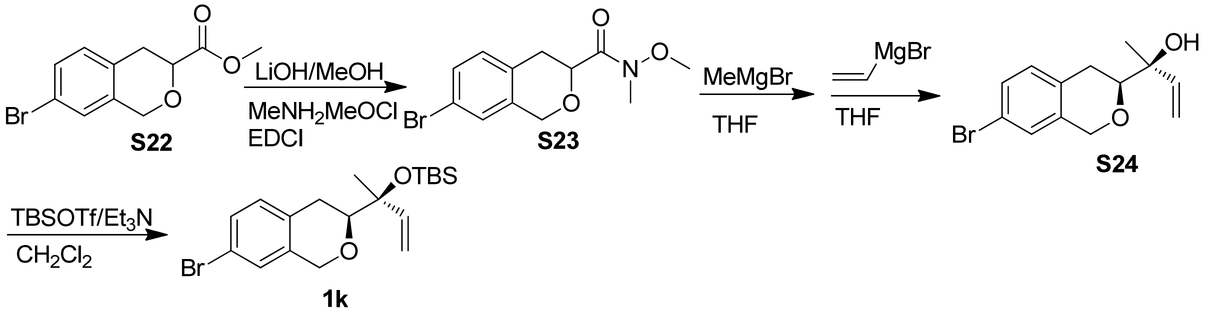

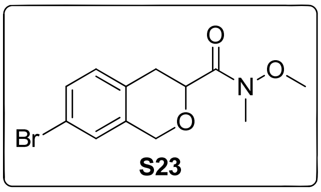

Prepared according to the same procedure with **S19** from **S22**[1] (see Supplementary Reference 1) (136 mg, 0.50 mmol) afforded **S23** as a white solid (130 mg, 88% yield). Mp: 109-112 ºC. ^{1}H NMR ($CDCl_3$,400 MHz) δ 2.83 (dd, J= 16.4 Hz, 3.6 Hz, 1H), 3.10 (dd, J= 16.0 Hz, 3.0 Hz, 1H), 3.26 (s, 3H), 3.76 (s, 3H), 4.61 (d, J= 8.8 Hz, 1H),

4.82 (d, J= 14.8 Hz, 1H), 4.94 (d, J= 14.8 Hz, 1H), 7.02 (d, J= 8.0 Hz, 1H), 7.17 (s, 1H), 7.30 (d, J= 8.0 Hz, 1H); ^{13}C NMR ($CDCl_3$, 100 MHz) δ 29.4, 32.3, 61.6, 67.4, 71.5, 119.8, 127.1, 129.7, 130.4, 131.4, 136.0, 170.8. IR ν (cm-1): 2922, 2853, 1658, 1594, 1115, 1094, 1056, 799. MS (EI) m/z (%): 301 ($[M+2]^+$, <1), 299 (M^+, <1), 241 (20), 239 (20), 61 (100). HRMS (ESI) calcd for $C_{12}H_{15}BrNO_3$ $[M+H]^+$: 300.0230, found 300.0239.

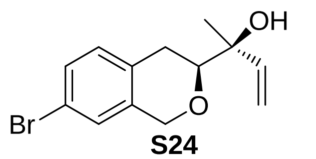

S24

Prepared according to the same procedure with **S11** from **S23** (134mg, 0.45 mmol) afforded **S24** as a clolrless oil (102 mg, 81% yield). ^{1}H NMR ($CDCl_3$, 400 MHz) δ 1.32 (s, 3H), 2.43 (s, 1H), 2.65 (dd, J= 16.0 Hz, 3.0 Hz, 1H), 2.85 (dd, J= 11.4 Hz, 3.2 Hz, 1H), 3.53 (dd, J= 11.4 Hz, 3.2 Hz, 1H), 4.75 (d, J= 15.2 Hz, 1H), 4.85 (d, J= 15.2 Hz, 1H), 5.20 (dd, J= 10.8 Hz, 1.2 Hz, 1H), 5.39 (dd, J= 17.2 Hz, 1.2 Hz, 1H), 6.00 (dd, J= 17.6 Hz, 10.8 Hz, 1H), 6.99 (d, J= 8.0 Hz, 1H), 7.14 (s, 1H), 7.28 (dd, J = 8.0 Hz, 1.8 Hz, 1H); ^{13}C NMR ($CDCl_3$, 100 MHz) δ 22.7, 27.7, 68.2, 74.3, 80.1, 113.9, 119.5, 127.0, 129.6, 130.8, 132.2, 136.4, 142.2. IR ν (cm-1): 3397, 2924, 2854, 1593, 1187, 1105, 927, 784. MS (EI) m/z (%): 284 ($[M+2]^+$, <1), 282 (M+, <1), 213 (30), 211 (30), 185(100), 183 (100). HRMS (ESI) calcd for $C_{13}H_{15}BrO_2Na$ $[M+Na]^+$: 305.0148, found 305.0156.

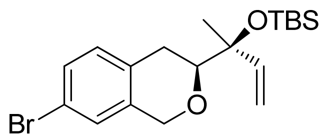

1k

Prepared according to the same procedure with **1a** from **S24** (208 mg, 0.74 mmol) afforded **1k** as a colorless oil (275 mg, 95% yield). ^{1}H NMR ($CDCl_3$, 400 MHz) δ 0.10 (s, 3H), 0.12 (s, 3H), 0.91 (s, 9H), 1.46 (s, 3H), 2.71-2.73 (m, 2H), 3.45 (dd, J= 8.4 Hz, 6.0 Hz, 1H), 4.69 (d, J= 15.2 Hz, 1H), 4.80 (d, J= 15.2 Hz, 1H), 5.15 (dd, J= 10.8 Hz, 1.6 Hz, 1H), 5.33 (dd, J= 17.2 Hz, 2.0 Hz, 2H), 6.97 (dd, J= 17.2 Hz, 10.8 Hz, 1H), 6.99 (d, J= 8.0 Hz, 1H), 7.12 (s, 1H), 7.26-7.27 (m, 1H); ^{13}C NMR ($CDCl_3$, 100 MHz) δ -1.97, -1.90, 18.5, 25.1, 26.0, 28.1, 68.2, 81.9, 114.1, 119.2, 127.0, 129.4, 130.9, 133.1, 136.9, 141.3. IR ν (cm-1): 2954, 2927, 2854, 1595, 1460, 1252, 1184, 1106, 836, 774. MS (EI) m/z (%): 341 ($[M+2-57]^+$, 30), 339 ($[M-57]^+$, <1), 185(100). HRMS (ESI) calcd for $C_{19}H_{29}BrO_2SiNa$ $[M+Na]^+$: 419.1012, found 419.1023.

1.12 Synthesis of the substrate 1m

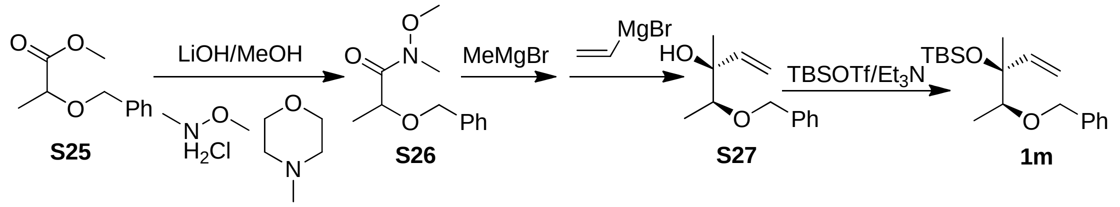

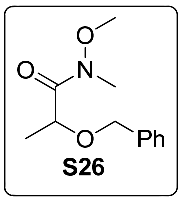

S26

Prepared according to the same procedure with **S19** from **S25** (8.0 g, 41.2 mmol) afforded **S26** as a colorless oil (6.73 g, 30.2 mmol, 73% yield). ^{1}H NMR ($CDCl_3$, 400 MHz) δ 1.39 (d, *J*= 6.80 Hz, 3H), 3.18 (s, 3H), 3.55 (s, 3H), 4.40 (d, *J*= 11.6 Hz, 2H), 4.65 (d, *J*= 11.6 Hz, 1H), 7.24-7.27 (m, 1H), 7.30-7.37 (m, 4H); ^{13}C NMR ($CDCl_3$, 100 MHz) δ 17.6, 32.0, 60.9, 70.8, 71.1, 127.4, 127.6, 128.0, 137.5, 173.2. MS (EI) m/z (%): 65 (14), 91 (100), 117 (39), 135 (8).

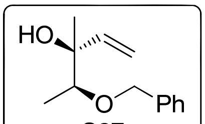

S27

Prepared according to the same procedure with **S11** from **S26** (2.33 g, 10.4 mmol) afforded **S27** as a colorless oil (1.35 g, 6.6 mmol, 66% yield). ^{1}H NMR ($CDCl_3$, 400 MHz) δ 2.20 (d, *J*= 6.4 Hz, 3H), 1.29 (s, 3H), 2.64 (brs, 1H), 3.45 (q, *J*= 6.4 Hz, 1H), 4.50 (d, *J*= 11.6 Hz, 1H), 4.69 (d, *J*= 11.6 Hz, 1H), 5.18 (dd, *J*= 10.8 Hz, 1.2 Hz, 1H), 5.38 (dd, *J* = 17.2 Hz, 3.4 Hz, 1H), 5.97 (dd, *J* = 17.2 Hz, 10.8 Hz, 1H), 7.30-7.40 (m, 5H); ^{13}C NMR ($CDCl_3$, 100 MHz) δ 13.8, 22.3, 71.4, 75.1, 80.6, 113.5, 127.49, 127.53, 128.2, 138.4, 142.6. MS (EI) m/z (%): 71 (34), 91 (100), 117 (14), 145 (10), 189 (18).

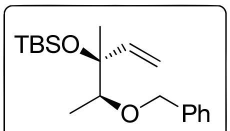

1m

Prepared according to the same procedure with **1a** from **S27** (1.30 g, 6.30 mmol) afforded **1m** as a colorless oil (1.50 g，4.69 mmol，74% yield). ^{1}H NMR ($CDCl_3$, 400 MHz) δ 0.20 (s, 3H), 0.21 (s, 3H), 1.04 (s, 9H), 1.25 (d, *J*= 6.0 Hz, 3H), 1.53 (s, 3H), 3.48 (q, *J*= 12.4 Hz, 6.0 Hz, 1H), 4.62 (d, *J*= 12.0 Hz, 1H), 4.76 (d, *J*= 12.0 Hz, 1H),

5.23 (dd, J= 10.8 Hz, 2.0 Hz, 1H), 5.39 (dd, J= 17.6 Hz, 2.0 Hz, 1H), 6.07 (dd, J= 17.6 Hz, 10.8 Hz, 1H), 7.35-7.38 (m, 1H), 7.39-7.46 (m, 4H); ^{13}C NMR ($CDCl_3$, 100 MHz) δ -2,0, -1.9, 14.4, 18.4, 24.5, 26.0, 71.7, 78.2, 82.8, 113.5, 127.3, 127.4, 128.2, 139.1, 141.9. IR ν (cm^{-1}): 3089, 3065, 2956, 2930, 2857, 1470, 1461, 1253, 1110, 1041, 835, 774. HRMS (ESI) calcd for $C_{19}H_{32}O_2SiNa$ [M+ Na]$^+$: 343.2064, found 343.2069.

1.13 Synthesis of the substrate 1n

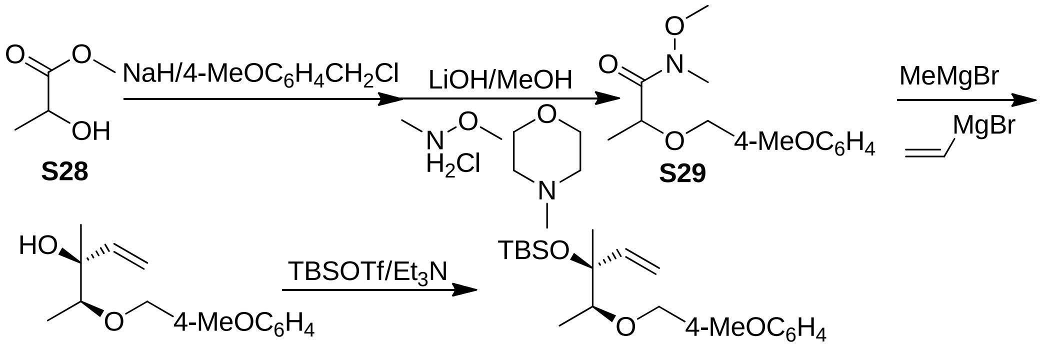

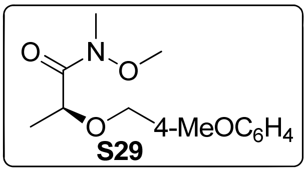

To a stirred solution of **S28** (5.2 g, 50 mmol) and 4-Methoxy benzyl chloride (8.6 g, 55 mmol) in dry DMF (50 mL) was added NaH (2.4 g, 60%, 60 mmol) slowly at 0°C, then the mixture was warmed to room temperature, 30minutes later, saturated aqueous NH_4Cl (20 mL) was added to the yellow mixture. The organic layer was separated and aqueous layer was extracted with Et_2O (2 × 120 mL). The combined organic layer was washed with brine (10 × 20 mL), dried over Na_2SO_4 and concentrated under vacuum. The crude product (11.0 g) was dissolved in absolute MeOH/H_2O (2:1/30 mL), LiOH·H_2O (6.02 g, 144 mmol) was added at room temperature under an argon atmosphere. After stirring at 0°C for 1 hour, aqueous HCl (2M/L, 10 mL) was added. The organic layer was separated and aqueous layer was extracted with EtOAc (2 × 120 mL). The combined organic layer was washed with brine (3 × 20 mL), dried over Na_2SO_4 and concentrated under vacuum. The obtained product was dissolved in 80 mL dry CH_2Cl_2 and the mixture was cooled to -15°C by ice-salt-bath, EDCI (11.3 g, 59 mmol), Me(MeO)NH·HCl (5.7 g, 58 mmol) and N-methyl morpholine (6.5 mL, 60 mmol) were added successively, 30 minutes later, saturated aqueous NH_4Cl (20 mL)

was added. The organic layer was separated and aqueous layer was extracted with EtOAc/Et_2O (1:1) (3 × 50 mL). The combined organic layer was washed with brine (3 × 10 mL), dried over Na_2SO_4 and concentrated under vacuum. The residue was purified via column chromatography on silica gel (petroleum ether: ethyl acetate = 3:1) to give compound **S29** as a colorless oil (6.42 g, 25.8 mmol, 52% yield). ^{1}H NMR ($CDCl_3$, 400 MHz) δ 7.29 (d, *J*= 8.4 Hz, 1H), 6.87 (d, *J*= 8.4 Hz, 1H), 4.59 (d, *J*= 11.6 Hz, 1H), 4.38-4.40 (m, 1H), 4.35 (d, *J*= 11.6 Hz, 1H), 3.79 (s, 3H), 3.59 (s, 3H), 3.20 (s, 3H), 1.38 (d, *J*= 6.8 Hz, 1H); ^{13}C NMR ($CDCl_3$, 100 MHz) δ 17.8, 32.2, 55.1, 61.2, 70.6, 71.0, 113.6, 129.5, 129.8, 159.4, 173.4. MS (EI) m/z (%): 77 (10) 87 (11), 117 (76), 121 (100), 135 (13).

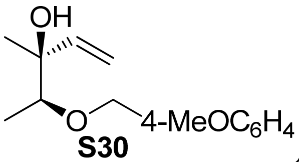

S30

Prepared according to the same procedure with **S11**from **S29** (1.11 g, 4.5 mmol) afforded **S30** as a colorless oil (808 mg, 3.4mmol, 76% yield). ^{1}H NMR ($CDCl_3$, 400 MHz) δ 7.24 (d, *J*= 8.4 Hz, 1H), 6.86 (d, *J*= 8.8 Hz, 1H), 5.90 (dd, *J*= 17.2 Hz, 10.8 Hz, 1H), 5.31 (dd, *J*= 17.2 Hz, 1.2 Hz, 1H), 5.11 (dd, *J*= 10.8 Hz, 1.2 Hz, 1H), 4.56 (d, *J*= 11.2 Hz, 1H), 4.37 (d, *J*= 11.2 Hz, 1H), 3.77 (s, 3H), 3.36 (q, *J*= 6.4 Hz, 1H), 2.61 (s, 1H), 1.21 (s, 3H), 1.13 (d, *J*= 6.4 Hz, 3H); ^{13}C NMR ($CDCl_3$, 100 MHz) δ 13.8, 22.3, 55.1, 71.0, 75.0, 80.2, 113.4, 113.6, 129.1, 130.5, 142.6, 159.1. MS (EI) m/z (%): 71 (9), 121 (100), 137 (6).

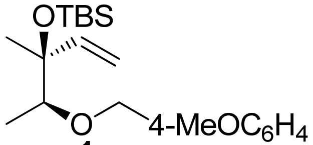

1n

Prepared according to the same procedure with **1a** from **S30** (648 mg, 2.8 mmol) afforded **1n** as a colorless oil (884 mg, 2.5 mmol, 90% yield). ^{1}H NMR ($CDCl_3$, 400 MHz) δ 7.32 (d, *J*= 8.4 Hz, 1H), 6.93 (d, *J*= 8.4 Hz, 1H), 6.00 (dd, *J*= 17.6 Hz, 10.8 Hz, 1H), 5.32 (d, *J*= 17.6 Hz, 1H), 5.16 (d, *J*= 10.8 Hz, 1H), 4.63 (d, *J*= 11.6 Hz, 1H), 4.50 (d, *J*= 11.6 Hz, 1H), 3.84 (s, 3H), 3.40 (q, *J*= 6.4 Hz, 1H), 1.45 (s, 3H), 1.17 (d, *J*= 6.4 Hz, 3H), 0.98 (s, 9H), 0.15 (s, 3H), 0.14 (s, 3H); ^{13}C NMR ($CDCl_3$, 100 MHz) δ -2.0, -1.9, 14.4, 18.4, 24.5, 26.0, 55.1, 71.4, 78.2, 81.9, 113.4, 113.6, 129.0, 131.2, 142.0, 159.0. IR *v* (cm^{-1}): 2956, 2932, 2856, 1613, 1513, 1463, 1249, 1104, 1039, 835, 774, 683. HRMS (ESI) calcd for $C_{20}H_{34}O_3SiNa$ [M+ Na]$^+$: 373.2169,

found 373.2174.

1.14 Synthesis of the substrate 1o

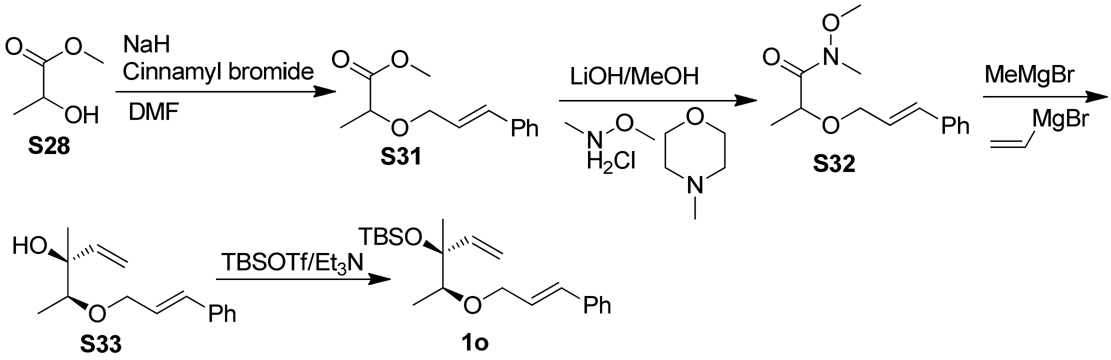

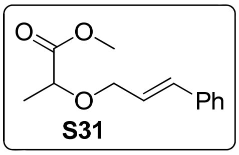

To a stirred solution of **S28** (2.5 g, 24 mmol) and Cinnamyl bromide (5.6 g, 29 mmol) in dry DMF (30.0 mL) was added NaH (1.44 g, 60%, 36 mmol) slowly at 0°C, then the mixture was warmed to room temperature, 30 minutes later, saturated aqueous NH_4Cl (20 mL) was added to the yellow mixture to quench the reaction. The organic layer was separated and aqueous layer was extracted with Et_2O (2 × 120 mL). The combined organic layer was washed with brine (10 × 20 mL), dried over Na_2SO_4 and concentrated under vacuum. The residue was purified by column chromatography on silica gel (petroleum ether: ethyl acetate = 15:1) to give product **S31** as a colorless oil (2.95 g, 13.4 mmol, 56% yield). ^{1}H NMR ($CDCl_3$, 400 MHz) δ 1.44 (d, *J* = 6.8 Hz, 3H), 3.74 (s, 3H), 4.06-4.14 (m, 2H), 4.26-4.31 (m, 1H), 6.25-6.33 (m, 1H), 6.61 (d, *J* = 16.0 Hz, 1H), 7.22-7.23 (m, 1H), 7.25-7.34 (m, 2H), 7.37-7.39 (m, 2H); ^{13}C NMR ($CDCl_3$, 100 MHz) δ 18.6, 51.9, 70.7, 73.8, 125.1, 126.4, 127.7, 128.4, 133.1, 136.3, 173.7. MS (EI) m/z (%): 55 (19), 77 (27), 105 (44), 117 (100), 133 (97).

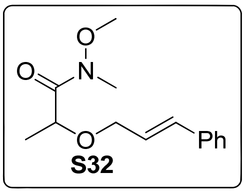

Prepared according to the same procedure with **S19** from **S31** (1.74 g, 7.91 mmol) afforded **S32** as a colorless oil (1.29 g, 5.18 mmol, 65% yield). ^{1}H NMR ($CDCl_3$, 400 MHz) δ 1.41 (d, *J* = 6.80 Hz, 3H), 3.18 (s, 3H), 3.65 (s, 3H), 4.09 (dd, *J* = 12.4 Hz, 6.8 Hz, 1H), 4.26 (dd, *J* = 12.4 Hz, 5.6 Hz, 1H), 4.45-4.47 (m, 1H), 6.28-6.35 (m, 1H),

6.60 (d, *J* = 16.0 Hz, 1H), 7.20-7.24 (m, 1H), 7.28-7.31 (m, 2H), 7.36-7.38 (m, 2H); ^{13}C NMR ($CDCl_3$, 100 MHz) δ 17.8, 32.0, 61.1, 69.9, 71.2, 125.5, 126.2, 127.5, 128.3, 132.7, 136.2, 173.6. MS (EI) m/z (%): 77 (14), 91 (21), 105 (16), 117 (100), 131 (40), 203 (10).

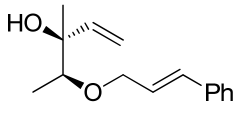

S33

Prepared according to the same procedure with **S11** from **S32** (1.17 g, 4.7 mmol) afforded **S33** as a colorless oil (730 mg, 3.15 mmol, 67% yield). ^{1}H NMR ($CDCl_3$, 400 MHz) δ 1.13 (d, *J* = 6.0 Hz, 3H), 1.24 (s, 3H), 2.66 (brs, 1H), 3.36 (q, *J* = 12.4 Hz, *J* = 6.4 Hz, 1H), 4.09 (dd, *J* = 12.4 Hz, 6.0 Hz, 1H), 4.27 (dd, *J* = 12.4 Hz, 5.8 Hz, 1H), 5.14 (d, *J* = 10.8 Hz, 1H), 5.34 (d, *J* = 17.2 Hz, 1H), 5.92 (dd, *J* = 17.6 Hz, 10.8 Hz, 1H), 6.23-6.60 (m, 1H), 6.58 (d, *J* = 16.0 Hz, 1H), 7.23-7.25 (m, 1H), 7.28-7.32 (m, 2H), 7.36-7.39 (m, 2H); ^{13}C NMR ($CDCl_3$, 100 MHz) δ 14.0, 22.1, 70.1, 75.0, 80.4, 113.6, 126.2, 126.4, 127.6, 128.4, 132.0, 136.5, 142.5. MS (EI) m/z (%): 71 (54), 91 (19), 117 (100), 133 (17).

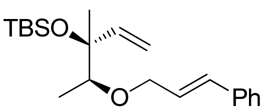

1o

Prepared according to the same procedure with **1a** from **S33** (730 mg, 3.15 mmol) afforded **1o** as a colorless oil (740 mg, 2.13 mmol, 68% yield). ^{1}H NMR ($CDCl_3$, 400 MHz) δ 0.21 (s, 6H), 1.03 (s, 9H), 1.23 (d, *J* = 6.0 Hz, 3H), 1.52 (s, 3H), 3.42 (q, *J* = 6.0 Hz, 1H), 4.17 (dd, *J* = 6.0 Hz, 1.2 Hz, 1H), 4.24 (dd, *J* = 6.0 Hz, 1.2 Hz, 1H), 4.34 (dd, *J* = 5.6 Hz, 1.2 Hz, 1H), 4.37 (dd, *J* = 5.6 Hz, 1.2 Hz, 1H), 5.22 (dd, *J* = 10.8 Hz, 2.0 Hz, 1H), 5.37 (dd, *J* = 17.2 Hz, 2.0 Hz, 1H), 6.05 (dd, *J* = 17.4 Hz, 2.6 Hz, 1H), 6.35-6.42 (m, 1H), 6,69 (d, *J* = 16.0 Hz, 1H), 7.30-7.49 (m, 5H); ^{13}C NMR ($CDCl_3$, 100 MHz) δ -2.0, -1.9, 14.6, 18.4, 24.2, 26.0, 70.6, 78.1, 82.2, 113.6, 126.4, 127.0, 127.4, 128.5, 131.4, 136.9, 141.9. IR ν (cm^{-1}): 3087, 3061, 3027, 2955, 2930, 2856, 1463, 1253, 1106, 1042, 835, 774, 692. HRMS (ESI) calcd for $C_{21}H_{34}O_2SiK$ [M+ K]$^+$: 385.1960, found 385.1976.

1.15 Synthesis of the substrate 1p

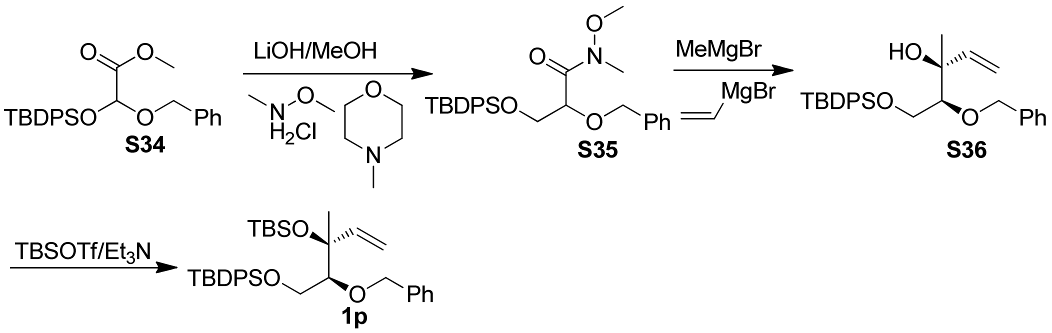

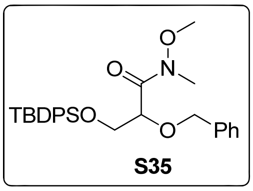

Prepared according to the same procedure with **S19** from **S34** (1.31 g, 3.02 mmol) afforded **S35** as a colorless oil (1.04 g, 2.17 mmol, 72% yield); ^{1}H NMR ($CDCl_3$, 400 MHz) δ 7.69-7.73 (m, 4H), 7.30-7.45 (m, 11H), 4.77 (d, *J*= 12.4 Hz, 1H), 4.57-4.60 (m, 2H), 3.99-4.00 (m, 2H), 3.51 (s, 3H), 3.20 (s, 3H), 1.09 (s, 9H); ^{13}C NMR ($CDCl_3$, 100 MHz) δ 19.1, 26.7, 32.2, 61.1, 64.4, 71.9, 76.4, 127.6, 127.9, 128.2, 129.6, 133.2, 135.5, 135.6, 137.7, 171.0. MS (EI) m/z (%): 91 (100), 163 (15), 252 (78), 420 (14).

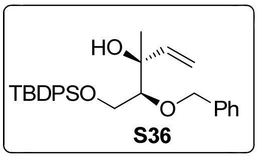

Prepared according to the same procedure with **S11** from **S35** (761 mg, 1.60 mmol) afforded **S36** as a colorless oil (630 mg, 1.37 mmol, 86% yield). ^{1}H NMR ($CDCl_3$, 400 MHz) δ 7.71-7.74 (m, 4H), 7.40-7.49 (m, 6H), 7.29-7.35 (m, 5H), 6.02 (dd, *J*= 17.6 Hz, 10.8 Hz, 1H), 5.44 (dd, *J*= 17.2 Hz, 1.6 Hz, 1H), 5.19 (dd, *J*= 10.4 Hz, 1.6 Hz, 1H), 4.71 (d, *J*= 11.6 Hz, 1H), 4.55 (d, *J*= 11.6 Hz, 1H), 3.79-3.89 (m, 2H), 3.49-3.52 (m, 2H), 1.32 (s, 3H), 1.11 (s, 9H); ^{13}C NMR ($CDCl_3$, 100 MHz) δ 19.0, 24.7, 26.8, 64.1, 73.7, 75.3, 84.0, 113.4, 127.67, 127.77, 127.81, 128.3, 129.9, 132.6, 132.8, 135.5, 135.6, 138.2, 142.0. MS (EI) m/z (%): 91 (100), 199 (22), 295 (63).

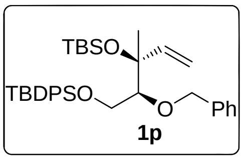

Prepared according to the same procedure with **1a** from **S36** (630 mg, 1.37 mmol) afforded **1p** as a colorless oil (732 mg, 1.28 mmol, 93% yield). ^{1}H NMR ($CDCl_3$, 400 MHz) δ 7.73-7.75 (m, 4H), 7.39-7.47 (m, 10H), 7.33-7.38 (m, 1H), 5.91 (dd, *J*= 17.6 Hz, 10.8 Hz, 1H), 5.16 (dd, *J*= 17.2 Hz, 1.6 Hz, 1H), 5.08 (d, *J*= 11.6 Hz, 1H), 4.99

(dd, J= 10.8 Hz, 1.6 Hz, 1H), 4.80 (d, J= 11.6 Hz, 1H), 4.07 (dd, J= 10.8 Hz, 2.0 Hz, 1H), 3.75 (dd, J= 10.8 Hz, 8.0 Hz, 1H), 3.55 (dd, J= 8.0 Hz, 1.8 Hz, 1H), 1.41 (s, 3H), 1.11 (s, 9H), 0.84 (s, 9H), 0.10 (s, 3H), 0.06 (s, 3H); ^{13}C NMR ($CDCl_3$, 100 MHz) δ -2.0, -1.9, 18.3, 19.2, 25.1, 25.9, 26.9, 65.8, 74.9, 87.9, 113.2, 127.2, 127.59, 127.62, 128.2, 129.48, 129.54, 133.63, 133.64, 135.7, 139.4, 141.9. IR ν (cm^{-1}): 3070, 2956, 2930, 2857, 1486, 1427, 1254, 1188, 1132, 1059, 1037, 835, 775, 703, 613, 504. HRMS (ESI) calcd for $C_{35}H_{50}O_3Si_2Na$ [M+ Na]$^+$: 597.3191, found 597.3188.

2.1 Preparation of benzoxa-[3. 2. 1] compounds (2a-2k)

All the benzoxa-[3.2.1] compounds **(2a-2k)** were synthesized according to the following general procedure (**2a** as an example)：To a solution of **1a** (249 mg, 0.78 mmol) in CH_2Cl_2 (8.0 mL) was added 4Å MS (400 mg), 2, 6-dibromopyridine (924 mg, 3.9 mmol, 5.0 eq.) and $InCl_3$ (18 mg, 0.078 mmol, 0.1 eq.) successively at room temperature under an argon atmosphere. After stirring for 15minutes, DDQ (361 mg, 98%, 1.56 mmol, 2.0 eq.) was added. The resulting brown mixture was stirred for 12 hours before it was filtered via a short silica gel column with petroleum ether: ethyl acetate = 4:1 as elute to remove the 4Å MS and 2, 6-dibromo-pyridine and the filtrate was concentrated under vacuum. The residue was chromatographed via column on silica gel (petroleum ether: ethyl acetate = 10:1) to give compound **2a** as a colorless oil (128 mg, 0.63 mmol, 81% yield).

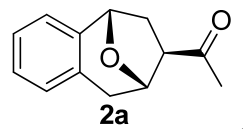

2a

^{1}H NMR ($CDCl_3$, 400 MHz) δ 2.22 (s, 3H), 2.25-2.41 (m, 2H), 2.59 (d, J= 16.8 Hz, 1H), 3.03-3.08 (m, 1H), 3.41 (dd, J= 16.8 Hz, 5.4 Hz, 1H), 4.94 (dd, J= 5.6 Hz, 1.6 Hz, 1H), 5.15 (d, J= 6.0 Hz, 1H), 7.00-7.02 (m, 1H), 7.08-7.10 (m, 1H), 7.13-7.22 (m, 2H); ^{13}C NMR ($CDCl_3$, 100 MHz) δ 28.2, 35.8, 40.2, 56.1, 75.6, 77.6, 124.2, 126.1, 127.4, 129.3, 130.7, 140.0, 207.4. IR ν (cm^{-1}): 3056, 2954, 2925, 1712, 1597, 1056, 757, 737. HRMS (ESI) calcd for $C_{13}H_{15}O_2$ [M+ H]$^+$: 203.1067, found 203.1065.

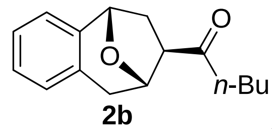

2b

Prepared according to general procedure from **1b** (364 mg, 1.01 mmol) afforded **2b** as a colorless oil (196 mg, 0.80 mmol, 80% yield, 12 hours). ^{1}H NMR ($CDCl_3$, 400 MHz)

δ 0.91 (t, J = 7.2 Hz, 3H), 1.30-1.35 (m, 2H), 1.55-1.63 (m, 2H), 2.25 (dd, J = 12.0 Hz, 9.0 Hz, 1H), 2.34-2.39 (m, 1H), 2.47 (t, J = 7.4 Hz, 1H), 2.59 (d, J = 16.8 Hz, 1H), 3.04-3.06 (m, 1H), 3.39 (dd, J = 16.4 Hz, 5.2 Hz, 1H), 4.90 (dd, J = 5.2 Hz, 1.2 Hz, 1H), 5.13 (d, J = 6.4 Hz, 1H), 7.00 (d, J = 2.0 Hz, 1H), 7.08 (d, J = 7.6 Hz, 1H), 7.14-7.21 (m, 2H); ^{13}C NMR ($CDCl_3$, 100 MHz) δ 13.8, 22.3, 25.8, 35.8, 40.3, 41.1, 55.1, 75.7, 77.6, 124.2, 126.0, 127.3, 129.2, 130.7, 140.0, 209.7. IR ν (cm^{-1}): 3067, 3020, 2956, 2932, 2871, 1711, 1604, 1061, 780, 759. HRMS (ESI) calcd for $C_{16}H_{21}O_2$ [M+ H]$^+$: 245.1536, found 245.1544.

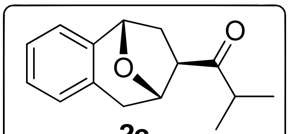

2c

Prepared according to the general procedure from **1c** (345 mg, 1.00 mmol) afforded **2c** as a colorless oil (190 mg, 0.83 mmol, 83% yield, 12 hours). ^{1}H NMR ($CDCl_3$, 400 MHz) δ 1.11 (d, J = 6.8 Hz, 3H), 1.12 (d, J = 6.8 Hz, 3H), 2.22 (dd, J = 11.6 Hz, 8.8 Hz, 1H), 2.35-2.41 (m, 1H), 2.60 (d, J = 16.4 Hz, 1H), 2.65-2.75 (m, 1H), 3.19-3.24 (m, 1H), 3.37 (dd, J = 16.4 Hz, 5.4 Hz, 1H), 4.83 (dd, J = 5.0 Hz, 1.8 Hz, 1H), 5.12 (d, J = 6.4 Hz, 1H), 6.99 (d, J = 7.2 Hz, 1H), 7.08 (d, J = 7.2 Hz, 1H), 7.12-7.21 (m, 2H); ^{13}C NMR ($CDCl_3$, 100 MHz) δ 18.25, 18.34, 35.7, 40.1, 40.6, 52.7, 76.0, 77.6, 124.1, 125.9, 127.2, 129.1, 130.6, 140.0, 213.5. IR ν (cm^{-1}): 3066, 3019, 2968, 2933, 2873, 1709, 1603, 1075, 1023, 761. HRMS (ESI) calcd for $C_{15}H_{19}O_2$ [M+H]$^+$: 231.1380, found 231.1378.

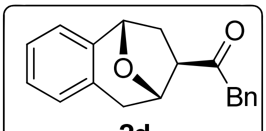

2d

Prepared according to general procedure from **1d** (358 mg, 0.91 mmol) afforded **2d** as a white solid (182 mg, 0.65 mmol, 72% yield, 10 hours). Mp: 118-120°C. ^{1}H NMR ($CDCl_3$, 400 MHz) δ 2.16 (dd, J = 11.60 Hz, 9.20 Hz, 1H), 2.34 (d, J = 16.80 Hz, 1H), 2.39-2.45 (m, 1H), 3.14 (dd, J = 8.20 Hz, 8.20 Hz, 1H), 3.27 (dd, J = 16.80 Hz, 5.20 Hz, 1H), 3.73 (d, J = 15.2 Hz, 1H), 3.78 (d, J = 14.8 Hz, 1H), 4.81 (d, J = 5.20 Hz, 1H), 5.11 (d, J = 6.40 Hz, 1H), 6.96 (d, J = 7.60 Hz, 1H), 7.02 (d, J = 7.60 Hz, 1H), 7.11-7.16 (m, 2H), 7.18-7.30 (m, 4H); ^{13}C NMR ($CDCl_3$, 100 MHz) δ 35.5, 40.3, 49.0, 54.3, 76.1, 77.6, 124.2, 126.0, 127.1, 127.3, 128.8, 129.2, 129.5, 130.6, 133.7, 139.9, 207.1. IR ν (cm^{-1}): 3061, 3032, 2965, 2940, 1714, 1700, 1604, 1028, 754, 736, 712. HRMS (ESI) calcd for $C_{19}H_{19}O_2$ [M+H]$^+$: 279.1380, found 279.1385.

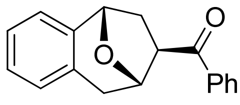

2e

Prepared according to the general procedure from **1e** (310 mg, 0.82 mmol) afforded **2e** as a colorless oil (160 mg, 0.61 mmol, 74% yield, 10 hours). ^{1}H NMR (CDCl$_3$, 400 MHz) δ 2.37 (dd, J = 12.0 Hz, 9.2 Hz, 1H), 2.55-2.60 (m, 1H), 2.76 (d, J = 6.8 Hz, 1H), 3.46 (dd, J = 16.8 Hz, 5.2 Hz, 1H), 3.85-3.90 (m, 1H), 5.06 (dd, J = 5.2 Hz, 2.0 Hz, 1H), 5.20 (d, J = 6.4 Hz, 1H), 7.05 (d, J = 6.8 Hz, 1H), 7.14-7.26 (m, 3H), 7.46-7.50 (m, 2H), 7.56-7.60 (m, 1H), 7.95-7.97 (m, 2H); ^{13}C NMR (CDCl$_3$, 100 MHz) δ 35.5, 41.3, 50.6, 76.3, 77.8, 124.2, 126.1, 127.4, 128.5, 128.7, 129.3, 130.7, 133.2, 136.3, 140.4, 199.1. IR ν (cm^{-1}): 3061, 2955, 2924, 2853, 1702, 1687, 1600, 1459, 1266, 1255, 1117, 740. HRMS (ESI) calcd for $C_{18}H_{17}O_2$ [M+H]$^+$: 265.1223, found 265.1226.

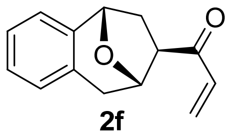

2f

Prepared according to the general procedure from **1f** (350 mg, 1.06 mmol) afforded **2f** as a colorless oil (144 mg, 0.67 mmol, 63% yield, 12 hours). ^{1}H NMR (CDCl$_3$, 400 MHz) δ 2.27 (dd, J = 12.0 Hz, 9.2 Hz, 1H), 2.41-2.47 (m, 1H), 2.63 (d, J = 16.8 Hz, 1H), 3.28-3.32 (m, 1H), 3.42 (dd, J = 16.8 Hz, 5.2 Hz, 1H), 4.94 (dd, J = 5.2 Hz, 1.2 Hz, 1H), 5.16 (d, J = 6.4 Hz, 1H), 5.87 (dd, J = 10.8 Hz, 1.0 Hz, 1H), 6.29 (dd, J = 17.6 Hz, 1.2 Hz, 1H), 6.48 (dd, J = 17.6 Hz, 10.4 Hz, 1H), 7.02 (d, J = 7.2 Hz, 1H), 7.10 (d, J = 7.6 Hz, 1H), 7.15-7.23 (m, 2H); ^{13}C NMR (CDCl$_3$, 100 MHz) δ 35.6, 40.4, 52.6, 75.9, 77.6, 124.2, 126.1, 127.4, 129.16, 129.21, 130.6, 134.9, 140.1, 199.1. IR ν (cm^{-1}): 3057, 3020, 2953, 2928, 1611, 1060, 1026, 975, 758, 734; HRMS (ESI) calcd for $C_{14}H_{15}O_2$ [M+H]$^+$: 215.1067, found 215.1065.

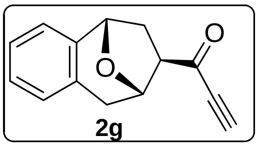

2g

Prepared according to the general procedure from **1g** (140 mg, 0.43 mmol) afforded **2g** as a colorless oil (21 mg, 0.10 mmol, 23% yield, 12 hours). ^{1}H NMR (CDCl$_3$, 400 MHz) δ 2.29 (dd, J = 12.0 Hz, 9.0 Hz, 1H), 2.58 -2.62 (m, 1H), 2.66 (d, J = 16.4 Hz, 1H), 3.15-3.19 (m, 1H), 3.33 (s, 1H), 3.44 (dd, J = 16.8 Hz, 5.2 Hz, 1H), 5.09 (dd, J = 5.2 Hz, 1.2 Hz, 1H), 5.18 (d, J = 6.8 Hz, 1H), 7.02 (d, J = 7.2 Hz, 1H), 7.10 (d, J = 7.6

Hz, 1H), 7.14-7.23 (m, 2H); ^{13}C NMR ($CDCl_3$, 100 MHz) δ 35.7, 39.6, 57.6, 76.0, 77.7, 80.3, 80.4, 124.3, 126.2, 127.6, 129.3, 130.3, 139.7, 186.5. IR ν (cm^{-1}): 3290, 3055, 3022, 2956, 2926, 2853, 2094, 1681, 1121, 1026, 975, 739, 704. HRMS (ESI) calcd for $C_{14}H_{16}O_2N$ $[M+NH_4]^+$: 230.1176, found 230.1178.

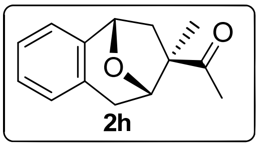

2h

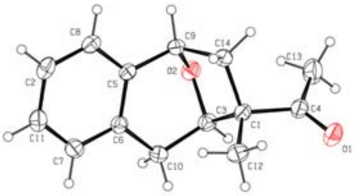

Prepared according to the general procedure from a mixture of **1h** and **1h′** (555 mg, 1.67 mmol) afforded **2h** as a colorless crystal (301 mg, 1.39 mmol, 83% yield, 6 hours). Mp: 129-131°C. ^{1}H NMR ($CDCl_3$, 400 MHz) δ 1.18 (s, 3H), 1.72 (d, J = 12.4 Hz, 1H), 2.27 (s, 3H), 2.75-2.79 (m, 2H), 3.32 (dd, J = 17.4 Hz, 5.8 Hz, 1H), 4.84 (d, J = 6.0 Hz, 1H), 5.06 (d, J = 7.2 Hz, 1H), 6.96 (d, J = 7.2 Hz, 1H), 7.06-7.19 (m, 3H); ^{13}C NMR ($CDCl_3$, 100 MHz) δ 20.5, 24.9, 30.5, 46.0, 57.7, 77.1, 78.4, 123.8, 126.0, 127.3, 128.4, 131.2, 140.8, 209.8. IR ν (cm^{-1}): 3058, 3020, 2983, 2953, 2898, 1702, 1456, 1426, 1050, 996, 761, 737. HRMS (ESI) calcd for $C_{14}H_{17}O_2$ $[M+H]^+$: 217.1223, found 217.1225. The crystal structure has been deposited at the Cambridge Crystallographic Data Centre and allocated the deposition number: 1000811.

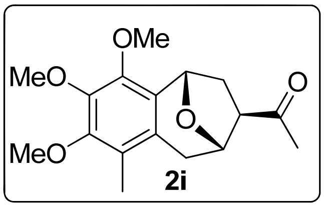

2i

Prepared according to the general procedure from **1i** (71 mg, 0.17 mmol) afforded **2i** as a white solid (43 mg, 0.14 mmol, 83% yield, 6 hours). Mp: 87-89°C. ^{1}H NMR ($CDCl_3$, 400 MHz) δ 2.03 (s, 3H), 2.21 (s, 3H), 2.24-2.38 (m, 3H), 2.97-3.01 (m, 1H), 3.09 (dd, J = 16.8 Hz, 5.2 Hz, 1H), 3.79 (s, 3H), 3.88 (s, 6H), 4.94-4.95 (m, 1H), 5.42 (d, J = 5.6 Hz, 1H); ^{13}C NMR ($CDCl_3$, 100 MHz) δ 10.9, 28.1, 34.2, 39.8, 56.4, 60.6, 60.7, 60.9, 72.6, 74.9, 125.0, 125.9, 128.7, 143.9, 146.3, 151.0, 207.7. IR ν (cm^{-1}): 3054, 2936, 2835, 1713, 1467, 1421, 1362, 1338, 1105, 1045, 972, 736. HRMS (ESI) calcd for $C_{17}H_{23}O_5$ $[M+H]^+$: 307.1540, found 307.1536.

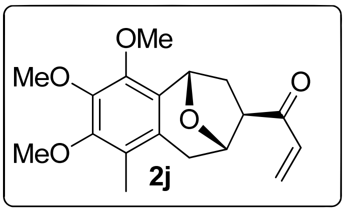

2j

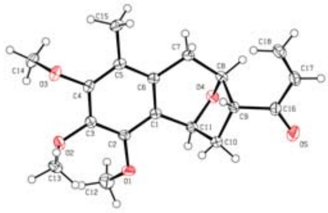

Prepared according to the general procedure from **1j** (74 mg, 0.17 mmol) afforded **2j** as a white solid (47 mg, 0.15 mmol, 87% yield, 6 hours). Mp: 114-115°C. ^{1}H NMR ($CDCl_3$, 400 MHz) δ 2.05 (s, 3H), 2.26 (dd, J = 12.0 Hz, 9.2 Hz, 1H), 2.36-2.42 (m, 2H), 3.11 (dd, J = 16.8 Hz, 5.6 Hz, 1H), 3.21-3.25 (m, 1H), 3.80 (s, 3H), 3.88 (s, 6H), 4.95 (d, J = 5.6 Hz, 1H), 5.44 (d, J = 6.4 Hz, 1H), 5.86 (dd, J = 10.6 Hz, 1.0 Hz, 1H), 6.29 (dd, J = 17.4 Hz, 1.0 Hz, 1H), 6.48 (dd, J = 17.4 Hz, 10.6 Hz, 1H); ^{13}C NMR ($CDCl_3$, 100 MHz) δ 10.9, 34.2, 40.0, 53.1, 60.6, 60.7, 60.9, 72.6, 75.3, 124.9, 125.9, 128.9, 129.2, 134.9, 144.0, 146.3, 151.0, 199.3. IR ν (cm^{-1}): 3054, 2926, 2853, 1699, 1466, 1265, 1105, 1045, 739, 704. HRMS (ESI) calcd for $C_{18}H_{23}O_5$ [M+H]$^+$: 319.1540, found 319.1543. The crystal structure has been deposited at the Cambridge Crystallographic Data Centre and allocated the deposition number: 1000827.

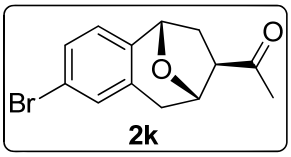

2k

Prepared according to the general procedure from **1k** (66.8 mg, 0.17 mmol) afforded **2k** as a white solid (30 mg, 63% yield, 6 hours). Mp: 62-64°C. ^{1}H NMR ($CDCl_3$, 400 MHz) δ 2.21 (s, 3H), 2.27-2.30 (m, 1H), 2.35-2.38 (m, 1H), 2.54 (d, J = 16.8 Hz 1H), 2.99-3.04 (m, 1H), 3.32 (dd, J = 16.8 Hz, 5.2 Hz, 1H), 4.94 (dd, J = 5.2 Hz, 1.6 Hz, 1H), 5.10 (d, J = 8.4 Hz, 1H), 6.97 (d, J = 8.0 Hz, 1H), 7.16 (d, J = 2.0 Hz, 1H), 7.32 (dd, J = 8.0 Hz, 2.0 Hz, 1H); ^{13}C NMR ($CDCl_3$, 100 MHz) δ 28.2, 35.3, 40.1, 55.9, 75.4, 77.1, 119.6, 127.2, 129.7, 130.5, 131.0, 142.0, 206.9. IR ν (cm-1): 3082, 2923, 2852, 1713, 1482, 1363, 1105, 1173, 792. MS (EI) m/z (%): 282 ([M+2]$^+$, 30), 280 ([M]$^+$, 30), 211(40), 209 (40), 185 (100). HRMS (ESI) calcd for $C_{13}H_{13}BrO_2Na$ [M+Na]$^+$: 302.9991, found 302.9998.

2.2 Preparation of multi-substitued tetrahydronfurans (2m-2p)

All the multi-substitued tetrahydronfurans (**2m-2p**) were synthesized via the following general procedure (**2m** as an example)：To a solution of **1m** (253 mg, 0.79 mmol) in CH_2Cl_2 (8.0 mL) was added 4Å molecular sieve (400 mg), 2, 6-dibromopyridine (936 mg, 3.95 mmol, 5.0 eq.) and $InCl_3$ (18 mg, 0.079 mmol, 0.1

eq.) successively at room temperature under an argon atmosphere, 15 minutes later, DDQ (200 mg, 98%, 0.87 mmol, 1.1 eq.) was added in one potion. The resulting mixture was stirred for 12 hours at room temperature before it was filtered via a short silica gel column with petroleum ether: ethyl acetate = 4:1 as eluent to separate the 4Å molecular sieve and 2, 6-dibromopyridine, the filtrate was concentrated under vacuum. The residue was purified via column chromatography on silica gel (petroleum ether: ethyl acetate = 10:1) to give product **2m** as a colorless oil (85 mg, 0.42 mmol, 53% yield) and **2m′** (30 mg, 0.15 mmol, 19% yield).

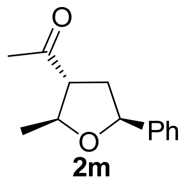

2m

^{1}H NMR ($CDCl_3$, 400 MHz) δ 1.25 (d, *J* = 6.40 Hz, 3H), 2.21 (s, 3H), 2.24-2.37 (m, 2H), 3.45 (dd, *J* = 16.8 Hz, 8.4 Hz, 1H), 4.46-4.49 (m, 1H), 4.85 (dd, *J* = 10.0 Hz, 6.4 Hz, 1H), 7.28 (t, *J* = 7.2 Hz, 1H), 7.35 (t, *J* = 7.2 Hz, 2H), 7.41 (d, *J* = 7.2 Hz, 2H); ^{13}C NMR ($CDCl_3$, 100 MHz) δ 17.8, 31.1, 36.4, 55.8, 75.7, 80.8, 126.1, 127.6, 128.4, 141.4, 207.5. IR *v* (cm^{-1}): 3060, 3032, 2954, 2925, 2857, 1711, 1378, 1356, 1104, 1022, 759, 700. HRMS (ESI) calcd for $C_{13}H_{17}O_2$ $[M+H]^+$: 205.1223, found 205.1221.

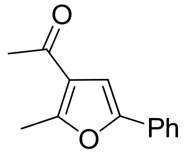

2m'

Prepared according to the general procedure from **1m** (470 mg, 1.47 mmol) and 3.0 eq. DDQ (1.02 g, 4.41 mmol) was used to afford **2m'** as a white solid (184 mg, 0.92 mmol, 63% yield, 12 hours). Mp: 54-55°C. ^{1}H NMR ($CDCl_3$, 400 MHz) δ 2.46 (s, 3H), 2.67 (s, 3H), 6.85 (s, 1H), 7.27-7.31 (m, 1H), 7.38-7.42 (m, 2H), 7.65-7.67 (m, 2H); ^{13}C NMR ($CDCl_3$, 100 MHz) δ 14.5, 29.2, 105.0, 123.4, 123.6, 127.7, 128.7, 129.8, 151.6, 157.9, 194.1. IR *v* (cm^{-1}): 3107, 3061, 2922, 2851, 1674, 1610, 1579, 1555, 1403, 1235, 1070, 950, 760, 692, 632. HRMS (ESI) calcd for $C_{13}H_{13}O_2$ $[M+H]^+$: 201.0910, found 201.0907.

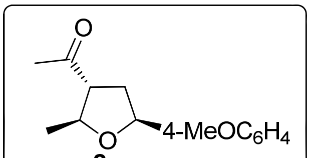

2n

Prepared according to the general procedure from **1n** (310 mg, 0.86 mmol) afforded **2n** as a colorless oil (91 mg, 0.39 mmol, 45% yield, 30 minutes). ^{1}H NMR ($CDCl_3$, 400 MHz) δ 7.35 (d, *J* = 8.4 Hz, 1H), 6.89 (d, *J* = 8.4 Hz, 1H), 4.80 (dd, *J* = 8.8 Hz, 7.2 Hz, 1H), 4.41-4.48 (m, 1H), 3.81 (s, 3H), 3.42-3.51 (m, 1H), 2.25-2.30 (m, 2H), 2.21 (s, 3H), 1.25 (d, *J* = 6.4 Hz, 1H); ^{13}C NMR ($CDCl_3$, 100 MHz) δ 17.8, 31.1, 36.4, 55.3, 55.9, 75.6, 80.6, 113.8, 127.6, 133.3, 159.2, 207.6. IR *v* (cm^{-1}): 2973, 2933, 2838, 1710, 1613, 1514, 1461, 1374, 1248, 1174, 1104, 1034, 832. HRMS (ESI) calcd for $C_{14}H_{18}O_3Na$ $[M+Na]^+$: 257.1148, found 257.1152.

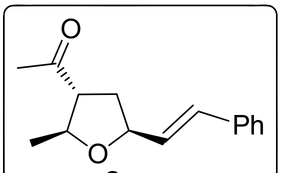

2o

Prepared according to the general procedure from **1o** (220 mg, 0.636 mmol) afforded **2o** as a colorless oil (54 mg, 0.235 mmol, 37% yield, 12 hours). ^{1}H NMR ($CDCl_3$, 400 MHz) δ 1.20 (d, *J* = 6.40 Hz, 3), 2.16-2.21 (m, 5H), 3.37 (dd, *J* = 16.40 Hz, 8.00 Hz, 1H), 4.34-4.41 (m, 1H), 4.47 (dd, *J* = 15.20 Hz, 7.60 Hz, 1H), 6.30 (dd, *J* = 23.20 Hz, 7.20 Hz, 1H), 6.62 (d, *J* = 16.00 Hz, 1H), 7.24-7.26 (m, 1H), 7.30-7.33 (m, 2H), 7.39-7.41 (m, 2H); ^{13}C NMR ($CDCl_3$, 100 MHz) δ 17.7, 31.0, 34.4, 55.9, 75.8, 79.9, 126.6, 127.7, 128.5, 129.5, 131.9 136.5, 207.6. IR *v* (cm^{-1}): 3058, 3026, 2974, 2927, 2861, 1710, 1375, 1353, 1161, 1098, 1070, 968, 906, 749, 694. HRMS (ESI) calcd for $C_{15}H_{19}O_2$ $[M+H]^+$: 231.1380, found 231.1382.

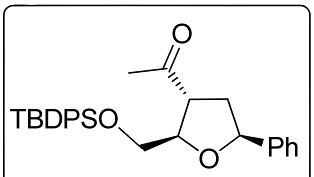

2p

Prepared according to the general procedure from **1p** (532 mg, 0.92 mmol) afforded **2p** as a colorless oil (178 mg, 0.39 mmol, 42% yield, 12 hours). ^{1}H NMR ($CDCl_3$, 400 MHz) δ 7.65-7.68 (m, 4H), 7.32-7.42 (m, 8H), 7.22-7.30 (m, 3H), 4.85 (dd, *J* = 9.8 Hz, 6.2 Hz, 1H), 4.46-4.48 (m, 1H), 3.77-3.78 (m, 2H), 3.53-3.56 (m,1H), 2.30-2.34 (m, 2H), 2.29 (s, 3H), 1.04 (s, 9H); ^{13}C NMR ($CDCl_3$, 100 MHz) δ 19.1,

26.8, 31.9, 36.9, 53.9, 63.7, 80.3, 80.8, 126.1, 127.63, 127.68, 127.70, 128.3, 129.7, 129.7, 132.98, 133.01, 135.58, 135.61, 141.0, 206.5. IR ν (cm^{-1}): 3070, 2955, 2928, 2856, 1714, 1461, 1427, 1362, 1254, 1111, 834, 701, 505. HRMS (ESI) calcd for $C_{29}H_{34}O_3SiNa$ $[M+Na]^+$: 481.2169, found 481.2178.

3. Total syntheses of (-)-brussonol and (-)-przewalskine *E*

3.1 Synthesis of compound 3h

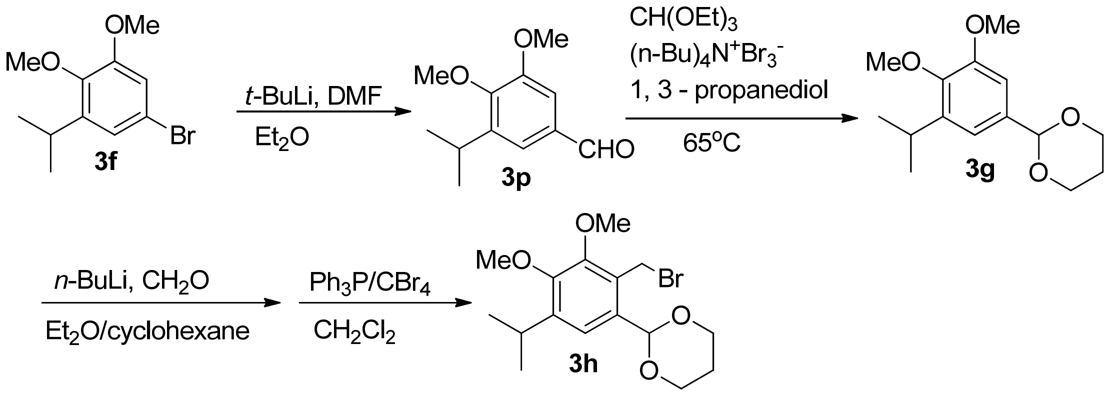

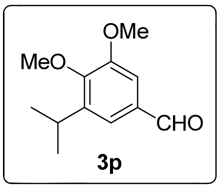

To a solution of **3f** (12.3 g, 47.7 mmol) was added *t*-BuLi (1.30 M, 55 mL, 71.5 mmol) at -78°C, after stirring for 2 hours later at -78°C, DMF (6.0 mL, 76 mmol) was added dropwise and then the mixture was warmed to room temperature spontaneously. Saturated aqueous NH_4Cl (10 mL) was added to quench the reaction, the organic layer was separated and the aqueous layer was extracted with EtOAc/Et_2O (1:1) (3 × 200 mL). The combined organic layer was washed with brine (3 × 30 mL), dried over Na_2SO_4 and concentrated under vacuum. The residue was purified by column chromatography on silica gel (petroleum ether: ethyl acetate = 20:1) to give product **3p** as a colorless oil (8.9 g, 42.8 mmol, 90% yield). ^{1}H NMR ($CDCl_3$, 400 MHz) δ 9.83 (s, 1H), 7.34 (d, J = 1.6 Hz, 1H), 7.25 (d, J = 2.0 Hz, 1H), 3.85 (s, 3H), 3.84 (s, 3H), 3.29-3.36 (m, 1H), 1.19 (d, J = 6.8 Hz, 6H); ^{13}C NMR ($CDCl_3$, 100 MHz) δ 22.9, 26.8, 55.5, 60.6, 108.4, 122.6, 132.3, 142.6, 151.7, 153.0, 191.2. IR ν (cm^{-1}): 2963, 2935, 2872, 2837, 1693, 1583, 1461, 1308, 1139, 1005, 857. HRMS (ESI) calcd for $C_{12}H_{17}O_3$ $[M+H]^+$: 209.1172, found 209.1129.

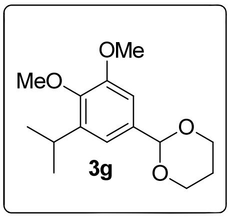

To a 100 mL round bottom flask containing **3p** (16.0 g, 77 mmol) was added 1, 3-propanediol (11 mL, 154 mmol), $CH(OEt)_3$ (9.6 mL, 57.8 mmol), $(n\text{-}Bu)_4N^+Br_3^-$ (222 mg, 0.46 mmol) successively at room temperature. After stirring for 72 hours at 65°C, 50 mL EtOAc was added and the mixture was poured into saturated aqueous $NaHCO_3$ (50 mL). The organic layer was separated and the aqueous layer was extracted with EtOAc/Et_2O (1:1) (3 × 200 mL). The combined organic layer was washed with brine (3 × 30 mL), dried over Na_2SO_4 and concentrated under vacuum. The residue was purified via column chromatography on silica gel (petroleum ether: ethyl acetate = 10:1) to give compound **3g** as a faint yellow solid (17.32 g, 65.1 mmol, 85% yield). Mp: 59-61°C. ^{1}H NMR ($CDCl_3$, 400 MHz) δ 6.95 (s, 1H), 6.94 (s, 1H), 5.45 (s, 1H), 4.25-4.29 (m, 2H), 3.95-4.02 (m, 2H), 3.89 (s, 3H), 3.80 (s, 3H), 3.31-3.38 (m, 1H), 2.19-2.30 (m, 1H), 1.42-1.46 (m, 1H), 1.22 (d, J = 6.8 Hz, 6H); ^{13}C NMR ($CDCl_3$, 100 MHz) δ 23.3, 25.7, 26.9, 55.6, 60.7, 67.3, 101.8, 107.3, 116.2, 134.4, 142.0, 146.6, 152.4. IR ν (cm^{-1}): 2961, 2932, 2851, 1591, 1462, 1387, 1309, 1148, 1107, 1009, 848. HRMS (ESI) calcd for $C_{15}H_{23}O_4$ $[M+H]^+$: 267.1591, found 267.1595.

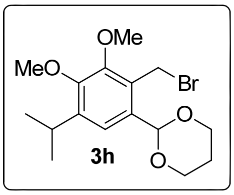

To a solution of **3g** (700 mg, 2.63 mmol) in Et_2O/Cyclohexane (8 mL + 8 mL) was added n-BuLi (2.50 M, 1.36 mL, 3.4 mmol) at 0°C, after stirring at room temperature overnight, CH_2O (*ca.* 0.5 M in THF, 10 mL, *ca.* 5.0 mmol) was added dropwise at -78°C and the mixture was warmed to room temperature spontaneously. Saturated aqueous NH_4Cl (10 mL) was added, the organic layer was separated and the aqueous layer was extracted with EtOAc/Et_2O (1:1) (3 × 50 mL). The combined organic layer was washed with brine (3 × 10 mL), dried over Na_2SO_4 and concentrated under vacuum. The residue was filtered via a short column chromatography and the filtrate was concentrated to give a white solid (483 mg) which was used for the next step directly. To a solution of the above product in CH_2Cl_2 (8 mL) was added Ph_3P (513

mg, 1.96 mmol), CBr_4 (594 mg, 1.80 mmol) successively at 0°C, 30 minutes later, 30 mL Et_2O was added. The mixture was filtered via a short column diatomite and the filtrate was concentrated, the residue was purified by column chromatography on silica gel (petroleum ether: ethyl acetate = 50:1) to give compound **3h** as a white solid (387 mg, 1.08 mmol, 41% yield, two steps). Mp: 94-96°C. ^1H NMR ($CDCl_3$, 400 MHz) δ 7.26 (s, 1H), 5.72 (s, 1H), 4.79 (s, 2H), 4.28-4.32 (m, 2H), 4.01-4.07 (m, 2H), 3.97 (s, 3H), 3.82 (s, 3H), 3.27-3.34 (m, 1H), 2.20-2.33 (m, 1H), 1.46-1.49 (m, 1H), 1.23 (d, J = 6.8 Hz, 6H); ^{13}C NMR ($CDCl_3$, 100 MHz) δ 23.3, 24.6, 25.6, 27.1, 60.4, 60.5, 67.5, 99.6, 127.1, 132.7, 143.4, 150.7, 151.6. IR ν (cm^{-1}): 2962, 2931, 2852, 1602, 1577, 1453, 1377, 1308, 1238, 1115, 1050, 1011, 674. HRMS (ESI) calcd for $C_{16}H_{24}O_4Br$ $[M+H]^+$: 359.0852, found 359.0856.

3.2 Synthesis of compound 3j

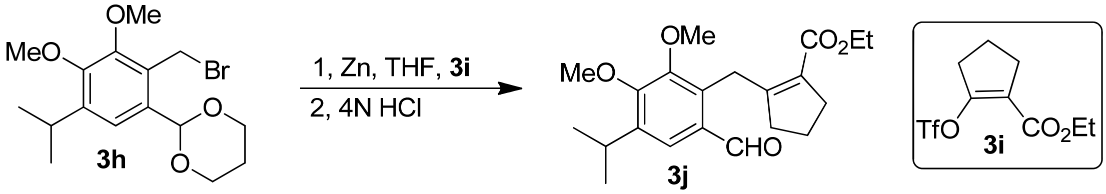

To a suspension of Zn (3.85 g, 59 mmol) in THF (10 mL) was added $Br(CH_2)_2Br$ (0.1 mL, *Cat.*), after refluxing for 10 minutes, the mixture was cooled to 0°C and a solution of **3h** (4.9 g, 13.7 mmol) in THF (20 mL) was added, then the mixture was warmed to room temperature. After stirring for 2 hours, a solution of **3i** (4.69 g, 16.3 mmol) in DMF (50 mL) was added dropwise and the mixture was heated to 90°C. 12 hours later, saturated aqueous NH_4Cl (30 mL) was added. The organic layer was separated and the aqueous layer was extracted with EtOAc (3 × 100 mL). The combined organic layer was washed with brine (3 × 20 mL), dried over Na_2SO_4 and concentrated under vacuum. The residue was dissolved in a mixture of THF (30 mL) and 4N HCl (10 mL), 1 hour later, saturated aqueous $NaHCO_3$ (30 mL) was added slowly. The organic layer was separated and the aqueous layer was extracted with EtOAc (3 × 150 mL). The combined organic layer was washed with brine (3 × 20 mL), dried over Na_2SO_4 and concentrated under vacuum. The residue was purified by column chromatography on silica gel (petroleum ether: ethyl acetate = 20:1) to give product **3j** as a colorless oil (3.39 g, 9.4 mmol, 69% yield). ^1H NMR ($CDCl_3$, 400 MHz) δ 10.17 (s, 1H), 7.54 (s, 1H), 4.48 (s, 2H), 4.24 (q, J = 7.2 Hz, 2H), 3.90 (s, 3H), 3.79 (s, 3H), 3.26-3.33 (m, 1H), 2.61-2.64 (m, 2H), 2.17-2.21 (m, 2H), 1.66-1.73 (m,

2H), 1.32 (t, J = 7.2 Hz, 3H), 1.22 (d, J = 6.8 Hz); ^{13}C NMR ($CDCl_3$, 100 MHz) δ 14.3, 21.1, 23.1, 25.2, 26.9, 33.5, 37.1, 59.8, 60.2, 60.5, 123.5, 127.1, 130.8, 134.0, 141.5, 151.5, 155.7, 157.0, 166.1, 191.7. IR ν (cm^{-1}): 2961, 2871, 1704, 1592, 1458, 1308, 1266, 1211, 1107, 1049, 736. HRMS (ESI) calcd for $C_{21}H_{29}O_5$ $[M+H]^+$: 361.2010, found 361.2010.

3.3 Synthesis of compound 3k

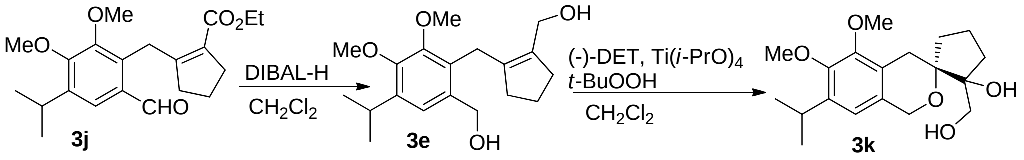

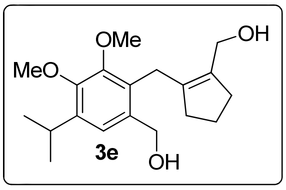

To a solution of **3j** (4.7 g, 13.1 mmol) in CH_2Cl_2 (60 mL) was added DIBAL-H (1.0 M, 58 mL, 58 mmol) at -78°C, then the mixture was warmed to room temperature and saturated aqueous Roche salt (50 mL) was added slowly to quench the reaction, and the resulting mixture was stirred overnight. The mixture was extracted with CH_2Cl_2 (3 × 100 mL) and EtOAc (2 × 100 mL). The combined organic layers were washed with brine, dried over Na_2SO_4 and concentrated under vacuum. The residue was purified by column chromatography on silica gel (petroleum ether: ethyl acetate = 1:1) to give product **3e** as a white solid (3.89 g, 12.2 mmol, 93% yield). Mp: 69-71°C. ^{1}H NMR ($CDCl_3$, 400 MHz) δ 7.94 (s, 1H), 4.60 (s, 2H), 4.23 (s, 2H), 3.82 (s, 6H), 3.61 (s, 2H), 3.25-3.32 (m, 1H), 2.70 (s, 2H), 2.45-2.49 (m, 2H), 2.17-2.20 (m, 2H), 1.71-1.75 (m, 2H), 1.21 (d, J = 6.8 Hz, 6H); ^{13}C NMR ($CDCl_3$, 100 MHz) δ 21.4, 23.5, 25.1, 26.7, 34.8, 36.0, 58.6, 60.2, 60.5, 63.5, 122.4, 129.7, 134.8, 135.1, 138.2, 140.5, 150.2, 151.5. IR ν (cm^{-1}): 3343, 2955, 2873, 2844, 1449, 1409, 1306, 1224, 1052, 1030, 983, 737. HRMS (ESI) calcd for $C_{19}H_{32}O_4N$ $[M+ NH_4]^+$: 338.2326, found 338.2329.

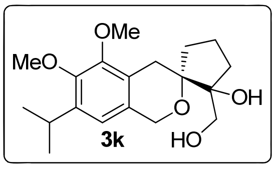

To a 100 mL round bottom flask containing 4Å (600 mg) in CH_2Cl_2 (20 mL) was added (-)-DET (952 μL, 5.6 mmol), Ti(i-PrO)$_4$ (1.41 mL, 4.7 mmol.) successively at

-25°C, 20minutes later, *t*-BuO_2H (5.5 M, 1.7 mL, 9.3 mmol) was added. After stirring for 40 minutes, the mixture was cooled to -50°C and a solution of **3e** (1.0 g, 3.1 mmol) in CH_2Cl_2 (10 mL) was added dropwise. The mixture was stirred for 12 hours at -50°C before it was warmed to room temperature and quenched with saturated aqueous Roche salt (30 mL). After further stirring at room temperature for 6 hours, the mixture was filtered via a short column chromatography on silica gel with EtOAc as eluent, the filtrate was concentrated under vacuum. The residue was purified by column chromatography on silica gel (petroleum ether: ethyl acetate = 1:1) to give product **3k** as a white solid (940 mg, 2.80 mmol, 90% yield, 83% ee). Mp: 96-97°C. $[\alpha]^{24}{}_D$= -23.0 (c= 1.0, $CHCl_3$). ^1H NMR ($CDCl_3$, 400 MHz) δ 6.61 (s, 1H), 4.71 (s, 2H), 4.00 (d, J= 10.8 Hz, 1H), 3.84 (s, 6H), 3.57 (d, J= 11.6 Hz, 1H), 3.26-3.33 (m, 1H), 3.04 (d, J= 16.8 Hz, 1H), 2.91 (brs, 2H), 2.73 (d, J= 16.8 Hz, 1H), 1.64-1.99 (m, 6H), 1.20 (d, J= 6.8 Hz, 3H), 1.19 (d, J= 6.8 Hz, 3H); ^{13}C NMR ($CDCl_3$, 100 MHz) δ 18.6, 23.4, 23.7, 26.1, 26.6, 31.4, 34.0, 60.0, 60.7, 63.2, 66.8, 82.0, 85.8, 116.4, 124.6, 129.2, 140.4, 148.7, 150.8. IR ν (cm^{-1}): 3407, 2957, 2928, 2863, 1589, 1457, 1421, 1308, 1112, 1047, 941, 738. HRMS (ESI) calcd for $C_{19}H_{32}O_5N$ $[M+ NH_4]^+$: 354.2275, found 354.2278. Enantiomeric excess is 83% determined by HPLC (Chiralcel IC, Hexane/Isopropanol 95/5, flow rate = 1.0 mL/min, 230 nm), minor isomer: t_R = 18.02 min; major isomer: t_R = 20.02 min.

3.4 Synthesis of compound 3d

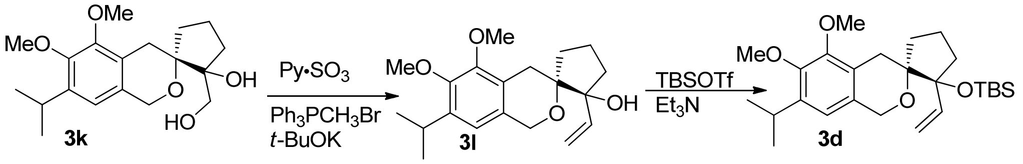

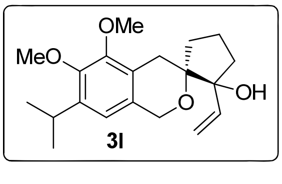

To a solution of **3k** (910 mg, 2.71 mmol) in CH_2Cl_2 (30 mL) was added DMSO (1.92 mL, 27.1 mmol), DIPEA (2.4 mL, 13.5 mmol) and SO_3·Py (1.3 g, 8.13 mmol) successively at room temperature under an argon atmosphere, 30 minutes later, the mixture was poured into aqueous 1N HCl (10 mL). The organic layer was separated and the aqueous layer was extracted with EtOAc (3 × 80 mL). The combined organic layer was washed with brine (3 × 20 mL), dried over Na_2SO_4 and concentrated under vacuum. The residue was used directly without purification. To a solution of

Ph_3PCH_3Br (2.9 g, 8.1 mmol) in toluene (30 mL) was added *t*-BuOK (758 mg, 6.8 mmol) at room temperature, 1 hour later, the mixture became yellow, the solution of above residue in toluene (10 mL) was added to the mixture slowly, 6 hours later, the mixture was poured into saturated aqueous $NaHCO_3$ (20 mL). The organic layer was separated and the aqueous layer was extracted with EtOAc (3 × 80 mL). The combined organic layer was washed with brine (3 × 20 mL), dried over Na_2SO_4 and concentrated under vacuum. The residue was purified via column chromatography on silica gel (petroleum ether: ethyl acetate = 10:1) to give product **3l** as a colorless oil (650 mg, 1.96 mmol, 72% yield, two steps). $[\alpha]^{25}{}_D$ = -37.0 (*c* = 1.0, $CHCl_3$). ^{1}H NMR ($CDCl_3$, 400 MHz) *δ* 6.63 (s, 1H), 6.26 (dd, *J* = 11.0 Hz, 3.0 Hz, 1H), 5.40 (dd, *J* = 17.6 Hz, 1.2 Hz, 1H), 5.23 (dd, *J* = 11.2 Hz, 1.4 Hz, 1H), 4.70 (dd, *J* = 20.4 Hz, 14.80 Hz, 2H), 3.84 (s, 6H), 3.26-3.33 (m, 1H), 2.71 (dd, *J* = 23.6 Hz, 15.2 Hz, 2H), 2.22-2.29 (m, 1H), 2.06-2.11 (m, 1H), 1.66-1.87 (m, 4H), 1.60 (s, 1H), 1.21 (d, *J* = 6.8 Hz, 3H), 1.20 (d, *J* = 7.2 Hz, 3H); ^{13}C NMR ($CDCl_3$, 100 MHz) *δ* 19.8, 23.5, 23.8, 24.9, 26.6, 30.2, 37.8, 59.9, 60.7, 62.3, 84.9, 85.4, 113.7, 116.5, 124.8, 129.9, 140.3, 140.29, 148.5, 150.5. IR *v* (cm^{-1}): 3481, 2957, 2927, 2859, 1481, 1456, 1334, 1110, 1047, 988, 917, 897, 682. HRMS (ESI) calcd for $C_{20}H_{32}O_4N$ $[M+NH_4]^+$: 350.2328, found 350.2329.

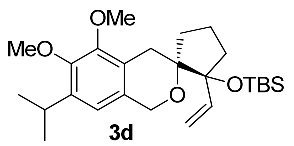

3d

To a solution of **3l** (88 mg, 0.27 mmol) in CH_2Cl_2 (5.0 mL) was added Et_3N (246 μL, 2.16 mmol) and TBSOTf (300 μL, 1.08 mmol) successively at 0°C. The solution was heated for 24 h at 40 °C before it was quenched with saturated aqueous $NaHCO_3$ (5 mL). The aqueous layer was extracted with Et_2O (3 × 30 mL). The combined organic layer was washed with brine (3 × 10 mL), dried over Na_2SO_4 and concentrated under vacuum. The residue was purified by column chromatography on silica gel (petroleum ether: ethyl acetate = 100:1) to give product **3d** as a white solid (116 mg, 0.26 mmol, 98% yield). Mp: 46-48°C. $[\alpha]^{25}{}_D$ = -22.0 (*c* = 1.0, $CHCl_3$). ^{1}H NMR ($CDCl_3$, 400 MHz) *δ* 6.63 (s, 1H), 6.08 (dd, *J* = 17.2 Hz, 10.8 Hz, 1H), 5.37 (dd, *J* = 17.6 Hz, 1.6 Hz, 1H), 5.24 (dd, *J* = 10.8 Hz, 1.6 Hz, 1H), 4.69 (s, 2H), 3.87 (s, 3H), 3.84 (s, 3H), 3.29-3.33 (m, 1H), 2.82 (s, 2H), 2.29-2.32 (m, 1H), 2.01-2.04 (m, 1H), 1.67-1.91 (m, 4H), 1.22 (d, *J* = 6.8 Hz, 3H), 1.21 (d, *J* = 7.2 Hz, 3H), 0.97 (s, 9H), 0.14 (s, 6H); ^{13}C

NMR ($CDCl_3$, 100 MHz) δ -2.7, -2.3, 18.4, 19.5, 23.5, 23.8, 25.6, 25.9, 26.6, 30.8, 34.9, 59.8, 60.8, 62.8, 86.1, 86.7, 115.5, 116.5, 125.7, 130.1, 139.8, 139.9, 148.6, 150.5. IR ν (cm^{-1}): 2956, 2935, 2895, 2858, 1460, 1416, 1361, 1253, 1162, 1111, 1043, 966, 836, 775, 679. HRMS (ESI) calcd for $C_{26}H_{46}O_4SiN$ $[M+NH_4]^+$: 464.3191, found 464.3190.

3.5 Synthesis of compound 3c

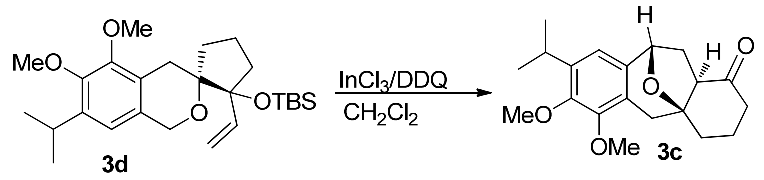

To a solution of **3d** (123 mg, 0.28 mmol) in CH_2Cl_2 (3.0 mL) was added 4Å MS (150 mg), 2, 6-dibromopyridine (331 mg, 5.0 eq.) and $InCl_3$ (7.0 mg, 0.1 eq.) successively at room temperature under an argon atmosphere. After stirring for 15 minutes, DDQ (127 mg, 2.0 eq.) was added. The resulting mixture was stirred for 5 hours at room temperature before it was filtered via a short silica gel column with petroleum ether: ethyl acetate = 4:1 as elute to remove the 4Å MS and 2, 6-dibromopyridine, and the filtrate was concentrated under vacuum. The residue was chromatographed via column on silica gel (petroleum ether: ethyl acetate = 10:1) to afford compound **3c** as a colorless oil (75 mg, 0.23 mmol, 82% yield). $[\alpha]^{17}_{D}$ = -48.0 (c = 1.0, $CHCl_3$). ^1H NMR ($CDCl_3$, 400 MHz) δ 6.66 (s, 1H), 4.99 (d, J = 6.8 Hz, 1H), 3.84 (s, 3H), 3.81 (s, 3H), 3.26-3.33 (m, 1H), 2.92 (d, J = 17.2 Hz, 1H), 2.78 (d, J = 16.8 Hz, 1H), 2.65-2.70 (m, 1H), 2.45-2.57 (m, 2H), 2.21-2.37 (m, 2H), 2.04-2.15 (m, 2H), 1.90-1.99 (m, 2H), 1.20 (d, J = 6.8 Hz, 6H); ^{13}C NMR ($CDCl_3$, 100 MHz) δ 18.4, 23.5, 23.6, 26.6, 34.4, 37.7, 39.2, 40.6, 54.6, 59.6, 60.5, 76.5, 81.7, 116.6, 122.6, 136.2, 140.7, 149.0, 150.7, 212.2. IR ν (cm^{-1}): 2956, 2927, 2870, 1706, 1456, 1330, 1048, 1002, 891, 798. HRMS (ESI) calcd for $C_{20}H_{30}O_4N$ $[M+NH_4]^+$: 348.2169, found 348.2173.

3.6 Synthesis of compound 3n

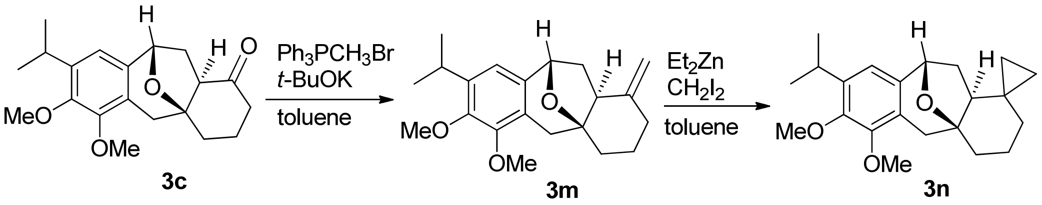

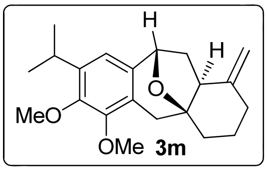

To a solution of Ph_3PCH_3Br (214 mg, 0.6 mmol) in toluene (3.0 mL) was added *t*-BuOK (56 mg, 0.5 mmol) at room temperature under an argon atmosphere. 1 hour later, the mixture became yellow, then a solution of **3c** (66 mg, 0.2 mmol) in toluene (2.0 mL) was added dropwise to the mixture. After further stirring for 10 minutes, the mixture was poured into saturated aqueous $NaHCO_3$ (10 mL). The mixture was extracted with Et_2O (3 × 30 mL). The combined organic layer was washed with brine (3 × 10 mL), dried over Na_2SO_4, concentrated under vacuum and the residue was chromatographed via column on silica gel (petroleum ether: ethyl acetate = 20:1) to afford product **3m** as a colorless oil (58 mg, 0.18 mmol, 88% yield). $[\alpha]^{17}{}_D$= -57.0 (*c* = 1.0, $CHCl_3$). ^1H NMR ($CDCl_3$, 400 MHz) δ 6.66 (s, 1H), 4.99 (d, *J* = 5.2 Hz, 1H), 4.71 (d, *J* = 12.0 Hz, 2H), 3.84 (s, 3H), 3.83 (s, 3H), 3.26-3.33 (m, 1H), 2.80 (d, *J* = 16.8 Hz, 1H), 2.75 (t, *J* = 8.4 Hz, 1H), 2.64 (d, *J* = 16.8 Hz, 1H), 2.43-2.46 (m, 1H), 2.13-2.22 (m, 3H), 1.86-2.00 (m, 2H), 1.64-1.70 (m, 2H), 1.22 (d, *J* = 6.8 Hz, 3H), 1.21 (d, *J* = 6.8 Hz, 3H); ^{13}C NMR ($CDCl_3$, 100 MHz) δ 17.9, 23.5, 23.7, 26.7, 26.9, 31.4, 38.0, 43.4, 48.4, 59.7, 60.6, 76.9, 81.8, 109.9, 116.8, 123.6, 136.6, 140.3, 148.7, 149.0, 150.9. IR ν (cm^{-1}):2928, 2868, 1645, 1582, 1455, 1414, 1328, 1163, 1089, 888, 739. HRMS (ESI) calcd for $C_{21}H_{29}O_3$ $[M+H]^+$: 329.2111, found 329.2114.

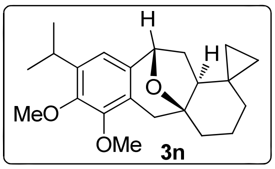

To a solution of **3m** (58 mg, 0.18 mmol) in toluene (5.0 mL) was added CH_2I_2 (150 μL, 1.8 mmol) and Et_2Zn (1.0 M, 900 μL, 0.9 mmol) successively at room temperature under an argon atmosphere. The resulting mixture was warmed to 50°C and stirred for 8 hours, then saturated aqueous NH_4Cl (5 mL) was added. The mixture was extracted with Et_2O (3 × 30 mL). The combined organic layer was washed with brine (3 × 10 mL), dried over Na_2SO_4, concentrated under vacuum and the residue was chromatographed via column on silica gel (petroleum ether: ethyl acetate = 20:1) to afford compound **3n** as a white solid (35 mg, 0.102 mmol, 58% yield). Mp: 60-62°C. $[\alpha]^{18}{}_D$= -68.0 (*c* = 1.0, $CHCl_3$). ^1H NMR ($CDCl_3$, 400 MHz) δ 6.64 (s, 1H), 4.96 (d, *J* = 6.4 Hz, 1H), 3.82 (s, 3H), 3.81 (s, 3H), 3.26-3.29 (m, 1H), 2.80 (d, *J* =

16.8 Hz, 1H), 2.52 (d, J = 16.8 Hz, 1H), 2.17-2.22 (m, 1H), 1.86-2.02 (m, 5H), 1.63-1.71 (m, 1H), 1.50 (t, J = 8.0 Hz, 1H), 1.20 (d, J = 6.8 Hz, 6H), 0.75-0.81 (m, 1H), 0.40-0.43 (m, 1H), 0.24-0.27 (m, 1H), 0.17-0.20 (m, 2H); ^{13}C NMR ($CDCl_3$, 100 MHz) δ 8.5, 13.8, 16.3, 17.9, 23.5, 23.7, 25.8, 26.7, 30.3, 38.6, 40.8, 49.4, 59.6, 60.6, 80.6, 116.8, 123.7, 137.1, 140.2, 148.8, 150.8. IR ν (cm^{-1}): 2955, 2925, 2864, 1732, 1455, 1328, 1263, 1090, 1047, 798, 739. HRMS (ESI) calcd for $C_{22}H_{31}O_3$ [M+H]$^+$: 343.2268, found 343.2266.

3.7 Synthesis of compound 3o

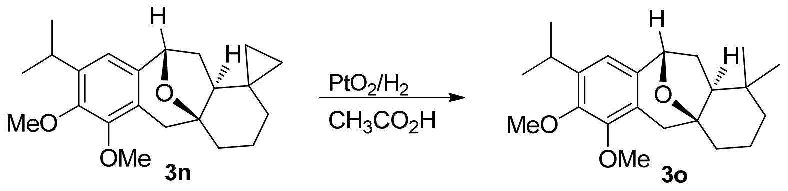

To a stirred solution of **3n** (15 mg, 0.044 mmol) in CH_3CO_2H (2.0 mL) was added PtO_2 (5.1 mg, 0.022 mmol) at room temperature under a hydrogen atmosphere (1 atm). After stirring at 60°C for 10 hours, the solution was filtered via a short column chromatography and the filtrate was concentrated under vacuum. The residue was purified via column chromatography on silica gel (petroleum ether: ethyl acetate = 20:1) to afford compound **3o** as a white solid (13.5 mg, 0.039 mmol, 89% yield). Mp: 68-70°C. $[\alpha]^{18}_{D}$ = -57.0 (c = 1.0, $CHCl_3$). ^{1}H NMR ($CDCl_3$, 400 MHz) δ 6.64 (s, 1H), 4.86 (d, J = 6.8 Hz, 1H), 3.83 (s, 3H), 3.82 (s, 3H), 3.25-3.30 (m, 1H), 2.79 (d, J = 16.8 Hz, 1H), 2.50 (m, J = 17.2 Hz, 1H), 2.09-2.16 (m, 1H), 2.00-2.02 (m, 1H), 1.89-1.97 (m, 1H), 1.78-1.87 (m, 3H), 1.61-1.67 (m, 1H), 1.49-1.56 (m, 1H), 1.21 (d, J = 6.8 Hz, 3H), 1.20 (d, J = 6.8 Hz, 3H), 1.19-1.20 (m, 1H), 0.97 (s, 3H), 0.85 (s, 3H); ^{13}C NMR ($CDCl_3$, 100 MHz) δ 16.2, 23.5, 23.8, 26.6, 26.7, 30.57, 30.60, 31.8, 32.2, 39.4, 39.6, 50.9, 59.7, 60.6, 76.0, 80.1, 116.4, 124.1, 137.8, 140.0, 148.8, 150.9. IR ν (cm^{-1}): 2949, 2868, 1455, 1327, 1095, 1047, 1009, 737. HRMS (ESI) calcd for $C_{22}H_{36}O_3N$ [M+NH_4]$^+$: 362.2690, found 362.2696.

3.8 Syntheses of (-)-brussonol and (-)-przewalskine *E*

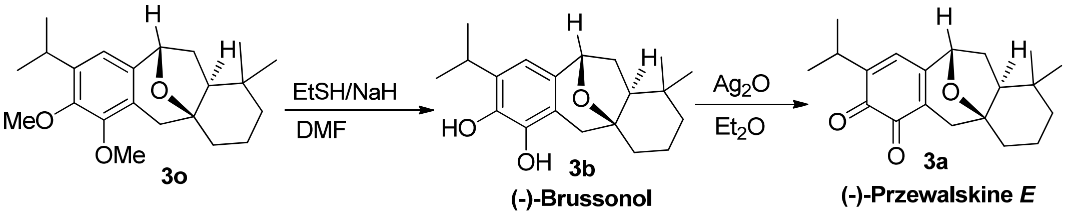

To a 10 mL round bottom flask containing NaH (70 mg, 1.74 mmol) in DMF (2.5 mL) was added EtSH (268 μL, 3.61 mmol) dropwise at 0°C under an argon atmosphere, then a solution of **3o** (50 mg, 0.145 mmol) in DMF (1.0 mL) was added. The resulting solution was heated for 12 h at 150°C before it was quenched with saturated aqueous NH_4Cl (5 mL) at room temperature. The mixture was extracted with EtOAc (3 × 30 mL). The combined organic layer was washed with brine (3 × 10 mL), dried over Na_2SO_4, concentrated under vacuum and the residue was chromatographed via column on silica gel (petroleum ether: ethyl acetate = 5:1) to give product **3b** as a yellow foam (35 mg, 0.111 mmol, 76% yield). $[\alpha]^{19}{}_D$ = -42.0 (*c* = 1.0, $CHCl_3$). ^{1}H NMR ($CDCl_3$, 400 MHz) δ 6.46 (s, 1H), 4.99 (s, 1H), 4.97 (s, 1H), 4.86 (d, *J* = 6.4 Hz, 1H), 3.08-3.15 (m, 1H), 2.74 (d, *J* = 16.4 Hz, 1H), 2.40 (d, *J* = 16.4 Hz, 1H), 2.10-2.16 (m, 1H), 1.98-2.04 (m, 1H), 1.88-1.93 (m, 1H), 1.77-1.87 (m, 3H), 1.59-1.66 (m, 1H), 1.49-1.57 (m, 1H), 1.26 (d, *J* = 6.8 Hz, 3H), 1.25 (d, *J* = 6.8 Hz, 3H), 1.23-1.24 (m, 1H), 0.97 (s, 3H), 0.85 (s, 3H); ^{13}C NMR ($CDCl_3$, 100 MHz) δ 16.1, 22.6, 22.7, 26.6, 27.1, 30.6, 30.6, 31.8, 32.1, 38.8, 39.7, 51.0, 76.2, 80.2, 112.7, 116.5, 132.0, 134.1, 139.5, 141.6. IR ν (cm^{-1}): 3360, 2954, 2925, 2869, 1454, 1319, 1270, 1163, 1090, 1019, 737. HRMS (ESI) calcd for $C_{20}H_{32}O_3N$ $[M+NH_4]^+$: 334.2377, found 334.2380.

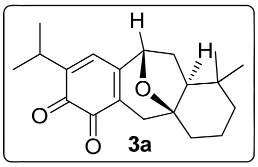

To a solution of **3b** (17 mg, 0.054 mmol) in Et_2O (2.0 mL) was added Ag_2O (36 mg, 0.156 mmol) at room temperature. After the starting material disappeared, the mixture was filtered via a short silica gel column with Et_2O as eluent and the filtrate was concentrated under vacuum. The residue was purified by column chromatography on silica gel (petroleum ether: ethyl acetate = 15:1) to give product **3a** as a brown foam (12 mg, 0.0382 mmol, 71% yield). $[\alpha]^{18}{}_D$ = -57.0 (*c* = 1.0, $CHCl_3$). ^{1}H NMR ($CDCl_3$, 400 MHz) δ 6.47 (s, 1H), 4.48 (d, *J* = 6.4 Hz, 1H), 2.92-2.99 (m, 1H), 2.49 (d, *J* = 18.4 Hz, 1H), 2.18 (d, *J* = 18.8 Hz, 1H), 2.03-2.14 (m, 2H), 1.93-2.00 (m, 1H), 1.73-1.81 (m, 3H), 1.58-1.65 (m, 1H), 1.46-1.53 (m, 1H), 1.16-1.19 (m, 1H), 1.12 (d,

J = 6.8 Hz, 3H), 1.11 (d, *J* = 6.8 Hz, 3H), 0.97 (s, 3H), 0.86 (s, 3H); ^{13}C NMR ($CDCl_3$, 100 MHz) δ 15.8, 21.5, 21.6, 26.7, 27.3, 29.8, 30.3, 31.6, 32.0, 37.9, 38.1, 51.5, 74.9, 80.4, 129.5, 131.9, 147.9, 152.9, 179.7, 180.6. IR ν (cm^{-1}): 2954, 2924, 2858, 1659, 1460, 1252, 1165, 1121, 1045, 742. HRMS (ESI) calcd for $C_{20}H_{30}O_3N$ $[M+NH_4]^+$: 332.2220, found 332.2223.

Supplementary References

1. Zhu, S.-F., Song, X.-G., Li, Y., Cai, Y., Zhou, Q.-L. *J. Am. Chem. Soc.* **132**, 16374-16376, (2010).
2. Simmons, E. M., Yen, J. R. & Sarpong, R. Reconciling Icetexane Biosynthetic Connections with Their Chemical Synthesis: Total Synthesis of (±)-5, 6-Dihydro-6α-hydroxysalviasperanol, (±)-Brussonol and (±)-Abrotanone. *Org. Lett.* **9**, 2705-2708, (2007).
3. Xu, G., Peng, L.-Y., Tu, L., Li, X.-L., Zhao, Y., Zhang, P.-T. & Zhao, Q.-S. Three New Diterpenoids from Salvia przewalskiiMaxim. *Helv. Chim. Acta* **92**, 409-413, (2009).
